# Dithienylethene-Based Photoswitchable Phosphines for the Palladium-Catalyzed Stille Coupling Reaction

**DOI:** 10.1021/acs.inorgchem.3c04423

**Published:** 2024-04-16

**Authors:** Anastasiia Sherstiuk, Agustí Lledós, Peter Lönnecke, Jordi Hernando, Rosa María Sebastián, Evamarie Hey-Hawkins

**Affiliations:** † Faculty of Chemistry and Mineralogy, Institute of Inorganic Chemistry, Leipzig University, Johannisallee 29, D-04103 Leipzig, Germany; ‡ Department of Chemistry, Universitat Autònoma de Barcelona, Cerdanyola del Vallès, Bellaterra 08193, Barcelona, Spain; § Centro de Innovación en Química Avanzada (ORFEO−CINQA), Universitat Autònoma de Barcelona, Cerdanyola del Vallès, Bellaterra 08193, Barcelona,Spain

## Abstract

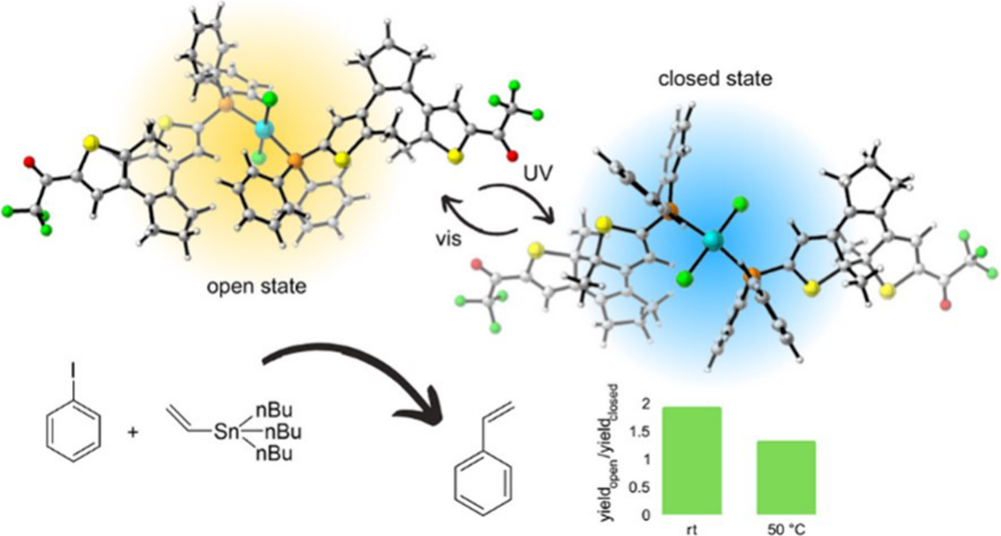

Homogeneous transition metal catalysis is a constantly developing field in chemical sciences. A growing interest in this area is photoswitchable catalysis, which pursues *in situ* modulation of catalyst activity through noninvasive light irradiation. Phosphorus ligands are excellent targets to accomplish this goal by introducing photoswitchable moieties; however, only a limited number of examples have been reported so far. In this work, we have developed a series of palladium complexes capable of catalyzing the Stille coupling reaction that contain photoisomerizable phosphine ligands based on dithienylethene switches. Incorporation of electron-withdrawing substituents into these dithienylethene moieties allows variation of the electron density on the phosphorus atom of the ligands upon light irradiation, which in turn leads to a modulation of the catalytic properties of the formed complexes and their activity in a model Stille coupling reaction. These results are supported by theoretical computations, which show that the energy barriers for the rate-determining steps of the catalytic cycle decrease when the photoswitchable phosphine ligands are converted to their closed state.

## Abstract 2

Two photoisomerizable phosphine ligands were synthesized based on dithienylethenes, whose electronic communication with strong electron-withdrawing groups was switched on and off upon light irradiation. As a result, the σ-donating ability of these phosphines can be efficiently light-controlled. This behavior was transferred to palladium(II) catalytic complexes and exploited to photomodulate the reaction rate of a model Stille reaction

## Introduction

Inspired by the dynamic behavior of enzymes in nature, controlling the operation of artificial molecular catalysts with external stimuli has become an active area of research with potential application in a variety of fields^1−3^ (e.g., polymer synthesis,^4,5^ bioorthogonal chemistry,^6,7^ and additive manufacturing^8^). The use of light to achieve this goal holds great promise, as it opens the way to catalyst regulation on-demand with high selectivity and spatiotemporal precision.^9−11^ A major strategy toward this purpose is the design of photoswitchable catalysts, whose activity and selectivity can be reversibly modulated with light by installing photochromic units into their structure, i.e., light-responsive moieties, such as azobenzenes,^12,13^ dithienylethenes^14^ and stilbenes,^12^ which alter the reactivity of nearby catalytic sites by photoisomerizing between two different states.

Because of their fundamental role in modern synthetic chemistry, transition metal complexes are among the principal targets of photoswitchable catalysis.^15−17^ Reversible light control of the catalytic activity of these compounds is generally accomplished by introducing photochromic ligands (e.g., photoswitchable phosphines^18^). In most of the cases, the geometrical changes that these ligands undergo upon photoisomerization cause catalyst reactivity modulation,^15−17^ for instance, by distorting the structure around the catalytic site^19−21^ or varying the separation distance between two cooperative active metal centers.^22,23^ However, the actual impact of these effects on catalytic activity and selectivity can be detrimentally affected by catalyst conformational flexibility^24^ and be dependent on substrate size and geometry. An alternative, less exploited approach to light-control the performance of transition metal catalysts is to capitalize on the electronic changes that occur upon ligand photoisomerization.^15−17^ For this strategy, dithienylethenes (DTEs) are the photochromic units of choice^14^ because, in contrast to azobenzenes and stilbenes, they undergo a large modification in electronic structure when reversibly toggling between their ring-open (**o**) and ring-closed (**c**) isomers.^25^

**Scheme 1:**
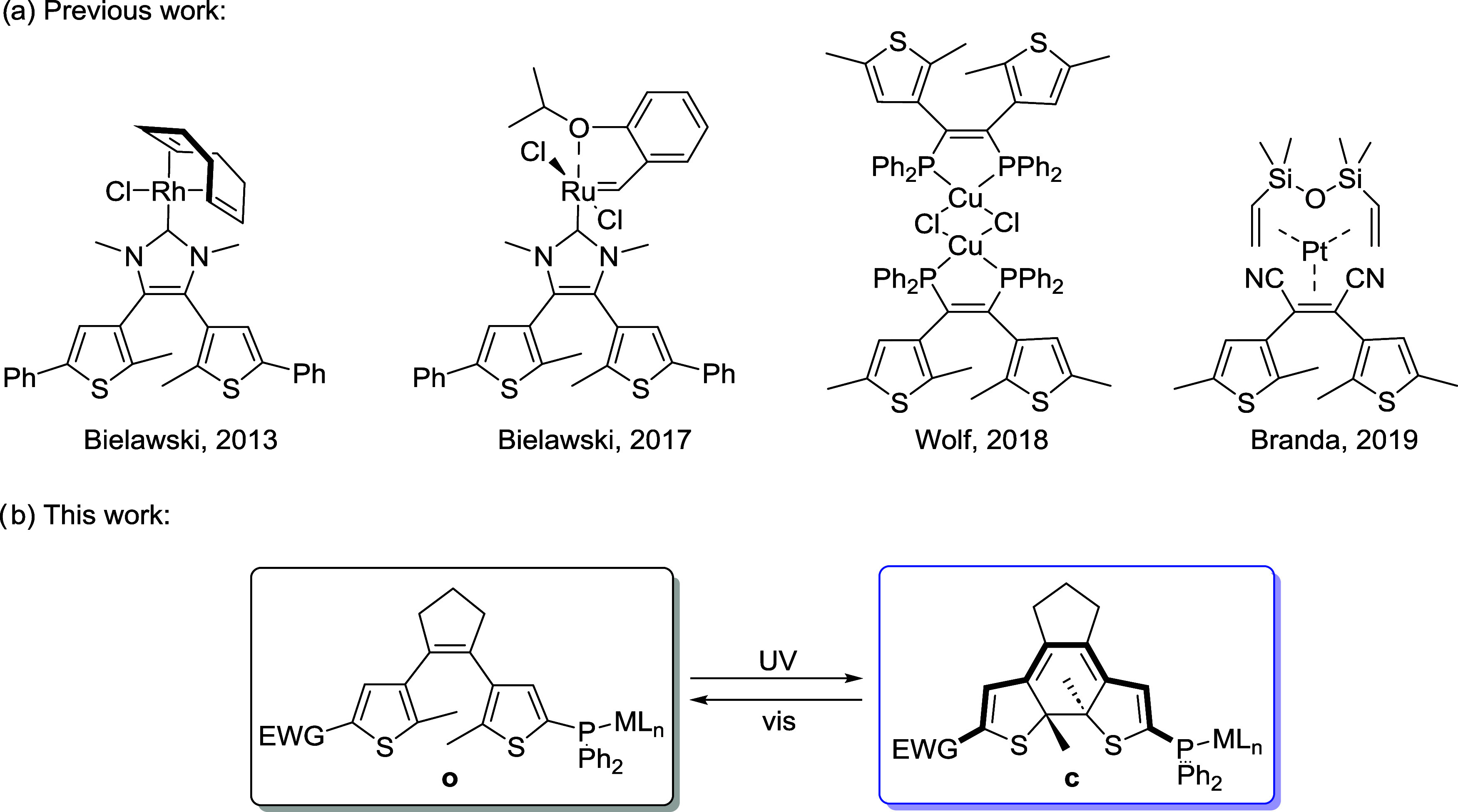
(a) Previously Reported Transition Metal Complexes Incorporating DTE-Based Photoswitchable Units as Ligands and (b) DTE-Based Photoswitchable Complexes Studied in This Work

To date, only a small number of DTE-based complexes have been described where photomodulation of catalysis is accomplished via electronic effects.^26−29^ All of these systems share a common design principle: they contain DTE ligands that bind to the metal center through the groups installed in their central ethylene bridging moiety (e.g., carbene,^26,27^ phosphines^28^), which are either removed^29^ or lose electron density^26−28^ upon ring-closing (Scheme 1a). In contrast, other electronic features that accompany DTE photoisomerization have yet to be exploited in metal-based photoswitchable catalysis. In this work, we proposed to explore one of these additional features: the variation in electronic communication between the external thiophene substituents upon photoconversion, which has already been utilized to control chemical reactivity with light-responsive organocatalysts^30,31^ and organic substrates.^32,33^ In particular, herein, we devised the synthesis of nonsymmetric DTE derivatives as photoswitchable ligands bearing (i) a metal-binding phosphine group at one thiophene ring and (ii) an electron-withdrawing substituent at the other (Scheme 1b). As these two groups must be electronically insulated in the open state of the system and become selectively conjugated upon ring-closing, our molecular design should allow modulating the electron density of the phosphine ligand with light and, eventually, the catalytic activity of metal centers upon coordination. To validate this hypothesis, we prepared palladium(II) complexes of our DTE ligands and tested them as precatalysts in a Stille coupling reaction, a widely employed Pd-catalyzed transformation that is sensitive to electronic effects^34−36^ and has not been tested yet in photoswitchable catalysis.

## Results and Discussion

### Synthesis of Photoswitchable Ligands and Monometallic Complexes

To explore our approach toward photoswitchable catalysts, two different electron-withdrawing groups (EWGs) were introduced in DTE-based phosphine ligands: (i) a trifluoroacetyl group in **DTE-COCF**_**3**_, which is a strong EWG according to its Hammett σ-*meta* and σ-*para* substituent constants (σ_m_ = 0.63, σ_p_ = 0.80),^37^ and (ii) a pentafluorophenyl group in **DTE-C**_**6**_**F**_**5**_, which presents a less pronounced electron-withdrawing power (σ_m_ = 0.26, σ_p_ = 0.27).^37^ As a reference, we also considered the preparation of a DTE-based phosphine ligand bearing a phenyl substituent (**DTE-Ph**), which should impart very minor electronic effects (σ_m_ = 0.06, σ_p_ = −0.01).^37^

**Scheme 2:**
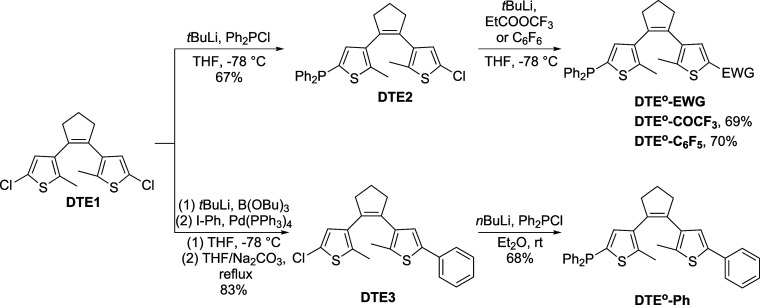
Synthetic Route toward Phosphanyl-Substituted DTEs

The ring-open isomer of the DTE-based ligands was synthesized through asymmetrical stepwise functionalization of 1,2-bis(2-chloro-5-methylthien-4-yl)cyclopentene (**DTE1**, Scheme 2), which is a common precursor for the preparation of dithienylethene derivatives via lithiation-mediated processes.^38^ For **DTE**^**o**^**-COCF**_**3**_ and **DTE**^**o**^**-C**_**6**_**F**_**5**_, the sequence to introduce the phosphine and EWG groups in **DTE1** was governed by their sensitivity toward lithium reagents. For this reason, we first conducted lithiation of **DTE1** with *t*-butyllithium (*t*BuLi), followed by quenching with chlorodiphenylphosphine to obtain phosphanyl compound **DTE2** (67% yield); next, additional lithiation with *t*BuLi followed by the reaction with ethyl trifluoroacetate (69% yield) or hexafluorobenzene (70% yield) furnished **DTE**^**o**^**-COCF**_**3**_ and **DTE**^**o**^**-C**_**6**_**F**_**5**_, respectively. As for **DTE**^**o**^**-Ph**, we inverted the order in which the external thiophene substituents were introduced to the DTE core according to a previously reported procedure.^39^ In particular, its phenyl side group was first installed through consecutive lithiation, borylation, and Suzuki coupling steps to produce compound **DTE3** (83% yield), which was then subjected to further lithiation with *n*-butyllithium (*n*BuLi) and subsequent treatment with chlorodiphenylphosphine (68% yield) to introduce the phosphanyl moiety of **DTE**^**o**^**-Ph**. All synthesized phosphines presented a singlet in the ^31^P{^1^H} NMR spectra at around δ ≈ – 19.6 ppm, a chemical shift value that is similar to those reported for (2-methyl-5-thienyl)diphenylphosphine (δ = −21.9 ppm)^40^ and symmetric bis(phosphine) DTE (δ ≈ – 20.0 ppm).^41,42^ As expected, this result corroborates that the phosphanyl and electron-modulating groups in the nonplanar structure of **DTE**^**o**^**-COCF**_**3**_, **DTE**^**o**^**-C**_**6**_**F**_**5**_, and **DTE**^**o**^**-Ph** are not conjugated and, therefore, do not significantly affect each other.

**Figure 1:**
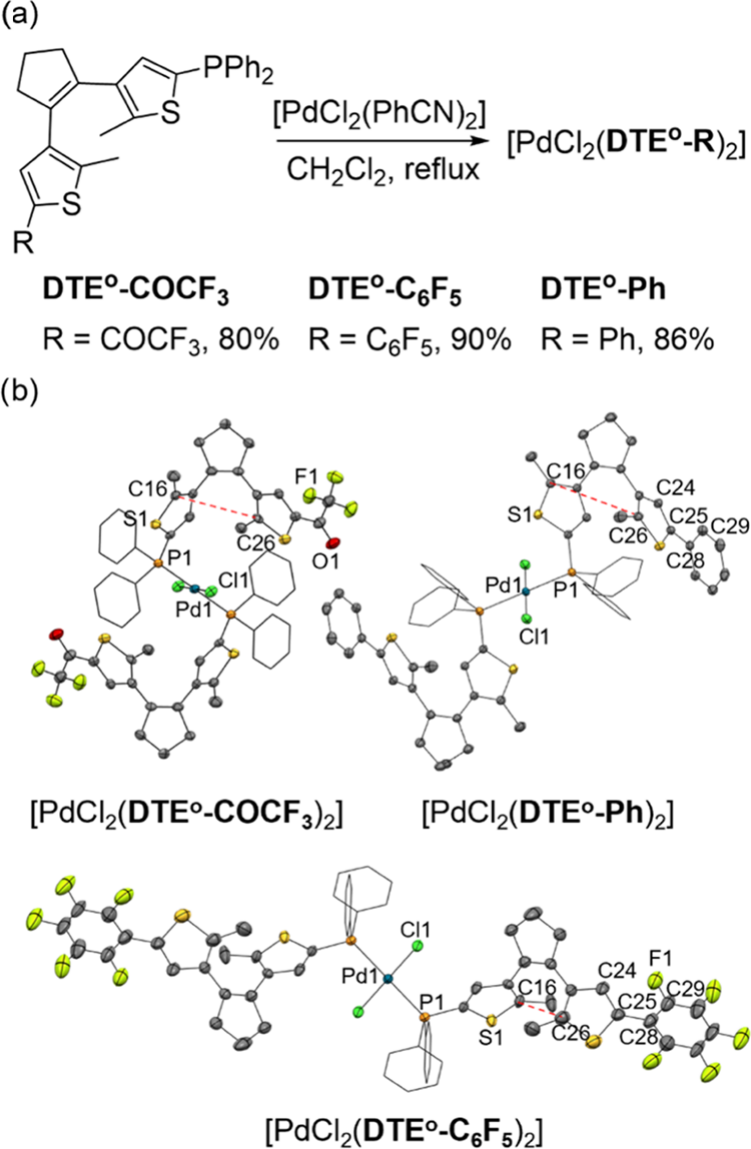
(a) Synthesis of [PdCl_2_(**DTE**^**o**^**-COCF**_**3**_)_2_], [PdCl_2_(**DTE**^**o**^**-C**_**6**_**F**_**5**_)_2_], and [PdCl_2_(**DTE**^**o**^**-Ph**)_2_]. (b) Molecular structures of [PdCl_2_(**DTE**^**o**^**-Ph**)_2_], [PdCl_2_(**DTE**^**o**^**-COCF**_**3**_)_2_], and [PdCl_2_(**DTE**^**o**^**-C**_**6**_**F**_**5**_)_2_]. Thermal ellipsoids are set at the 50% probability level. For clarity, P-bound phenyl rings are depicted in wireframe style, and cocrystallized solvent and hydrogen atoms are omitted. Distances between the reactive carbon atoms in DTE photoisomerization are marked with dashed red line.

When two equivalents of the phosphine ligands were used to react with *trans*-[PdCl_2_(PhCN)_2_], monometallic palladium(II) 1:2 type complexes were formed (Figure 1a). For all of these compounds, an isolated singlet was registered in their ^31^P{^1^H} NMR spectra that shifted downfield to δ ≈ 12 ppm, thereby corroborating metal complexation and the formation of exclusively one isomer (*cis* or *trans*). In addition, negligible differences were found in the chemical shift of this ^31^P{^1^H} NMR signal for the three Pd complexes prepared, which again demonstrates the lack of electronic communication between the external thiophene substituents in the open isomers of the ligands. Furthermore, complexation to the Pd center could also be verified by the downfield shift of the ^1^H NMR resonance of the thiophene ring proton next to the phosphanyl group. Single crystals suitable for X-ray structure determination were obtained for the three complexes, which revealed that the Pd complexes have a square-planar geometry with *trans* orientation of the phosphine ligands (Figure 1b, Figures S1–S3 and Table S1). The main difference observed between the crystal structures of these compounds was the conformation of their DTE ligands. In [PdCl_2_(**DTE**^**o**^**-Ph**)_2_] and [PdCl_2_(**DTE**^**o**^**-COCF**_**3**_)_2_], the ligands are locked in a distorted parallel open-state conformation with a distance of 4.31 and 5.19 Å, respectively, between the carbon atoms that should react upon ring-closing photoisomerization (C_16_–C_26_). As previously described in the literature,^25^ this type of conformation cannot undergo the light-induced conrotatory electrocyclization reaction to produce the corresponding closed isomer and, consequently, no photochromism was observed in the solid state for [PdCl_2_(**DTE**^**o**^**-Ph**)_2_] and [PdCl_2_(**DTE**^**o**^**-COCF**_**3**_)_2_]. Contrarily, in the case of [PdCl_2_(**DTE**^**o**^**-C**_**6**_**F**_**5**_)_2_], the DTE ligands are present in an antiparallel open-state conformation with a shorter distance between the reactive carbons (C_16_–C_26_, 3.69 Å), two structural features that are compatible with the photoinduced ring-closing reaction.^43^ Indeed, irradiation at 312 nm yielded a color change from yellow to red, and the process was reversed with irradiation at 520 nm, thus demonstrating solid-state photoswitching for [PdCl_2_(**DTE**^**o**^**-C**_**6**_**F**_**5**_)_2_] (Figure S4).

### Photochemical Behavior of Ligands and Complexes

**Scheme 3:**
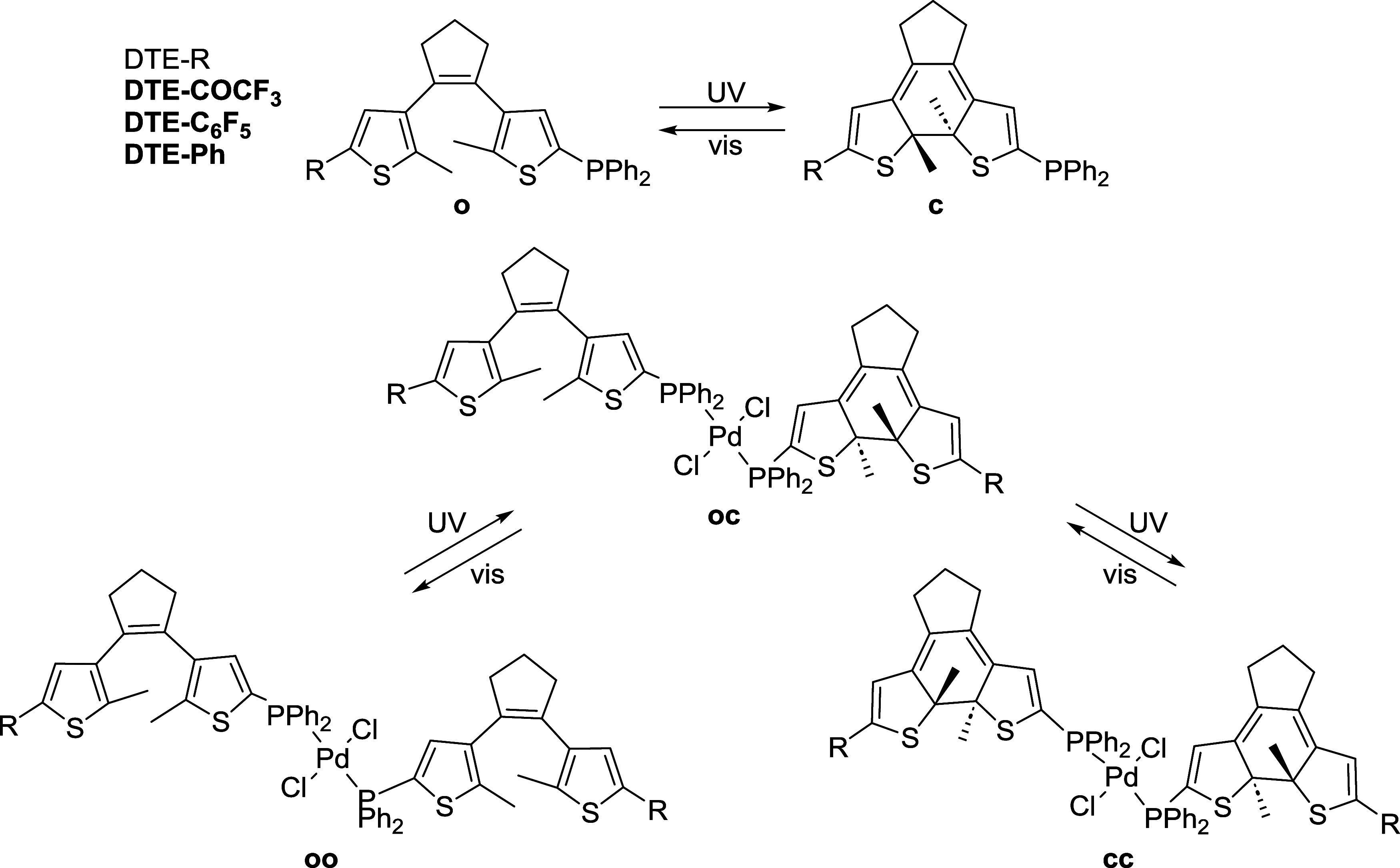
Photoisomerization Processes for DTE-Based Free Ligands and Their Palladium(II) Complexes

Irrespective of their behavior in the solid state, all of the DTE-based ligands and complexes prepared should photoisomerize in solution upon irradiation. However, while **DTE-COCF**_**3**_, **DTE-C**_**6**_**F**_**5**_, and **DTE-Ph** should just photoconvert between their ring-open and ring-closed isomers, a more complex situation is expected for their complexes (Scheme 3). Because of their 2:1 phosphine/metal stoichiometry, these compounds must toggle between three different states, where DTE-based ligands are both ring-open (**oo**), one of them ring-open and the other ring-closed (**oc**), or both ring-closed (**cc**).

**Figure 2:**
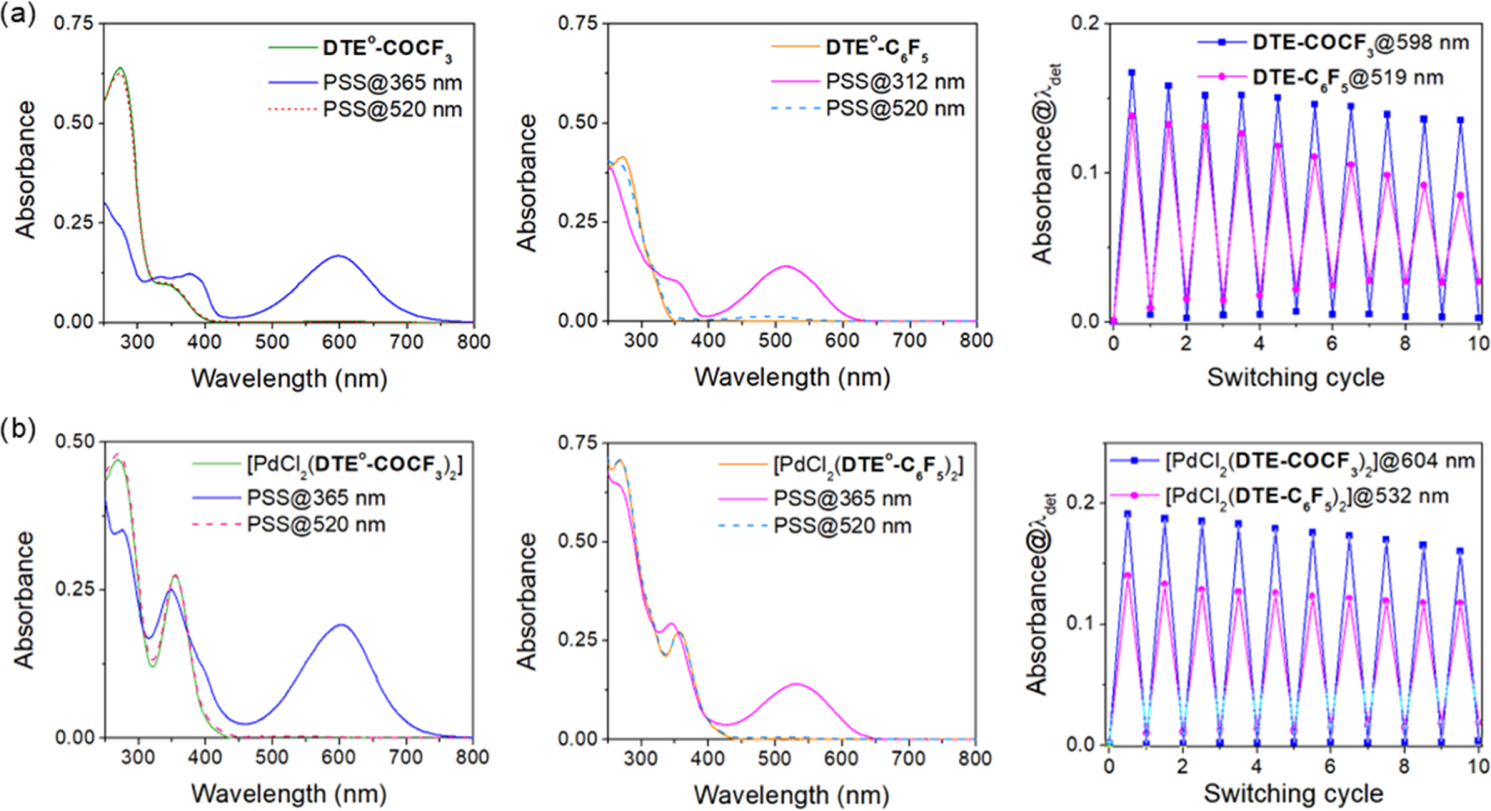
(a) Variation of the absorption spectrum of free ligands **DTE**^**o**^**-COCF**_**3**_ (*c* = 1.5 × 10^–5^ M) and **DTE**^**o**^**-C**_**6**_**F**_**5**_ (*c* = 1.5 × 10^–5^ M) in cyclohexane upon sequential irradiation with UV (λ_exc_ = 365 for 90 s, or λ_exc_ = 312 nm for 110 s) and green light (λ_exc_ = 520 nm for 120–160 s) until the corresponding ring-closing and ring-opening photostationary states (PSSs) are obtained, respectively. The variation of the absorbance at the spectral maximum of the ring-closed isomer of these compounds (λ_det_ = 598 or 519 nm) upon 10 consecutive photoswitching cycles under the same concentration and irradiation conditions is also given. (b) Variation of the absorption spectrum of the open-state complexes [PdCl_2_(**DTE**^**o**^**-COCF**_**3**_)_2_] (*c* = 1.5 × 10^–5^ M) and [PdCl_2_(**DTE**^**o**^**-C**_**6**_**F**_**5**_)_2_] (*c* = 1.5 × 10^–5^ M) in cyclohexane upon sequential irradiation with UV (λ_exc_ = 365 nm for 90 and 150 s, respectively) and green light (λ_exc_ = 520 nm for 180 s) until the corresponding ring-closing and ring-opening PSSs are obtained, respectively. The variation of the absorbance at the spectral maximum of the ring-closed isomer of these compounds (λ_det_ = 604 or 532 nm) upon 10 consecutive photoswitching cycles at the same concentration and irradiation conditions is also given.

To study these photoinduced processes, the UV–vis absorption spectra of the initial ring-open isomer of the synthesized DTE-based ligands and their palladium(II) complexes were first recorded in cyclohexane (Figures 2a,b and S5). In addition, TD-DFT calculations at the CAM-B3LYP-D3/6,31G(d,p) level were performed to further investigate the electronic excitations of these compounds (Tables S2–S5), for which ground state geometries were first computed. For ligands and complexes bearing open-state DTE units, we considered only their photocyclizable antiparallel conformation in our calculations. All open-state phosphine ligands were characterized by a strong absorption in the UV region with maxima around λ_abs_ ≈ 290 nm, which was reproduced in computations and could be attributed to π–π* transitions (HOMO – LUMO+1 or HOMO – LUMO) of their core (Tables S3 and S5, Figures S24–S26). In the case of **DTE-COCF**_**3**_, a shallower absorption band ranging up to λ_abs_ ≈ 400 nm was detected due to the lowering of the energy of the LUMO caused by the introduction of a strong EWG (Table S2).^44^ As for the UV–vis absorption of the open-state Pd^II^ complexes, they not only preserved all of the spectral features of their constituting DTE^o^ ligands but also exhibited a new red-shifted band with a maximum at λ_abs_ ≈ 350 nm (Table 1). According to our TD-DFT calculations, this additional absorption band can be mainly assigned to a ligand-to-metal charge transfer (LMCT) transition, as the LUMO of all of the open-state complexes is located on the palladium center (Tables S4 and S5 and Figures S27–S29).

**Table 1:** Photochemical Properties of DTE-Based Ligands and Complexes

	λ_abs_^o^ [nm] (ε [M^–1^ cm^–1^])^a^	λ_abs_^c^ [nm] (ε [M^–1^ cm^–1^])^b^	PSS_o–c_ composition [%]^c^	Φ_o–c_^d^	Φ_c–o_^e^
**DTE-COCF**_**3**_	268 (35 673), 339 (6 431)	598 (12 261)	91:9	0.480	0.012
[PdCl_2_(**DTE-COCF**_**3**_)_2_]	271 (34 149), 355 (19 065)	604 (18 870)	45:45:10	0.148/0.048	0.013/0.014
**DTE-C**_**6**_**F**_**5**_	272 (28 303)	519 (11 379)	81:19	0.530	0.015
[PdCl_2_(**DTE-C**_**6**_**F**_**5**_)_2_]	268 (40 394), 357 (14 367)	532 (19 391)	25:45:30	0.047/0.019	0.015/0.018
**DTE-Ph**	273 (28 117)	518 (13 673)	39:61	0.485	0.011
[PdCl_2_(**DTE-Ph**)_2_]	270 (42 700), 359 (15 400)	538 (16 310)	27:44:29	0.060/0.023	0.015/0.014

a Wavelength and molar absorptivity coefficient of the absorption band maxima of the open isomer (for complexes, **oo** state) in cyclohexane.

b Wavelength and molar absorptivity coefficient of the maximum of the visible absorption band of the closed isomer (for complexes, **cc** state) in cyclohexane.

c Composition of the PSS reached for the photocyclization process in toluene-*d*_8_ upon irradiation at λ_exc_ = 365 nm (**DTE-COCF**_**3**_ and all of the complexes) or 312 nm (**DTE-C**_**6**_**F**_**5**_ and **DTE-Ph**). DTE^c^/DTE^o^ and DTE^cc^/DTE^oc^/DTE^oo^ concentration ratios are given for free ligands and complexes, respectively.

d Photocyclization quantum yields measured in cyclohexane at λ_exc_ = 355 nm (**DTE-COCF**_**3**_ and all of the complexes) or 312 nm (**DTE-C**_**6**_**F**_**5**_ and **DTE-Ph**). For the complexes, separate Φ_o–c_ values are given for the **oo** → **oc** and **oc** → **cc** ring-closing processes.

e Photocycloreversion quantum yields measured in cyclohexane at λ_exc_ = 532 nm. For the complexes, separate Φ_c–o_ values are given for the **cc** → **oc** and **oc** → **oo** ring-opening processes.

Based on the electronic absorption properties of the open-state ligands and complexes, their ring-closing photoisomerization was assayed upon irradiation with UV light. For open-state ligands, a broad, red-shifted absorption band emerged in the visible part of the spectrum upon UV illumination of their cyclohexane solutions, which changed from colorless to deep blue (λ_abs_ = 598 nm for **DTE**^**c**^**-COCF**_**3**_) or deep pink (λ_abs_ ≈ 518 nm for **DTE**^**c**^**-C**_**6**_**F**_**5**_ and **DTE**^**c**^**-Ph**) (Figure 2a, Table 1 and Figure S5). This behavior is characteristic of closed-state DTEs,^45^ as confirmed by TD-DFT calculations (Table S5, Figures S24–S26) and additional NMR spectroscopic measurements. In particular, we observed the appearance of a new set of signals for the closed isomer in the ^1^H, ^31^P (proton-coupled and decoupled), and, when applicable, ^19^F NMR spectra of the UV-irradiated ligands in toluene-*d*_8_, which were upfield- (^1^H NMR) or downfield-shifted (^31^P, ^31^P{^1^H}, and ^19^F NMR) relative to the NMR resonances of the open state (Figures S7–S14). Analysis of these NMR data also revealed that the photocyclization reaction of DTE-based ligands was not quantitative. Instead, a photostationary state (PSS) composed of an equilibrium mixture of the ligands’ **o** and **c** states was obtained in all of the cases since both isomers absorb at the UV excitation wavelength used and, therefore, should simultaneously undergo ring-closing and ring-opening reactions. Under our illumination conditions in toluene-*d*_8_, better photocyclization conversions were observed with **DTE**^**o**^**-COCF**_**3**_ (91% at λ_exc_ = 365 nm) and **DTE**^**o**^**-C**_**6**_**F**_**5**_ (81% at λ_exc_ = 312 nm) compared to **DTE**^**o**^**-Ph** (39% at λ_exc_ = 312 nm) (Table 1). This can be ascribed to the lower ring-closing quantum yield and high photodegradation tendency of **DTE-Ph**, which limited the UV exposure time for photocyclization and led to a lower percentage of the closed isomer.

Similar to free DTE ligands, irradiation of the Pd^II^ complexes in cyclohexane with UV light also caused the appearance of an absorption band in the visible part of the spectrum and a concomitant color change (Figure 2b, Table 1 and Figure S5). In combination with NMR spectroscopic measurements in toluene-*d*_8_ and TD-DFT calculations, it was demonstrated that DTE photocyclization takes place in the complexes, in contrast to some previously reported DTE-based phosphine-metal compounds.^39,46^ However, palladium(II) complexation did have a relevant effect on the UV-induced ring-closing process of the phosphine ligands. First, we could use less energetic UV radiation to promote the photocyclization of [PdCl_2_(**DTE**^**o**^**-C**_**6**_**F**_**5**_)_2_] and [PdCl_2_(**DTE**^**o**^**-Ph**)_2_] (λ_exc_ = 365 nm) compared to **DTE**^**o**^**-C**_**6**_**F**_**5**_ and **DTE**^**o**^**-Ph** (λ_exc_ = 312 nm) because of the new red-shifted band measured for the two complexes at λ_abs_ ∼ 350 nm. Second, a bathochromic shift of the absorption band of the closed isomer in the visible range was registered relative to the free ligands (λ_abs_ = 604, 532, and 538 nm for [PdCl_2_(**DTE**^**c**^**-COCF**_**3**_)_2_], [PdCl_2_(**DTE**^**c**^**-C**_**6**_**F**_**5**_)_2_], and [PdCl_2_(**DTE**^**c**^**-Ph**)_2_]) (Figure 2a,b, Table 1 and Figures S27–S29). According to TD-DFT calculations, this effect is due to the decrease in the HOMO–LUMO gap of DTE-based phosphines upon Pd^II^ complexation, which should be especially important for **DTE-C**_**6**_**F**_**5**_ and **DTE-Ph**, as experimentally observed (Table S2). On the other hand, the spectral features of the closed DTE absorption band were not influenced by the state of the nearby DTE ligand in the same complex (open or closed), i.e., it did not evolve when photoconverting from the **oc** species with one open and one closed DTE unit to the fully closed **cc** complex, which was substantiated by TD-DFT calculations (Figures S27–S29). As a result, these two different photocyclization products could only be discriminated by NMR spectroscopic experiments, which proved that they were sequentially formed upon UV irradiation and allowed us to determine the composition of the equilibrium PSS generated in toluene-*d*_8_ (Table 1 and Figures S15–S22). Interestingly, only one set of signals was detected for the **cc** state of the complexes in the NMR spectra, though it must comprise a mixture of different stereoisomers because of the chiral centers created in their DTE units upon conrotatory photocyclization.^25^ Therefore, this suggests that the stereochemistry of these ring-closed units has a minor effect on the chemical environment of the phosphorus atoms of the phosphines, and consequently, it should not affect the catalytic activity of the complexes. Further analysis of the NMR spectra of the complexes allowed us concluding that metal coordination detrimentally affected the photocyclization conversion of **DTE**^**o**^**-COCF**_**3**_ and **DTE**^**o**^**-C**_**6**_**F**_**5**_, as only 66 and 48% of the DTE units in [PdCl_2_(**DTE**^**o**^**-COCF**_**3**_)_2_] and [PdCl_2_(**DTE**^**o**^**-C**_**6**_**F**_**5**_)_2_] could be ring-cycled. This result is consistent with the lower photocyclization quantum yields (Φ_o–c_) measured for both the **oo** and **oc** states of the complexes relative to the free ligands (Table 1), which can be attributed to two main effects: (i) the competition between photoisomerization and ligand-to-metal charge transfer, which, as previously mentioned, gives rise to additional absorption bands in the UV region of the complexes at which they are excited to promote photocyclization, and (ii) the further reduction of Φ_o–c_ for the second ring-closing step in the complexes, a behavior already reported for other DTE dimers,^47^ which can be ascribed to intramolecular energy transfer between the open and closed DTE units in the **oc** state upon photoexcitation, i.e., the photocyclization of one of these units severely hinders the ring-closing reaction for the second DTE group.

Once photocyclized with UV light, the closed states of the DTE-based ligands and their palladium(II) complexes were found to be thermally stable in solution, and no spontaneous back-isomerization was detected in the dark at room temperature. Accordingly, visible irradiation (λ_exc_ = 520 nm) was applied to promote photoinduced ring-opening of these compounds and to demonstrate the reversibility of their photoswitching behavior in solution. For all of them, complete disappearance of the visible absorption band associated with the closed isomer was registered, which demonstrates quantitative photochemical cycloreversion, regardless of metal complexation (Figure 2a,b and Figures S5, S15, S18, S19, S21, and S22, see SI Materials and Methods for additional details). This is due to the selective photoexcitation of closed-state DTEs with visible light that counterbalances their commonly low ring-opening quantum yields,^25^ as we also measured herein for the ligands and the **cc** and **oc** states of the complexes (Φ_c–o_ < 0.02, Table 1). In spite of this, some residual visible absorption was registered for some of these compounds upon light-induced photocyclization reversion (**DTE-C**_**6**_**F**_**5**_ in Figure 2a; **DTE-Ph** and [PdCl_2_(**DTE-Ph**)_2_] in Figure S5), which could not be attributed to unreacted closed-state species. Instead, it arose from DTE photodegradation, which is normally associated with the UV irradiation of the closed isomer during photocyclization and leads to a characteristic product that absorbs at λ_abs_ ≈ 500 nm.^48^ This effect was further proven by an examination of the fatigue resistance of ligands and complexes upon repetitive photoinduced ring-closing and ring-opening cycles in cyclohexane (Figure 2a,b and Figures S5 and S6). Although some deterioration of their photoswitching behavior was eventually observed for all of these compounds, the highest photodegradation effects were registered for **DTE-Ph**, **DTE-C**_**6**_**F**_**5**_, and [PdCl_2_(**DTE-Ph**)_2_]. We ascribe these results to two main factors that increase the photostability of the remaining ligands and complexes: (i) the presence of the strong trifluoroacetyl EWG at the external position of the thiophene ring, which is known to slow down DTE photodegradation,^48^ and (ii) the use of less energetic UV light to photoisomerize the palladium(II) complexes relative to the free ligands. Consequently, target compounds [PdCl_2_(**DTE-COCF**_**3**_)_2_] and [PdCl_2_(**DTE-C**_**6**_**F**_**5**_)_2_] showed the highest resistance to photodegradation.

### Photomodulation of the Properties of the Phosphine Ligand

As anticipated by molecular design and demonstrated by ^31^P (proton-coupled and -decoupled) NMR spectroscopic measurements discussed above, the phosphanyl and electron-modulating groups of the synthesized DTE-based ligands are electronically decoupled in their open state. As a result, these ligands should present similar binding properties to metals, a behavior that we aimed to modulate upon photoisomerization. In fact, UV-induced ligand ring-closing caused a measurable downfield shift of the ^31^P and ^31^P{^1^H} NMR signal of these compounds (Δδ = 9.4, 8.2, and 8.3 ppm for **DTE-COCF**_**3**_, **DTE-C**_**6**_**F**_**5**_, and **DTE-Ph**, respectively), thus suggesting a change in the electronic properties of their phosphine groups that might be dependent on the nature of the external substituent present in the other thiophene of the DTE core. In particular, introduction of electron-withdrawing substituents such as trifluoroacetyl and pentafluorophenyl to phosphines is expected to (i) increase the *s* character of the lone pair of electrons at the phosphorus atom involved in σ bonding to metals while (ii) stabilizing and enlarging the size of the phosphine’s σ* antibonding orbital participating in metal π backbonding.

**Scheme 4:**
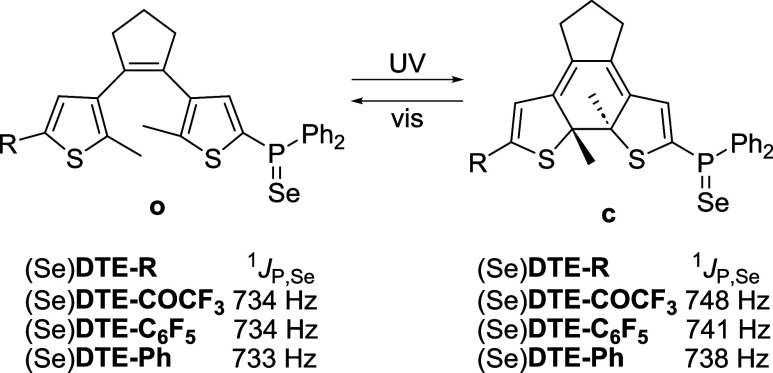
Variation of ^1^*J*_P,Se_ in the Open and Closed Isomers of the Selenides of **DTE-COCF**_**3**_, **DTE-C**_**6**_**F**_**5**_, and **DTE-Ph**

A well-established method to assess such an effect for phosphines is to measure the spin–spin coupling constant between ^31^P and ^77^Se (^1^*J*_P,Se_) for the corresponding selenide derivatives (Scheme 4),^39,41^ which were prepared by heating the ligands and gray selenium in CDCl_3_. In these compounds, ^1^*J*_P,Se_ values depend on the *s* character of the P = Se bond, which is related to the electronic character and size of the substituents on the phosphorus atom.^49−51^ Consequently, they provide an estimate of the σ-donating ability of phosphines, which is lower for higher values of ^1^*J*_P,Se_. For the open-state selenides of **DTE-COCF**_**3**_, **DTE-C**_**6**_**F**_**5**_, and **DTE-Ph**, measured ^1^*J*_P,Se_ values are almost identical and match the reported coupling constant for the selenide of (2-methyl-5-thienyl)diphenylphosphine (^1^*J*_P,Se_ = 733 Hz)^40^ (Scheme 4). Again, this result corroborates that the two thiophenes in these DTE structures are electronically isolated, and for that reason, their phosphanyl groups have similar electronic properties. In contrast, as ring-closing extends the conjugation throughout the DTE moiety, the electron density on the phosphorus atom should decrease and can be affected by the external substituent in the other thiophene ring with which it communicates. This behavior was indeed proven by NMR measurements of the closed selenide isomers: higher ^1^*J*_P,Se_ values were detected relative to the open isomers with increments (Δ^c–o^(^1^*J*_P,Se_)) that scaled up with the electron-withdrawing nature of the electron-modulating substituents (Scheme 4, Table 2), i.e., the studied phosphines became electron-poorer upon photocyclization and introduction of a stronger EWG in the opposite thiophene ring. Thus, the highest value of Δ^c–o^(^1^*J*_P,Se_) was registered for the selenide of **DTE-COCF**_**3**_ (Δ^c–o^(^1^*J*_P,Se_) = 14 Hz), which is the highest reported for DTE-based phosphine ligands^39,41^ and mimics the electronic effects caused by substituting one phenyl ring in triphenylphosphine for a *tert*-butyl group.

**Table 2:** Experimental and Computed Parameters to Estimate the Photomodulation of the Properties of the Phosphine Ligands **DTE-COCF**_**3**_, **DTE-C**_**6**_**F**_**5**_, and **DTE-Ph**

	free ligands			Pd^II^ complexes^a^
	Δ^c–o^(^1^*J*_P,Se_) [Hz]^b^	Δ^c–o^(*q*_P_^Mulliken^)^c^^,^^d^	Δ^c–o^(%*s*_P_)^c^^,^^e^	Δ^c–o^(BE_P–Pd_) [kcal·mol^–1^]^c^^,^^f^
**DTE-COCF**_**3**_	14	0.014	0.79	–1.98
**DTE-C**_**6**_**F**_**5**_	7	0.004	0.42	–0.92
**DTE-Ph**	5	<0.001	0.34	–0.64

a *trans*-[PdCl_2_(**DTE-COCF**_**3**_)_2_], *trans*-[PdCl_2_(**DTE-C**_**6**_**F**_**5**_)_2_], and *trans-*[PdCl_2_(**DTE-Ph**)_2_].

b Difference in ^1^*J*_P,Se_ for the corresponding selenides measured in CDCl_3_.

c Computed at the B3LYP-D3 level in THF (see the Experimental Section for further details).

d Difference in Mulliken charges in electronic units on the phosphorus atom.

e Difference in percentage of *s* character of the phosphorus lone pair of electrons.

f Difference in phosphine-Pd^II^ bond energy (per one bond) between the **oo** and **cc** isomers.

To further investigate the photomodulation of the electronic features for the prepared DTE-based phosphines, we analyzed a set of properties derived from DFT calculations of their ground state structures. As a first step, we considered the variation of the Mulliken charges on the phosphorus atom (Δ^c–o^(*q*_P_^Mulliken^)) and the percentage of *s* character of the lone pair of electrons at phosphorus (Δ^c–o^(%*s*_P_)) for open antiparallel and closed conformations of **DTE-COCF**_**3**_, **DTE-C**_**6**_**F**_**5**_, and **DTE-Ph** (Table 2). According to the Mulliken charges, photocyclization decreases electron density on phosphorus, while through NBO analysis,^52^ we can anticipate an increase in *s* orbital participation in the phosphorus lone pair of electrons. More importantly, the variation of these parameters was found to increase with the electron-withdrawing power of the introduced electron-modulating group, thus again validating that **DTE-COCF**_**3**_ and, to a lesser extent, **DTE-C**_**6**_**F**_**5**_ experience the largest change in phosphine’s electronic properties upon photoisomerization.

As a second step, the effect of the light-induced modulation of the DTE-based phosphine ligands on the bond energy in their Pd^II^ complexes was investigated computationally. For this, we analyzed the difference in phosphine-Pd^II^ binding energy (Δ^c–o^(BE_P–Pd_)) between the **oo** and **cc** isomers of their 2:1 *trans*-phosphine–palladium(II) complexes (Table 2). As expected, due to the loss of the phosphine’s σ-donating ability, a varying decrease in BE_P–Pd_ for the complexes bearing ring-closed DTE ligands is observed, depending on the nature of the introduced electron-modulating group. Thus, weakening of the phosphine–palladium(II) binding upon photocyclization was observed for the ligands bearing the electron-withdrawing pentafluorophenyl and especially trifluoroacetyl substituents, as we initially devised.

### Catalytic Studies

Among the vast range of Pd-catalyzed reactions, Stille coupling was chosen to evaluate the activity of the open and closed states of the prepared metal complexes. For this reaction, previous mechanistic studies have established that bulky phosphines accelerate the rate of oxidative addition while electron-poor phosphines are advantageous for the transmetalation step.^36,53−55^ This makes Stille coupling a suitable benchmark process to validate our model toward photoswitchable catalysis, as we have proven above that the electron density on the DTE-based phosphines developed herein can be modulated upon photoisomerization. With this aim, the palladium(II) complexes were tested as precatalysts for the Stille reaction between iodobenzene and tributylvinyltin in THF-*d*_8_ at two different temperatures (room temperature and 50 °C) (Table 3). In all cases, catalytic experiments were conducted in the dark and separately for the pure **oo** complexes and for **cc**-enriched mixtures of isomers, which were observed to be thermally stable even at the highest temperature considered (50 °C; Figures S33–S36). Because of the moderate efficiency of **oo**-to-**cc** photocyclization in the complexes (see Table 1), we maximized the relative amount of **cc** species in such mixtures by first ring-closing the corresponding free ligand and then conducting metal complexation (see the Supporting Information for further details). In this way, more **cc**-enriched precatalyst mixtures could be prepared, which yet contained a moderate relative concentration of **cc** isomers: 52, 38, and 49% for [PdCl_2_(**DTE-COCF**_**3**_)_2_], [PdCl_2_(**DTE-C**_**6**_**F**_**5**_)_2_], and [PdCl_2_(**DTE-Ph**)_2_], respectively (Figures S30–S32). To compare the catalytic efficiency of these mixtures with those of their **oo** samples, we monitored the kinetics of the Stille reaction for 6 h and measured the difference in product formation after this time (entries 1–6 and 8–13 in Table 3; Figure S37). In addition, equivalent measurements were performed using [PdCl_2_(PPh_3_)_2_] (entries 7 and 14 in Table 3) as a non-light-responsive reference precatalyst, which, in most of the cases, turned out to be less efficient than our DTE-based complexes.

**Table 3:** Comparison of the Investigated Complexes as Precatalysts in the Stille Coupling Reaction

		25 °C		50 °C	
precatalyst [Pd]	state^a^	entry	yield (%)^b^	entry	yield (%)^b^
[PdCl_2_(**DTE-COCF**_**3**_)_2_]	**oo**	1	4.5	8	36.4
	**cc**	2	8.8	9	48.9
[PdCl_2_(**DTE-C**_**6**_**F**_**5**_)_2_]	**oo**	3	21.2	10	42.5
	**cc**	4	29.5	11	59.6
[PdCl_2_(**DTE-Ph**)_2_]	**oo**	5	13.4	12	53.8
	**cc**	6	14.2	13	43.4
[PdCl_2_(PPh_3_)_2_]		7	9.8	14	28.0

a **cc** state here is a closed-state enriched precatalyst complex as specified in the text.

b The average yields of two repetitions were determined by ^1^H NMR spectroscopy using 1,3,5,-trimethoxybenzene as a standard.

As shown in Table 3, two different behaviors were observed in the catalytic tests. For complexes bearing DTE units with external electron-withdrawing substituents, a large increase in yields for the Stille coupling at 6 h was registered for the **cc**-enriched mixtures compared to the **oo** complexes: about 2- (from entry 1 to entry 2) and 1.35-fold increase in yield (from entry 8 to entry 9) for [PdCl_2_(**DTE-COCF**_**3**_)_2_] at room temperature and 50 °C, respectively, and about 1.4-fold increase in yield for [PdCl_2_(**DTE-C**_**6**_**F**_**5**_)_2_] (from entries 3 to 4 and 10 to 11) both at room temperature and 50 °C. By contrast, DTE photocyclization did not enhance the Stille coupling efficiency for [PdCl_2_(**DTE-Ph**)_2_], which lacks the electron-withdrawing substituent on the DTE core. In this case, similar reaction conversions were measured for the **oo** complex and **cc**-enriched mixture at room temperature (entries 5 and 6), while we observed a decrease in reactivity upon ring-closing at 50 °C (entries 12 and 13). Importantly, these results are in agreement with the prediction made to accomplish photoswitchable catalysis by installing external EWGs in DTE-based phosphines. Upon photocyclization, the electron-withdrawing and phosphanyl substituents become selectively conjugated in these compounds, thus varying the electronic properties of the phosphine ligand and affecting the catalytic behavior of its metal complexes. Unfortunately, the catalytic modulation accomplished in this way is limited by the nonquantitative nature of DTE photocyclization, which prevented us from conducting experiments with pure **cc** precatalysts.

**Figure 3:**
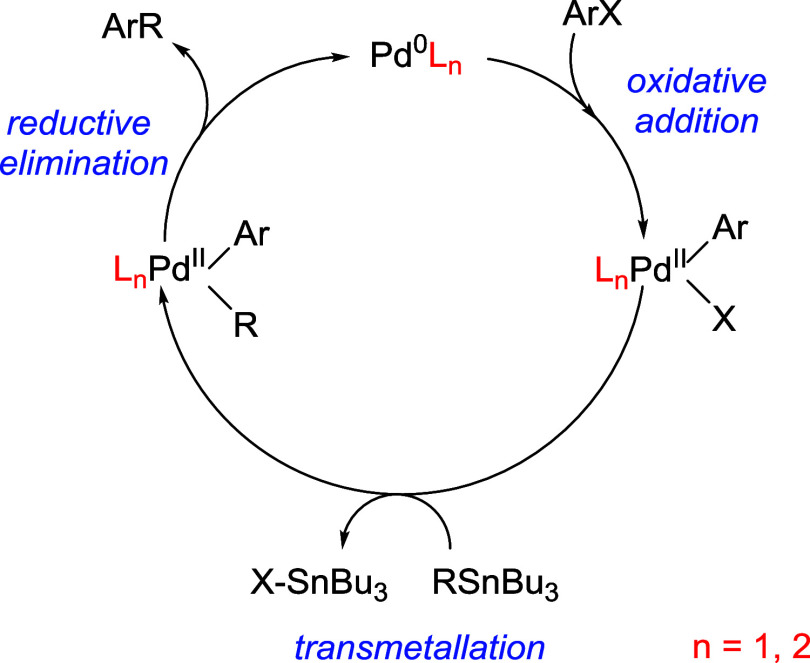
General catalytic cycle for the palladium-catalyzed Stille coupling reaction.

To rationalize the modulation of Stille coupling reactivity determined for the open and closed states of [PdCl_2_(**DTE-COCF**_**3**_)_2_] and [PdCl_2_(**DTE-C**_**6**_**F**_**5**_)_2_], we conducted additional DFT calculations. For this, we considered the Stille reaction mechanism,^36,56^ which requires previous reduction of the palladium(II) precatalyst used to palladium(0) before the catalytic cycle begins. Similar to other palladium-catalyzed couplings, the catalytic cycle consists of three major steps: oxidative addition, transmetalation, and reductive elimination,^57^ among which the first two are typically the rate-determining steps when aryl halides are used as substrates (Figure 3).^35,58^ Over the years, various pathways have been proposed for each of these steps. On the one hand, the oxidative addition of the organic electrophile to Pd^0^ can occur through a monophosphine pathway, an associative displacement pathway, and a bisphosphine pathway.^59,60^ Principally, the presence of bulky ligands should favor oxidative addition via a monoligated transition state owing to steric repulsion.^61^ Nevertheless, it was recently established that intramolecular dispersion forces can serve to stabilize a bisligated transition state for bulky ligands, such as P*t*Bu_3_,^62^ thus demonstrating the importance of incorporating dispersion effects into the analysis. Accordingly, we explored both types of pathways in our computations. As for the transmetalation step, it can also proceed through three different mechanisms: cyclic, open, and ionic.^58^ In our calculations, we only considered the first of these cases, as halides are considered to be good bridging ligands that facilitate the formation of cyclic transition states; in contrast, open and ionic mechanisms are preferred in the case of poorly coordinating anionic ligands and highly polar solvents. Finally, the reductive elimination step can also proceed through bisligated and monoligated transition states.^58^ For simplicity, herein, we only explored the second of these options and did not compute the bisligated pathway.

**Figure 4:**
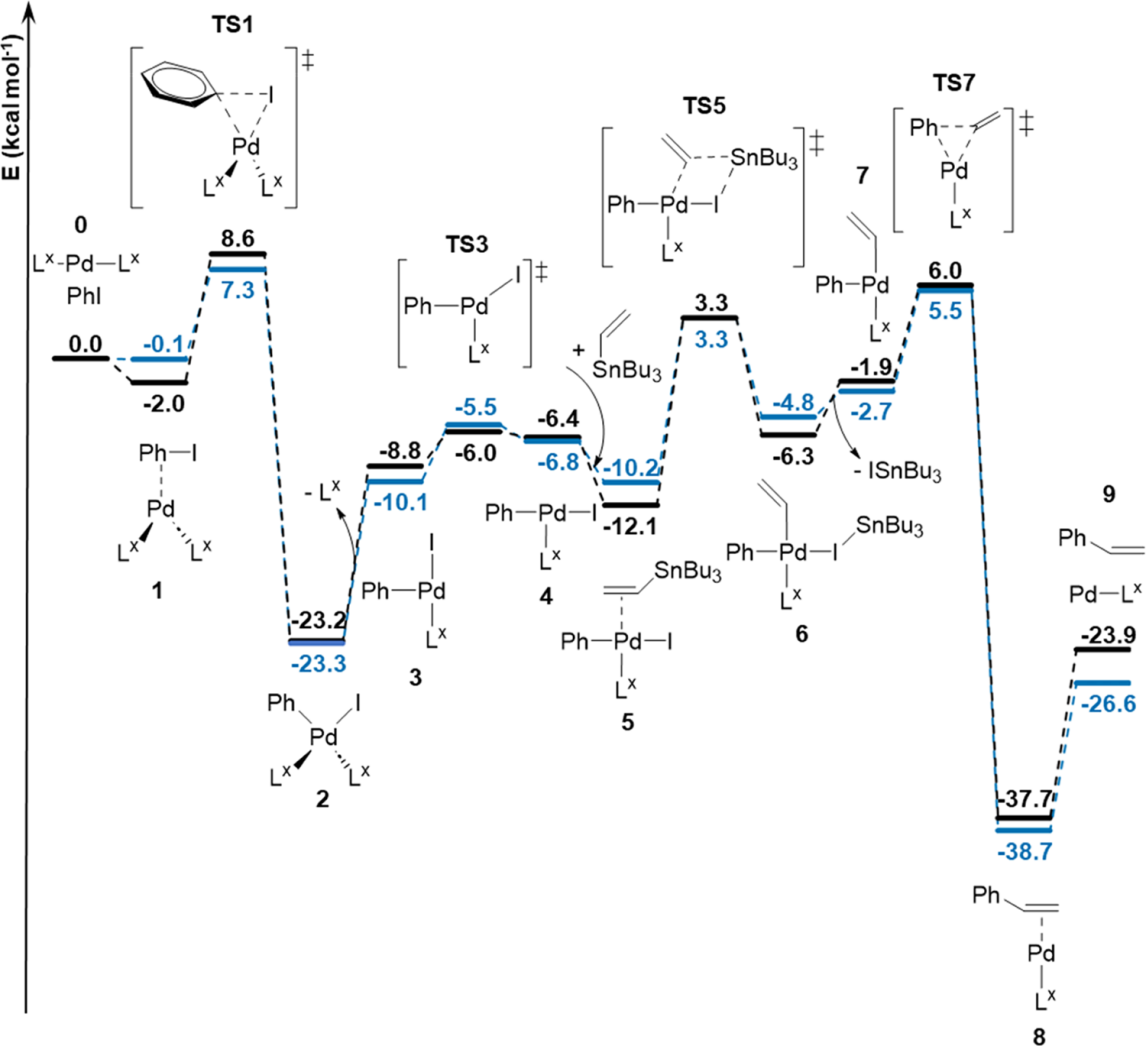
Calculated Gibbs energy profile for the Stille coupling reaction in solution (THF) using [PdCl_2_(**DTE**^**o**^**-COCF**_**3**_)_2_] or [PdCl_2_(**DTE**^**c**^**-COCF**_**3**_)_2_] as a precatalyst, where L^x^ is **DTE**^**o**^**-COCF**_**3**_ (black line) or **DTE**^**c**^**-COCF**_**3**_ (blue line).

Based on these mechanistic assumptions, we computed the catalytic cycle of the Stille coupling reaction investigated experimentally. Calculations were carried out for complexes formed with ligand **DTE-COCF**_**3**_, as it should impart the strongest electronic effects upon photoisomerization. Three possible states of the **DTE-COCF**_**3**_-based Pd^0^ catalyst were considered in these calculations: [Pd(**DTE**^**o**^**-COCF**_**3**_)_2_], [Pd(**DTE**^**o**^**-COCF**_**3**_)(**DTE**^**c**^**-COCF**_**3**_)], and [Pd(**DTE**^**c**^**-COCF**_**3**_)_2_] (Figure 4, Figures S38–S40 and Table S6). Reaction intermediates and transition states were computed at the B3LYP-D3 level of theory (see the Supporting Information for additional details). The Gibbs energies of the species at 298 K are presented relative to the zero point consisting of the corresponding bisligated palladium(0) complex (**0**, Figure 4), phenyl iodide, and tributylvinyltin. For the oxidative addition step, the monophosphine pathway was found to be disfavored, as phosphine ligand dissociation was computed to have a high energy requirement (>20 kcal/mol, Figures S39–S40). It is worth noting, however, that the energy barrier for the dissociation of the closed-state ligand from the **cc** (23.1 kcal/mol) and **oc** (26.8 kcal/mol) species is lower than that for the dissociation of the open ligand from the **oo** (25.8 kcal/mol) and **oc** (27.3 kcal/mol) states of the catalyst, thus corroborating our predictions on the effect of the **DTE-COCF**_**3**_ isomerization state on the stability of the phosphine–palladium bond. The bisphosphine oxidative addition pathway was found to be preferred as it proceeds through the less energetic three-center transition state **TS1**, which is accessed through the previous intermediate **1** (Figure 4). Among the transition states **TS1** computed for the three different catalytic systems, the lowest energy values are associated with the **cc** and **oc** states, at 7.4 and 7.8 kcal/mol, respectively, while the **oo** isomer requires 10.6 kcal/mol. As the transition state for the oxidative addition of the bisligated complex leads to the formation of the fully *cis*-coordinated Pd^II^ species **2**, ligand dissociation is a prerequisite for subsequent steps, which was found to be slightly less energy demanding for the **cc** system (13.2 kcal/mol for the **cc** species vs 14.4 kcal/mol for the **oo** species). Then, the formed intermediate **3** isomerizes through the transition state **TS3** (energy barrier ≈2.8–4.7 kcal/mol), and upon tin coordination, transmetalation takes place with the highest energy barrier among all of the steps. In particular, the energy barrier for the cyclic transition state **TS5** involving the closed-state Pd^0^ species is 13.5 kcal/mol, which is lower than that for the open-state catalytic system (15.4 kcal/mol). Lastly, reductive elimination of intermediate **6** was observed to take place through transition state **TS7**, with an energy barrier difference of only 0.3 kcal/mol between the closed- and open-state isomers. In conclusion, our computational analysis revealed that the barriers for the most energy-demanding steps of the investigated Stille coupling reaction, i.e., oxidative addition, ligand dissociation, and transmetalation, are lower for the catalytic palladium species bearing closed-state **DTE-COCF**_**3**_ ligands. This is in agreement with our experimental results, which showed higher catalytic activity for the **cc**-enriched state of the [PdCl_2_(**DTE-COCF**_**3**_)_2_] (and [PdCl_2_(**DTE-C**_**6**_**F**_**5**_)_2_]) precatalyst. Therefore, these results prove that reducing the σ-donation ability of the phosphines of these complexes by making them conjugated with electron-withdrawing groups upon DTE photocyclization favors their catalytic activity in the Stille coupling reaction. Nevertheless, the differences in energy barriers computed for the open- and closed-state isomers of the system along the catalytic cycle are moderate (≈1–3 kcal/mol), which in combination with incomplete DTE photocyclization should account for the limited modulation of reactivity accomplished in our experiments.

## Conclusions

Dithienylethenes were used to prepare the photoisomerizable phosphine ligands **DTE-COCF**_**3**_ and **DTE-C**_**6**_**F**_**5**_, where the electronic communication between the phosphorus atom and electron-withdrawing groups is switched on and off by reversible open- to closed-state conversion. As a result, the σ-donating ability of these phosphines can be efficiently modulated upon light irradiation, in agreement with DFT calculations, improving the behavior observed for the analogous **DTE-Ph** ligand bearing an EWG-free dithienylethene moiety. Interestingly, when coordinated to palladium(II), the resulting bisphosphine complexes preserve the ligand’s capacity to undergo ring cyclization under illumination, albeit resulting in incomplete phototransformation into their dual closed-state isomer. Finally, when testing the synthesized complexes as precatalysts in the Stille coupling reaction between phenyl iodide and tributylvinyltin, a clear modulation in reaction rate was observed upon photoisomerization of the compounds bearing **DTE-COCF**_**3**_ and **DTE-C**_**6**_**F**_**5**_ ligands. In particular, the catalytic activity of the complexes increased in the closed state of these ligands, which could be rationalized by DFT calculations. Therefore, these results validate our molecular design toward photoswitchable catalysis, which can be extended to other metals and reactions in the future.

## Experimental Section

### Synthesis

A detailed description of the synthesis of ligands and complexes is given in the Supporting Information. No uncommon hazards are noted.

### Single-Crystal X-ray Diffraction Analysis

The data were collected on a Gemini diffractometer (Rigaku Oxford Diffraction) using Mo Kα radiation and ω-scan rotation. Data reduction was performed with CrysAlisPro,^63^ including the program SCALE3 ABSPACK for an empirical absorption correction. As a result of the extremely thin crystal, for [PdCl_2_(**DTE**^**o**^**-C**_**6**_**F**_**5**_)_2_], a numerical absorption correction was applied as well using a multifaceted crystal model based on expressions derived by Clark and Reid.^64^ All structures were solved by dual space methods with SHELXT^65^ and the refinement was performed with SHELXL.^65^ Hydrogen atoms were calculated on idealized positions by using the riding model. Structure figures were generated with DIAMOND–4^66^ and Mercury (version 2022.2.0).^67^

### Photochemical Characterization

Photoswitching was monitored by UV–vis and NMR spectroscopy. The photostationary state PSS^oc^ composition was determined through ^31^P or ^19^F NMR spectroscopy from a PSS^oc^ state produced by irradiating a toluene-*d*_8_ solution in an NMR tube with the appropriate wavelength. Spectra of the closed-state isomers shown in Figures S24−S29 were estimated from the PSS^oc^ and open-state UV–vis spectra. Photoisomerization quantum yields were determined by monitoring the variation of the UV–vis absorption spectra of ligands and complexes in cyclohexane upon irradiation with UV (for photocyclization, λ_exc_ = 312 or 355 nm) or visible light (for photocycloreversion, λ_exc_ = 532 nm). In the case of the free ligands bearing one DTE unit, spectral data were fitted to a simple kinetic model previously reported.^68^ For the complexes containing two DTE groups, a more complex kinetic model had to be used to separately determine the photoisomerization quantum yields of their **oo**, **oc**, and **cc** isomers.^69^ To apply this model, we assumed the UV–vis absorption spectrum of each DTE unit in the complexes to be independent of the isomerization state of the other, i.e., the extinction coefficients of open DTE units are the same in the **oo** and **oc** states, while those of closed DTE groups are equal in the **oc** and **cc** states, as suggested by our TD-DFT calculations and observed in previous works on DTE dimers.^70^ In all of the cases, the irradiation intensities used in our photoisomerization quantum yield experiments were determined by monitoring the photocyclization and photocycloreversion processes of 1,2-bis(2-methyl-5-trifluoroacetylthien-3-yl)cyclopentene in toluene as a reference (Φ_oc_ = 0.37 and Φ_co_ = 0.031).^33^

### Computational Details

DFT calculations were carried out using the Gaussian16 program package.^71^ Geometry optimizations were conducted without any constraints using the B3LYP functional^72−74^ with Grimme’s D3 correction to account for dispersion effects.^75^ Optimizations were performed in THF using the solvation model density (SMD) continuum model^76^ with basis set 1 (BS1). BS1 included the 6-31G(d,p) basis set for the main group atoms^77,78^ (H, C, O, F, P, S) and the Stuttgart–Dresden SDD effective core potential (ECP) and its corresponding double-ζ basis set,^79^ with a set of *d* polarization functions^80^ for I and Sn and *f* polarization functions^81^ for Pd. Frequency calculations were performed for all of the optimized geometries to determine the stationary points as either minima or transition states. Energies in THF were refined through single-point calculations of the optimized BS1 geometries with an extended basis set (BS2). BS2 consisted of def2-TZVP for main group atoms and the quadruple-ζ def2-QZVP basis set for Pd, together with the def2 ECP.^82^ Gibbs energies in THF were calculated by adding thermal and entropic correction from BS1 to BS2 energies in THF. An additional correction of 1.9 kcal/mol was applied to all of the Gibbs energies to change the standard state from the gas phase (1 atm) to the condensed phase (1 M) at 298.15 K.^83^ Frontier molecular orbitals and natural bond orbital (NBO)^84^ analysis were calculated at the B3LYP-D3/BS1 level in THF using the SMD continuum model. TD-DFT calculations were carried out using the CAM-B3LYP functional^85^ with Grimme’s D3 correction to account for dispersion effects.^75^ The first 15 electronic transitions were calculated in cyclohexane using the SMD continuum model with BS1 described above.

### General Procedure for Catalytic Studies

In an NMR tube, 0.033 mL of iodobenzene (0.30 mmol, 1.0 equiv), 0.097 mL of tributylvinyltin (0.33 mmol, 1.1 equiv), 5 mol % [Pd] catalyst, and 0.05 g of (0.03 mmol, 0.1 equiv) 1,3,5-trimethoxybenzene were dissolved in 1 mL dry, degassed THF-*d*_8_. The reactions were carried out in the dark and monitored every 2 h by ^1^H, ^31^P{^1^H}, and, when applicable, ^19^F NMR spectroscopy. The reaction yields are an average of two replicates.

## Supporting Information Available

Synthetic procedures, additional experimental details, photochemical and catalytic studies, additional theoretical data, and NMR spectra (PDF)
