# Supplementary material for: Dithienylethene-Based Photoswitchable Phosphines for the Palladium-Catalyzed Stille Coupling Reaction
Source: Inorg Chem. 2024 Apr 16;63(17):7652–64. doi: 10.1021/acs.inorgchem.3c04423 (PMC11061837; doi:10.1021/acs.inorgchem.3c04423)
Supplement: Supplementary file 1 — ic3c04423_si_001.pdf [file ic3c04423_si_001.pdf]

Supporting Information

Dithienylethene-based photoswitchable phosphines for palladium-catalyzed Stille coupling reaction

Anastasiia Sherstiuk,[a,b] Agustí Lledós,[b] Peter Lönnecke,[a] Jordi Hernando,[b]* Rosa María Sebastián,[b,c]* and Evamarie Hey-Hawkins[a]*

[a] Faculty of Chemistry and Mineralogy, Institute of Inorganic Chemistry, Leipzig University, Johannisallee 29, D-04103 Leipzig, Germany

[b] Department of Chemistry, Universitat Autònoma de Barcelona, Cerdanyola del Vallès, Bellaterra, 08193 Barcelona, Spain

[c] Centro de Innovación en Química Avanzada (ORFEO-CINQA), Universitat Autònoma de Barcelona, Cerdanyola del Vallès, Bellaterra, 08193 Barcelona, Spain

jordi.hernando@uab.cat, rosamaria.sebastian@uab.cat, hey@uni-leipzig.de

Contents

1 General procedures S2

1.1 Materials and methods S2

1.2 Synthetic procedures S3

1.3 Crystallographic data S10

2 Experimental studies of the photochemical behavior of ligands and complexes S13

2.1 Solid-state photoisomerization of [$PdCl_2$(**DTE-C_6F_5**)$_2$] S13

2.2 Study of the photoisomerization of ligands and complexes by UV-vis absorption spectroscopy S13

2.3 NMR characterization of the photoisomerization of ligands and complexes S14

3 DFT calculations of the optical properties of ligands and complexes S24

3.1 Frontier molecular orbitals analysis S24

3.2 Vertical transitions S30

4 Catalytic studies S34

4.1 NMR characterization of the **cc**-enriched state of the palladium complexes tested S34

4.2 Thermal stability of [$PdCl_2$(**DTE-$COCF_3$**)$_2$] S35

4.3 Catalytic reaction kinetic profiles S37

4.4 Gibbs energy profiles S38

5 NMR spectra of the reported compounds S42

6 Mass spectra of novel free ligands and complexes S61

7 References S64

8 Cartesian coordinates S65

1 General procedures

1.1 Materials and methods

All reactions were carried out under nitrogen atmosphere in the absence of air and water using standard Schlenk line techniques. All solvents (hexanes, CH_2Cl_2, Et_2O, ethyl acetate, *n*-pentane, cyclohexane) were dried and degassed prior to use. THF was distilled over sodium/benzophenone and stored over 4Å activated molecular sieves. $CDCl_3$ and THF-d_8 were degassed by freeze–pump–thaw cycling. Toluene-d_8 was degassed with nitrogen. All starting materials and reagents were commercially purchased and used without further purification. $[Pd(PPh_3)_4]$, $[PdCl_2(PPh_3)_2]$ and *trans*-$[PdCl_2(PhCN)_2]$ were synthesized according to previously reported procedures.[1–3] Flash column chromatography was done using silica gel (230-400 mesh) using a stream of nitrogen.

NMR spectra were recorded on a BRUKER Avance III HD 400 MHz, BRUKER Ascend 300 MHz and BRUKER Ascend 400 MHz at 25 °C. Tetramethylsilane (TMS) was used as an internal reference in ^{1}H and ^{13}C NMR spectra; all other nuclei were referenced to TMS using the Ξ scale.[4] Chemical shifts are reported in parts per million (ppm). Assignment of ^{1}H and ^{13}C NMR signals was carried out using ^{1}H-^{1}H COSY, ^{1}H-^{13}C HSQC and ^{1}H-^{13}C HMBC NMR experiments. IR spectra were recorded on FT-IR spectrometers Thermo Scientific Nicolet iS5, BRUKER Tensor 27 Golden Gate and BRUKER Alpha II. Electrospray ionization mass spectrometry was carried out with BRUKER Impact II, BRUKER Esquire 3,000+ and a micrOTOF-Q II BRUKER spectrometer in positive ion mode. UV-vis absorption spectra were recorded on an Agilent HP 8453 spectrophotometer using HPLC quality solvents and 1 cm quartz cuvettes.

Photoisomerization studies were carried out using different irradiation sources: 365 nm and 520 nm LEDs (Chanzon), a VL-6.M UV lamp (λ_{exc} = 312 nm, 6 W), and a Nd:YAG pulsed laser (Brilliant, Quantel, λ_{exc} = 355 or 532 nm). The irradiation times applied to conduct the photocyclization and cycloreversion reactions depended on the compound concentration, irradiation source and physical parameters of the experimental set up (sample geometry and distance to the irradiation source). For the photoisomerization experiments monitored by NMR spectroscopy, typical irradiation times required to reach the PSSs were: (a) using a 365 nm LED: 2 h for **DTE-COCF$_3$** and 4 h for $[PdCl_2($**DTE-COCF$_3$**$)_2]$, $[PdCl_2($**DTE-C$_6$F$_5$**$)_2]$ and $[PdCl_2($**DTE-Ph**$)_2]$; (b) using a 312 nm UV lamp: 6 h for **DTE-C$_6$F$_5$** and **DTE-Ph**; (c) using a 532 nm LED: 30 min for all the compounds. As for photoisomerization experiments monitored by UV-vis absorption in cuvette at more diluted conditions: (a) using a 365 nm LED: 90 s for **DTE-COCF$_3$** and $[PdCl_2($**DTE-COCF$_3$**$)_2]$, and 150 s for $PdCl_2($**DTE-C$_6$F$_5$**$)_2]$ and $[PdCl_2($**DTE-Ph**$)_2]$; (b) using a 312 nm UV lamp: 110 s for **DTE-C$_6$F$_5$** and 180 s for **DTE-Ph**; and (c) using a 520 nm LED: 160 s for **DTE-COCF$_3$**, 120 s for **DTE-C$_6$F$_5$** and **DTE-Ph**, and 180 s for $[PdCl_2($**DTE-COCF$_3$**$)_2]$, $[PdCl_2($**DTE-Ph**$)_2]$ and $[PdCl_2($**DTE-C$_6$F$_5$**$)_2]$.

To determine photoisomerization quantum yields, we monitored the variation of the UV-vis absorption spectra of ligands and complexes in cyclohexane upon irradiation with UV (for photocyclization, λ_{exc} = 312 or 355 nm) or visible light (for photocycloreversion, λ_{exc} = 532 nm). Typically, 10-15 spectra were taken for each photoisomerization process at different irradiation times. For each of these spectra, two main data points were used in the subsequent mathematical treatment: (a) the absorbance at the excitation wavelength (A_{exc}); and (b) the absorbance at the spectral maximum of the ring-closed isomer of each compound (A_{det}). In the case of the free ligands with one DTE unit, the variation of A_{det} with time was fitted to a simple kinetic model where the concentration of the ring-closed isomer (c_c) depends on the photoexcitation intensity (I_0), the optical path (l), the molar absorptivity of the open- (ε_o^{exc}) and ring-closed states (ε_c^{exc}) at the excitation wavelength, the total absorbance at the excitation wavelength (A_{exc}), the total concentration of ligand that remains constant during irradiation (c_T), and the quantum yields for ring-closing ($\Phi_{o\text{-}c}$) and ring-opening ($\Phi_{c\text{-}o}$) (Equations S1 and S2).

$$A_{det} = \varepsilon_c^{det}\, l\, c_c(t) \qquad \text{(S1)}$$

$$\frac{dc_c(t)}{dt} = I_0 \frac{(1-10^{-A_{exc}})}{A_{exc}} \left(\phi_{o-c}\, \varepsilon_{o}^{exc}\, l\, (c_T - c_c(t)) - \phi_{c-o}\, \varepsilon_{c}^{exc}\, l\, c_c(t)\right) \quad \text{(S2)}$$

In the case of the complexes bearing two DTE units, a more complex kinetic model was used to fit the variation of A_{det} with time, which now depends on the concentrations of both the **oc** (c_{oc}) and **cc** (c_{cc}) isomers - i.e., on I_0, l, the molar absorptivity of the **oo** (ε_{oo}^{exc}), **oc** (ε_{oc}^{exc}) and **oc** (ε_{cc}^{exc}) states at the excitation wavelength, A_{exc}, c_T and the four different quantum yields of the system ($\Phi_{oo\text{-}oc}$, $\Phi_{oc\text{-}cc}$, $\Phi_{oc\text{-}oo}$ and $\Phi_{cc\text{-}oc}$) (Equations S3-S5).

$$A_{det} = \varepsilon_{oc}^{det}\, l\, c_{oc}(t) + \varepsilon_{cc}^{det}\, l\, c_{cc}(t) \quad \text{(S3)}$$

$$\frac{dc_{oc}(t)}{dt} = I_0 \frac{(1-10^{-A_{exc}})}{A_{exc}} \left(\phi_{oo-oc}\, \varepsilon_{oo}^{exc}\, l\, (c_T - c_{oc}(t) - c_{cc}(t)) - \phi_{oc-oo}\, \varepsilon_{oc}^{exc}\, l\, c_{oc}(t) - \phi_{oc-cc}\, \varepsilon_{oc}^{exc}\, l\, c_{oc}(t) + \phi_{cc-oc}\, \varepsilon_{cc}^{exc}\, l\, c_{cc}(t)\right) \quad \text{(S4)}$$

$$\frac{dc_{cc}(t)}{dt} = I_0 \frac{(1-10^{-A_{exc}})}{A_{exc}} \left(\phi_{oc-cc}\, \varepsilon_{oc}^{exc}\, l\, c_{oc}(t) - \phi_{cc-oc}\, \varepsilon_{cc}^{exc}\, l\, c_{cc}(t)\right) \quad \text{(S5)}$$

Of all these parameters: (a) I_0 was determined by monitoring the photocyclization and photocycloreversion processes of 1,2-bis(2-methyl-5-trifluoroacetylthien-3-yl)cyclopentene in toluene as a reference (Φ_{oc} = 0.37 and Φ_{co} = 0.031); (b) l = 1 cm; (c) ε_o^{exc}, ε_c^{exc}, ε_c^{det}, ε_{oo}^{exc}, ε_{oc}^{exc}, ε_{cc}^{exc}, ε_{oc}^{det} and ε_{cc}^{det} were obtained from the absorption spectra of solutions with known concentrations of all isomers and, for the case of ε_{oc}^{exc}, ε_{cc}^{exc}, ε_{oc}^{det} and ε_{cc}^{det}, assuming the UV-vis absorption spectrum of each DTE unit in the complexes to be independent of the isomerization state of the other; (d) A_{exc} was measured experimentally; and (e) $\Phi_{o\text{-}c}$, $\Phi_{c\text{-}o}$, $\Phi_{oo\text{-}oc}$, $\Phi_{oc\text{-}cc}$, $\Phi_{oc\text{-}oo}$ and $\Phi_{cc\text{-}oc}$ were obtained from the fitting of the time variation of A_{det} in each case.

1.2 Synthetic procedures

Synthesis of ligands and complexes

{4-[2-(5-Chloro-2-methylthiophen-3-yl)cyclopent-1-en-1-yl]-5-methylthiophen-2-yl}diphenylphosphine (**DTE2**)

This compound was synthesized following a modified procedure.[5] A stirred yellow solution of 0.330 g (1.0 mmol, 1.0 eq.) **DTE1**[6] in 20 mL THF was cooled to -78 °C (hexanes/$N_{2(l)}$), and then 0.78 mL (1.41 mol·L^{-1}, 1.1 mmol, 1.1 eq.) *t*BuLi in *n*-pentane were added dropwise. The resultant bright yellow mixture was kept stirring for 50 min at -78 °C, followed by the addition of 0.20 mL (1.1 mmol, 1.1 eq.) of chlorodiphenylphosphine in one swift motion. The reaction mixture was left overnight to warm up to room temperature, and then quenched with a degassed brine solution. Under nitrogen atmosphere, the phases were separated, the aqueous phase was extracted with THF (2 x 5 mL), and the combined organic phases were dried over degassed Na_2SO_4. After canula filtration, the product mixture was absorbed on silica gel and purified through flash column chromatography (hexanes/dichloromethane 85:15). After solvent removal *in vacuo*, a white oil was obtained (0.317 g, 67% yield).

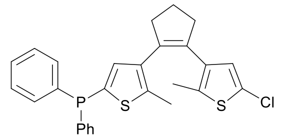

R_f 0.38 (hexanes/dichloromethane 85:15)

^{1}H NMR (400 MHz, $CDCl_3$, δ): 7.34 – 7.33 (m, 10H), 6.92 (d, *J* = 6.3 Hz, 1H), 6.54 (s, 1H), 2.80 – 2.65 (m, 4H), 2.09 – 1.96 (m, 2H), 2.01 (s, 3H), 1.87 (s, 3H) ppm.

^{13}C{^{1}H} NMR (75 MHz, $CDCl_3$, δ): 141.9, 138.0 (d, *J* = 8.6 Hz), 137.8 (d, *J* = 26.4 Hz), 136.8 (d, *J* = 7.2 Hz), 135.2, 135.1, 134.1, 133.0 (d, *J* = 19.5 Hz), 131.7 (d, *J* = 10.4 Hz), 128.8, 128.5, 128.4 (d, *J* = 7.0 Hz), 126.9, 125.0, 38.2, 38.2, 22.9, 14.6, 14.1 ppm.

^{31}P{^{1}H} NMR (162 MHz, $CDCl_3$, δ): -19.7 (s) ppm.

{4-[2-(5-Trifluoroacetyl-2-methylthiophen-3-yl)cyclopent-1-en-1-yl]-5-methylthiophen-2-yl}diphenyl-phosphine (**DTE°-COCF$_3$**)

A stirred solution of 0.385 g (0.8 mmol, 1.0 eq.) **2** in 20 mL THF was cooled to -78 °C (hexane/$N_{2(l)}$), and 0.55 mL (1.6 mol·L^{-1}, 0.9 mmol, 1.1 eq.) *t*BuLi in pentane were added dropwise. The resultant deep red mixture was kept stirring for 45 min at -78 °C, followed by the addition of 0.75 mL (6.4 mmol, 8.0 eq.) of anhydrous ethyl trifluoroacetate in one swift motion. The reaction mixture turned bright yellow and was left to warm up to room temperature over an hour. After quenching with a degassed brine solution, the organic phase was separated, the aqueous phase was extracted with THF (2 x 5 mL), and the combined organic phases were dried over degassed Na_2SO_4. After canula filtration, the product mixture was absorbed on silica gel and purified through flash column chromatography (hexanes/dichloromethane 90:10). After solvent removal *in vacuo*, a yellow oil was obtained (0.298 g, 69% yield). Caution: product is very sensitive to oxygen nucleophiles (water, alcohols); special care should be taken during the work up to avoid formation of the corresponding hydrate.

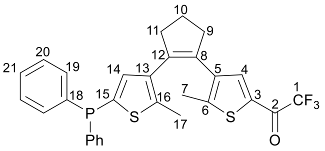

R$_f$ 0.17 (hexanes/dichloromethane 90:10)

^{1}H NMR (400 MHz, $CDCl_3$, δ): 7.37 – 7.30 (m, 10H, H18-21), 7.04 (d, $^2J_{H,P}$ = 6.6 Hz, 1H, H14), 6.85 (s, 1H, H4), 2.74 (t, *J* = 7.4 Hz, 4H, H9,11), 2.02 (p, *J* = 7.5 Hz, 2H, H10), 1.95 (s, 3H, H7), 1.88 (s, 3H, H17) ppm.

^{13}C{^{1}H} NMR (101 MHz, $CDCl_3$, δ): 142.2 (s, C16), 138.2 (d, $^1J_{C,P}$ = 8.4 Hz, C18), 137.9 (d, $^2J_{C,P}$ = 28.6 Hz, C14), 137.1 (d, $^3J_{C,P}$ = 8.4 Hz, C13), 136.1 (s, C6), 135.9 (s, C5), 135.1 (s, C12), 134.6 (s, C8), 133.5 (d, $^1J_{C,P}$ = 26.8 Hz, C15), 133.1 (d, $^2J_{C,P}$ = 19.5 Hz, C19), 128.8 (s, C21), 128.5 (d, $^3J_{C,P}$ = 7.0 Hz, C20), 128.1 (s, C4), 123.1 (q, $^1J_{C,F}$ = 286.0 Hz, C1), 38.5 (s, C9/11), 38.4 (s, C9/11), 23.1 (s, C10), 14.6 (s, C17), 14.3.(s, C7) ppm (not all ^{13}C signals were detected due to coupling to ^{19}F).

^{19}F NMR (376 MHz, $CDCl_3$, δ): -71.9 (s) ppm.

^{31}P{^{1}H} NMR (162 MHz, $CDCl_3$, δ): -19.6 (s) ppm.

IR (ATR, ṽ): 3053 (w, νC–H), 2918 (w, νC–H), 1681 (s, νC=O), 1585 (w), 1528 (w), 1478 (s), 1373 (w, νCF_3), 1220 (s, νCF_3), 1167 (s, νCF_3), 1069 (m), 1026 (m), 969 (w), 922 (s), 868 (m), 803 (s), 739 (m), 717 (s), 619 (m), 554 (s), 501 (s) cm^{-1}.

HRMS (ESI-TOF, m/z): calculated for $[M+H]^+$ 541.1031; found 541.1026.

UV-vis (cyclohexane, λ_{max} (ε)): 268 (35 673), 339 (6 431) nm (M^{-1} cm^{-1}).

{4-[2-(2-Methyl-5-(2,3,4,5,6-pentafluorophenyl)-thiophen-3-yl]cyclopent-1-en-1-yl}-5-methylthiophen-2-yl)diphenylphosphine (**DTE°-C$_6$F$_5$**)

A stirred solution of 0.327 g (0.7 mmol, 1.0 eq.) **2** in 9 mL THF was cooled to -78 °C (hexane/$N_{2(l)}$), and 0.85 mL (1.6 mol·L^{-1}, 1.4 mmol, 2.0 eq.) *t*BuLi in pentane were then added dropwise. The resultant deep red mixture was kept stirring for 45 min at -78 °C. In order to avoid complications during the purification step, full lithiation of the substrate was checked by ^{1}H NMR spectroscopy after quenching a reaction aliquot with methanol. After that, 0.55 mL (4.8 mmol, 7.0 eq.) of hexafluorobenzene were added in one swift motion. The reaction mixture turned yellow and was left to warm up to room temperature over an hour. After quenching with a degassed brine solution, the organic phase was separated, the aqueous phase was extracted with THF (2 x 5 mL), and the combined organic phases were dried over degassed Na_2SO_4. After canula filtration, the product mixture was absorbed on silica gel and purified through flash column

chromatography (hexanes/dichloromethane gradient from 95:5 to 70:30). After solvent removal *in vacuo*, a colourless oil was obtained (0.290 g, 70% yield).

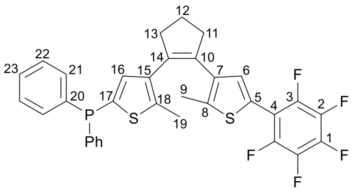

R$_f$ 0.18 (hexanes/dichloromethane 90:10)

^{1}H NMR (400 MHz, $CDCl_3$, δ): 7.37 – 7.33 (m, 10H, H21-23), 7.16 (s, 1H, H6), 7.01 (d, $^2J_{H,P}$ = 6.6 Hz, 1H, H16), 2.82 – 2.78 (m, 4H, H11,13), 2.10 – 2.02 (m, 5H, H12,19), 1.96 (s, 3H, H9) ppm.

^{13}C{^{1}H} NMR (101 MHz, $CDCl_3$, δ): 142.2 (s, C18), 138.1 (d, $^1J_{C,P}$ = 8.5 Hz, C20), 137.8 (d, $^2J_{C,P}$ = 27.8 Hz, C16), 136.9 (d, $^3J_{C,P}$ = 8.4 Hz, C15), 136.4 (s, C7), 135.5, 134.3, 133.6 (d, $^1J_{C,P}$ = 27.7 Hz, C17), 133.2 (d, $^2J_{C,P}$ = 19.7 Hz, C21), 133.0 (t, $^2J_{C,F}$ = 5.5 Hz, C6), 128.9 (s, C23), 128.6 (d, $^3J_{C,P}$ = 6.9 Hz, C22), 128.2, 38.5 (s, C11/13), 38.4 (s, C11/13), 23.1 (s, C12), 14.7 (s, C9), 14.2 (s, C19) ppm (not all ^{13}C signals were detected due to high substitution pattern and coupling to ^{19}F).

^{19}F NMR (376 MHz, $CDCl_3$, δ): -140.2 – 140.3 (m, 2F, F3), -157.1 (t, J = 21.1 Hz, 1F, F1), -162.4 – -162.5 (m, 2F, F2) ppm.

^{31}P{^{1}H} NMR (162 MHz, $CDCl_3$, δ): -19.5 (s) ppm.

IR (ATR, ṽ): 3052 (w, νC–H), 2921 (w, νC–H), 2846 (w), 1518 (s, νC-F), 1494 (s), 1433 (m), 1413 (w), 1326 (w), 1277 (w), 1239 (w), 1207 (w), 1119 (w), 1053 (m), 1037 (m), 1026 (s), 984 (w), 917 (m), 881 (m), 851 (w), 809 (s), 766 (w), 741 (s), 693 (w), 650 (w), 620 (w), 577 (s), 528 (m), 517 (s), 502 (m) cm^{-1}.

HRMS (ESI-TOF, m/z): calculated for $[M+H]^+$ 611.1050; found 611.1039.

UV-vis (cyclohexane, λ_{max} (ε)): 285 (23 756) nm (M^{-1} cm^{-1}).

5-Chloro-2-methyl-3-[2-(2-methyl-5-phenyl-3-thienyl)-1-cyclopenten-1-yl]thiophene (**DTE3**)

Compound **DTE3** was synthesized following a modified procedure.[5] A stirred solution of 0.532 mg (1.6 mmol, 1.0 eq.) **DTE1**[6] in 10 mL THF was cooled to -78 °C (hexane/$N_{2(l)}$) and 1.16 mL (1.6 mol·L^{-1}, 1.9 mmol, 1.2 eq.) *t*BuLi in pentane were added dropwise. After 15 min, 0.52 mL (1.9 mmol, 1.2 eq.) tri-*n*-butyl borate was added in one swift motion and reaction mixture was left stirring at -78 °C for 30 min more. Once reaching room temperature, crude was added through a canula to a degassed biphasic solution THF/2M Na_2CO_3 (10 mL:10 mL) containing 0.22 mL (1.9 mmol, 1.2 eq.) iodobenzene and 0.075 g (0.06 mmol, 0.04 eq.) [Pd(PPh_3)$_4$]. The reaction mixture was left at reflux for 2 hours, cooled down to room temperature and 10 mL Et_2O were added. The organic phase was separated, the aqueous phase was further extracted with Et_2O (2 x 10mL) and the combined organic phases were dried over anhydrous Na_2SO_4. The solvent was evaporated, and the crude was purified by flash column chromatography (hexanes 100) yielding 0.532 g (83% yield) of a clear oil.

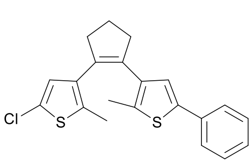

R$_f$ 0.56 (hexanes 100)

^{1}H NMR (300 MHz, $CDCl_3$, δ): 7.46 (m, 2H), 7.29 (m, 2H), 7.17 (m, 1H), 6.97 (s, 1H), 6.60 (s, 1H), 2.80 – 2.68 (m, 4H), 2.02 – 1.96 (m, 5H), 1.85 (s, 3H) ppm.

^{13}C{^{1}H} NMR (75 MHz, $CDCl_3$, δ): 140.0, 136.5, 135.4, 135.3, 134.6, 134.6, 133.9, 133.4, 129.0, 127.2, 127.0, 125.4, 125.2, 123.9, 38.7, 38.6, 23.1, 14.6, 14.4.

{4-[2-(5-chloro-2-methylthiophen-3-yl)cyclopent-1-en-1-yl]-5-methylthiophen-2-yl}diphenylphosphine (**DTE-Ph**)

Compound **DTE-Ph** was synthesized following a modified procedure.[5] **DTE3** was dissolved in 30 mL Et_2O and 0.59 mL (1.6 mol·L^{-1}, 0.9 mmol, 1.1 eq.) *n*BuLi in hexanes were added dropwise at room temperature to a stirred solution. The resultant deep red mixture was kept stirring for 15 min, followed by the addition of 0.18 mL (1.0 mmol, 1.2 eq.) of chlorodiphenylphosphine in one swift motion. The reaction mixture was left overnight at room temperature, and then quenched with a degassed brine solution. Under nitrogen atmosphere, the phases were separated, the aqueous phase was extracted with Et_2O (2 x 10 mL), and the combined organic phases were dried over degassed Na_2SO_4. After canula filtration, the product mixture was absorbed on silica gel and purified through column chromatography (hexanes/dichloromethane 90:10). After solvent removal *in vacuo*, a white oil was obtained (0.305 g, 68% yield).

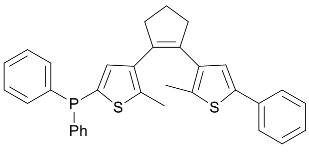

R_f 0.34 (hexanes/dichloromethane 90:10)

^{1}H NMR (400 MHz, $CDCl_3$, δ): 7.48 (m, 2H), 7.36 – 7.23 (m, 13H), 7.02 (d, *J* = 6.4 Hz, 1H), 6.97 (s, 1H), 2.87 – 2.75 (m, 4H), 2.09 (p, *J* = 7.5 Hz, 2H), 2.03 (s, 3H), 2.01 (s, 3H) ppm.

^{13}C{^{1}H} NMR (101 MHz, $CDCl_3$, δ): 142.0, 139.7, 138.1 (d, *J* = 1.9 Hz), 137.9 (d, *J* = 20.0 Hz), 137.1 (d, *J* = 8.0 Hz), 136.6, 135.0, 134.5, 134.3, 133.2, 133.1 (d, *J* = 19.6 Hz), 133.0, 131.7 (d, *J* = 10.3 Hz), 128.7 (d, *J* = 10.7 Hz), 128.4 (d, *J* = 7.0 Hz), 127.0, 125.3, 124.0, 38.3, 38.3, 23.0, 14.6, 14.4 ppm.

^{31}P{^{1}H} NMR (162 MHz, $CDCl_3$, δ): -19.6 (s) ppm.

UV-vis (cyclohexane, λ_{max} (ε)): 273 (28 117) nm (M^{-1} cm^{-1}).

[$PdCl_2$(**DTE°-COCF$_3$**)$_2$]

0.014 g (0.04 mmol, 0.5 eq.) [$PdCl_2(PhCN)_2$] and 0.040 g (0.02 mmol, 1.0 eq.) **DTE-COCF$_3$** were dissolved in 2 mL CH_2Cl_2. The reaction mixture was stirred at 50 °C overnight and then cooled down to room temperature. After solvent removal *in vacuo*, the complex was crystallized from CH_2Cl_2/pentane, yielding a yellow solid (0.037 g, 80% yield). Single crystals suitable for X-ray structure analysis were obtained by slow evaporation from CH_2Cl_2/Et_2O.

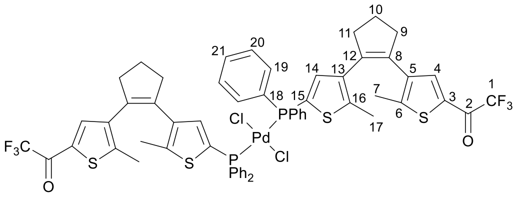

^{1}H NMR (300 MHz, $CDCl_3$, δ): 7.65 – 7.52 (m, 10H, H4,19), 7.48 – 7.29 (m, 12H, H20,21), 7.16 (t, $^2J_{H,P}$ = 3.4 Hz, 2H, H14), 2.85 – 2.70 (m, 8H, H9/11), 2.15 – 2.00 (m, 16H, H10, 7, 17) ppm.

^{13}C{^{1}H} NMR (75 MHz, $CDCl_3$, δ): 172.8 (q, $^2J_{C,F}$ = 36.1 Hz, C2), 150.3 (s, C6), 144.2 (s, C16), 140.6 (t, $^2J_{C,P}$ = 4.9 Hz, C14), 138.5 (s, C5), 137.7 (q, $^3J_{C,F}$ = 3.1 Hz, C4), 136.8 (s, C8/12), 136.2 (t, $^3J_{C,P}$ = 5.1 Hz, C13), 134.3 (t, $^2J_{C,P}$ = 6.5 Hz, C19), 133.2 (s, C8/12), 132.1 (s, C3), 130.7 (s, C21), 130.1 (t, $^1J_{C,P}$ = 26.1 Hz, C18), 128.0 (t, $^3J_{C,P}$ = 5.5 Hz, C20), 125.6 (t, $^1J_{C,P}$ = 25.1 Hz, C15), 116.5 (q, $^1J_{C,F}$ = 114.5 Hz, C1), 38.2 (s, C9/11), 38.2 (s, C9/11), 22.9 (s, C10), 15.5 (s, C17), 14.6 (s, C7) ppm (not all ^{13}C signals were detected due to coupling to ^{19}F).

^{19}F NMR (282 MHz, $CDCl_3$, δ): -71.8 (s) ppm.

^{31}P{^{1}H} NMR (162 MHz, $CDCl_3$, δ): 12.2 (s) ppm.

IR (ATR, ṽ): 2959 (w, νC–H), 2922 (w, νC–H), 2848 (w, νC–H), 1683 (m, νC=O), 1539 (w), 1478 (w), 1434 (m), 1420 (m), 1373 (w, νCF_3), 1350 (w), 1328 (w), 1305 (w), 1260 (m), 1217 (m, νCF_3), 1184 (m), 1166 (s, νCF_3), 1141 (s), 1094 (s), 1014 (s), 866 (s), 848 (m), 796 (s), 739 (s), 716 (m), 685 (s), 663 (m), 581 (w), 530 (s), 503 (s) cm^{-1}.

HRMS (ESI-TOF, m/z): calculated for $[M-Cl]^+$ 1221.0640, found 1221.0600.

UV-vis (cyclohexane, λ_{max} (ε)): 285 (31 151), 355 (19 065) nm (M^{-1} cm^{-1}).

[$PdCl_2$(**DTE^c-COCF3**)$_2$]

A closed state-enriched mixture of complex [$PdCl_2$(**DTE-COCF$_3$**)$_2$] was prepared by first isomerizing the ligand **DTE^o-COCF$_3$** in cyclohexane solution and then complexing with palladium(II). In the dark, 0.100 g (0.18 mmol, 1.0 eq.) **DTE^o-COCF$_3$** were dissolved in degassed cyclohexane and irradiated with 365 nm until no further change in the PSS composition was observed by ^{19}F NMR spectroscopy. A stock solution of 0.032 g (0.08 mmol, 0.5 eq.) [$PdCl_2(PhCN)_2$] in 5.7 mL CH_2Cl_2 was added to the ligand and the reaction was kept at 50 °C overnight. After cooling down to room temperature, the solvent was removed *in vacuo* and the product was obtained after flash column chromatography (hexanes/ethyl acetate gradient from 70:30 to 30:70) yielding a deep blue solid – closed state-enriched complex mixture (0.101 g, 87%) with the composition: **cc** 52%, **oc** 40% and **oo** 7%.

^{19}F NMR (377 MHz, $CDCl_3$, δ): -71.7 (s, **cc**), -71.7 (s, **oc**), -72.1 (s, **oc**+**oo**) ppm.

^{31}P{^{1}H} NMR (122 MHz, $CDCl_3$, δ): 18.6 (d, *J* = 582.0 MHz, **oc**), 18.4 (s, **cc**), 12.2 (s, **oo**), 12.1 (d, *J* = 582.0 MHz, **oc**) ppm.

[$PdCl_2$(**DTE^o-C$_6$F$_5$**)$_2$]

Complex [$PdCl_2$(**DTE^o-C$_6$F$_5$**)$_2$] was synthesized following the procedure described for [$PdCl_2$(**DTE^o-COCF$_3$**)$_2$] using 0.087 g (0.14 mmol, 1 eq.) **DTE^o-C$_6$F$_5$** as a ligand and 0.027 g (0.07 mmol, 0.5 eq.) [$PdCl_2(PhCN)_2$] in 5 mL CH_2Cl_2. After complexation was completed, the solvent was removed *in vacuo* and the product was precipitated from Et_2O/pentane as a yellow solid (0.088 g, 90%). Single crystals suitable for X-ray structure analysis were obtained by slow vapor diffusion of pentane into a CH_2Cl_2 solution.

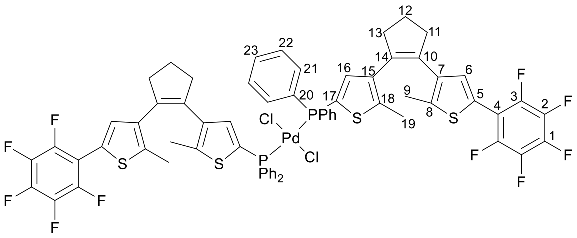

^{1}H NMR (300 MHz, $CDCl_3$, δ): 7.61 (q, $^2J_{H,P}$ = 6.0, 3.5 Hz, 8H, H21), 7.45 – 7.29 (m, 14H, H16, 22, 23), 7.13 (s, 2H, H6), 2.78 (t, *J* = 7.5 Hz, 8H, H11, 13), 2.03 – 1.98 (m, 16H, H12, 9, 19) ppm.

^{13}C{^{1}H} NMR (75 MHz, $CDCl_3$, δ): 144.2 (s, C18), 141.1 (t, $^2J_{C,P}$ = 4.9 Hz, C16), 138.0 (s, C8), 136.9 (t, $^3J_{C,P}$ = 5.4 Hz, C15), 136.1 (s, C7), 135.0 (s, C10/14), 134.5 (s, C10/14), 134.3 (t, $^2J_{C,P}$ = 6.5 Hz, C21), 131.4 (s, C6), 130.6 (s, C23), 130.5 (t, $^1J_{C,P}$ = 26.3 Hz, C20), 127.9 (t, $^3J_{C,P}$ = 5.4 Hz, C22), 124.8 (t, $^1J_{C,P}$ = 24.6, C17), 122.2 (m, C5), 38.4 (s, C11/13), 22.9 (s, C12), 14.5 (s, C9), 14.2 (s, C19) ppm (not all ^{13}C signals were detected due to high substitution pattern and coupling to ^{19}F).

^{19}F NMR (282 MHz, $CDCl_3$, δ): -140.1 – -140.2 (m, 2F, F3), -156.9 (t, *J* = 21.1 Hz, 1F, F1), -162.2 – -162.4 (m, 2F, F2) ppm.

^{31}P{^{1}H} NMR (122 MHz, $CDCl_3$, δ): 12.1 (s) ppm.

IR (ATR, ṽ): 3070 (w, νC–H), 2961 (w, νC–H), 2838 (w), 1517 (m, νC-F), 1494 (s), 1435 (m), 1344 (w), 1309 (w), 1260 (m), 1211 (w), 1155 (w), 1097 (s), 1062 (m), 1027 (s), 984 (s), 851 (m), 805 (s), 744 (s), 690 (s), 576 (w), 558 (w), 531 (s), 497 (s) cm^{-1}.

HRMS (ESI-TOF, m/z): calculated for $[M-Cl]^+$ 1361.0678, found 1361.0710.

UV-vis (cyclohexane, λ_{max} (ε)): 286 (33 147), 357 (14 367) nm (M^{-1} cm^{-1}).

[$PdCl_2$(**DTE^c-C$_6$F$_5$**)$_2$]

A closed state-enriched mixture of complex [$PdCl_2$(**DTE-C$_6$F$_5$**)$_2$] was synthesized following the procedure described for [$PdCl_2$(**DTE^c-COCF$_3$**)$_2$]. First, 0.132 g (0.22 mmol, 1 eq.) **DTE^o-C$_6$F$_5$** were photoisomerized in cyclohexane using a 312 nm lamp, then a stock solution of 0.041 mg (0.11 mmol, 0.5 eq.) [$PdCl_2(PhCN)_2$] in 6.6 mL CH_2Cl_2 was added. After stirring overnight at 50 °C and cooling down to room temperature, the solvent was evaporated *in vacuo* and the product was obtained after flash column chromatography (hexanes/ethyl acetate gradient from 70:30 to 30:70) yielding a deep pink solid – closed state-enriched complex with the composition: **cc** 38%, **oc** 48% and **oo** 14%.

^{19}F NMR (282 MHz, $CDCl_3$, δ): -137.94 (dd, *J* = 22.9, 7.1 Hz, **oc**+**cc**), -140.88 (dd, *J* = 22.9, 7.1 Hz, **oc**+**oo**), -154.99 (m, **oc**+**cc**), -157.78 (m, **oc**+**oo**), -162.07 – -162.68 (m, **oc**+**cc**), -162.67 – -163.27 (m, **oc**+**oo**) ppm.

^{31}P{^{1}H} NMR (162 MHz, $CDCl_3$, δ): 17.9 (d, *J* = 583.6 Hz, **oc**), 17.7 (s, **cc**), 12.1 (s, **oo**), 12.1 (d, *J* = 583.2 Hz, **oc**) ppm.

[$PdCl_2$(**DTE^o-Ph**)$_2$]

Complex [$PdCl_2$(**DTE^o-Ph**)$_2$] was synthesized following the procedure described for [$PdCl_2$(**DTE^o-COCF$_3$**)$_2$] using 0.094 g (0.18 mmol, 1 eq.) **DTE^o-Ph** as a ligand and 0.035 g (0.09 mmol, 0.5 eq.) [$PdCl_2(PhCN)_2$] in 5 mL CH_2Cl_2. After complexation was completed, the solvent was removed *in vacuo* and the product was precipitated from CH_2Cl_2/pentane as a yellow solid (0.093 g, 86%). Single crystals suitable for X-ray structure analysis were obtained by slow evaporation from CH_2Cl_2/Et_2O.

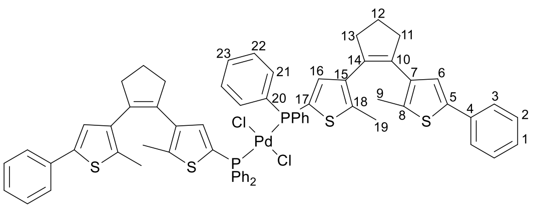

^{1}H NMR (400 MHz, $CDCl_3$, δ): 7.60 (m, 8H, H21), 7.45 (d, *J* = 7.4 Hz, 4H, H3), 7.37 – 7.26 (m, 18H, H2, 16, 22, 23), 7.21 (t, *J* = 7.3 Hz, 2H, H1), 6.91 (s, 2H, H6), 2.77 (t, *J* = 7.5 Hz, 8H, H11, 13), 2.06 – 1.96 (m, 16H, H12, 9, 19) ppm.

^{13}C{^{1}H} NMR (101 MHz, $CDCl_3$, δ): 144.2 (s, C18), 141.2 (t, $^2J_{C,P}$ = 4.9 Hz, C16), 139.7 (C5), 137.1 (t, $^3J_{C,P}$ = 5.0 Hz, C15), 136.3 (s, C7/8), 135.3 (C10,14), 134.3 (s, C7/8), 134.2 (t, $^2J_{C,P}$ = 6.0 Hz, C21), 134.0 (s, C4), 130.4 (t, $^1J_{C,P}$ = 26.0 Hz, C20), 130.4 (s, C23), 128.7 (s, C3), 127.8 (t, $^3J_{C,P}$ = 5.5 Hz, C22), 126.9 (s, C1), 125.2 (C2), 124.5 (t, $^1J_{C,P}$ = 25.4 Hz, C17), 123.8 (s, C6), 38.3 (s, C11/13), 38.1 (s, C11/13), 22.8 (s, C12), 14.5 (s, C9/19), 14.4 (s, C9/19) ppm.

^{31}P{^{1}H} NMR (122 MHz, $CDCl_3$, δ): 12.2 (s) ppm.

IR (ATR, ṽ): 3107 (w, νC–H), 3050 (w, νC–H), 2962 (m, νC–H), 2922 (m, νC–H), 2848 (w), 1596 (w), 1498 (w), 1482 (w), 1463 (w), 1435 (m), 1410 (w), 1309 (w), 1261 (m), 1214 (w), 1095 (s), 1026 (m), 861 (w), 845 (w), 802 (m), 760 (m), 751 (w), 739 (w), 707 (w), 694 (m), 687 (m), 658 (w), 526 (m), 513 (m), 495 (m) cm^{-1}.

HRMS (ESI-TOF, m/z): calculated for $[M-Cl]^+$ 1181.1620, found 1181.1617.

UV-vis (cyclohexane, λ_{max} (ε)): 270 (42 700), 359 (15 400) nm (M^{-1} cm^{-1}).

[$PdCl_2$(**DTE^c-Ph**)$_2$]

A closed state-enriched mixture of complex [$PdCl_2$(**DTE-Ph**)$_2$]was synthesized following the procedure described for [$PdCl_2$(**DTE^c-COCF$_3$**)$_2$]. First 0.058 g (0.11 mmol, 1 eq.) **DTE^o-Ph** were photoisomerized in cyclohexane using a 312 nm lamp, then stock solution of 0.021 mg (0.05 mmol, 0.5 eq.) [$PdCl_2(PhCN)_2$] in

4.0 mL CH_2Cl_2 was added. After stirring overnight at 50 °C and cooling down to room temperature, the solvent was evaporated *in vacuo* and the product was obtained after flash column chromatography (hexanes/ethyl acetate 70:30) yielding a deep pink solid – closed state-enriched complex with the composition: **cc** 49%, **oc** 39% and **oo** 12%.

$^{31}P\{^1H\}$ NMR (122 MHz, $CDCl_3$, δ): 18.2 (d, *J* = 587.5 Hz, **oc**), 17.6 (s, **cc**), 12.2 (s, **oo**), 11.7 (d, *J* = 587.4 Hz, **oc**) ppm.

Preparation of phosphine selenides

Phosphine selenides were prepared by the addition of selenium grey powder to the NMR tube containing the free ligand in $CDCl_3$ and leaving the tube at 30 °C for 30 min. The reaction proceeded with 100% yield. The obtained selenides were not isolated and studied *in situ*.

1.3 Crystallographic data

Table S1 – Fundamental structure parameters

Compound	[$PdCl_2$(**DTE°-$COCF_3$**)$_2$]	[$PdCl_2$(**DTE°-C_6F_5**)$_2$]	[$PdCl_2$(**DTE°-Ph**)$_2$]
Molecular formula	$C_{58}H_{48}Cl_2F_6O_2P_2PdS_4$	$C_{66}H_{48}Cl_2F_{10}P_2PdS_4 \cdot 2\ CH_2Cl_2$	$C_{66}H_{58}Cl_2P_2PdS_4$
Empirical formula	$C_{58}H_{48}Cl_2F_6O_2P_2PdS_4$	$C_{68}H_{52}Cl_6F_{10}P_2PdS_4$	$C_{66}H_{58}Cl_2P_2PdS_4$
Formula weight	1258.44	1568.37	1218.60
Temperature [K]	130(2)	130(2)	130(2)
Wavelength [pm]	71.073	71.073	71.073
Crystal system	Triclinic	Triclinic	Monoclinic
Space group	$P\,\bar{1}$	$P\,\bar{1}$	$P2_1/n$
Unit cell dimensions			
a [pm]	939.39(6)	779.12(6)	1132.74(2)
b [pm]	1016.51(6)	1191.26(7)	2230.11(2)
c [pm]	1548.13(9)	1943.0(1)	1192.49(2)
α [deg]	75.581(5)	102.024(5)	90
β [deg]	75.020(5)	99.679(6)	113.502(2)
γ [deg]	73.269(5)	107.099(6)	90
Volume [nm^3]	1.3430(2)	1.6343(2)	2.76250(8)
Z	1	1	2
$\rho_{(calculated)}$ [Mg/m^3]	1.556	1.594	1.465
μ [mm^{-1}]	0.725	0.777	0.685
F(000)	640	792	1256
Crystal size [mm^3]	0.25 · 0.14 · 0.03	0.17 · 0.16 · 0.01	0.44 · 0.21 · 0.10
Θ_{Min} / Θ_{Max} [deg]	2.311 / 27.479	2.766 / 28.166	2.288 / 32.517
Index ranges	-12 ≤ h ≤ 12<br>-12 ≤ k ≤ 13<br>-19 ≤ l ≤ 19	-10 ≤ h ≤ 10<br>-14 ≤ k ≤ 15<br>-25 ≤ l ≤ 25	-15 ≤ h ≤ 16<br>-33 ≤ k ≤ 33<br>-17 ≤ l ≤ 17
Reflections collected	9885	20166	37948
Indp. reflections (R_{int})	9885 (0.0480)	6951 (0.1070)	9263 (0.0384)
Completeness (Θ_{Max})	99.9 % (25.35)	99.9 % (25.35)	100.0 % (30.51)
T_{Max} / T_{Min}	1.00000 / 0.99350	0.992 / 0.895	1.00000 / 0.98121
Restraints / parameters	3 / 350	19 / 446	6 / 346
Gof on F^2	0.799	1.020	1.038
R1 / wR2 (I>2σ(I))	0.0391, 0.0655	0.0766, 0.1201	0.0368, 0.0784
R1 / wR2 (all data)	0.0813, 0.0705	0.1627, 0.1503	0.0516, 0.0856
Residual electron density [e·Å^{-3}]	0.763 / -0.603	0.827 / -0.622	0.818 / -0.350
Comments	†[1]	†[2]	†[3]
CCDC No	2310549	2310550	2310551

†[1]: Two-component twin. Twin domain ratio 0.7984(4):0.2016(4). C19, C20 and C21 with envelope disorder (ratio 0.54(2):0.46(2)). †[2]: Disorder detectable for Cl1 (ratio 0.767(5):0.233(5)) and the solvent molecule C34, Cl2, Cl3 (ratio 0.91(1):0.09(1)). †[3]: C20 with envelope disorder (ratio 0.74(2):0.26(2)).

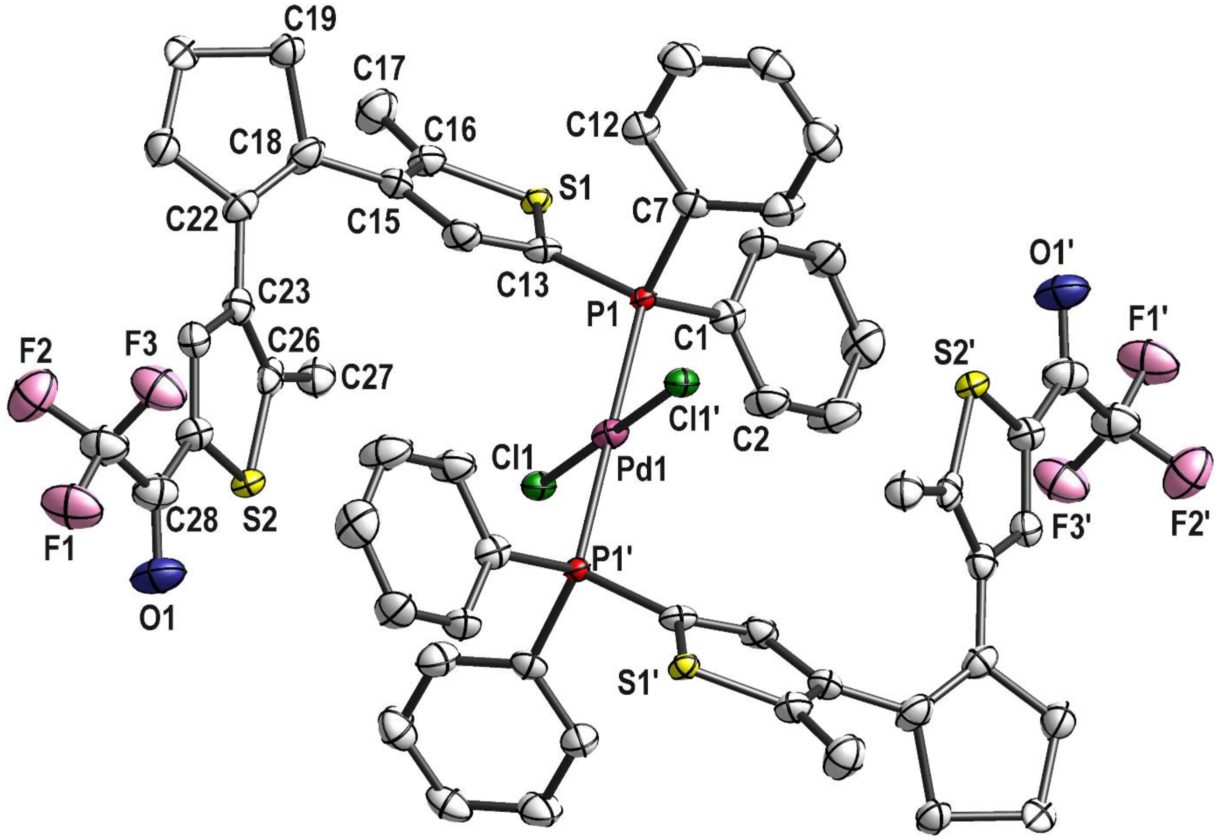

Figure S1. Molecular structure of [PdCl$_2$(**DTE°-COCF$_3$**)$_2$]. Hydrogen atoms and disordered atoms were omitted for clarity. Displacement ellipsoids are drawn at the 50 % probability level.

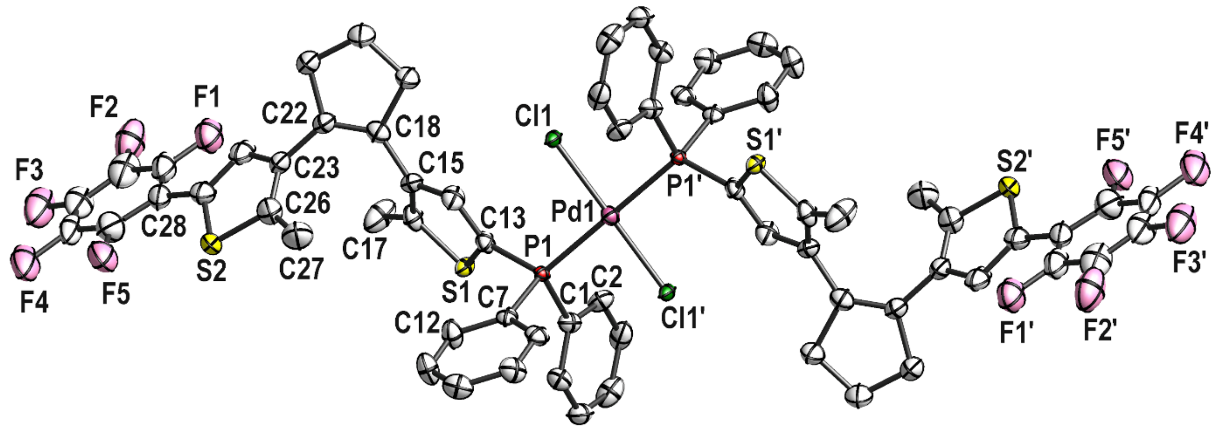

Figure S2. Molecular structure of [PdCl$_2$(**DTE°-C$_6$F$_5$**)$_2$]. Hydrogen atoms and disordered atoms were omitted for clarity. Displacement ellipsoids are drawn at the 50 % probability level.

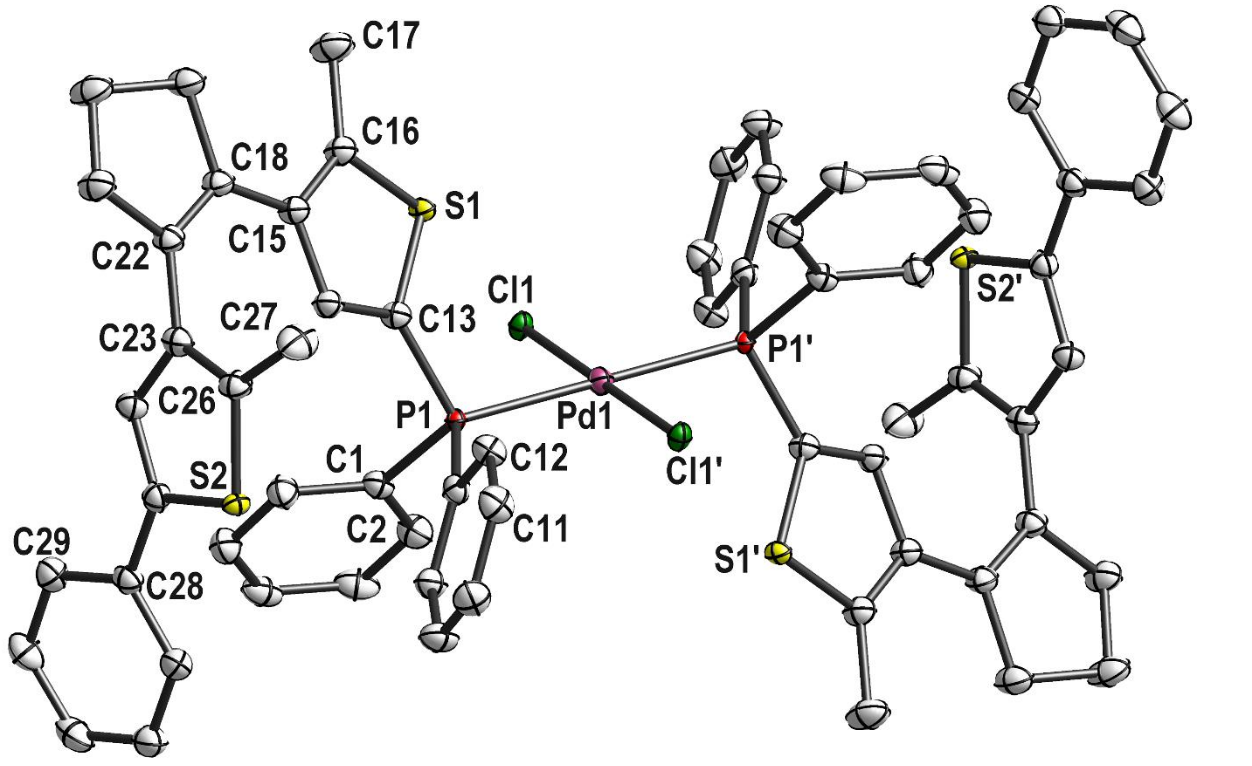

Figure S3. Molecular structure of [$PdCl_2$(**DTE°-Ph**)$_2$]. Hydrogen atoms and disordered atoms were omitted for clarity. Displacement ellipsoids are drawn at the 50 % probability level.

2 Experimental studies of the photochemical behavior of ligands and complexes

2.1 Solid-state photoisomerization of [PdCl$_2$(**DTE-C$_6$F$_5$**)$_2$]

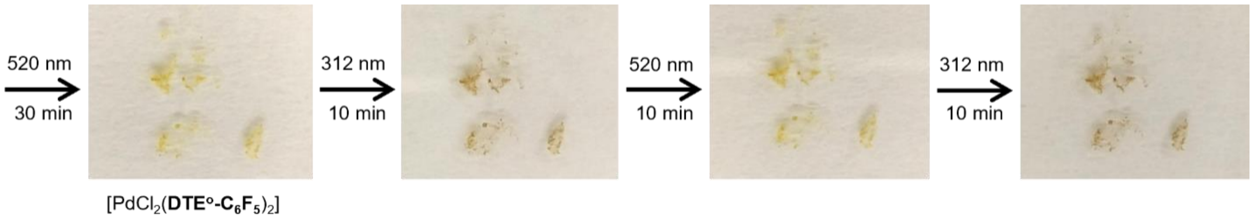

Figure S4. Illustration of the solid-state photoisomerization of [PdCl$_2$(**DTE-C$_6$F$_5$**)$_2$]. Complex crystals were first irradiated with λ_{exc} = 520 nm to ensure that no residual closed state ligand was initially present. Two cycles of subsequent irradiation with λ_{exc} = 312 nm (for photoinduced ring-closing) and λ_{exc} = 520 nm (for photoinduced ring-opening) are presented. Clear changes in color were observed for the crystals in these experiments, which reversibly switched from the characteristic yellow color of the open state [PdCl$_2$(**DTE°-C$_6$F$_5$**)$_2$] to the reddish color expected upon photocyclization of the DTE ligands. Therefore, these results prove that [PdCl$_2$(**DTE-C$_6$F$_5$**)$_2$] can successfully photoisomerize in the solid state.

2.2 Study of the photoisomerization of ligands and complexes by UV-vis absorption spectroscopy

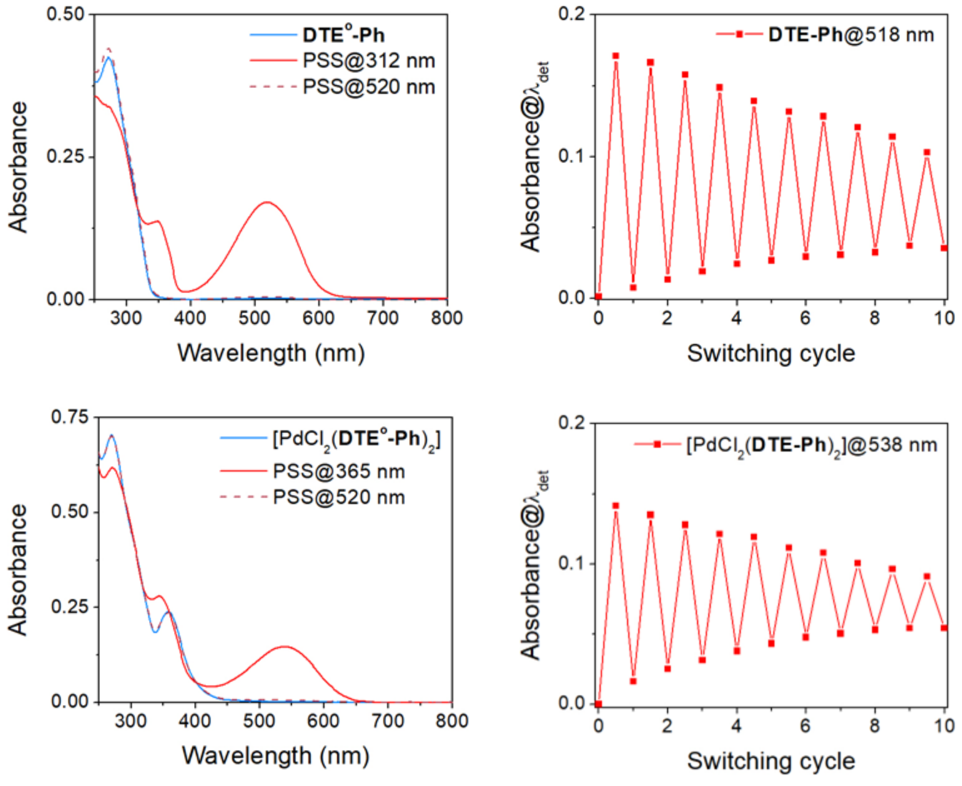

Figure S5. (Left) Variation of the absorption spectrum of the open state of the free ligand **DTE-Ph** (c = 1.5·10^{-5} M) and of the complex [PdCl$_2$(**DTE-Ph**)$_2$] (c = 1.5·10^{-5} M) in cyclohexane upon sequential irradiation with UV (λ_{exc} = 312 for 180 s or λ_{exc} = 365 nm for 150 s) and green light (λ_{exc} = 520 nm for 120 or 180 s, respectively) until the corresponding ring-closing and ring-opening PSSs were obtained. (Right) Variation of the absorbance at the spectral maximum of the ring-closed isomer of the ligand **DTE-Ph** (c = 1.5·10^{-5} M) (λ_{det} = 518 nm) and of the complex [PdCl$_2$(**DTE-Ph**)$_2$] (c = 1.5·10^{-5} M) (λ_{det} = 538 nm) in cyclohexane upon 10 consecutive photoswitching cycles. For ring-closing, UV irradiation was conducted at λ_{exc} = 312 (ligand) or 365 (complex) nm, while ring-opening was promoted with green light (λ_{exc} = 520 nm).

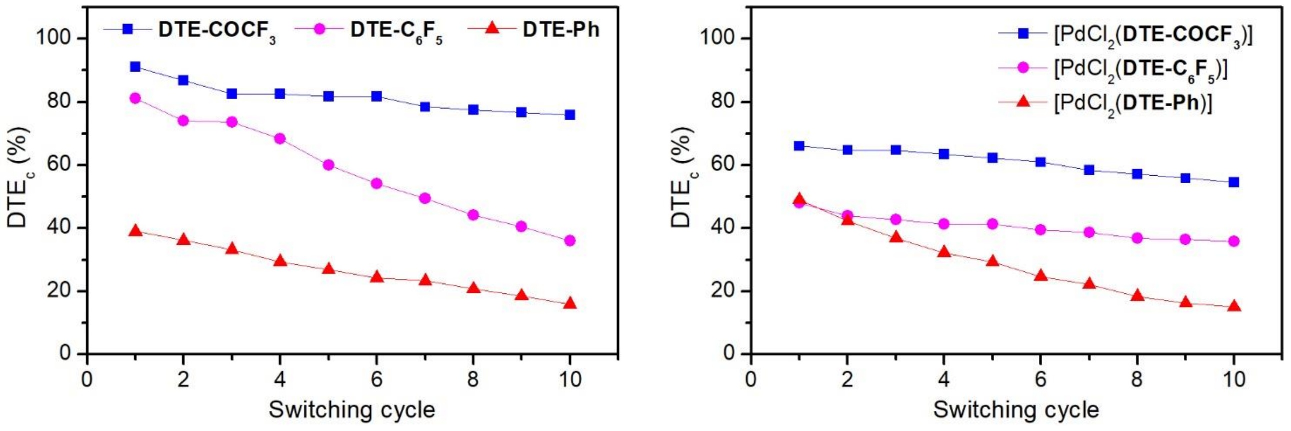

Figure S6. Fatigue effect on the photoisomerization of ligands and complexes. (Left) Variation of the percentage of ring-closed DTE units (DTE_c) in the UV-induced PSSs accomplished for free ligands **DTE-COCF$_3$** (λ_{exc} = 365 nm), **DTE-C$_6$F$_5$** (λ_{exc} = 312 nm) and **DTE-Ph** (λ_{exc} = 312 nm) upon consecutive photoswitching cycles. (Right) Variation of the percentage of ring-closed DTE units (DTE_c) in the UV-induced PSSs accomplished for the palladium complexes [PdCl$_2$(**DTE-COCF$_3$**)$_2$], [PdCl$_2$(**DTE-C$_6$F$_5$**)$_2$] and [PdCl$_2$(**DTE-Ph**)$_2$] upon consecutive photoswitching cycles (λ_{exc} = 365 nm). In all the cases, a clear decrease in DTEc is observed upon sequential switching, which is due to photodegradation.

2.3 NMR characterization of the photoisomerization of ligands and complexes

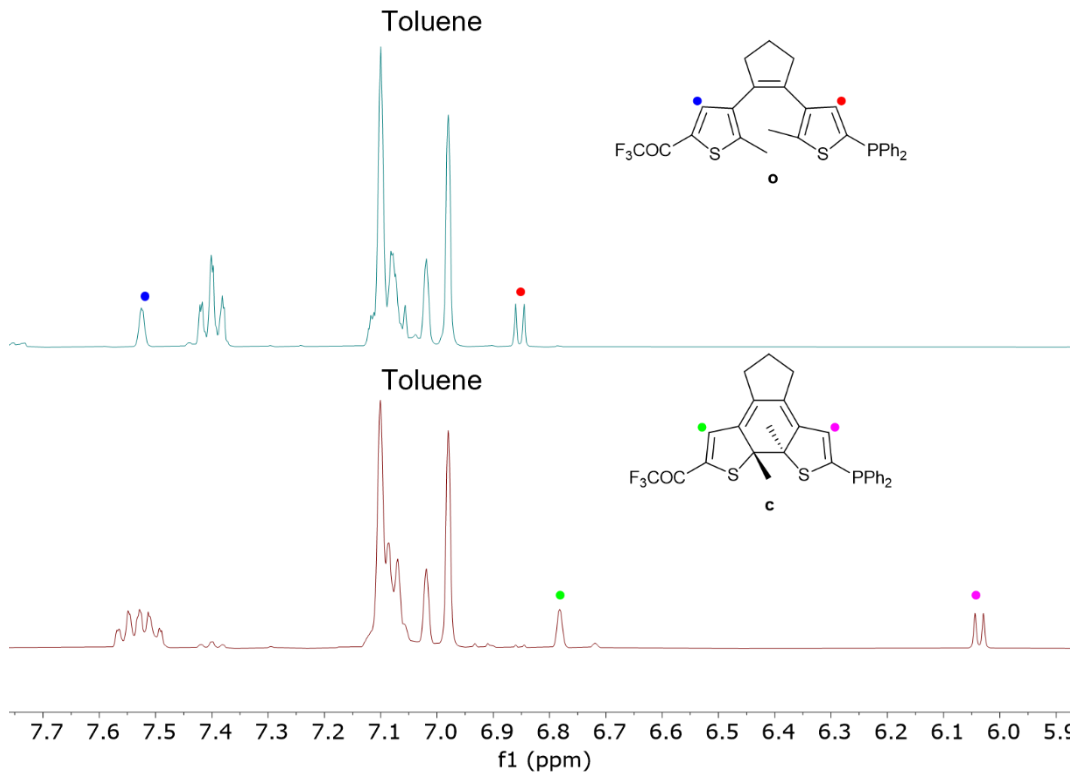

Figure S7. Changes in the low-field region of the ^{1}H NMR spectrum (toluene-d_8, 400 MHz) of **DTE-COCF$_3$** upon photocyclization: (top) open state ligand (**o**), (bottom) PSS@365 nm, where major conversion to the closed isomer (**c**) is observed. In both cases, the positions of the ^{1}H NMR signals of the sulfur-containing rings are marked.

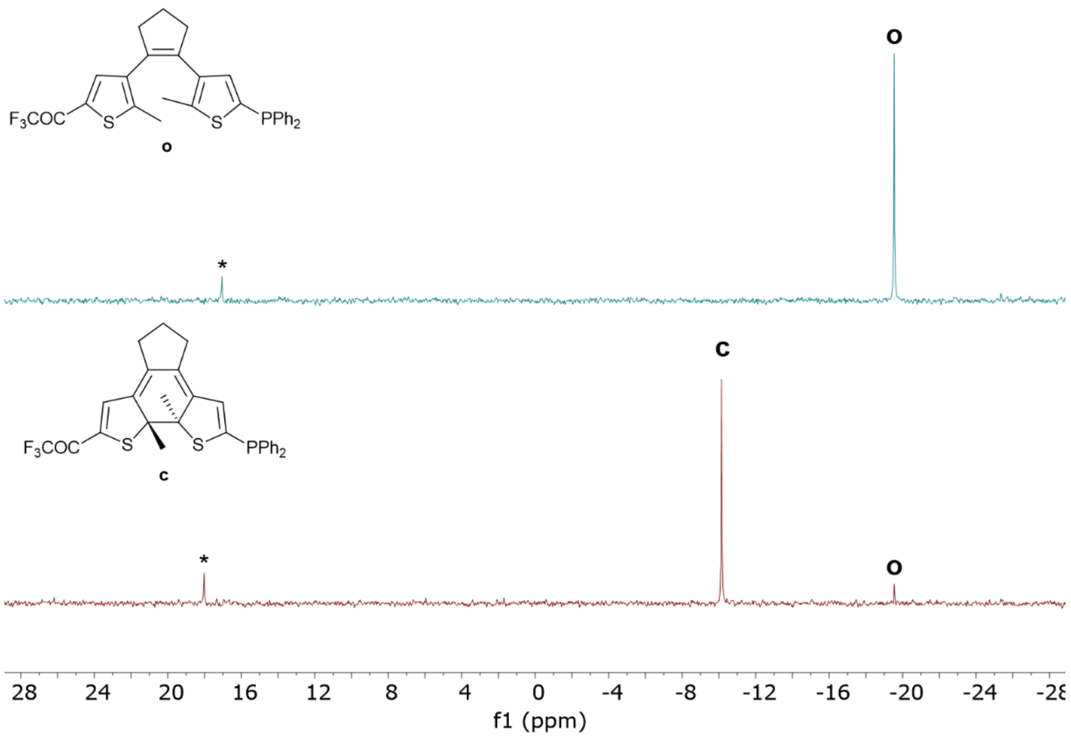

Figure S8. Changes in the ³¹P{¹H} NMR spectrum (toluene-d_8, 162 MHz) of **DTE-COCF₃** upon photocyclization: (top) open state ligand (**o**), (bottom) PSS@365 nm, where major conversion to the closed isomer (**c**) is observed. Additional low-intensity signals are found for the corresponding oxidized ligands (*).

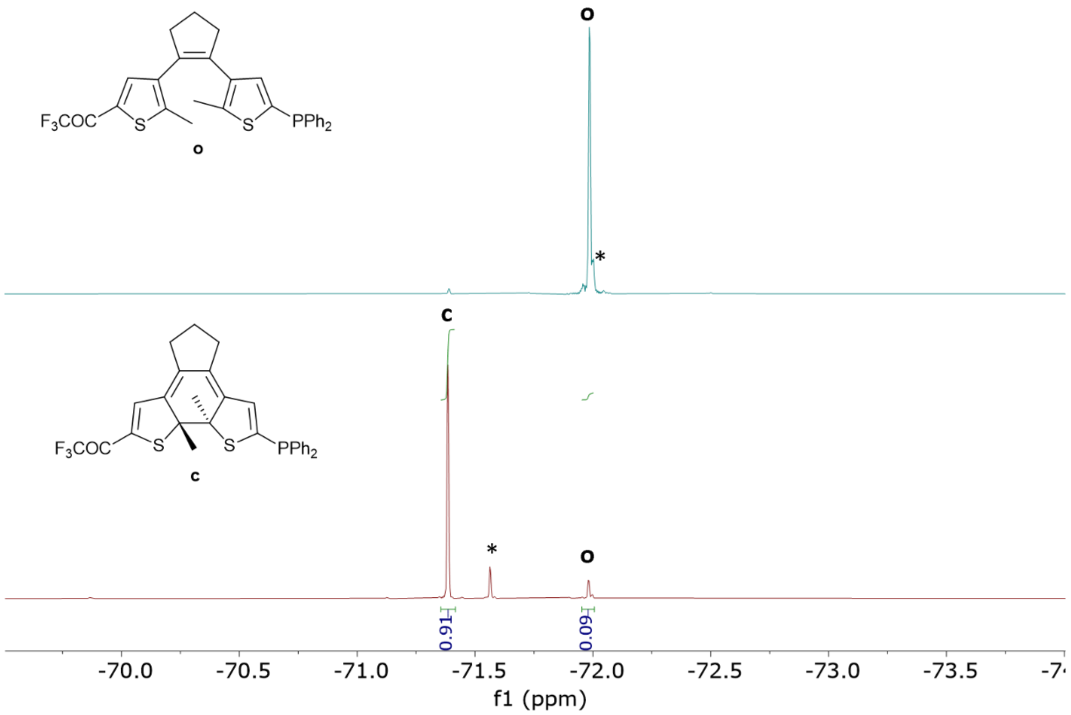

Figure S9. Changes in the ¹⁹F NMR spectra (toluene-d_8, 376 MHz) of **DTE-COCF₃** upon photocyclization: (top) open state ligand (**o**), (bottom) PSS@365 nm, where 91% conversion to the closed isomer (**c**) is determined from integrals. Additional low-intensity signals are found for the corresponding oxidized ligands (*).

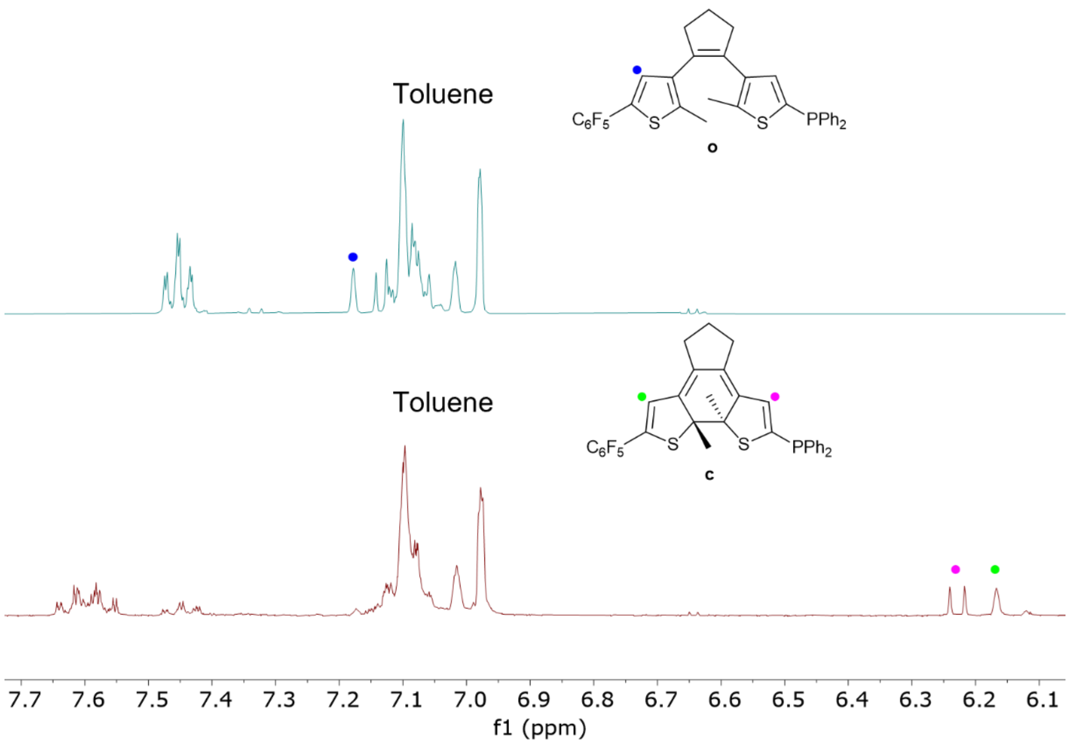

Figure S10. Changes in the low-field region of the ^{1}H NMR spectrum (toluene-d_8) of **DTE-C$_6$F$_5$** upon photocyclization: (top, 400 MHz) open state ligand (**o**); (bottom, 300 MHz) PSS@312 nm, where major conversion to the closed isomer (**c**) is observed. In both cases, the positions of the ^{1}H NMR signals of the sulfur-containing rings are marked.

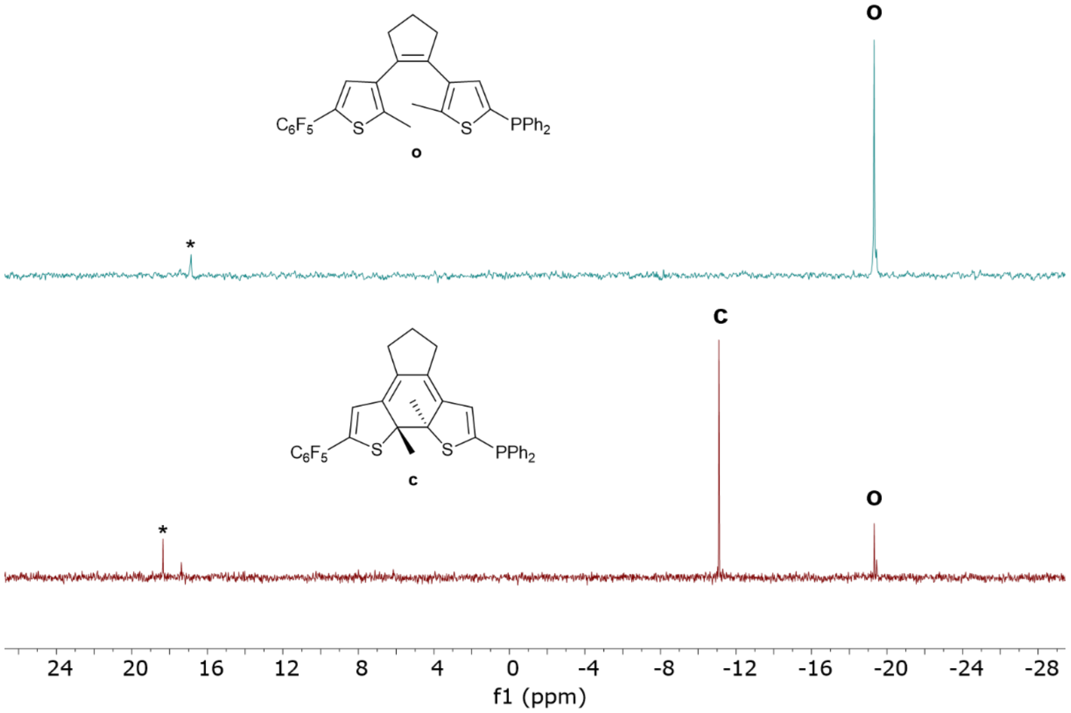

Figure S11. Changes in the $^{31}P\{^{1}H\}$ NMR spectrum (toluene-d_8) of **DTE-C$_6$F$_5$** upon photocyclization: (top, 162 MHz) open state ligand (**o**); (bottom, 121 MHz) PSS@312 nm, where major conversion to the closed isomer (**c**) is observed. Additional low-intensity signals are found for the corresponding oxidized ligands (*).

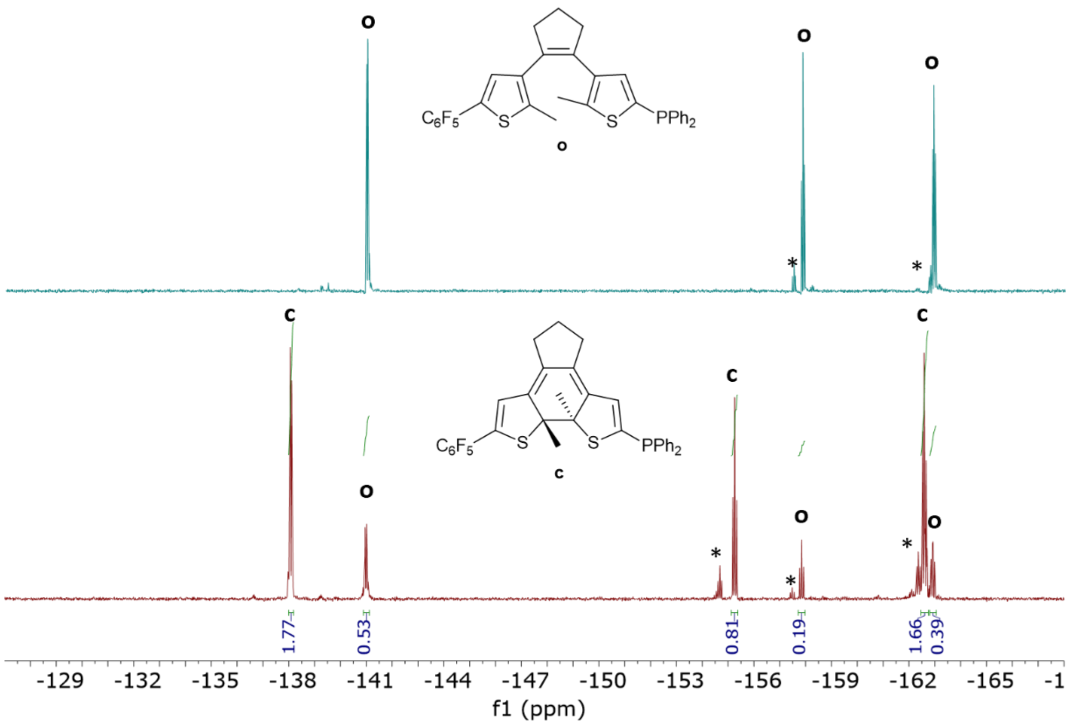

Figure S12. Changes in the ^{19}F NMR spectrum (toluene-d_8) of **DTE-C₆F₅** upon photocyclization: (top, 376 MHz) open state ligand (**o**); (bottom, 282 MHz) PSS@312 nm, where 81% conversion to the closed isomer (**c**) is determined from integrals. Additional low-intensity signals are found for the corresponding oxidized ligands (*).

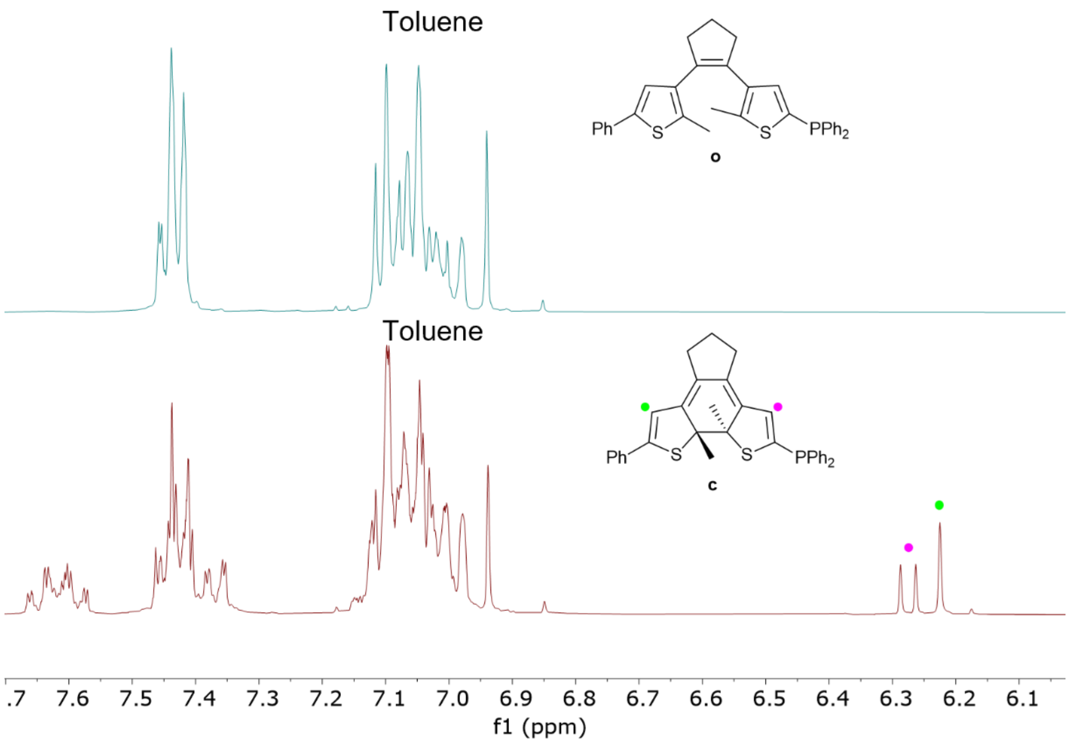

Figure S13. Changes in the low-field region of the ^{1}H NMR spectrum (toluene-d_8) of **DTE-Ph** upon photocyclization: (top, 400 MHz) open state ligand (**o**); (bottom, 300 MHz) PSS@312 nm, where partial conversion to the closed state (**c**) is observed. For the PSS@312 nm spectrum, the positions of the ^{1}H NMR signals of the sulfur-containing rings are marked for the closed isomer.

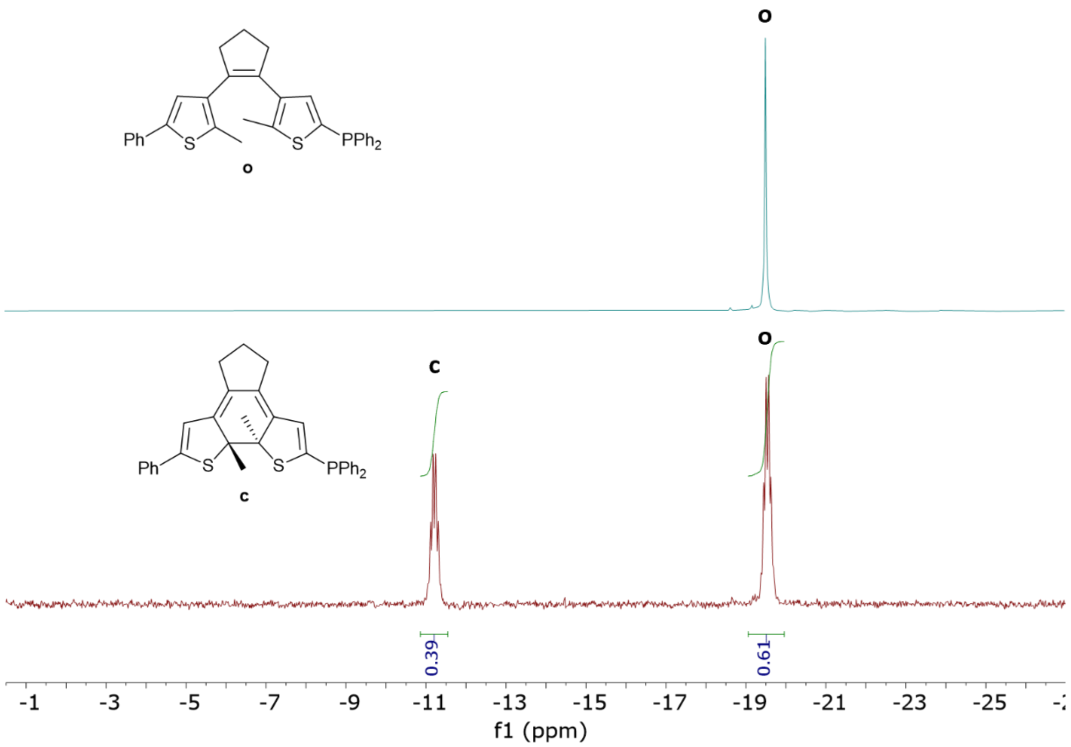

Figure S14. Changes in the $^{31}P\{^{1}H\}$ NMR spectrum (toluene-d_8) of **DTE-Ph** upon photocyclization: (top, 162 MHz) open state ligand (**o**); (bottom, 121 MHz) PSS@312 nm, where 39% open-to-closed conversion is determined from integrals.

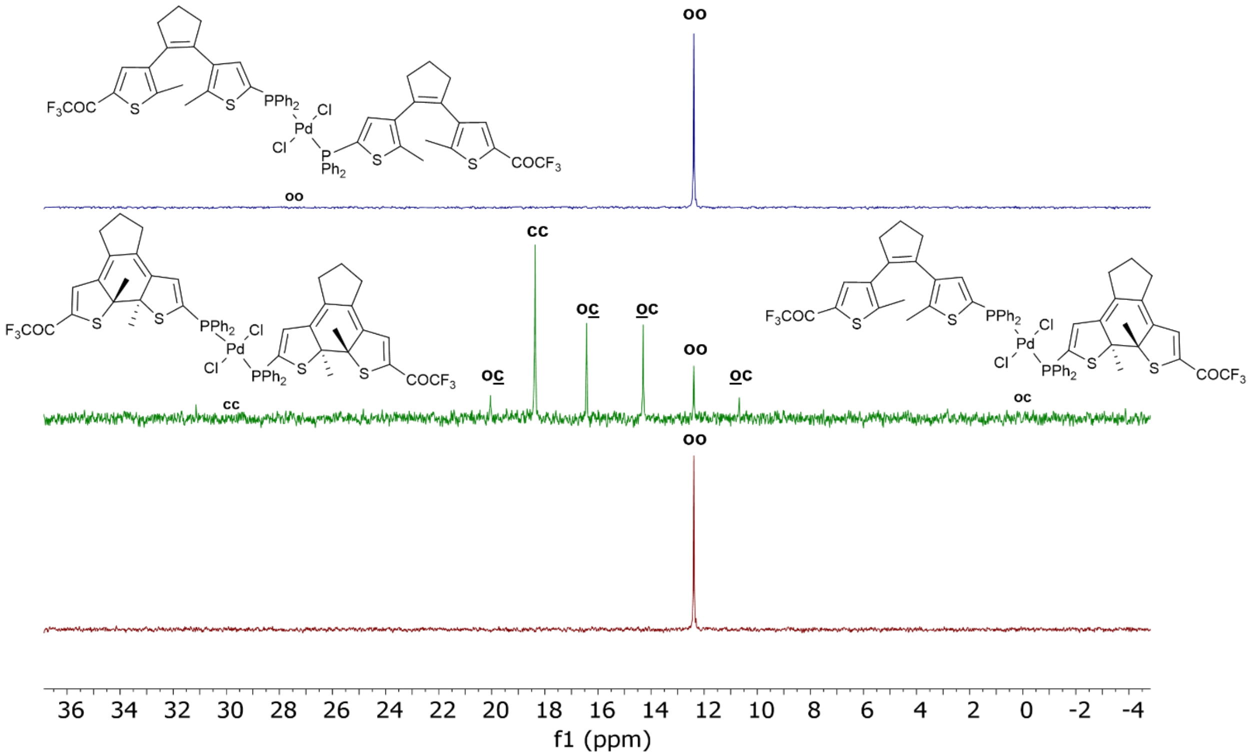

Figure S15. Changes in the $^{31}P\{^{1}H\}$ NMR spectrum (toluene-d_8, 162 MHz) of [$PdCl_2$(**DTE-COCF₃**)$_2$] upon photoswitching: (top) initial open state complex (**oo**); (middle) PSS@365 nm, where partial DTE photocyclization produces both **oc** ($^{2}J_{P,P}$ = 581 Hz) and **cc** complexes; (bottom) PSS@520 nm, where full back-photoisomerization to **oo** is observed. Labels **o̲c** and **oc̲** are used to identify the NMR signals of the

phosphorous nucleus attached to the ring-open and ring-closed units of the **oc** isomer, respectively. Only one signal was detected for the **cc** state, though it must comprise a mixture of three stereoisomers because of the four chiral centers created upon conrotatory photocyclization of their two DTE units: two enantiomers (RRRR and SSSS) and one diastereomeric *meso* form (RRSS).

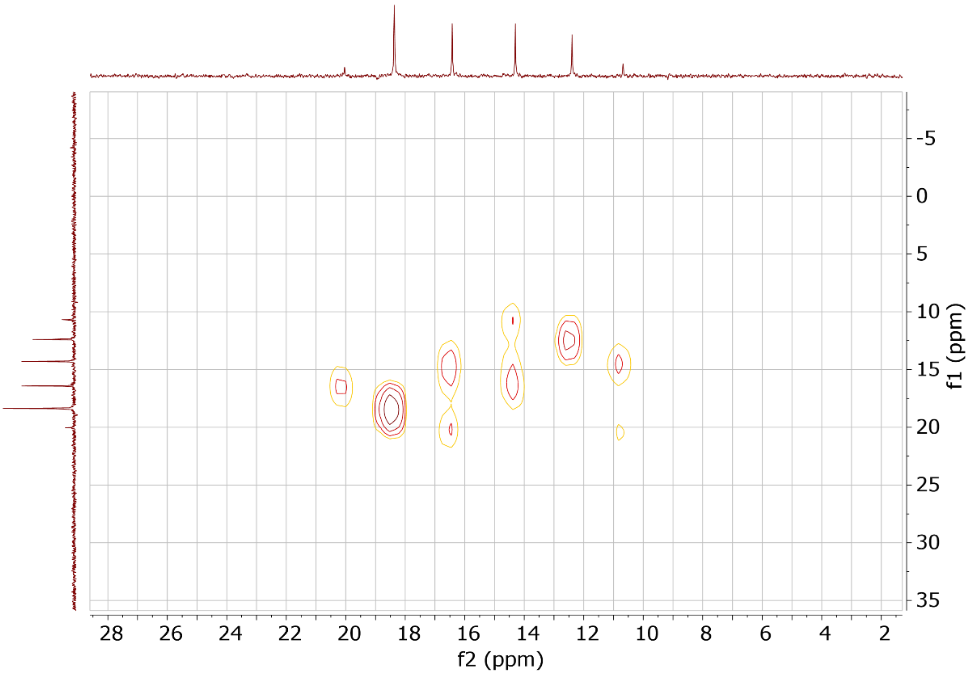

Figure S16. $^{31}P\{^{1}H\}$-$^{31}P\{^{1}H\}$ COSY spectrum (toluene-d_8, 162 MHz) of [$PdCl_2$(**DTE-COCF$_3$**)$_2$] at PSS@365 nm, where the cross-correlation between the two phosphorus nuclei in the **oc** isomer can be observed.

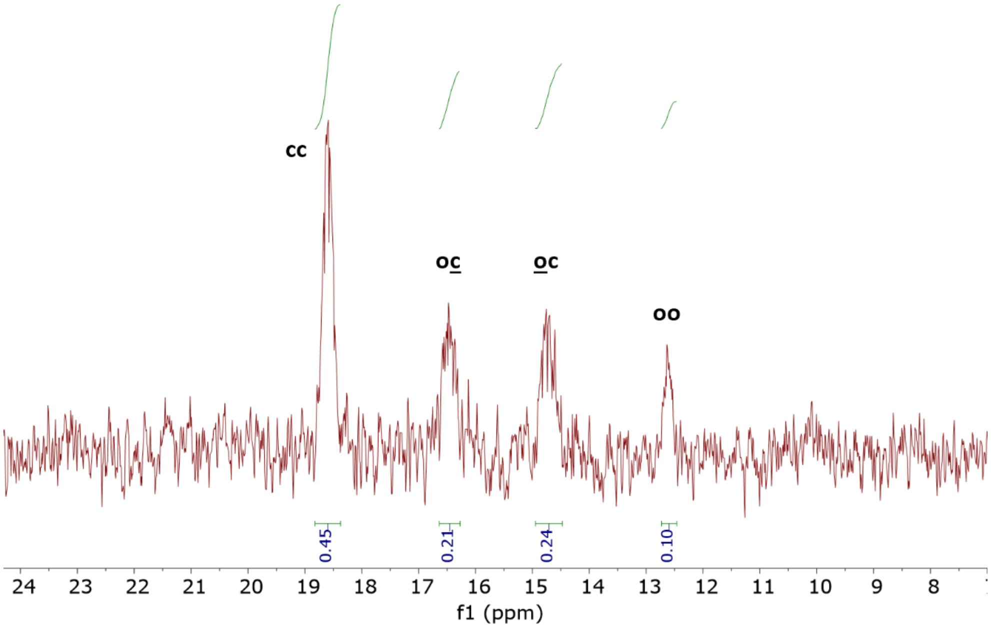

Figure S17. ^{31}P NMR spectrum (toluene-d_8, 162 MHz) of PSS@365 nm for [$PdCl_2$(**DTE-COCF$_3$**)$_2$], from which the composition of the photostationary state can be determined: 45:45:10 for **cc**:**oc**:**oo** complexes. Labels **oc** and **oc** are used to identify the NMR signals of the phosphorous nucleus attached to the ring-open and ring-closed units of the **oc** isomer, respectively.

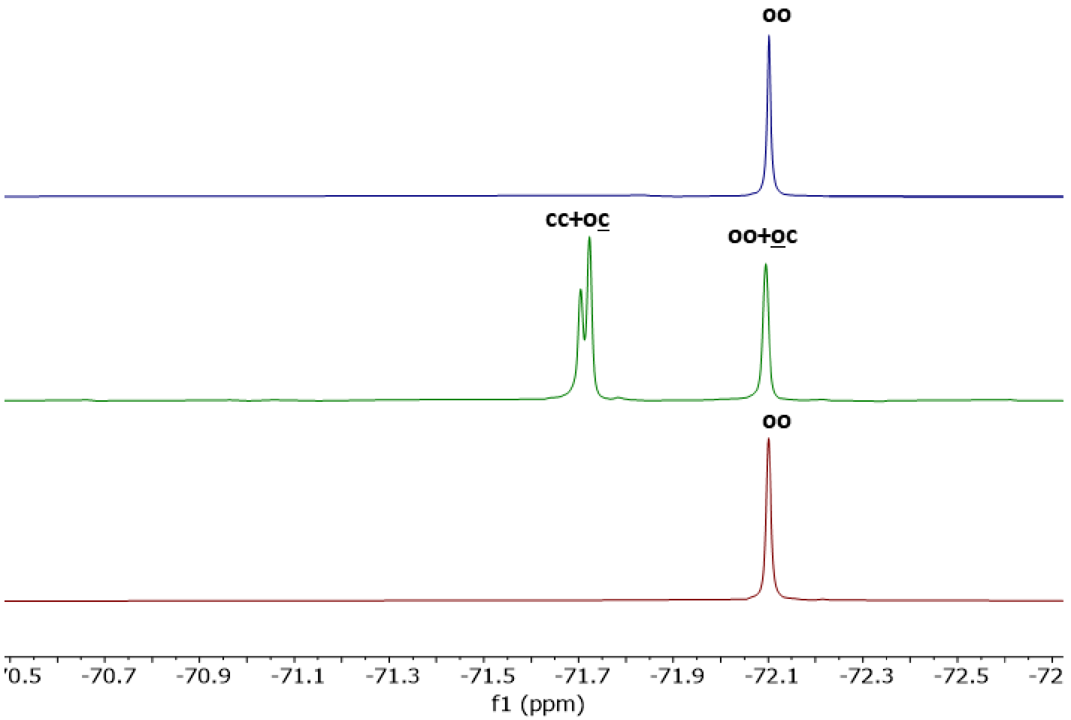

Figure S18. Changes in the ^{19}F NMR spectrum (toluene-d_8, 376 MHz) of [$PdCl_2$(**DTE-COCF$_3$**)$_2$] upon photoswitching: (top) initial open state complex (**oo**); (middle) PSS@365 nm; (bottom) PSS@520 nm, where full back-photoisomerization to **oo** is observed. Labels **oc** and **oc** are used to identify the NMR signals of the trifluoromethyl ketone group attached to the ring-open and ring-closed units of the **oc** isomer, respectively. Only one signal was detected for the **cc** state, though it must comprise a mixture of three stereoisomers because of the four chiral centers created upon conrotatory photocyclization of their two DTE units: two enantiomers (RRRR and SSSS) and one diastereomeric *meso* form (RRSS).

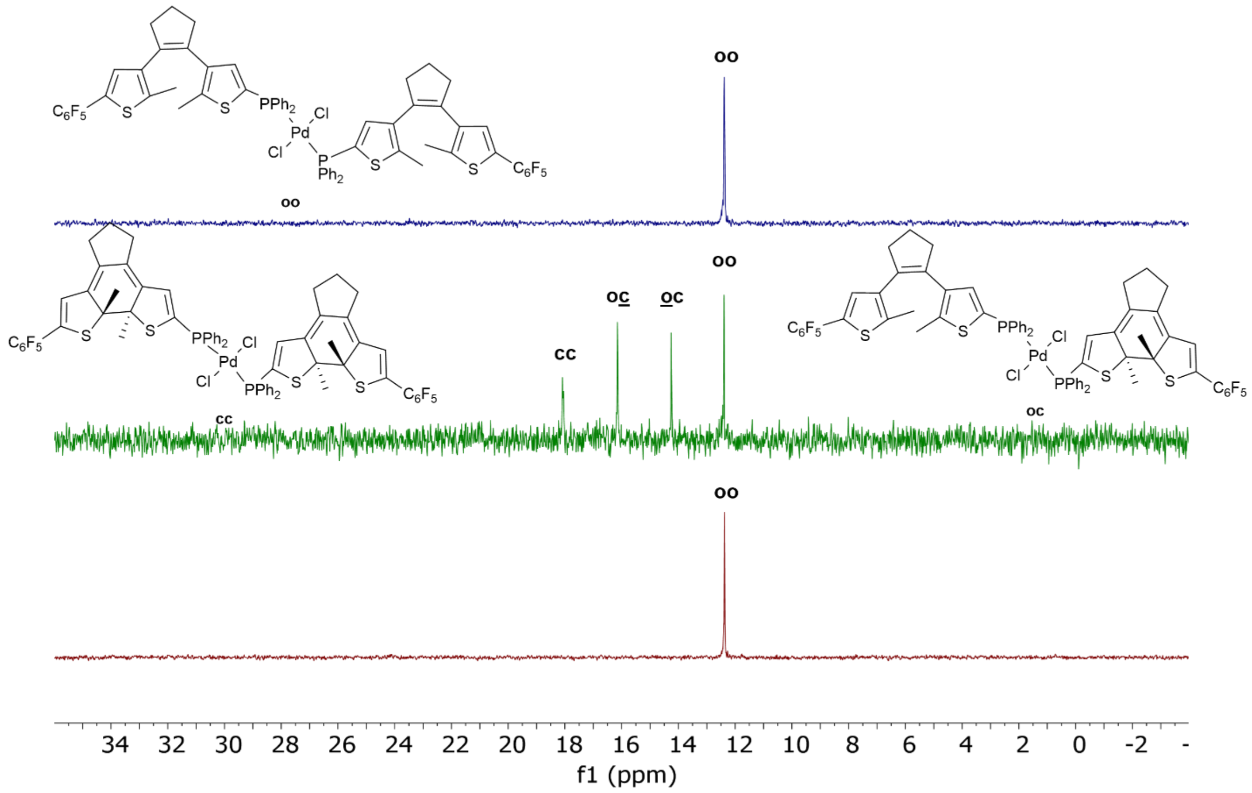

Figure S19. Changes in the $^{31}P\{^1H\}$ NMR spectrum (toluene-d_8, 121 MHz) of [PdCl$_2$(**DTE-C$_6$F$_5$**)$_2$] upon photoswitching: (top) initial open state complex (**oo**); (middle) PSS@365 nm, where partial DTE photocyclization produces both **oc** ($^2J_{P,P}$ not determined due to low signal-to-noise ratio) and **cc** complexes; (bottom) PSS@520 nm, where full back-photoisomerization to **oo** is observed. Labels **oc** and **oc** are used to identify the NMR signals of the phosphorous nucleus attached to the ring-open and ring-closed units of the **oc** isomer, respectively. Only one signal was detected for the **cc** state, though it must comprise a mixture of three stereoisomers because of the four chiral centers created upon conrotatory photocyclization of their two DTE units: two enantiomers (RRRR and SSSS) and one diastereomeric *meso* form (RRSS).

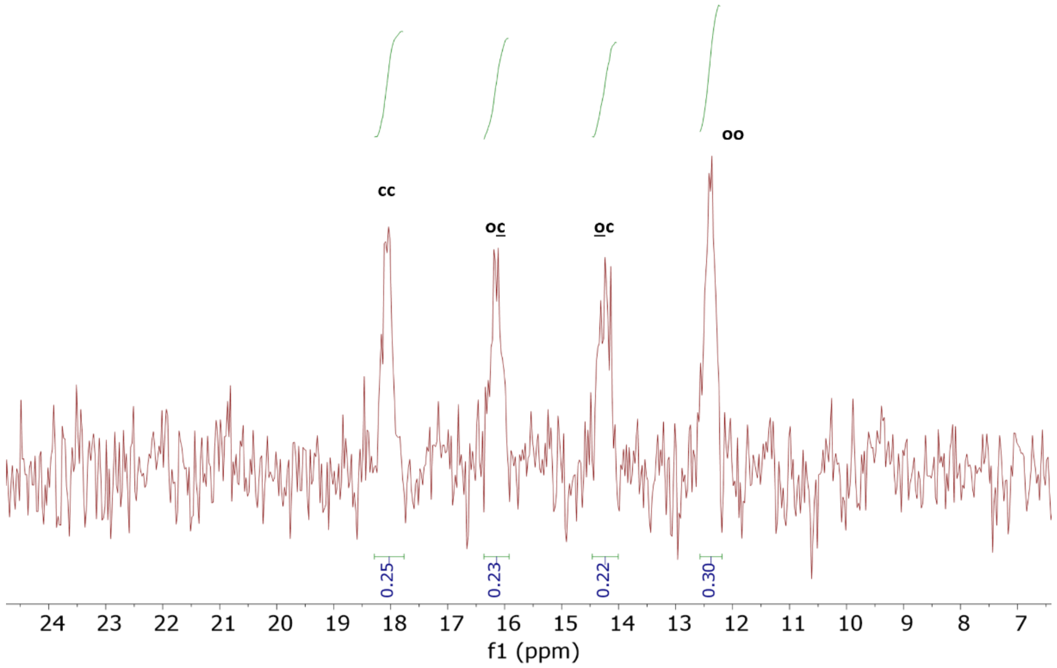

Figure S20. ^{31}P NMR spectrum (toluene-d_8, 121 MHz) of PSS@365 nm [PdCl$_2$(**DTE-C$_6$F$_5$**)$_2$]. From integrals, a 25:45:30 molar ratio can be estimated for the photoequilibrium **cc**:**oc**:**oo** mixture produced. Labels **oc** and **oc** are used to identify the NMR signals of the phosphorous nucleus attached to the ring-open and ring-closed units of the **oc** isomer, respectively.

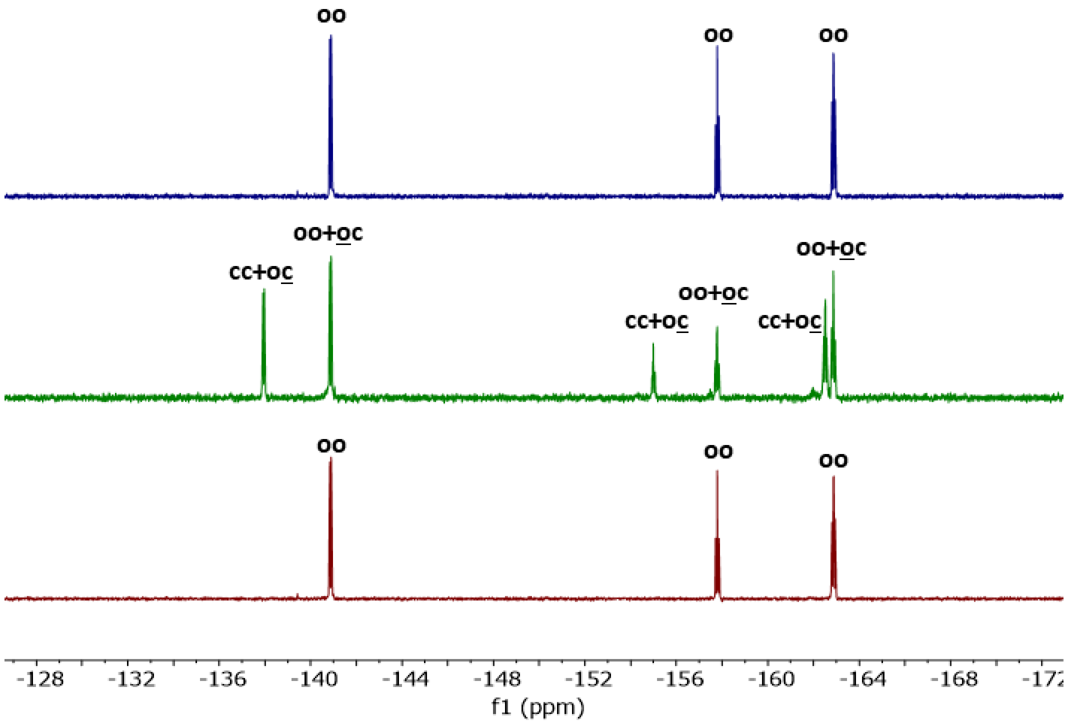

Figure S21. Changes in the ^{19}F NMR spectrum (toluene-d_8, 282 MHz) of $[PdCl_2(\mathbf{DTE\text{-}C_6F_5})_2]$ upon photoswitching: (top) initial open state complex (**oo**); (middle) PSS@365 nm, where partial DTE photocyclization produces both **oc** and **cc** complexes; (bottom) PSS@520 nm, where full back-photoisomerization to **oo** is observed. Labels $\underline{\mathbf{o}}\mathbf{c}$ and $\mathbf{o}\underline{\mathbf{c}}$ are used to identify the NMR signals of the pentafluorophenyl group attached to the ring-open and ring-closed units of the **oc** isomer, respectively. Only one signal was detected for the **cc** state, though it must comprise a mixture of three stereoisomers because of the four chiral centers created upon conrotatory photocyclization of their two DTE units: two enantiomers (RRRR and SSSS) and one diastereomeric *meso* form (RRSS).

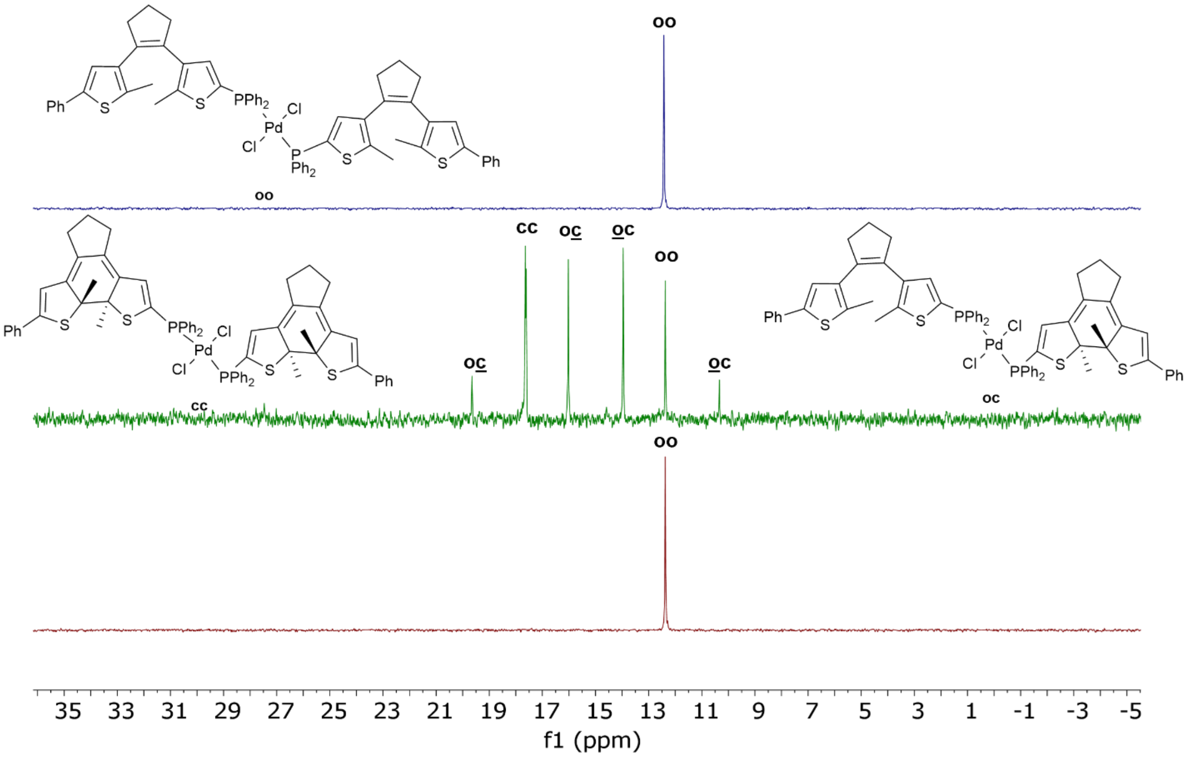

Figure S22. Changes in the ${}^{31}P\{{}^{1}H\}$ NMR spectrum (toluene-d_8, 162 MHz) of [$PdCl_2$(**DTE-Ph**)$_2$] upon photoswitching: (top) initial open state complex (**oo**); (middle) PSS@365 nm, where partial DTE photocyclization produces both **oc** (${}^{2}J_{P,P}$ = 586 Hz) and **cc** complexes; (bottom) PSS@520 nm, where full back-photoisomerization to **oo** is observed. Labels **oc** and **oc** are used to identify the NMR signals of the phosphorous nucleus attached to the ring-open and ring-closed units of the **oc** isomer, respectively. Only one signal was detected for the **cc** state, though it must comprise a mixture of three stereoisomers because of the four chiral centers created upon conrotatory photocyclization of their two DTE units: two enantiomers (RRRR and SSSS) and one diastereomeric *meso* form (RRSS).

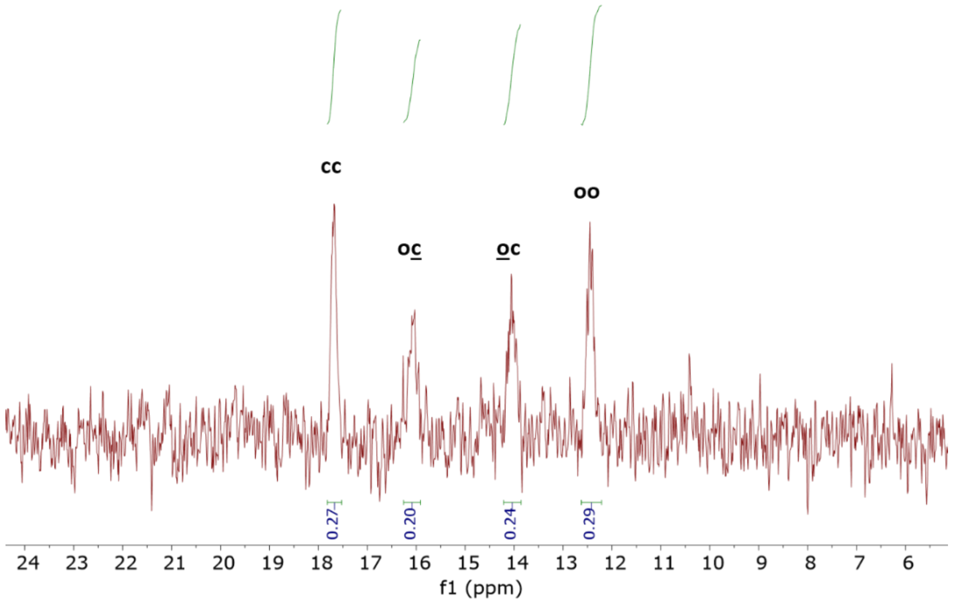

Figure S23. ${}^{31}P$ NMR spectrum (toluene-d_8, 162 MHz) of PSS@365 nm for [$PdCl_2$(**DTE-Ph**)$_2$]. From integrals, a 27:44:29 molar ratio can be estimated for the photoequilibrium **cc**:**oc**:**oo** mixture produced. Labels **oc** and **oc** are used to identify the NMR signals of the phosphorous nucleus attached to the ring-open and ring-closed units of the **oc** isomer, respectively.

3 DFT calculations of the optical properties of ligands and complexes

Optimized ligand structures in the ground state show characteristic geometry changes accompanying the isomerization process. For instance, the distance between the reactive carbon atoms changes from 3.49 Å in the open state to 1.54 Å in the closed state, in accordance with previously reported data.[7] However, no significant change in bond lengths or dihedral angles associated with different substituents in one of the thiophene rings was found.

3.1 Frontier molecular orbitals analysis

Table S2. Variation of the HOMO-LUMO gap in ligands and their palladium complexes.

State	Parameter	**DTE-COCF$_3$**	**DTE-C$_6$F$_5$**	**DTE-Ph**
**o**	HOMO	-5.63	-5.42	-5.28
	LUMO	-2.15	-1.34	-0.96
	ΔE	**3.48**	**4.09**	**4.32**
**c**	HOMO	-4.94	-4.69	-4.57
	LUMO	-2.72	-2.09	-1.93
	ΔE	**2.22**	**2.60**	**2.64**
		[PdCl$_2$(**DTE-COCF$_3$**)$_2$]	[PdCl$_2$(**DTE-C$_6$F$_5$**)$_2$]	[PdCl$_2$(**DTE-Ph**)$_2$]
**oo**	HOMO	-5.71	-5.43	-5.29
	LUMO	-2.32	-2.21	-2.20
	ΔE	**3.39**	**3.22**	**3.09**
**oc**	HOMO	-5.03	-4.72	-4.61
	LUMO	-2.80	-2.29	-2.29
	ΔE	**2.23**	**2.43**	**2.32**
**cc**	HOMO	-5.01	-4.74	-4.61
	LUMO	-2.80	-2.36	-2.31
	ΔE	**2.21**	**2.38**	**2.31**

Table S3. Frontier molecular orbitals of the open and closed forms of the ligands.

	HOMO	LUMO	LUMO+1
**DTE^{o}-COCF$_3$**	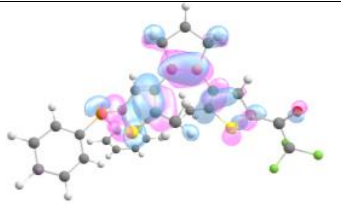	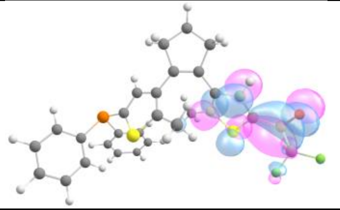	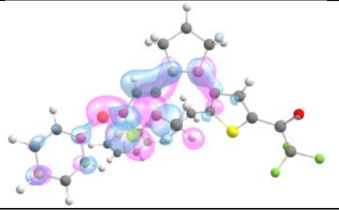
**DTE^{c}-COCF$_3$**	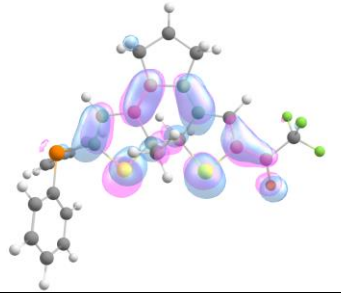	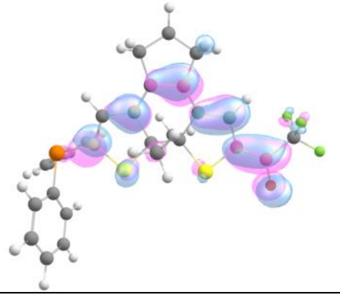	

**DTE^{o}-C$_6$F$_5$**	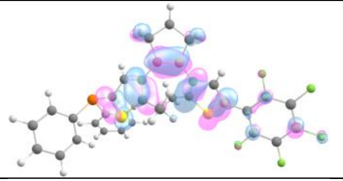	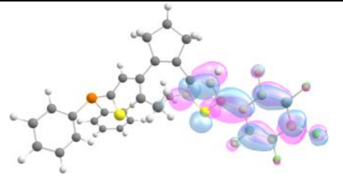	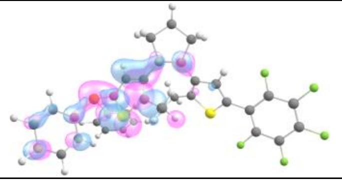
**DTE^{c}-C$_6$F$_5$**	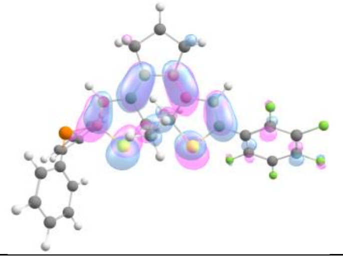	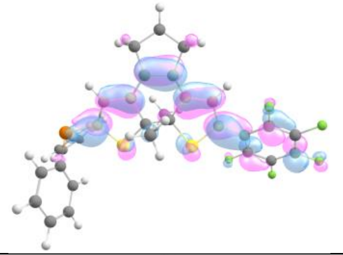	
**DTE^{o}-Ph**	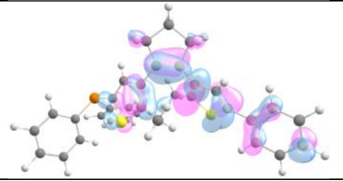	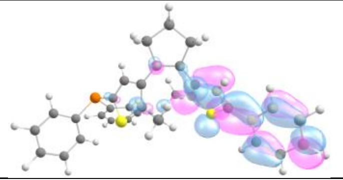	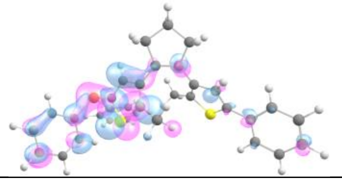
**DTE^{c}-Ph**	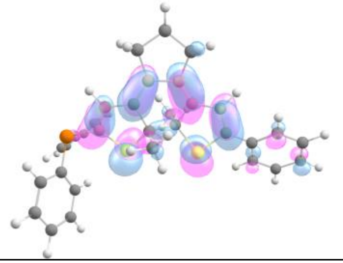	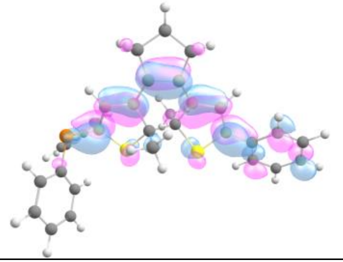	

Table S4. Selected frontier molecular orbitals of the **oo**, **oc** and **cc** forms of the complexes.

[PdCl$_2$(**DTE^{o}-COCF$_3$**)$_2$]

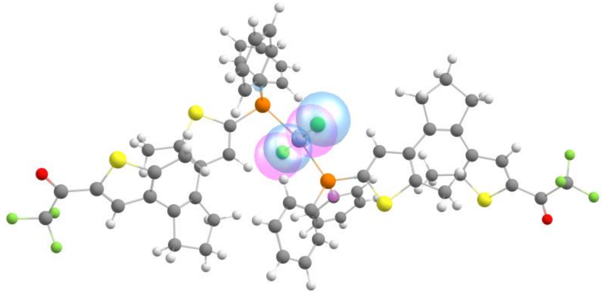

HOMO-26

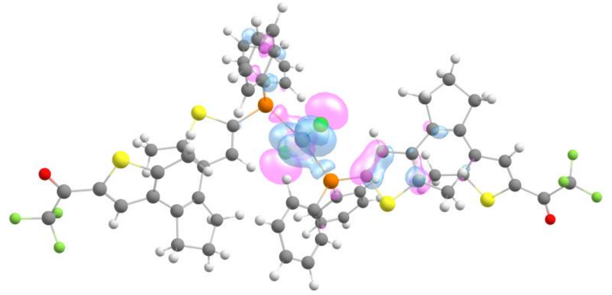

HOMO-3

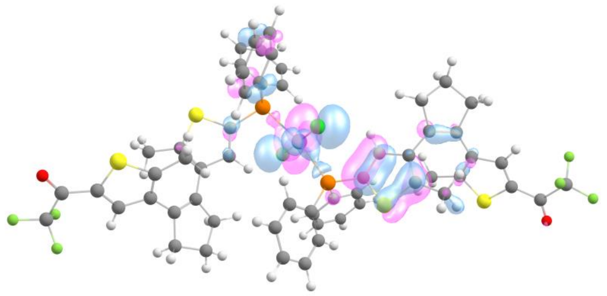

HOMO-2

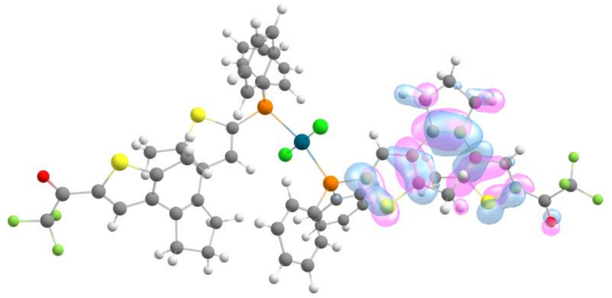

HOMO

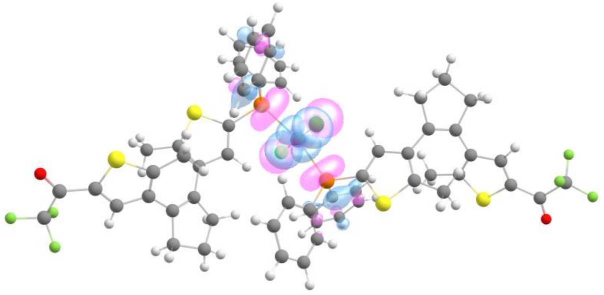

LUMO

[PdCl$_2$(**DTE^{o}-COCF$_3$**)(**DTE^{c}-COCF$_3$**)]

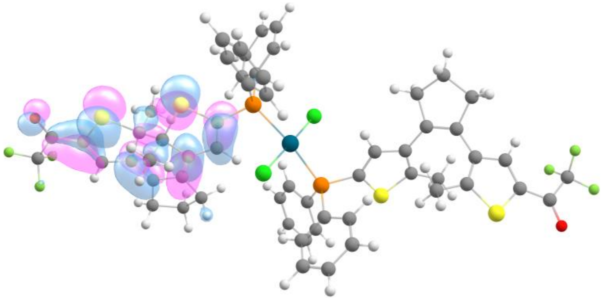

HOMO

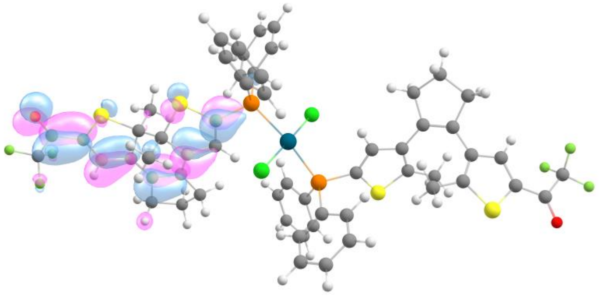

LUMO

[PdCl$_2$(**DTE^{c}-COCF$_3$**)$_2$]

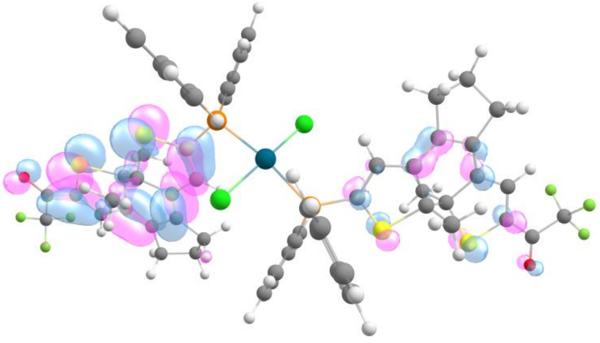

HOMO-1

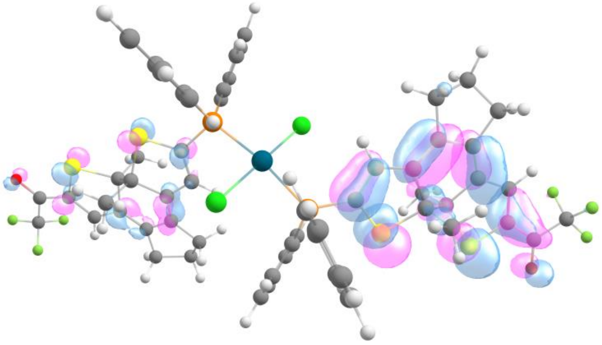

HOMO

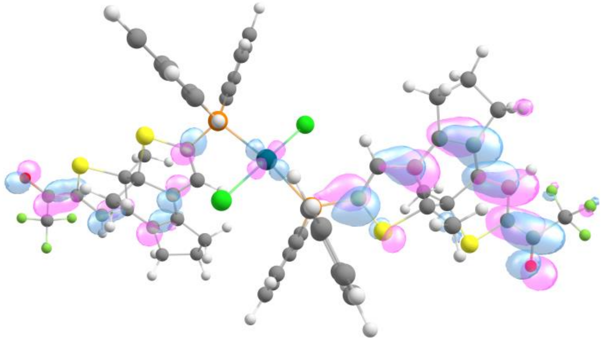

LUMO

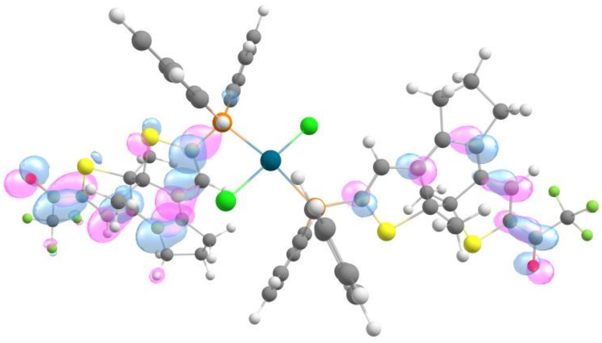

LUMO+1

[$PdCl_2$(**DTE^{o}-C_6F_5**)$_2$]

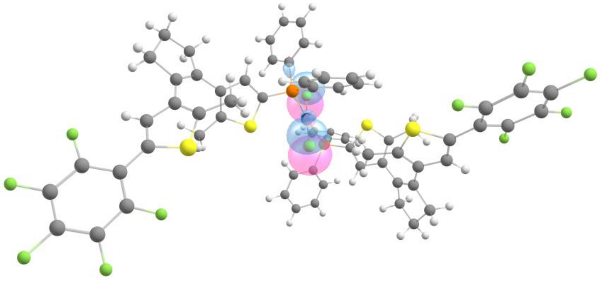

HOMO-28

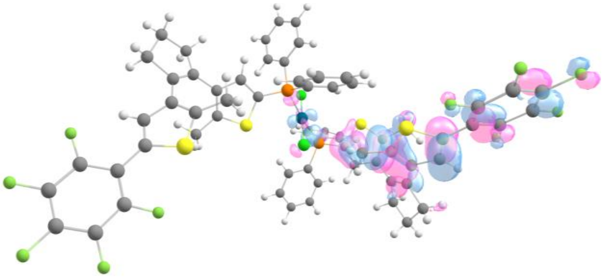

HOMO-2

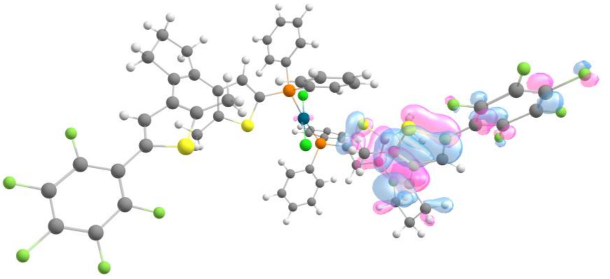

HOMO

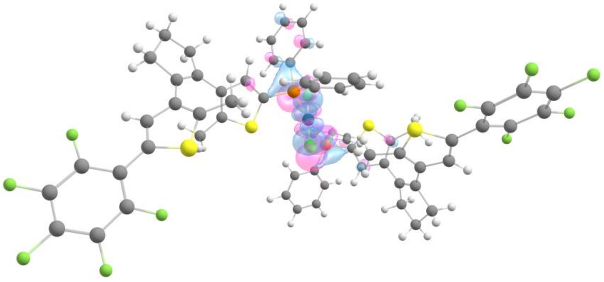

LUMO

[$PdCl_2$(**DTE^{o}-C_6F_5**)(**DTE^{c}-C_6F_5**)]

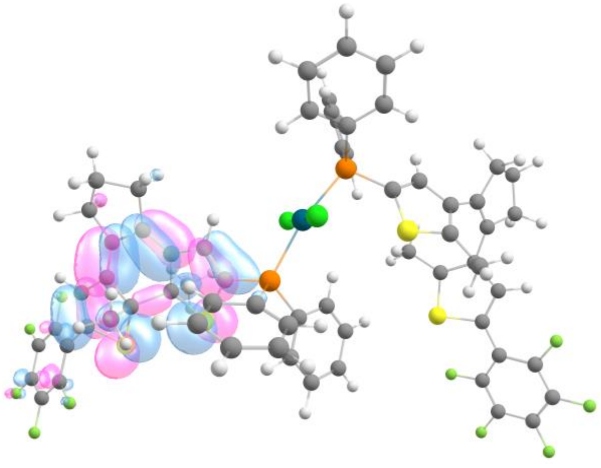

HOMO

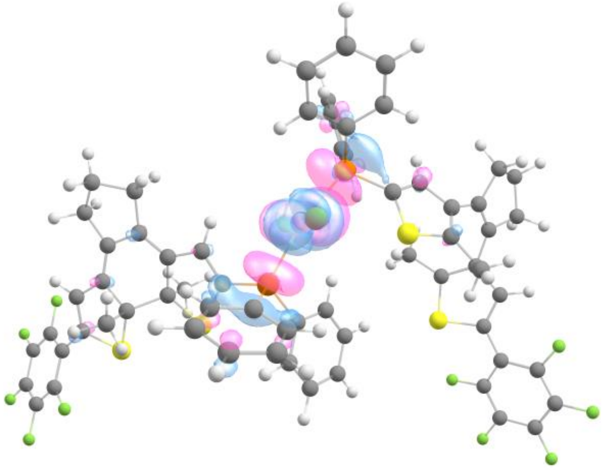

LUMO

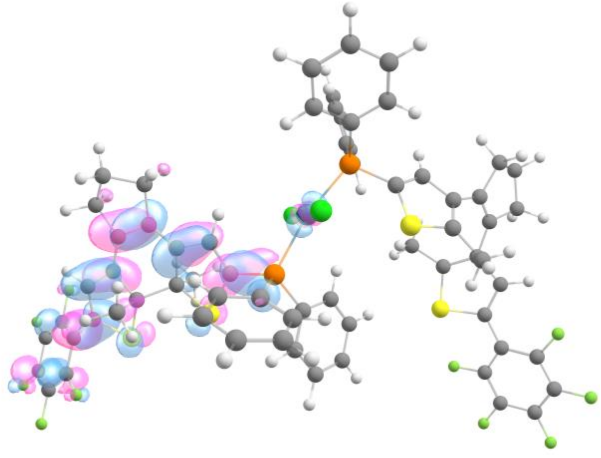

LUMO+1

[PdCl$_2$(**DTE^c-C$_6$F$_5$**)$_2$]

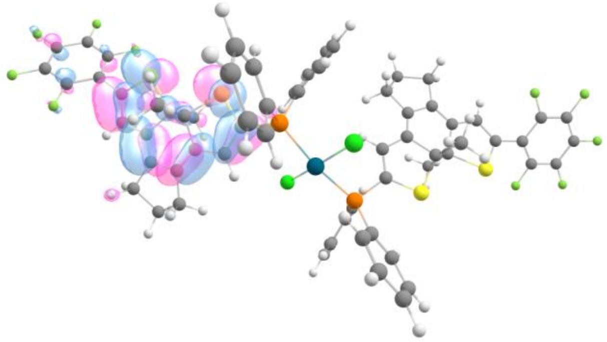

HOMO-1

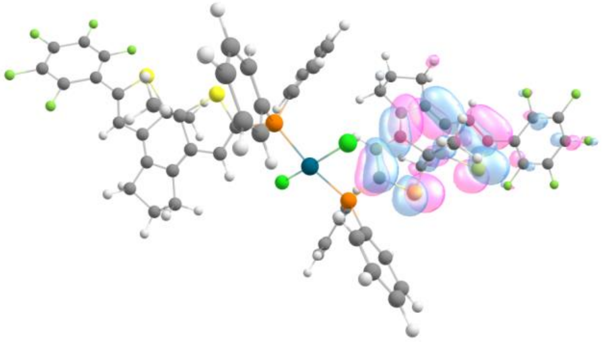

HOMO

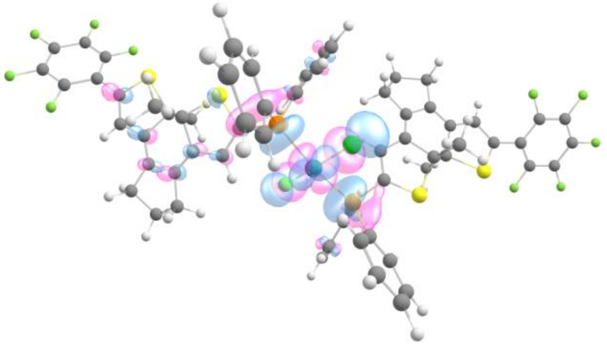

LUMO

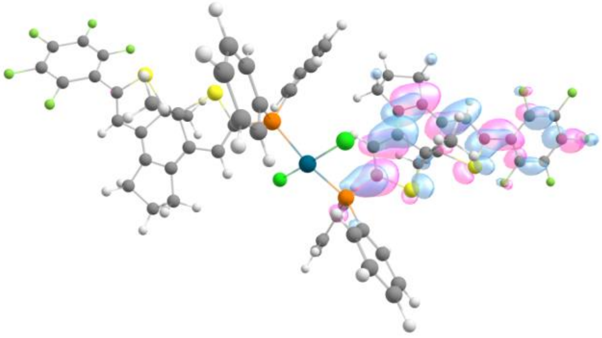

LUMO+1

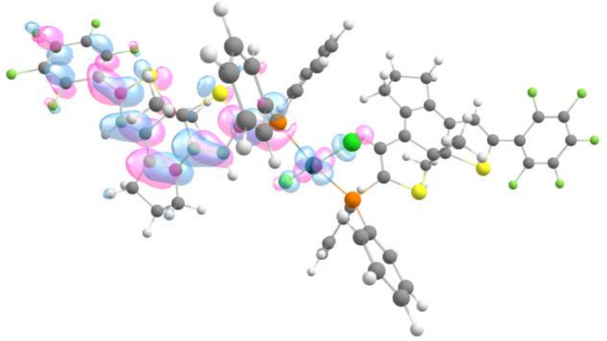

LUMO+2

[PdCl$_2$(**DTE^o-Ph**)$_2$]

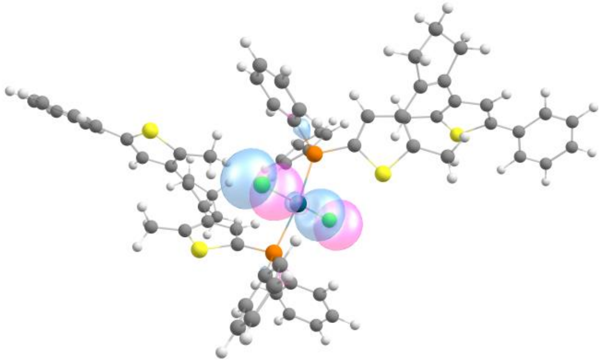

HOMO-28

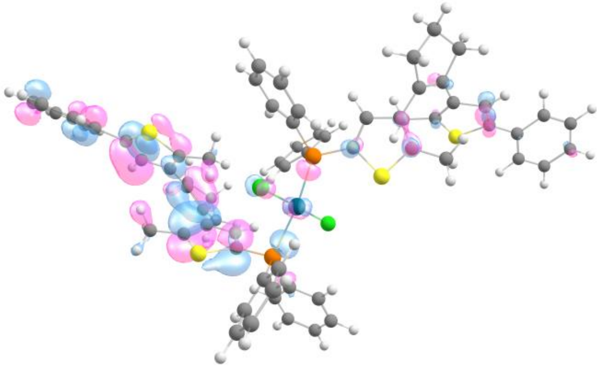

HOMO-2

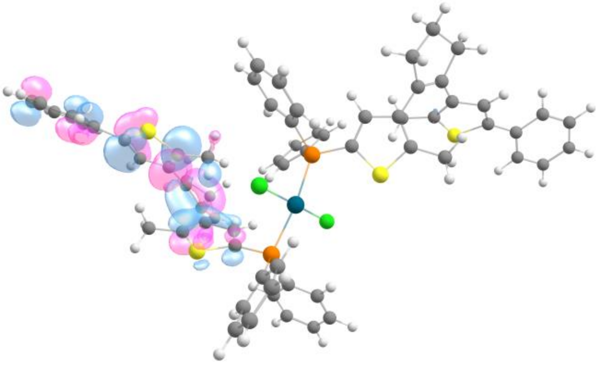

HOMO

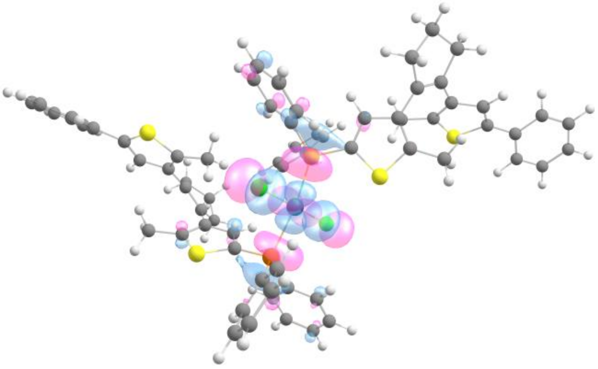

LUMO

[$PdCl_2$(**DTE^{o}-Ph**)(**DTE^{c}-Ph**)]

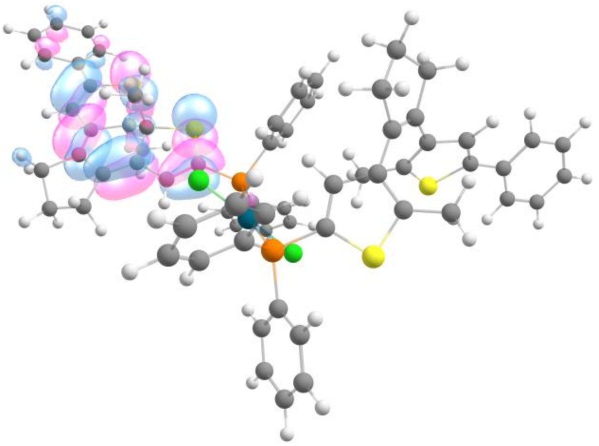

HOMO

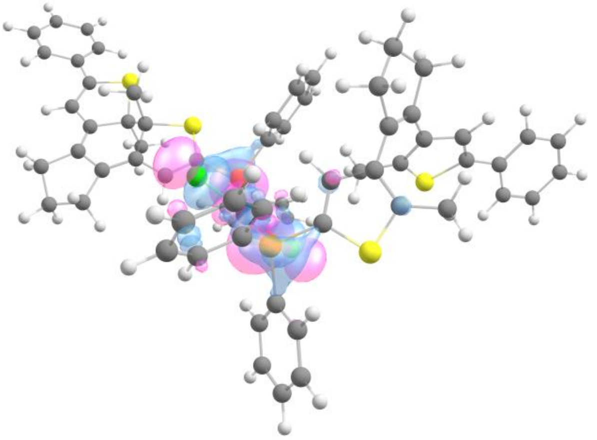

LUMO

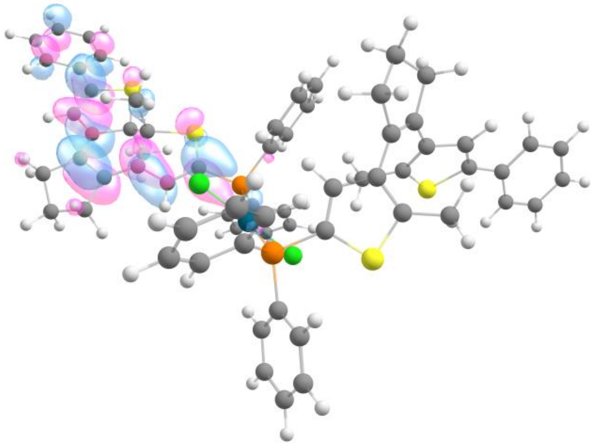

LUMO+1

[$PdCl_2$(**DTE^{c}-Ph**)$_2$]

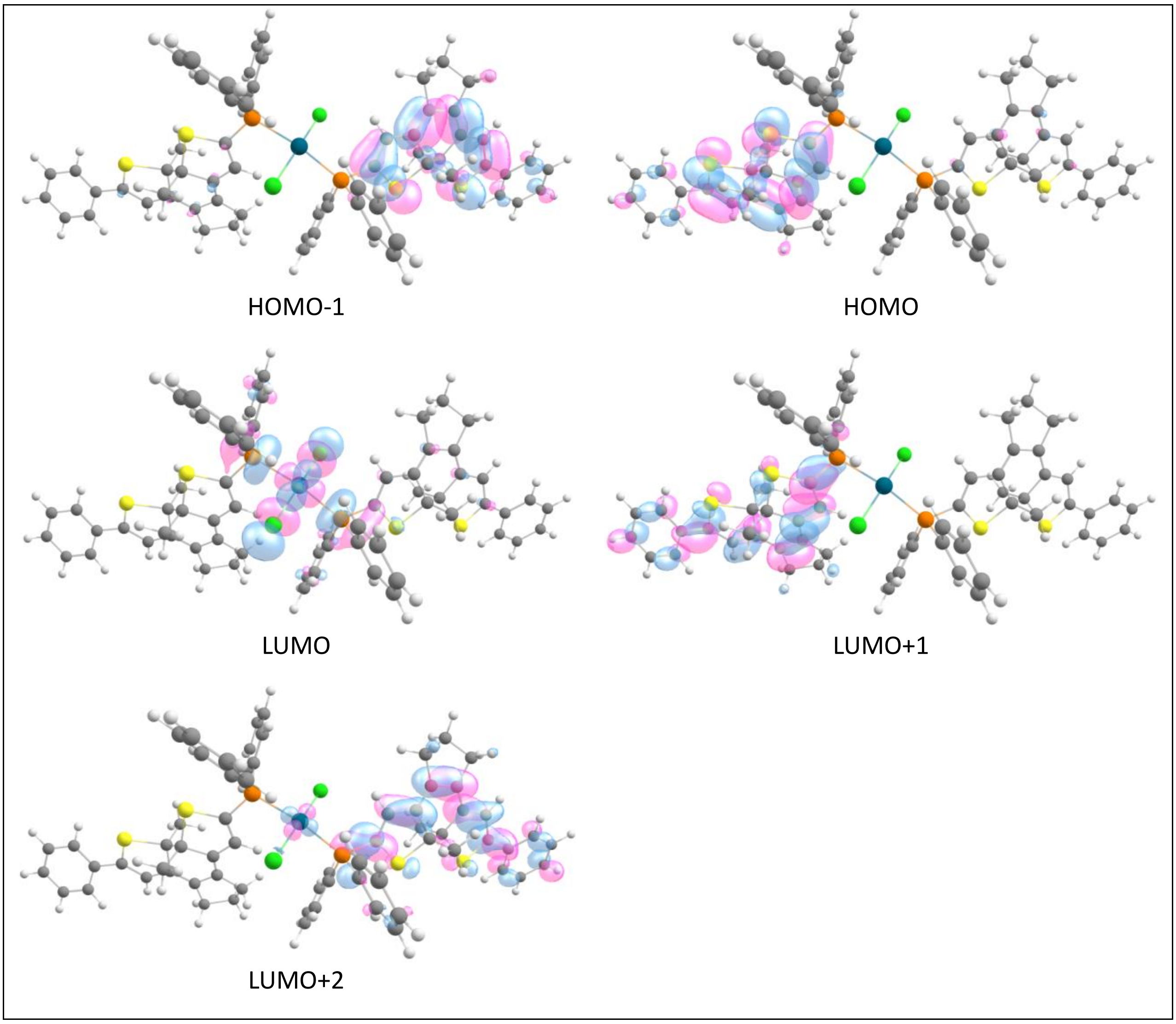

3.2 Vertical transitions

Table S5. Calculated absorption wavelengths (λ), oscillator strengths (*f*) and assigned transitions for singlet state ligands and complexes in toluene within the 250-800 nm range.

	λ (nm)	*f*	Assigned transitions
**DTE^{o}-COCF$_3$**	319	0.1265	H – L (78.7%)
	272	0.4535	H-4 – L (24.8%); H-3 – L (23.3%); H-2 – L (22.7%); H-5 – L (6.6%); H – L+1 (5.7%)
**DTE^{c}-COCF$_3$**	576	0.3497	H – L (96.5%)
**DTE^{o}-C$_6$F$_5$**	292	0.4847	H – L (76.8%)
	268	0.3325	H – L+1 (61.4%)
**DTE^{c}-C$_6$F$_5$**	500	0.4337	H – L (95.9%)
**DTE^{o}-Ph**	284	0.4609	H – L (84.5%)
	270	0.3146	H – L+1 (54.7%)
**DTE^{c}-Ph**	495	0.4667	H – L (96.5%)
[PdCl$_2$(**DTE^{o}-COCF$_3$**)$_2$]	350	0.2612	H-26 – L (33.4%); H-3 – L (22.7%); H-2 – L (19.9%)
[PdCl$_2$(**DTE^{o}-COCF$_3$**)(**DTE^{c}-COCF$_3$**)]	573	0.4026	H – L (96.6%)
[PdCl$_2$(**DTE^{c}-COCF$_3$**)$_2$]	575	0.6814	H-1 – L+1 (31.5%); H – L (27.8%); H – L+1 (27.0%); H-1 – L (9.9%)
	568	0.1539	H-1 – L (53.9%); H – L+1 (37.2%)
[PdCl$_2$(**DTE^{o}-C$_6$F$_5$**)$_2$]	335	0.2618	H-28 – L (34.2%)
	324	0.2353	H-28 – L (37.1%); H-2 – L (20.5%)
[PdCl$_2$(**DTE^{o}-C$_6$F$_5$**)(**DTE^{c}-C$_6$F$_5$**)]	506	0.5280	H – L (70.4%); H – L+1 (25.4%)
[PdCl$_2$(**DTE^{c}-C$_6$F$_5$**)$_2$]	508	0.8420	H – L+1 (61.8%)
	502	0.1962	H-1 – L (44.0%); H – L+1 (26.1%); H-1 – L+2 (23.2%); H-1 – L+1 (2.6%)
[PdCl$_2$(**DTE^{o}-Ph**)$_2$]	348	0.2910	H-28 – L (27.5%)
	324	0.2141	H-28 – L (37.4%); H-2 – L (19.8%)
[PdCl$_2$(**DTE^{o}-Ph**)(**DTE^{c}-Ph**)]	503	0.5810	H – L+1 (87.6%)
[PdCl$_2$(**DTE^{c}-Ph**)$_2$]	506	0.9577	H – L+1 (50.9%); H-1 – L+1 (22.6%)
	500	0.1759	H – L+1 (40.0%); H-1 – L+2 (35.3%); H-1 – L (20.2%)

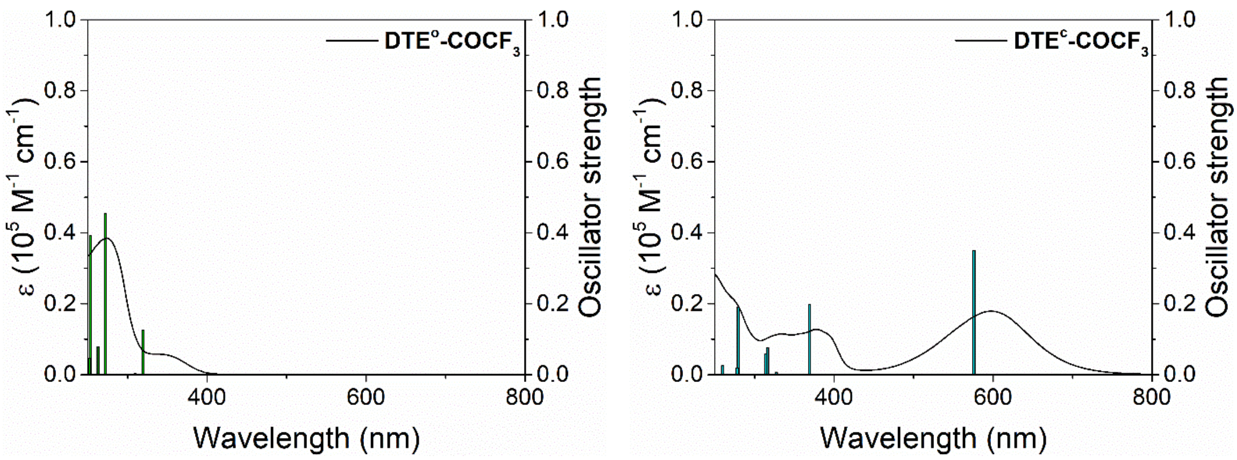

Figure S24. Experimental absorption spectra (shown as lines) and vertical transitions computed (shown as bars) for **DTE-COCF$_3$** in toluene: (left) **DTE°-COCF$_3$**; (right) **DTE^c-COCF$_3$**.

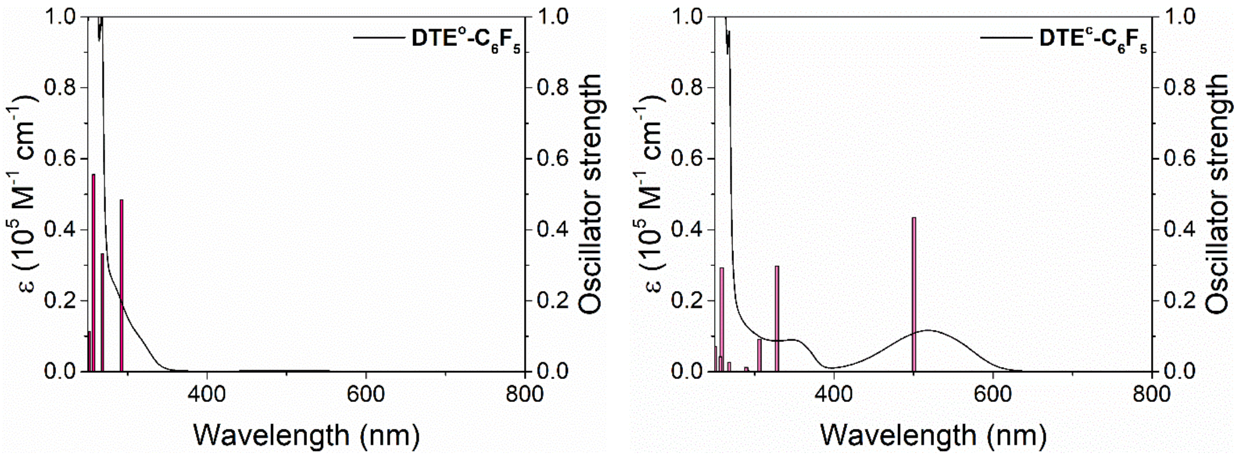

Figure S25. Experimental absorption spectra (shown as lines) and vertical transitions computed (shown as bars) for **DTE-C$_6$F$_5$** in toluene: (left) **DTE°-C$_6$F$_5$**; (right) **DTE^c-C$_6$F$_5$**.

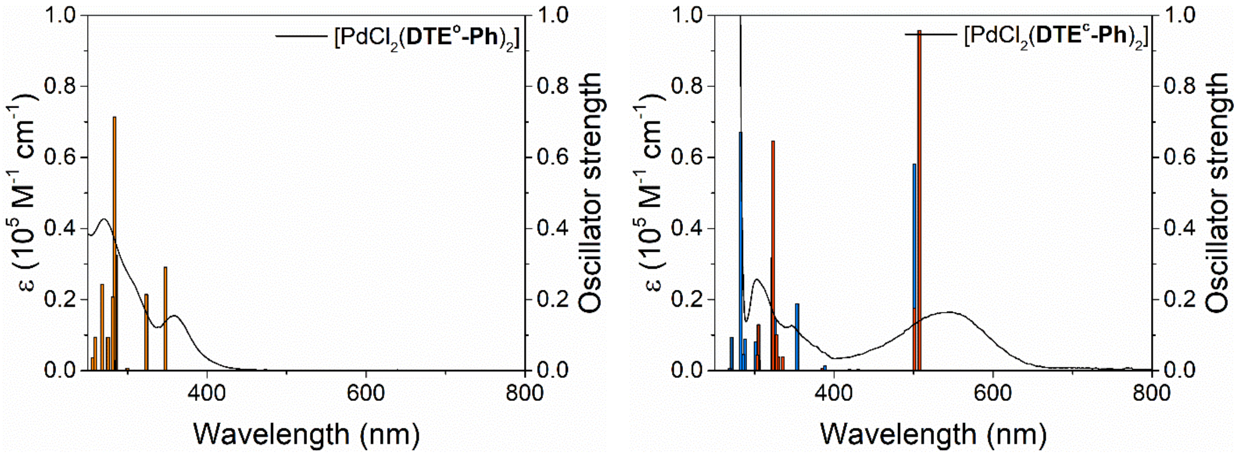

Figure S26. Experimental absorption spectra (shown as lines) and vertical transitions computed (shown as bars) for **DTE-Ph** in toluene: (left) **DTE°-Ph**; (right) **DTE^c-Ph**.

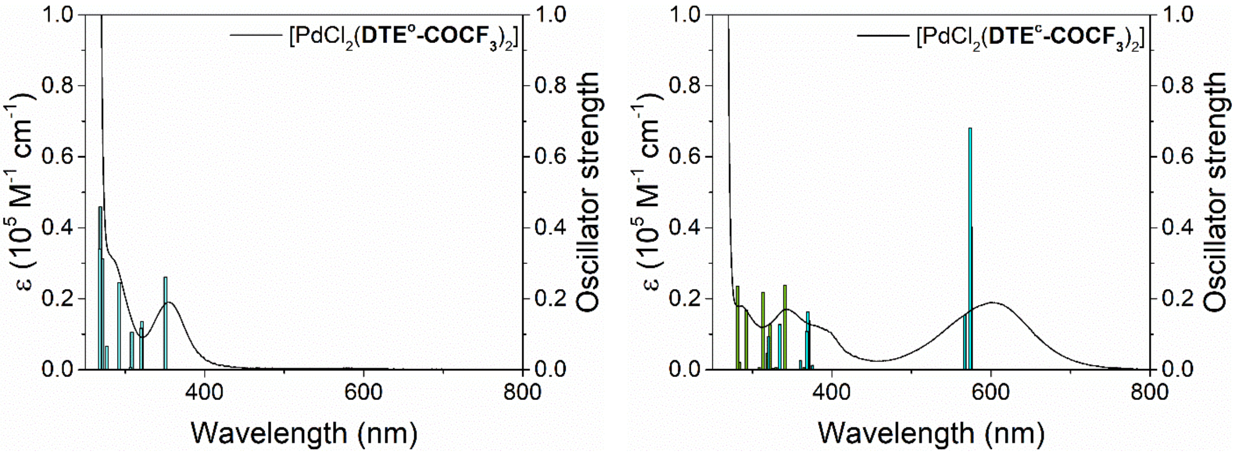

Figure S27. Experimental absorption spectra (shown as lines) and vertical transitions computed (shown as bars) for [$PdCl_2$(**DTE-COCF$_3$**)$_2$] in toluene: (left) [$PdCl_2$(**DTE^o-COCF$_3$**)$_2$]; (right) [$PdCl_2$(**DTE^c-COCF$_3$**)$_2$], for which vertical transitions are shown for both **oc** (green) and **cc** (cyan) isomers.

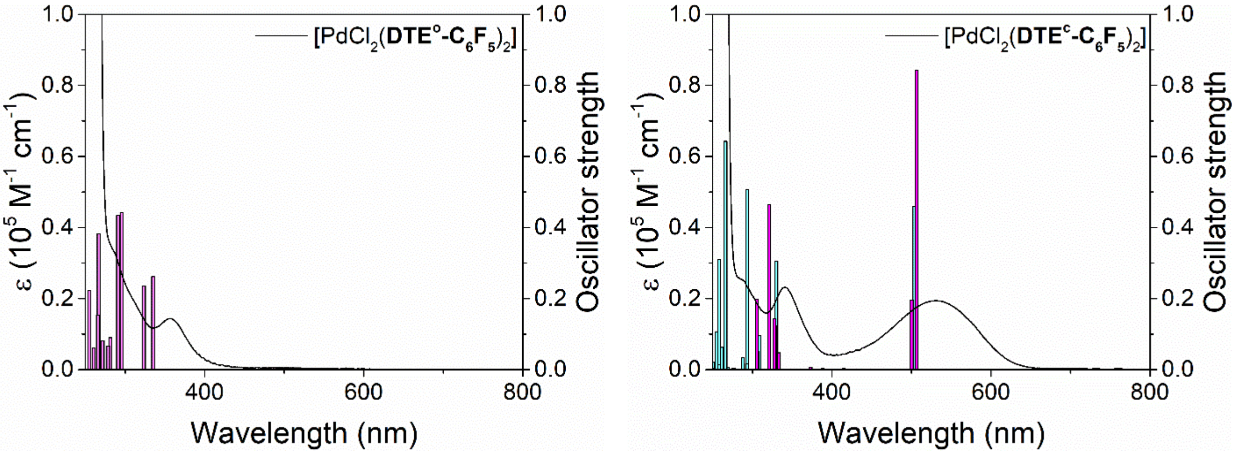

Figure S28. Experimental absorption spectra (shown as lines) and vertical transitions computed (shown as bars) for [$PdCl_2$(**DTE-C$_6$F$_5$**)$_2$] in toluene: (left) [$PdCl_2$(**DTE^o-C$_6$F$_5$**)$_2$]; (right) [$PdCl_2$(**DTE^c-C$_6$F$_5$**)$_2$], for which vertical transitions are shown for both **oc** (light cyan) and **cc** (magenta) isomers.

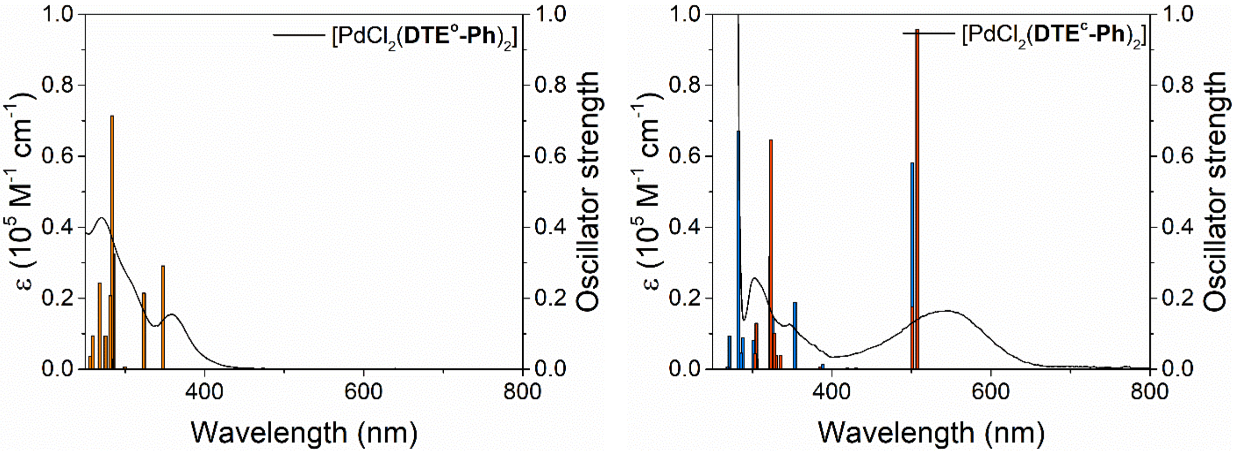

Figure S29. Experimental absorption spectra (shown as lines) and vertical transitions computed (shown as bars) for [$PdCl_2$(**DTE-Ph**)$_2$] in toluene: (left) [$PdCl_2$(**DTE^o-Ph**)$_2$]; (right) [$PdCl_2$(**DTE^c-Ph**)$_2$], for which vertical transitions are shown for both **oc** (blue) and **cc** (red) isomers.

4 Catalytic studies

4.1 NMR characterization of the **cc**-enriched state of the palladium complexes tested

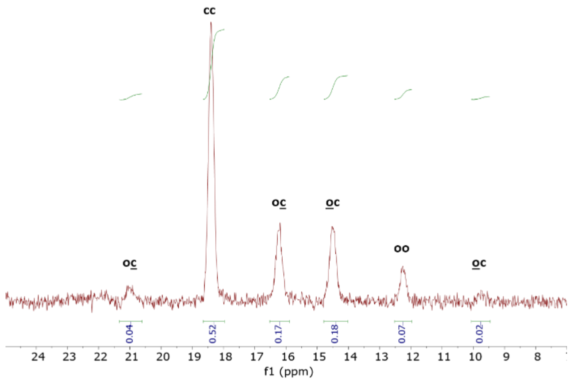

Figure S30. ^{31}P NMR spectrum ($CDCl_3$, 121 MHz) of the closed state-enriched [$PdCl_2$(**DTE-COCF$_3$**)$_2$] tested in the catalytic reaction. From integrals, a 52:41:7 molar ratio can be assigned to the **cc**, **oc** and **oo** isomers of the complex in this mixture, respectively. Labels **oc** and **oc** are used to identify the NMR signals of the phosphorous nucleus attached to the ring-open and ring-closed units of the **oc** isomer, respectively.

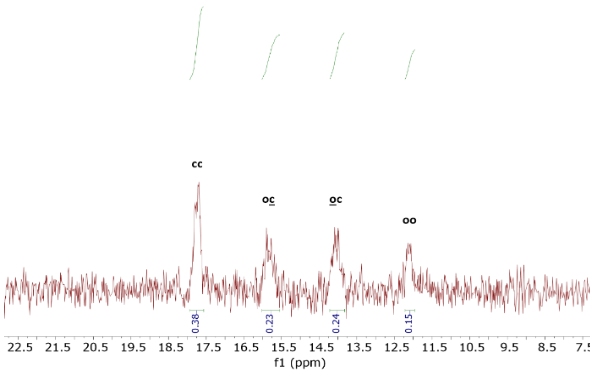

Figure S31. ^{31}P NMR spectrum ($CDCl_3$, 121 MHz) of the closed state-enriched [$PdCl_2$(**DTE-C$_6$F$_5$**)$_2$] tested in the catalytic reaction. From integrals, a 38:47:15 molar ratio can be assigned to the **cc**, **oc** and **oo** isomers of the complex in this mixture, respectively. Labels **oc** and **oc** are used to identify the NMR signals of the phosphorous nucleus attached to the ring-open and ring-closed units of the **oc** isomer, respectively.

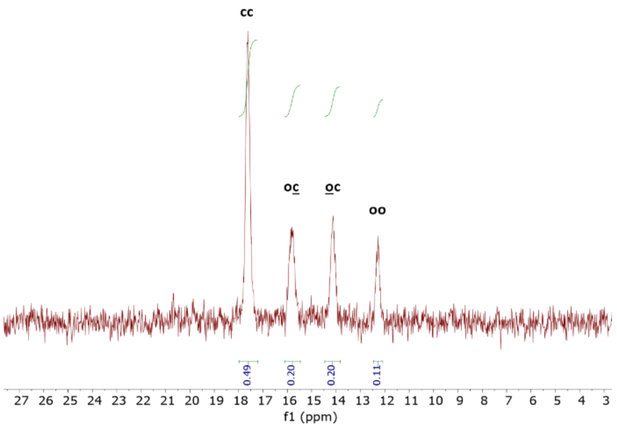

Figure S32. ^{31}P NMR spectrum ($CDCl_3$, 121 MHz) of the closed state-enriched [$PdCl_2$(**DTE-Ph**)$_2$] tested in the catalytic reaction. From integrals, a 49:40:11 molar ratio can be assigned to the **cc**, **oc** and **oo** isomers of the complex in this mixture, respectively. Labels **oc** and **oc** are used to identify the NMR signals of the phosphorous nucleus attached to the ring-open and ring-closed units of the **oc** isomer, respectively.

4.2 Thermal stability of [$PdCl_2$(**DTE-COCF₃**)$_2$]

The thermal stability of [$PdCl_2$(**DTE-$COCF_3$**)$_2$] in its open and closed state-enriched forms was measured for a stock solution of the complex (0.045 mmol) in THF-d_8 at 50 °C for 6 h. Heteronuclear NMR experiments were used to confirm the absence of decomposition products and thermally-induced ring-opening of the closed DTE units.

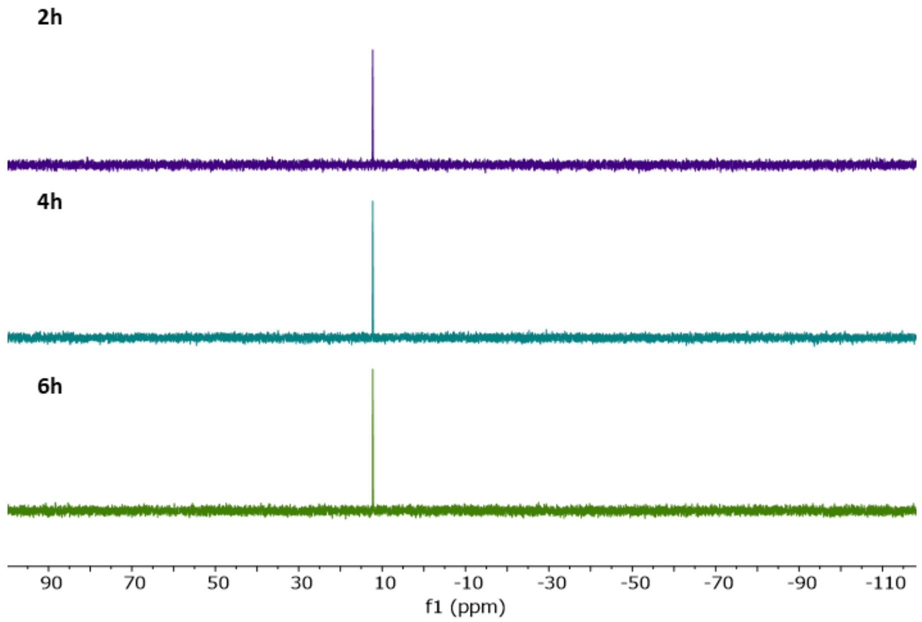

Figure S33. $^{31}P\{^{1}H\}$ NMR spectra (THF-d_8, 121 MHz) of the **oo** isomer of [$PdCl_2$(**DTE-$COCF_3$**)$_2$] kept at 50 °C for 6 h.

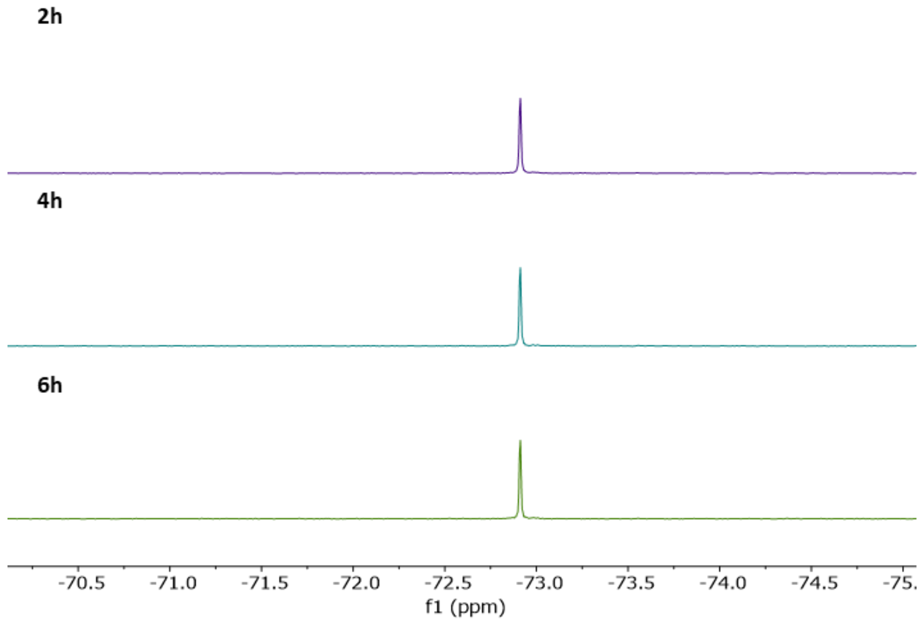

Figure S34. ^{19}F NMR spectra (THF-d_8, 282 MHz) of the **oo** isomer of [$PdCl_2$(**DTE-$COCF_3$**)$_2$] kept at 50 °C for 6 h.

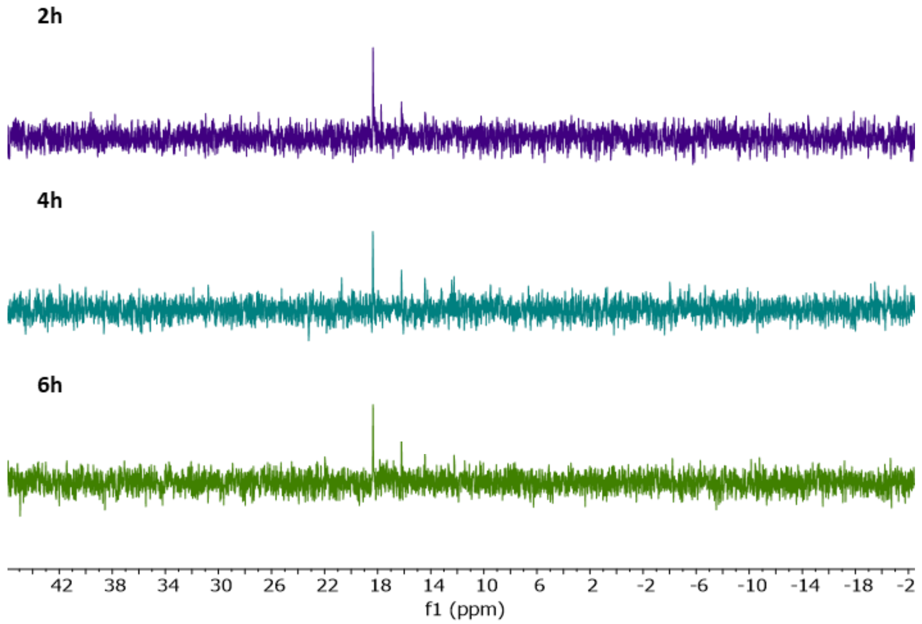

Figure S35. $^{31}P\{^{1}H\}$ NMR spectra (THF-d_8, 121 MHz) of the closed state-enriched mixture of **oo**, **oc** and **cc** isomers of [PdCl$_2$(**DTE-COCF$_3$**)$_2$] used in the catalytic experiments kept at 50 °C for 6 h.

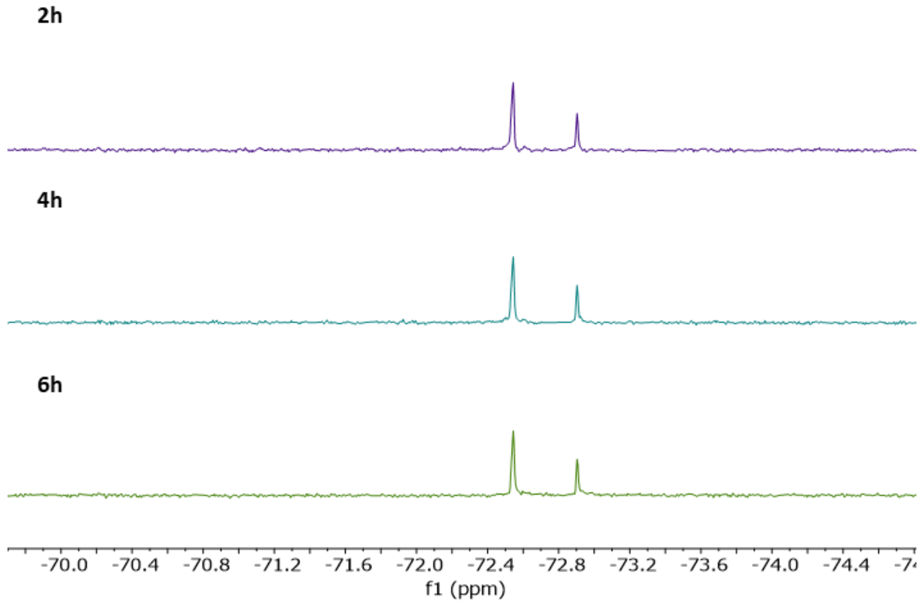

Figure S36. ^{19}F NMR spectra (THF-d_8, 282 MHz) of the closed state-enriched mixture of **oo**, **oc** and **cc** isomers of [PdCl$_2$(**DTE-COCF$_3$**)$_2$] used in the catalytic experiments kept at 50 °C for 6 h..

4.3 Catalytic reaction kinetic profiles

Several preliminary experiments were conducted to select the final conditions for catalytic experiments. First, the solubility of some of the complexes in toluene was found not to be high enough to achieve the desired catalyst loadings. Such high solubilities were accomplished with THF, which is another typical solvent for Stille coupling reactions. Second, we also explored the use of higher temperatures (refluxing THF), but we observed degradation of the palladium complexes after a few hours. For this reason, catalytic experiments were only carried out at room temperature and 50 °C. Finally, assays were also conducted at higher pre-catalyst loadings (5.0 mol%), but no significant improvement in conversions were observed. In light of that, a pre-catalyst loading of 1.5 mol% was finally selected for the experiments.

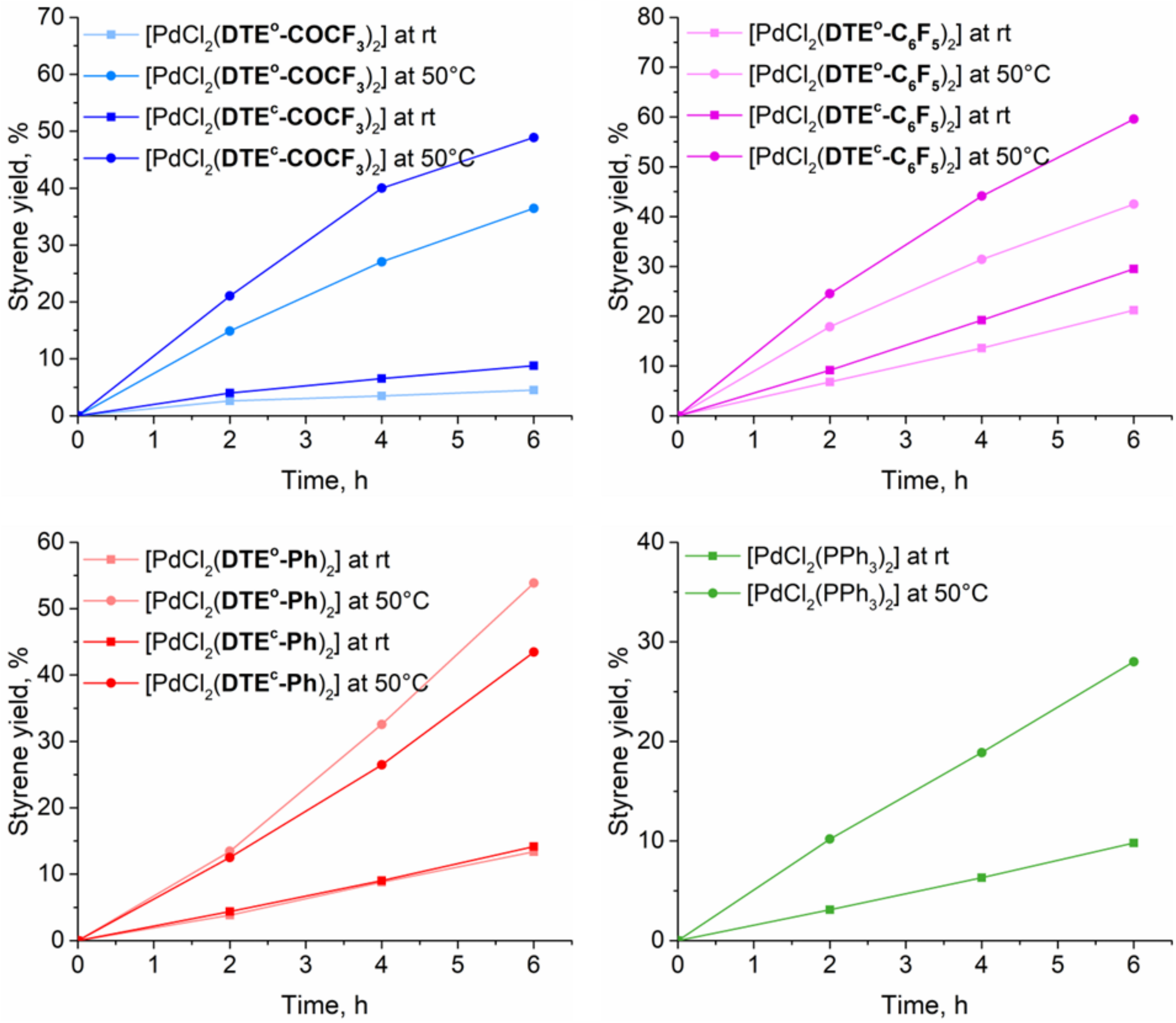

Figure S37. Yield-over-time graphs for the Stille reaction between iodobenzene and tributylvinyltin (1.5 mol% [Pd], 0.30 mmol iodobenzene, 0.33 mmol tributylvinyltin, 1 mL THF). Data is shown for each of the Pd precatalysts used at rt and 50 °C: the open ([PdCl$_2$(**DTE^o-R**)$_2$]) and closed state-enriched mixture ([PdCl$_2$(**DTE^c-R**)$_2$]) of the DTE-based complexes, and [PdCl$_2$(PPh$_3$)$_2$]. In each case, the average yields of two repetitions were determined by ^{1}H NMR spectroscopy using 1,3,5,-trimethoxybenzene as a standard.

4.4 Gibbs energy profiles

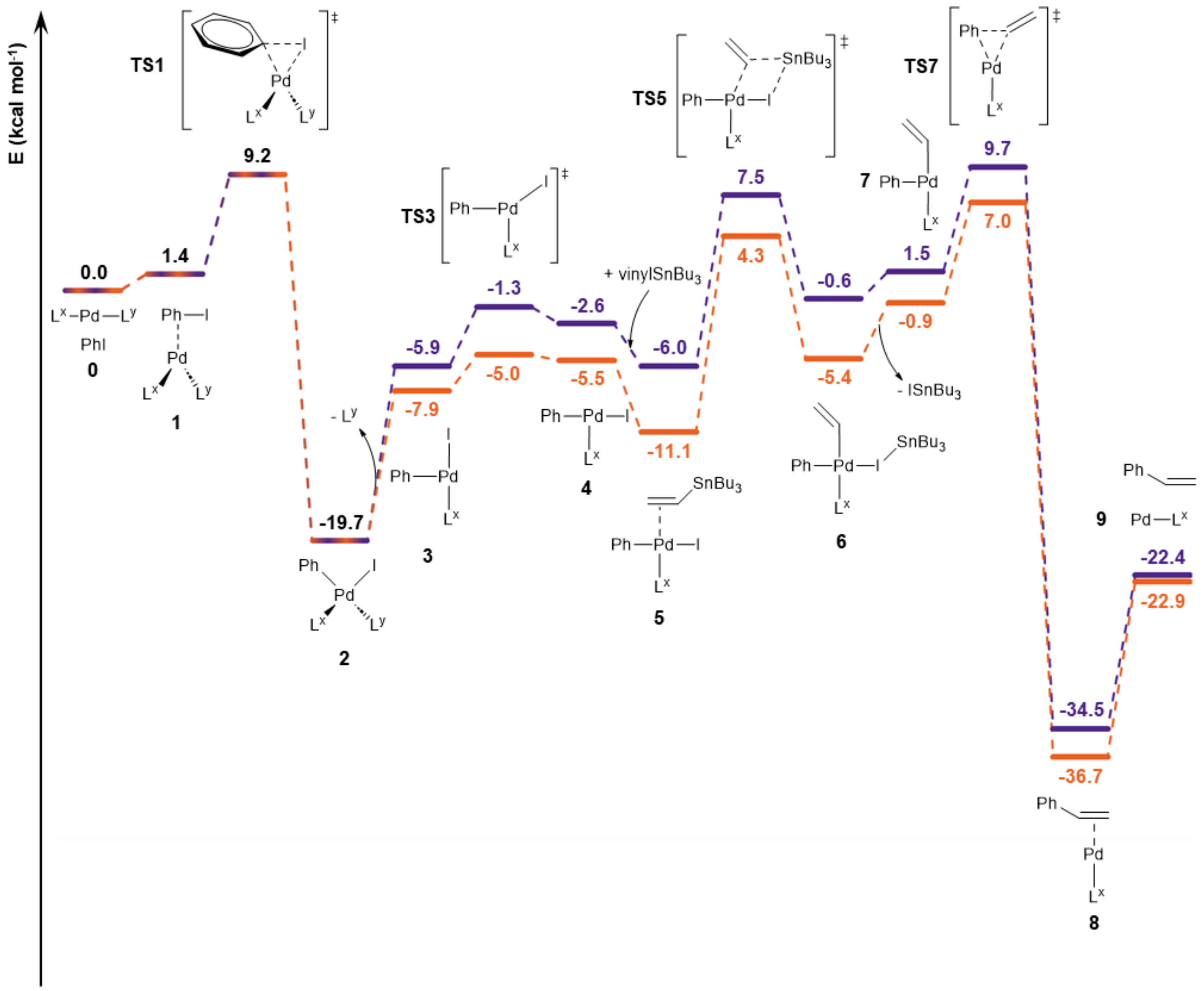

Figure S38. Calculated Gibbs energy profile for the Stille coupling reaction in solution (THF) using [$PdCl_2$(**DTE°-COCF$_3$**)(**DTE^c-COCF$_3$**)] as a pre-catalyst, where the orange line corresponds to L^x = **DTE°-COCF$_3$** and L^y = **DTE^c-COCF$_3$**, and the purple line corresponds to L^x = **DTE^c-COCF$_3$** and L^y = **DTE°-COCF$_3$**.

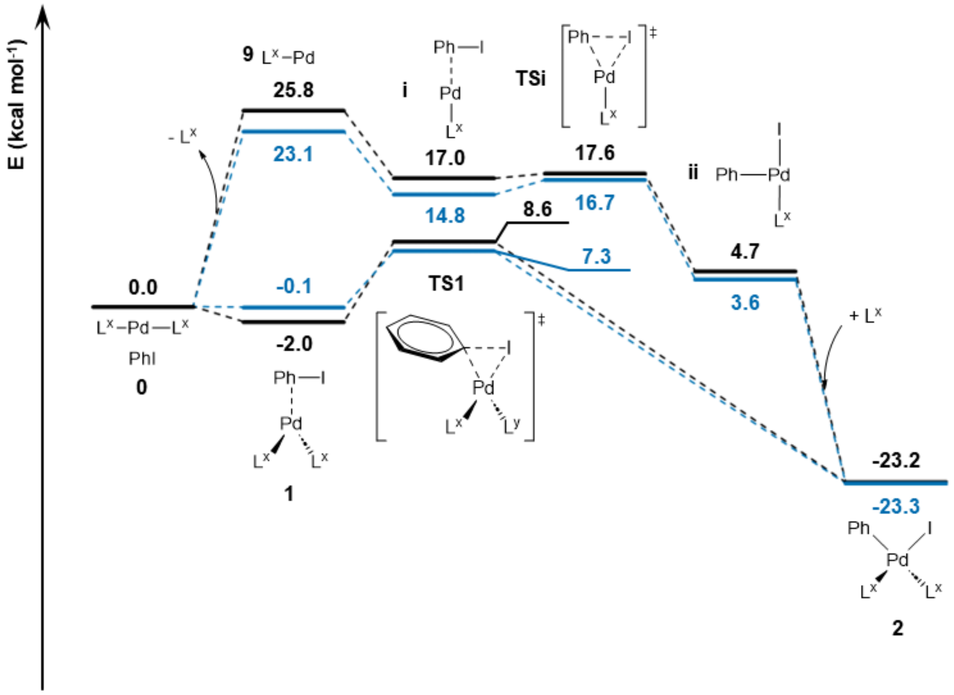

Figure S39. Comparison of the monoligated and bisligated oxidative addition for the Stille coupling reaction in solution (THF) using [PdCl$_2$(**DTE^o-COCF$_3$**)$_2$] or [PdCl$_2$(**DTE^c-COCF$_3$**)$_2$] as a pre-catalyst, where L^x is **DTE^o-COCF$_3$** (black line) or **DTE^c-COCF$_3$** (blue line).

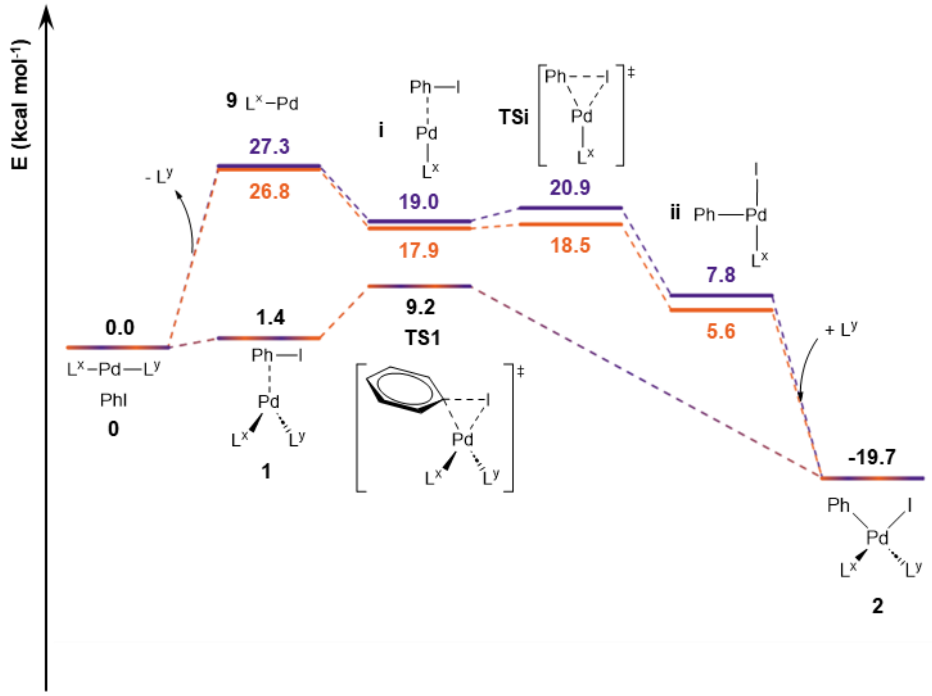

Figure S40. Comparison of the monoligated and bisligated oxidative addition for the Stille coupling reaction in solution (THF) using [PdCl$_2$(**DTE^o-COCF$_3$**)(**DTE^c-COCF$_3$**)] as a pre-catalyst, where the orange line corresponds to L^x = **DTE^o-COCF$_3$** and L^y = **DTE^c-COCF$_3$**, and the purple line corresponds to L^x = **DTE^c-COCF$_3$** and L^y = **DTE^o-COCF$_3$**.

Table S6. Absolute energies of the calculated species (a. u.) for the Stille coupling reaction. Gibbs energy profiles are presented in Figures 4, S33-S35.

Structure	Parameter	L_x = **DTE^{o}-COCF$_3$**	L_x = **DTE^{c}-COCF$_3$**	L_x = **DTE^{o}-COCF$_3$,** L_y = **DTE^{c}-COCF$_3$**	L_x = **DTE^{c}-COCF$_3$,** L_y = **DTE^{o}-COCF$_3$**
**0**	E (BS1)	-5392.239321	-5392.205567	-5392.220537	
	E (BS2)	-5393.608067	-5393.557983	-5393.5821	
	$G_{298.15K}$ (BS2)	-5392.812613	-5392.752535	-5392.786663	
**1**	E (BS1)	-5635.349282	-5635.313115	-5635.329212	
	E (BS2)	-5923.177619	-5923.124946	-5923.150639	
	$G_{298.15K}$ (BS2)	-5922.302111	-5922.238982	-5922.270821	
**TS1**	E (BS1)	-5635.33444	-5635.299611	-5635.319281	
	E (BS2)	-5923.163128	-5923.111584	-5923.139066	
	$G_{298.15K}$ (BS2)	-5922.285269	-5922.227193	-5922.258337	
**2**	E (BS1)	-5635.394044	-5635.358098	-5635.375825	
	E (BS2)	-5923.220121	-5923.167801	-5923.193047	
	$G_{298.15K}$ (BS2)	-5922.335855	-5922.276059	-5922.304301	
**3**	E (BS1)	-3003.228512	-3003.208841		
	E (BS2)	-3290.39413	-3290.366143		
	$G_{298.15K}$ (BS2)	-3289.930467	-3289.899937		
**TS3**	E (BS1)	-3003.224764	-3003.204962		
	E (BS2)	-3290.390739	-3290.362707		
	$G_{298.15K}$ (BS2)	-3289.925962	-3289.892501		
**4**	E (BS1)	-3003.225294	-3003.205886		
	E (BS2)	-3290.391244	-3290.363581		
	$G_{298.15K}$ (BS2)	-3289.926688	-3289.894564		
**5**	E (BS1)	-3558.264195	-3558.241926		
	E (BS2)	-4056.593907	-4056.563138		
	$G_{298.15K}$ (BS2)	-4055.75176	-4055.716096		
**TS5**	E (BS1)	-3558.239633	-3558.217603		
	E (BS2)	-4056.570546	-4056.540686		
	$G_{298.15K}$ (BS2)	-4055.727214	-4055.694618		
**6**	E (BS1)	-3558.248848	-3558.230558		
	E (BS2)	-4056.582015	-4056.555734		
	$G_{298.15K}$ (BS2)	-4055.742589	-4055.707574		
**7**	E (BS1)	-3069.723993	-3069.704812		
	E (BS2)	-3070.53496	-3070.507582		
	$G_{298.15K}$ (BS2)	-3070.031406	-3070.000007		
**TS7**	E (BS1)	-3069.712283	-3069.693922		
	E (BS2)	-3070.523049	-3070.496522		
	$G_{298.15K}$ (BS2)	-3070.018803	-3069.986939		
**8**	E (BS1)	-3069.785511	-3069.76739		
	E (BS2)	-3070.594751	-3070.568412		
	$G_{298.15K}$ (BS2)	-3070.088414	-3070.057437		
**9**	E (BS1)	-2760.067449	-2760.049102		
	E (BS2)	-2760.773088	-2760.747087		
	$G_{298.15K}$ (BS2)	-2760.388924	-2760.360675		
**i**	E (BS1)	-3003.183733	-3003.165024		
	E (BS2)	-3290.350084	-3290.323456		
	$G_{298.15K}$ (BS2)	-3289.889373	-3289.860206		
**TSi**	E (BS1)	-3003.182966	-3003.164299		
	E (BS2)	-3290.349626	-3290.322925		

	$G_{298.15K}$ (BS2)	-3289.888408	-3289.857102	
**ii**	E (BS1)	-3003.2053	-3003.186745	
	E (BS2)	-3290.370492	-3290.34397	
	$G_{298.15K}$ (BS2)	-3289.909013	-3289.878047	

PhI E (BS1) = -243.0808839

E (BS2) = -529.5483685

$G_{298.15K}$ (BS2) = -529.4893265

DTE^{o}-COCF$_3$ E (BS1) = -2632.101518

E (BS2) = -2632.771651

$G_{298.15K}$ (BS2) = -2632.385516

DTE^{c}-COCF$_3$ E (BS1) = -2632.086005

E (BS2) = -2632.748231

$G_{298.15K}$ (BS2) = -2632.358075

Sn(CH$_2$CH)Bu$_3$ E (BS1) = -554.9948309

E (BS2) = -766.1689977

$G_{298.15K}$ (BS2) = -765.8191377

SnIBu$_3$ E (BS1) = -488.4879923

E (BS2) = -986.0148693

$G_{298.15K}$ (BS2) = -985.7071573

Styrene E (BS1) = -309.6714908

E (BS2) = -309.7830068

$G_{298.15K}$ (BS2) = -309.6805298

5 NMR spectra of the reported compounds

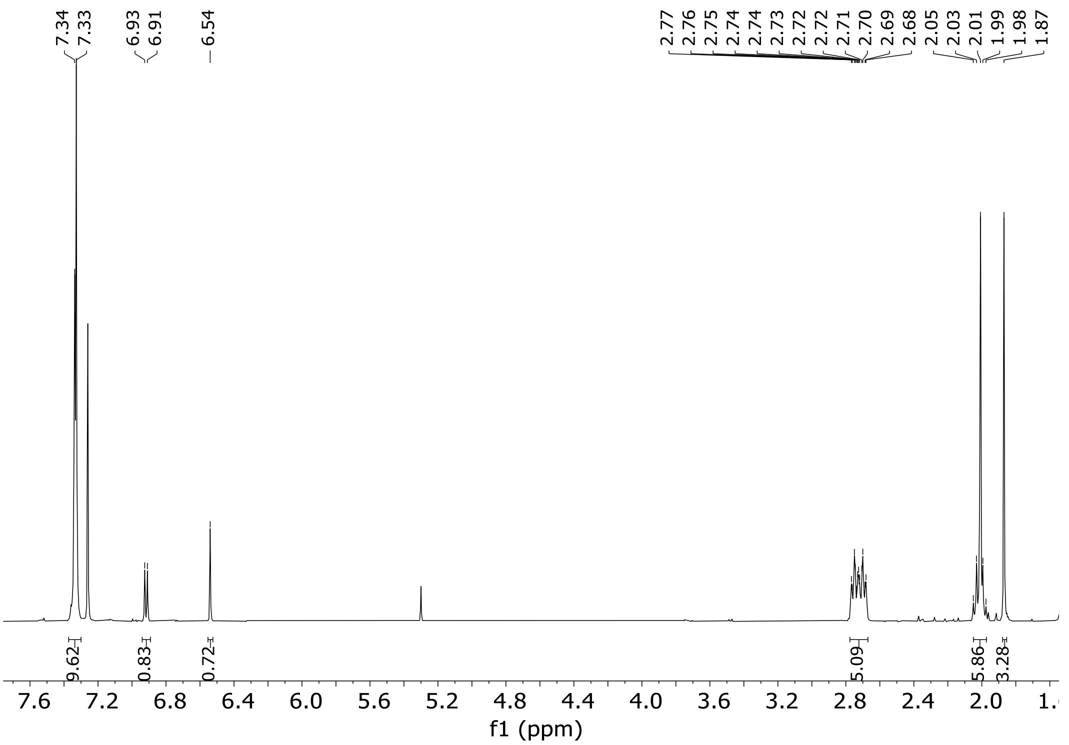

Figure S41. ^{1}H NMR spectrum (400 MHz) of **DTE2** in $CDCl_3$ recorded at 25 °C.

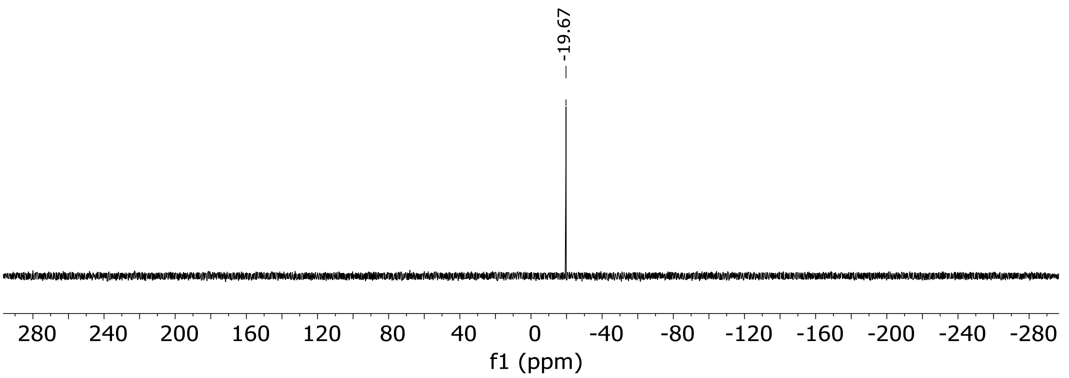

Figure S42. $^{31}P\{^{1}H\}$ NMR spectrum (162 MHz) of **DTE2** in $CDCl_3$ recorded at 25 °C.

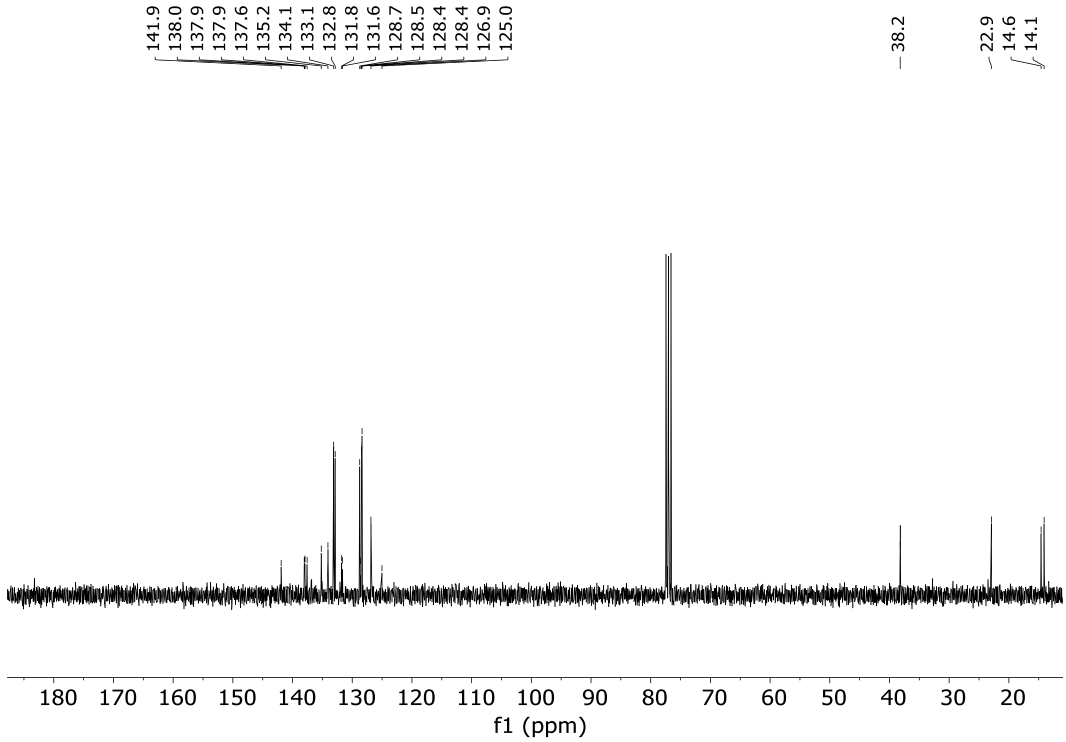

Figure S43. ${}^{13}C\{{}^{1}H\}$ NMR spectrum (75 MHz) of **DTE2** in $CDCl_3$ recorded at 25 °C.

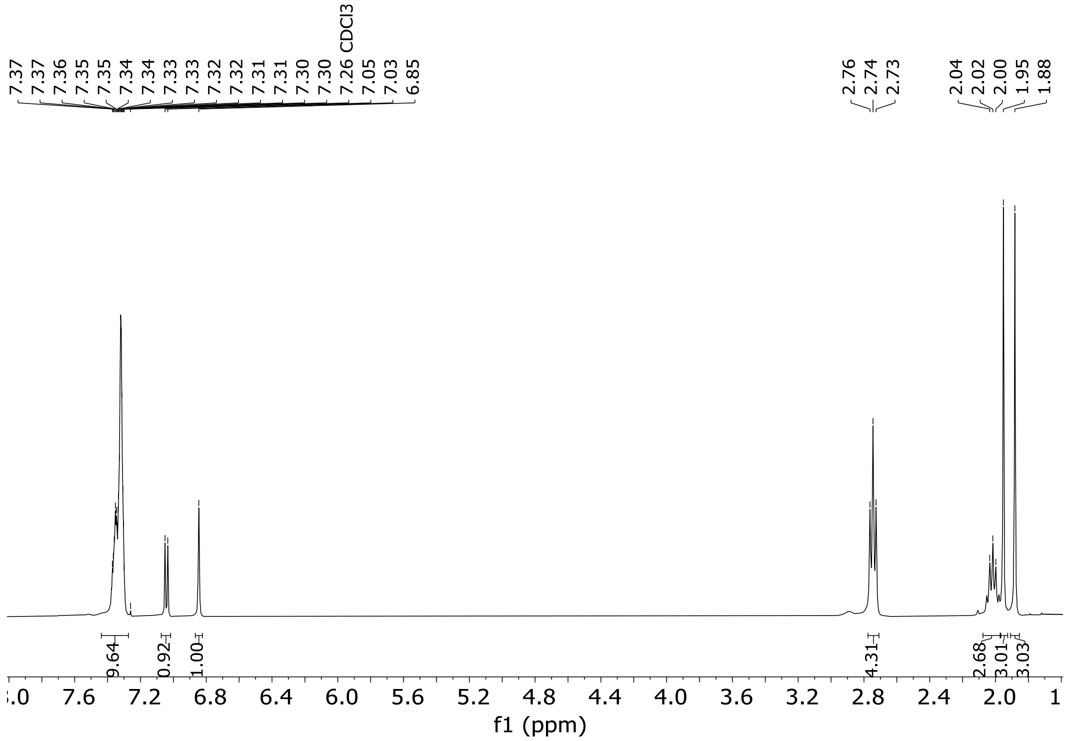

Figure S44. ${}^{1}H$ NMR spectrum (400 MHz) of **DTE-COCF₃** in $CDCl_3$ recorded at 25 °C.

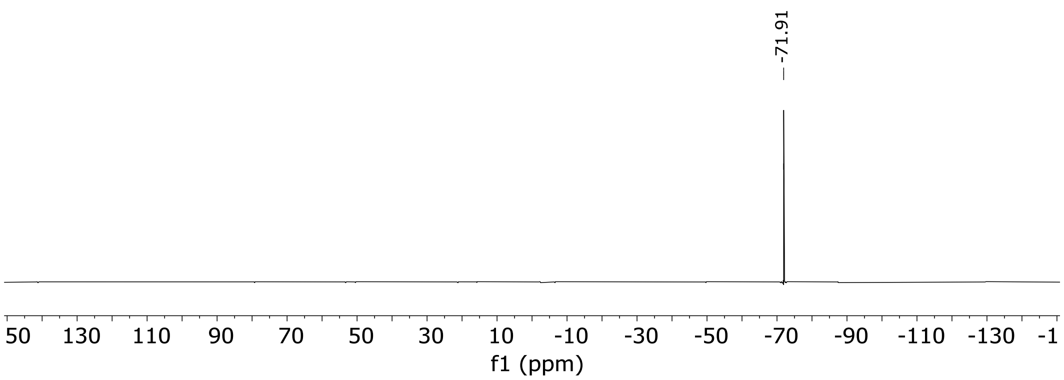

Figure S45. ^{19}F NMR spectrum (376 MHz) of **DTE-COCF₃** in $CDCl_3$ recorded at 25 °C.

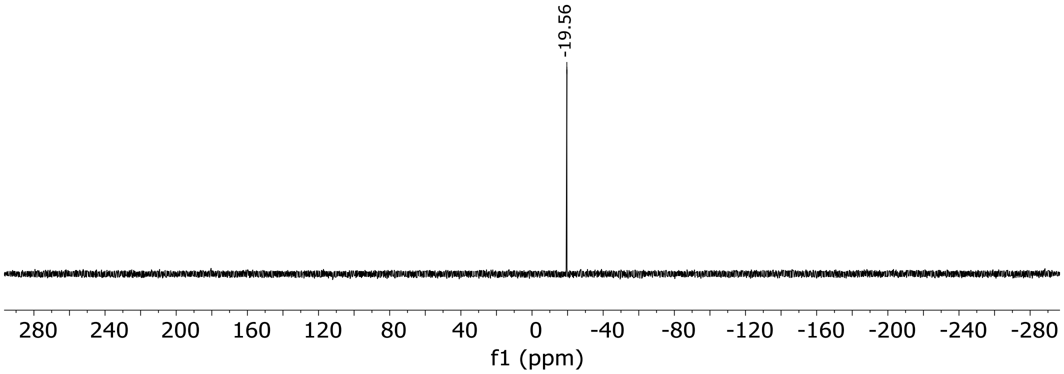

Figure S46. $^{31}P\{^{1}H\}$ NMR spectrum (162 MHz) of **DTE-COCF₃** in $CDCl_3$ recorded at 25 °C.

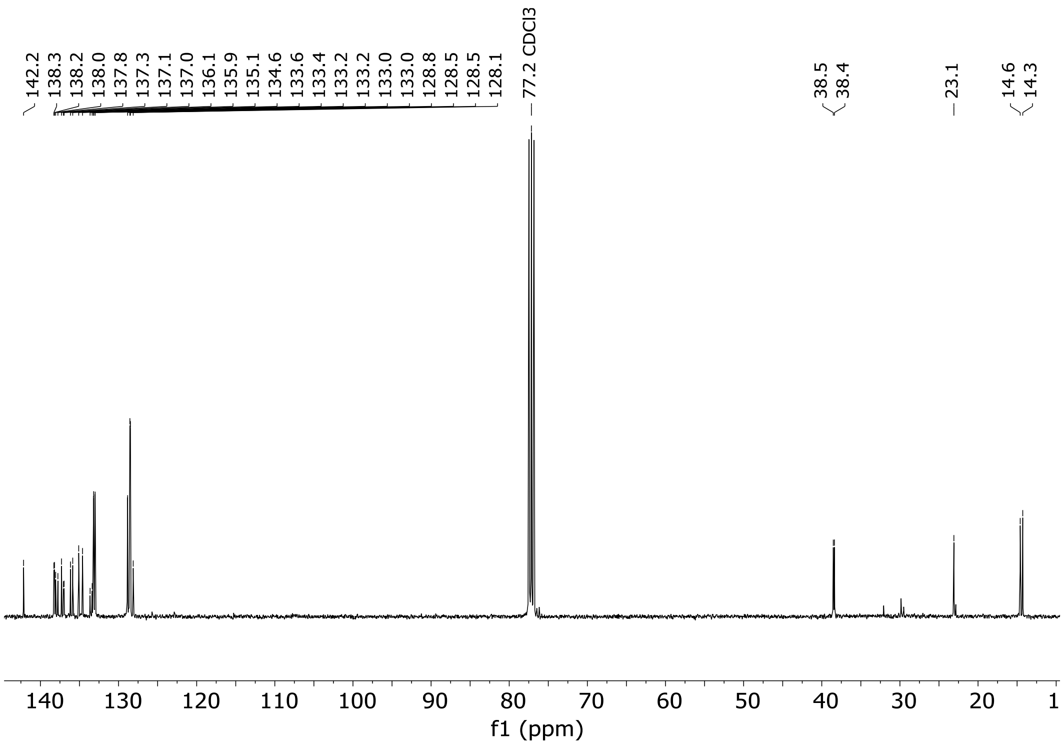

Figure S47. $^{13}C\{^{1}H\}$ NMR spectrum (101 MHz) of **DTE-COCF₃** in $CDCl_3$ recorded at 25 °C.

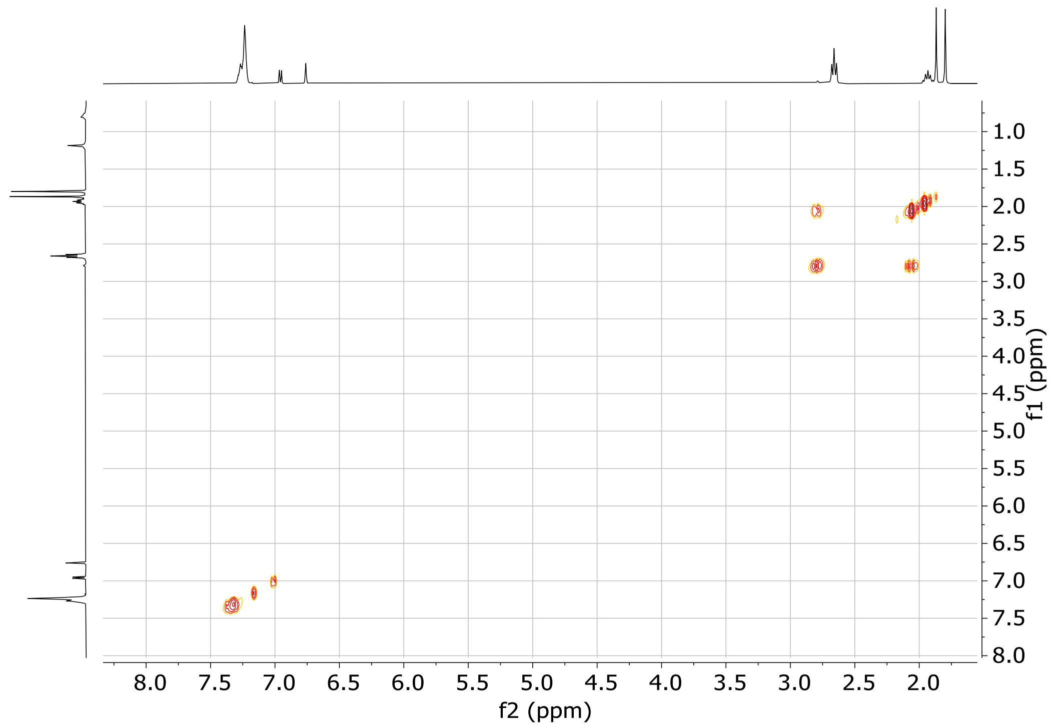

Figure S48. ^1H-^1H COSY spectrum of **DTE-COCF$_3$** in $CDCl_3$ recorded at 25 °C.

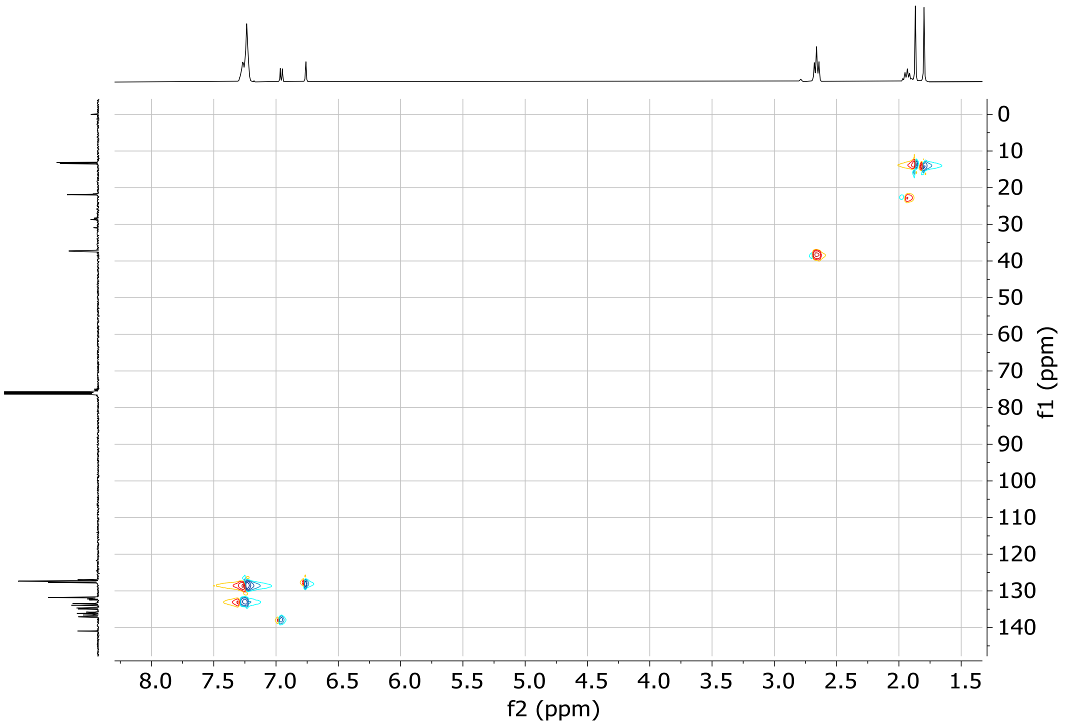

Figure S49. ^1H-^{13}C HSQC spectrum of **DTE-COCF$_3$** in $CDCl_3$ recorded at 25 °C.

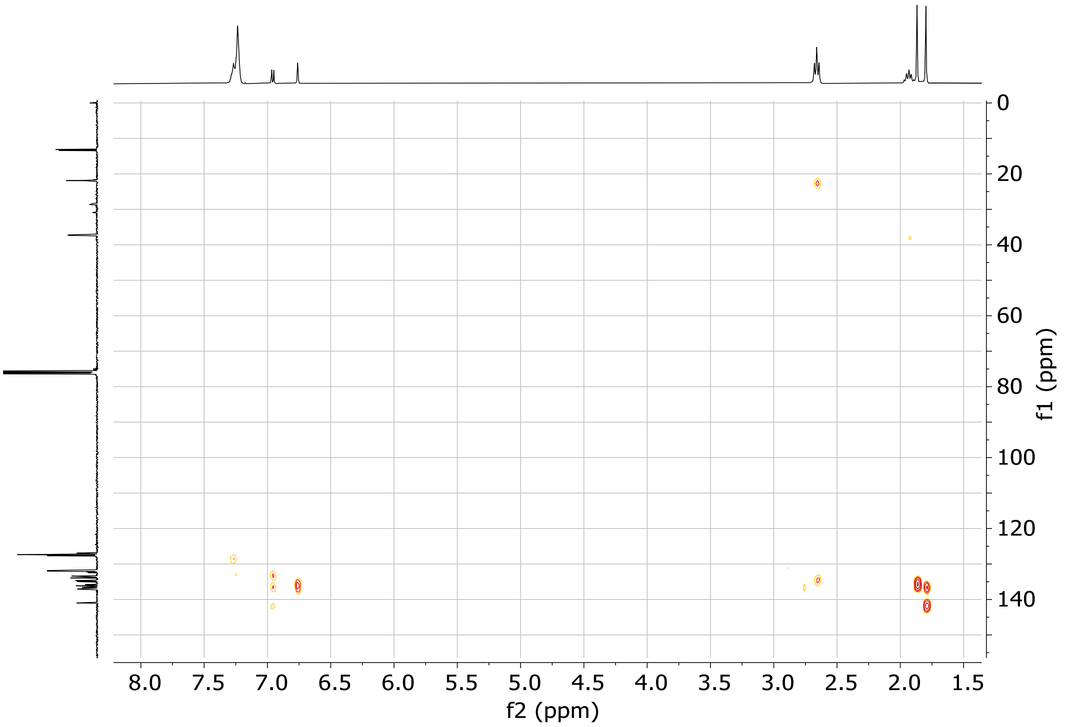

Figure S50. ^{1}H-^{13}C HMBC spectrum of **DTE-COCF$_3$** in CDCl$_3$ recorded at 25 °C.

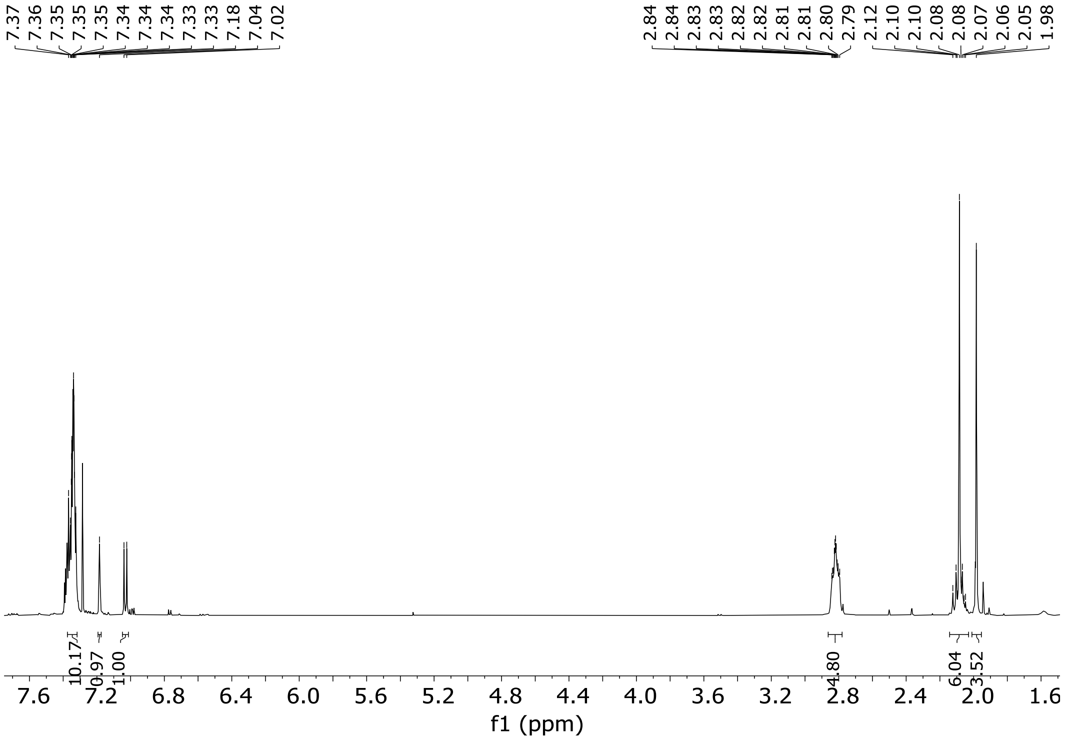

Figure S51. ^{1}H NMR spectrum (400 MHz) of **DTE-C$_6$F$_5$** in CDCl$_3$ recorded at 25 °C.

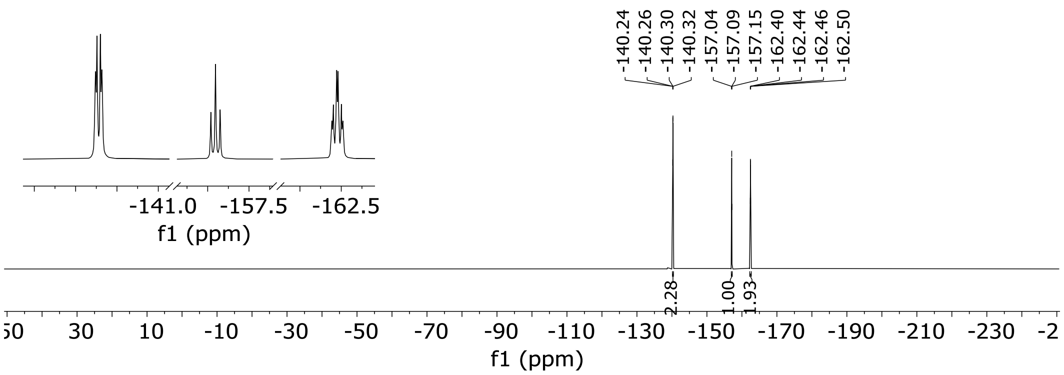

Figure S52. ^{19}F NMR spectrum (376 MHz) of **DTE-C_6F_5** in $CDCl_3$ recorded at 25 °C.

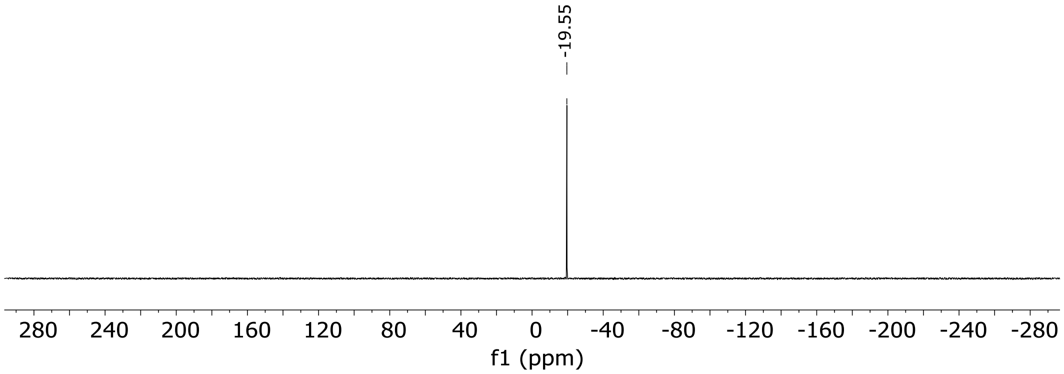

Figure S53. ^{31}P{^{1}H} NMR spectrum (162 MHz) of **DTE-C_6F_5** in $CDCl_3$ recorded at 25 °C.

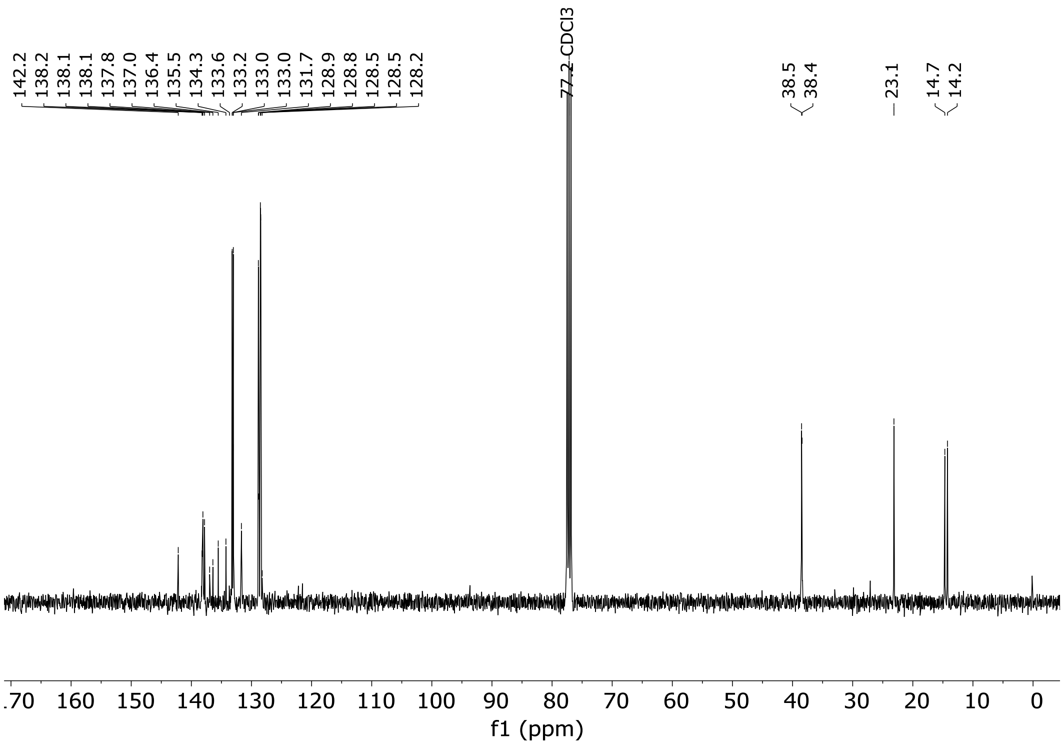

Figure S54. ^{13}C{^{1}H} NMR spectrum (101 MHz) of **DTE-C_6F_5** in $CDCl_3$ recorded at 25 °C.

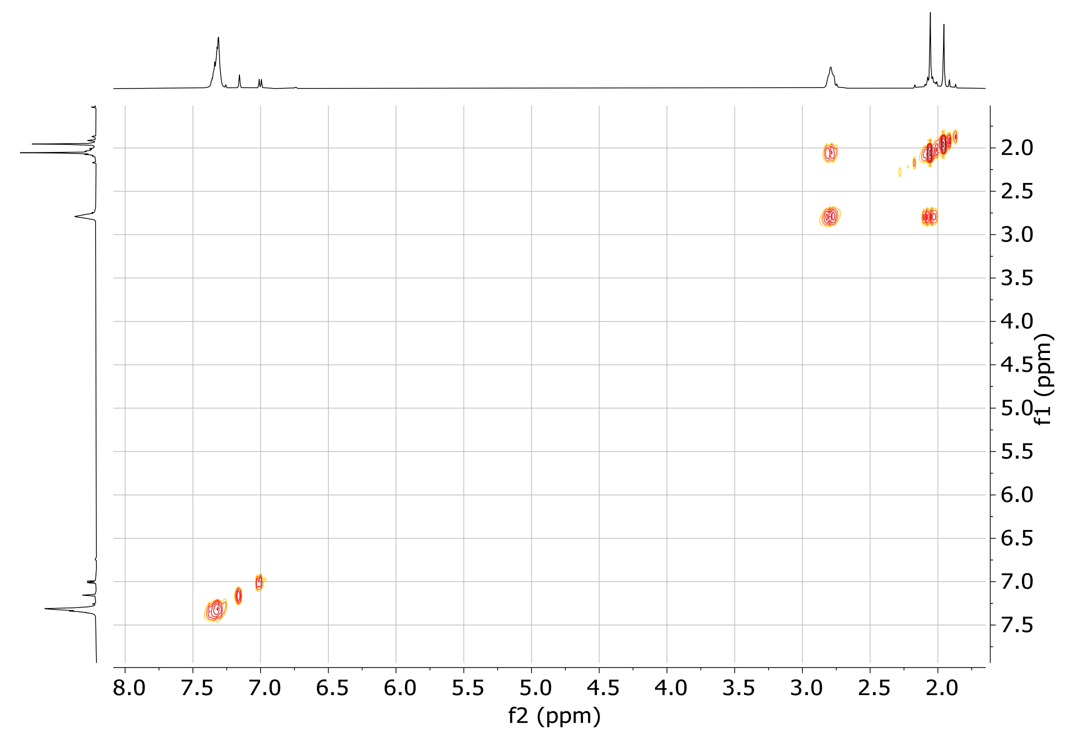

Figure S55. ^{1}H-^{1}H COSY spectrum of **DTE-C_6F_5** in $CDCl_3$ recorded at 25 °C.

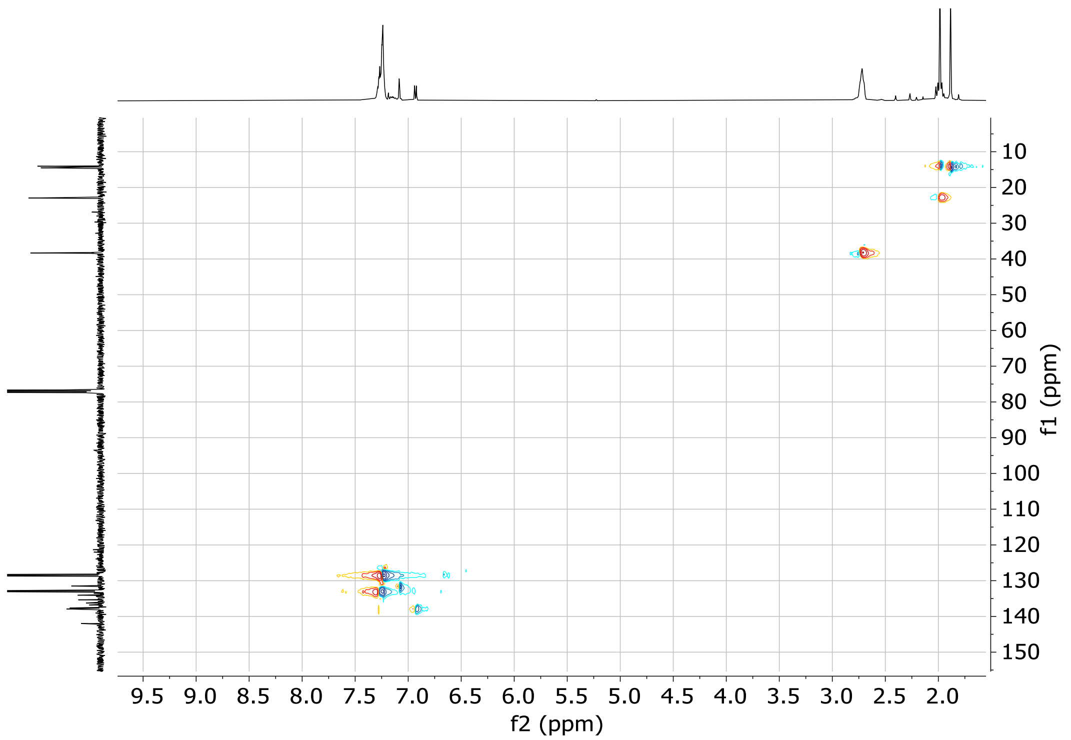

Figure S56. ^{1}H-^{13}C HSQC spectrum of **DTE-C_6F_5** in $CDCl_3$ recorded at 25 °C.

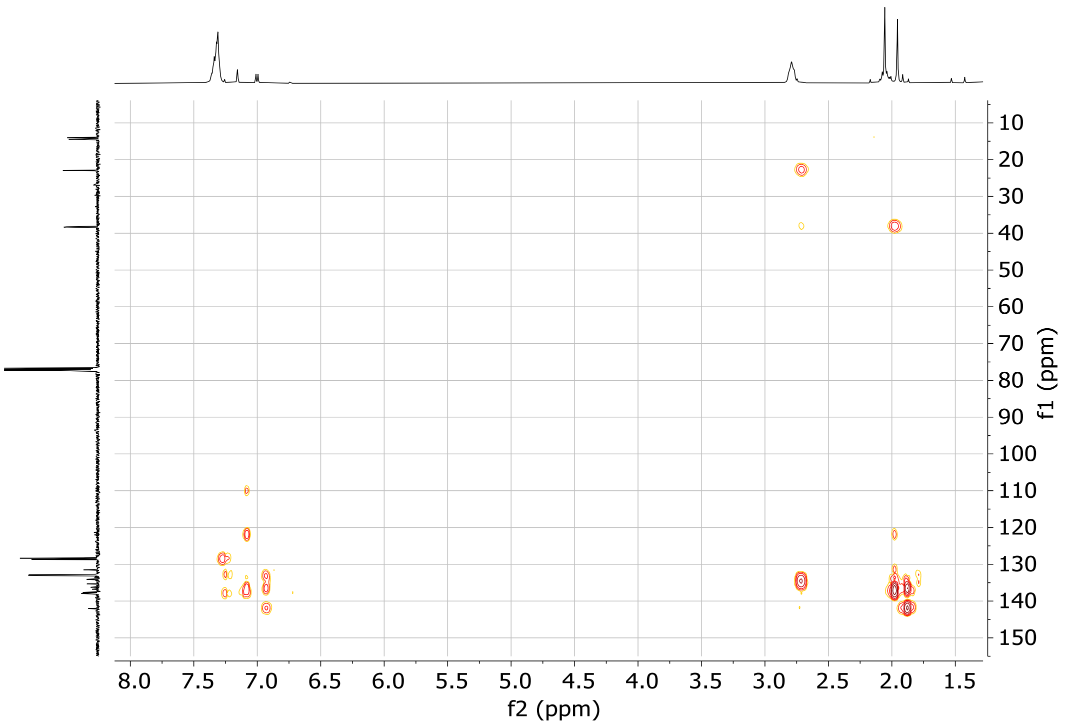

Figure S57. ^{1}H-^{13}C HMBC spectrum of **DTE-C$_6$F$_5$** in $CDCl_3$ recorded at 25 °C.

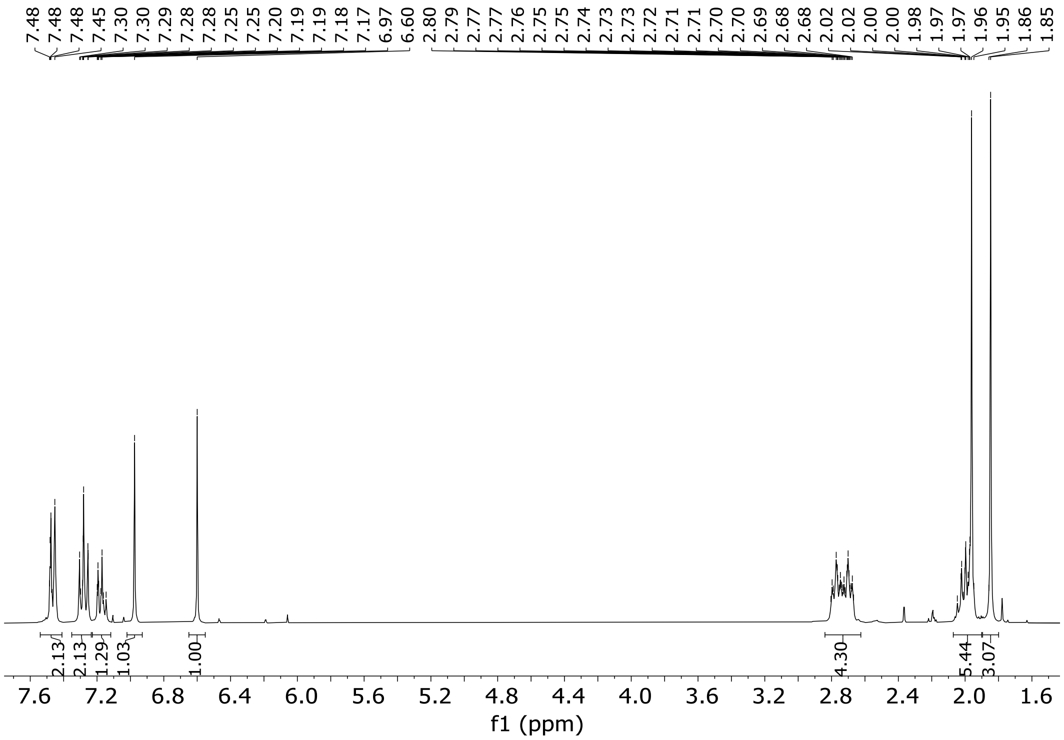

Figure S58. ^{1}H NMR spectrum (300 MHz) of **DTE3** in $CDCl_3$ recorded at 25 °C.

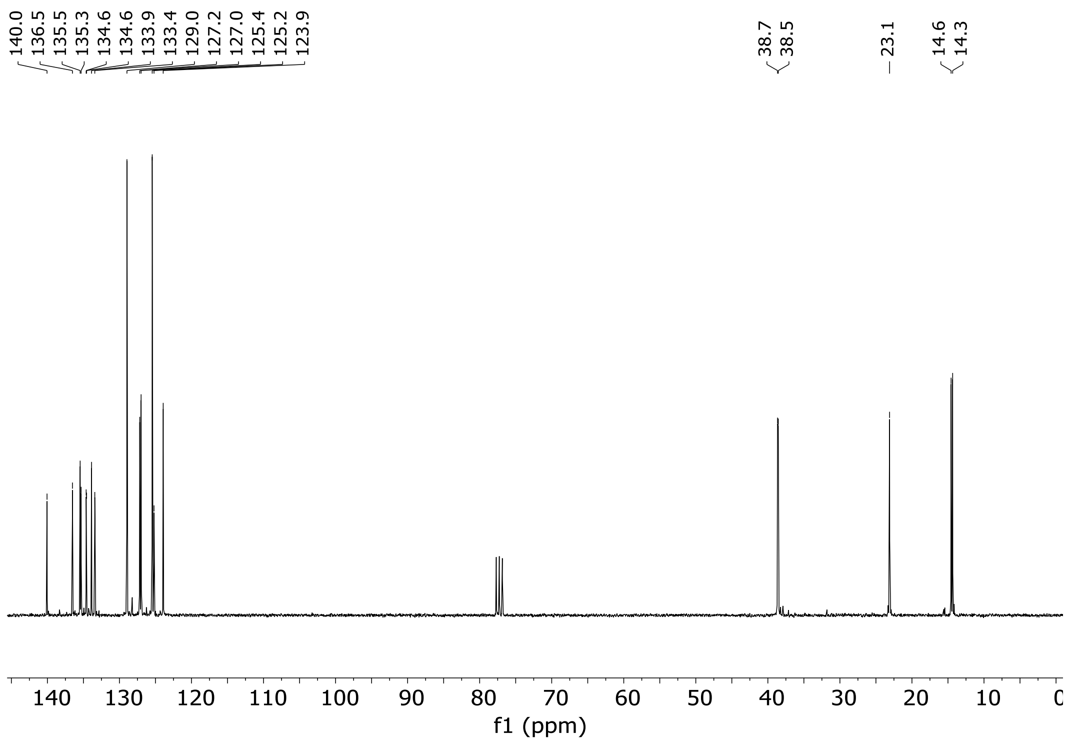

Figure S59. $^{13}C\{^1H\}$ NMR spectrum (75 MHz) of **DTE3** in $CDCl_3$ recorded at 25 °C.

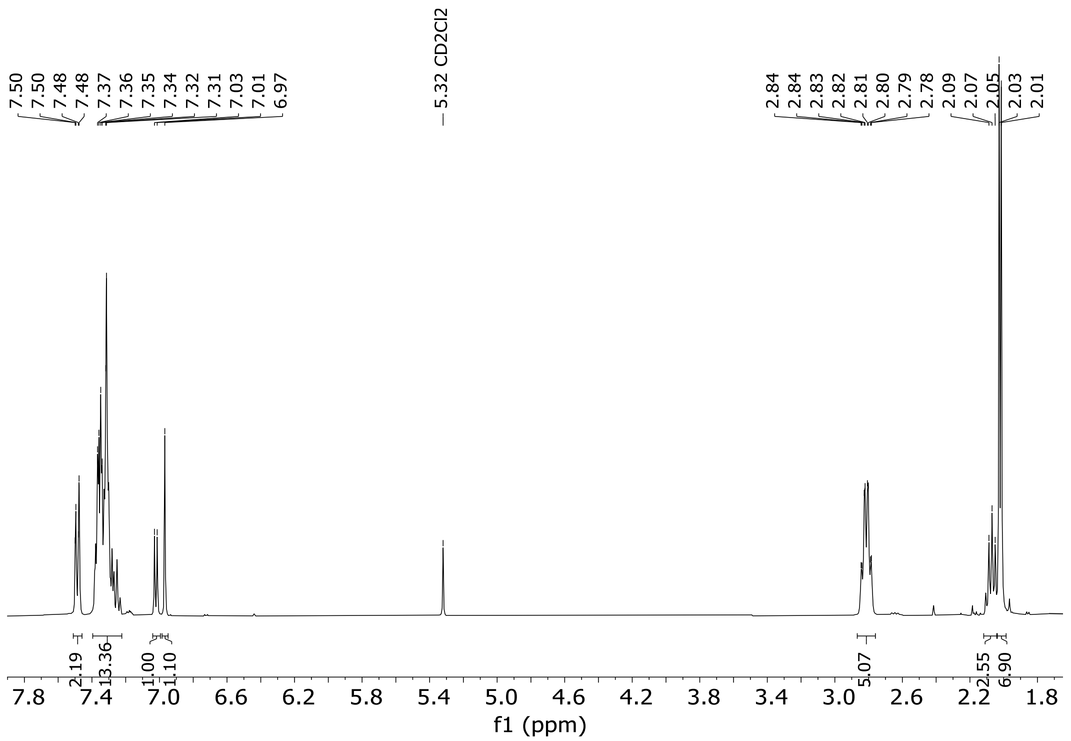

Figure S60. ^1H NMR spectrum (400 MHz) of **DTE-Ph** in $CDCl_3$ recorded at 25 °C.

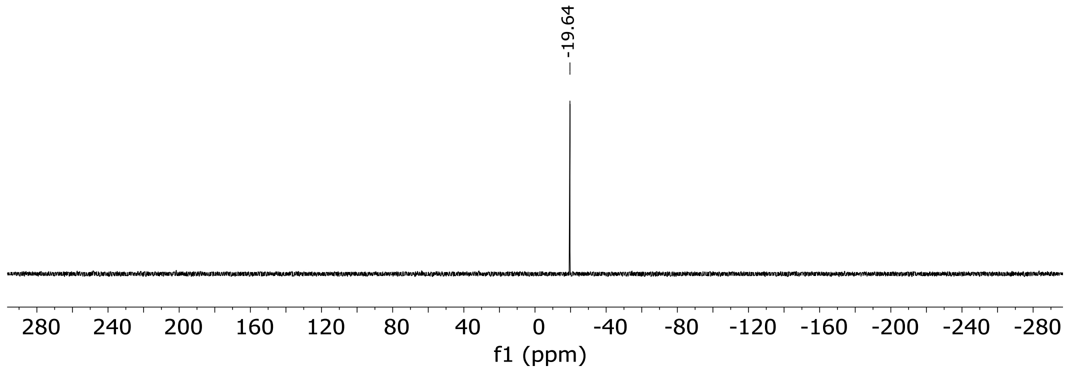

Figure S61. $^{31}P\{^{1}H\}$ NMR spectrum (162 MHz) of **DTE-Ph** in $CDCl_3$ recorded at 25 °C.

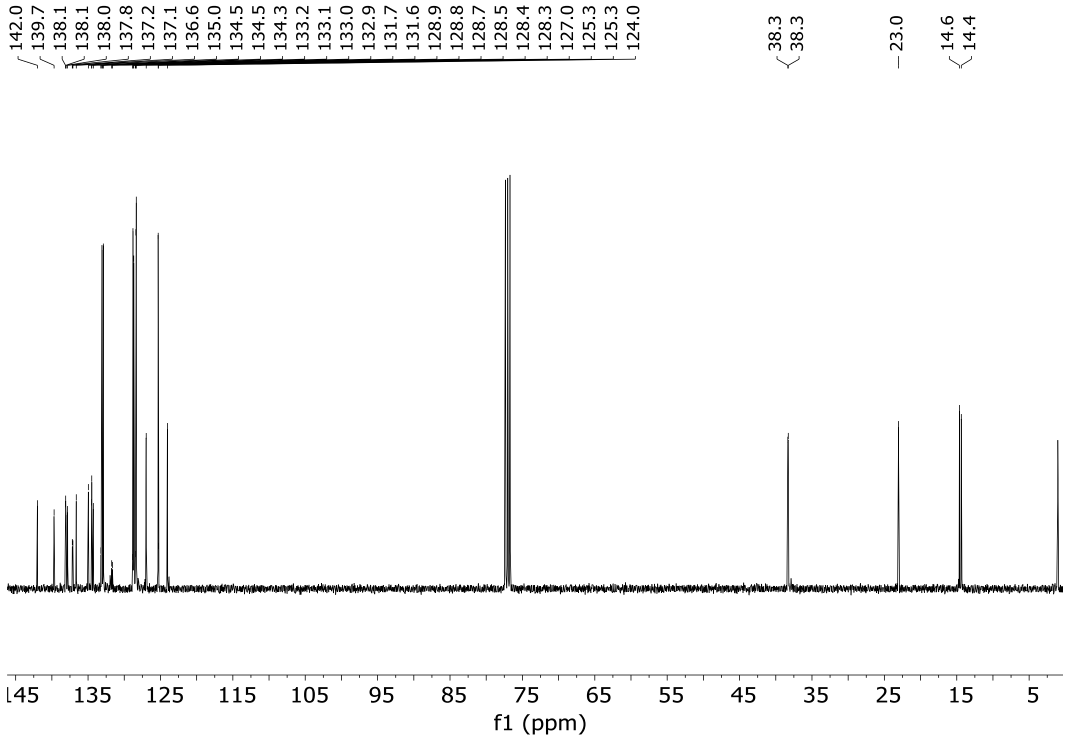

Figure S62. $^{13}C\{^{1}H\}$ NMR spectrum (101 MHz) of **DTE-Ph** in $CDCl_3$ recorded at 25 °C.

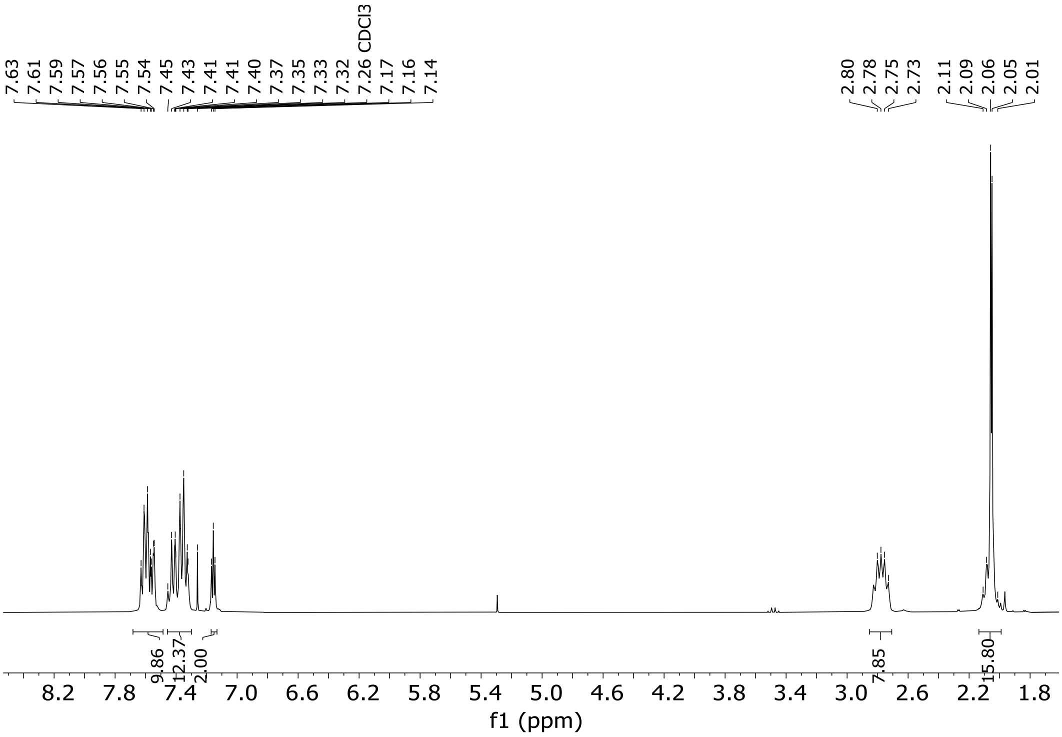

Figure S63. ^{1}H NMR spectrum (300 MHz) of [PdCl$_2$(**DTE°-COCF$_3$**)$_2$] in CDCl$_3$ recorded at 25 °C.

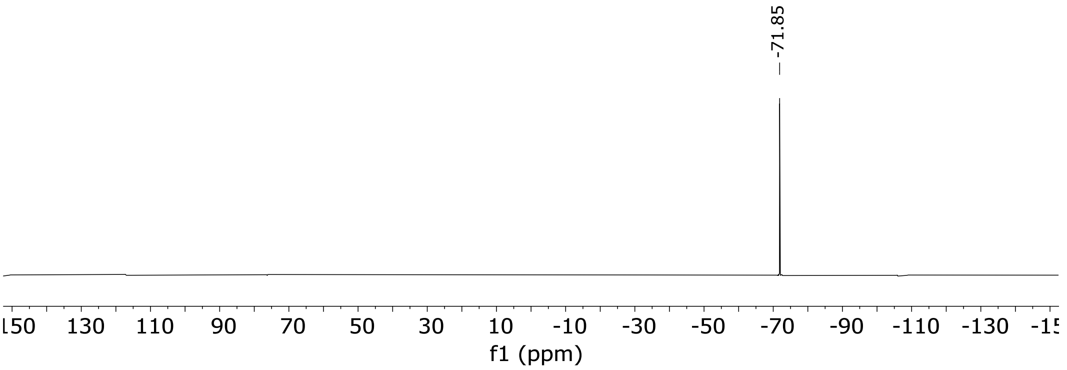

Figure S64. ^{19}F NMR spectrum (282 MHz) of [PdCl$_2$(**DTE°-COCF$_3$**)$_2$] in CDCl$_3$ recorded at 25 °C.

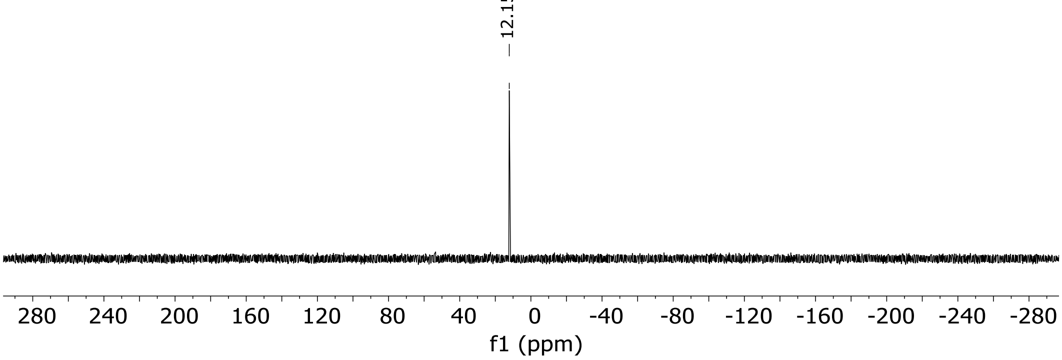

Figure S65. ^{31}P{^{1}H} NMR spectrum (162 MHz) of [PdCl$_2$(**DTE°-COCF$_3$**)$_2$] in CDCl$_3$ recorded at 25 °C.

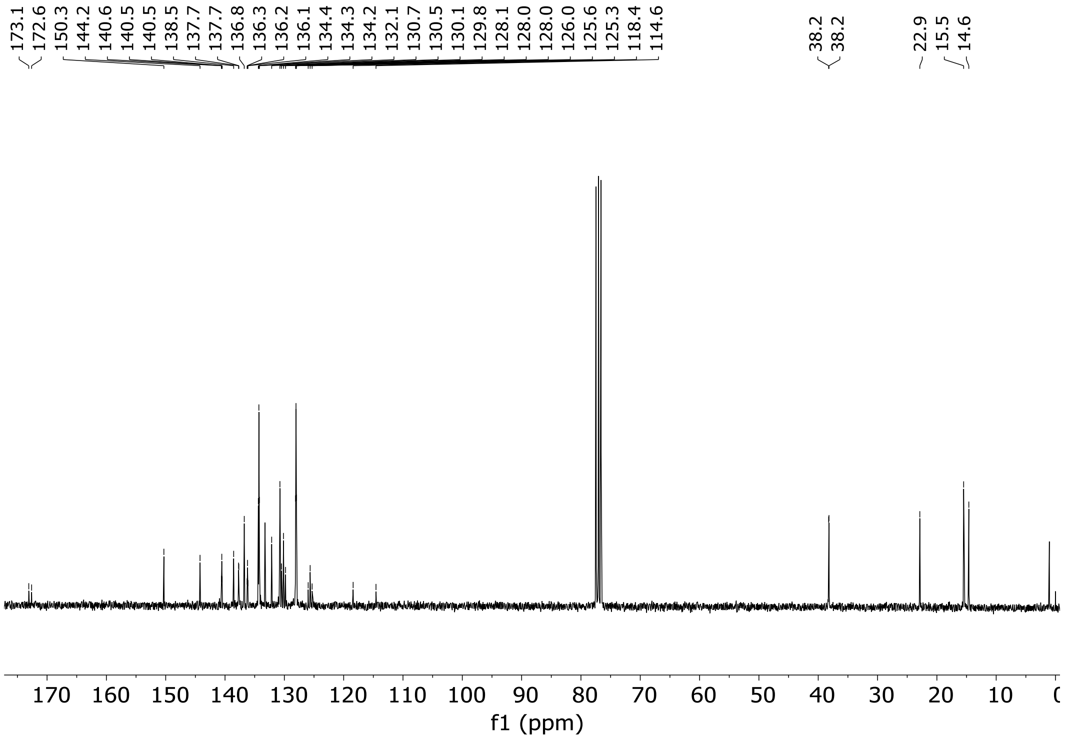

Figure S66. $^{13}C\{^{1}H\}$ NMR spectrum (75 MHz) of [$PdCl_2$(**DTE°-COCF$_3$**)$_2$] in $CDCl_3$ recorded at 25 °C.

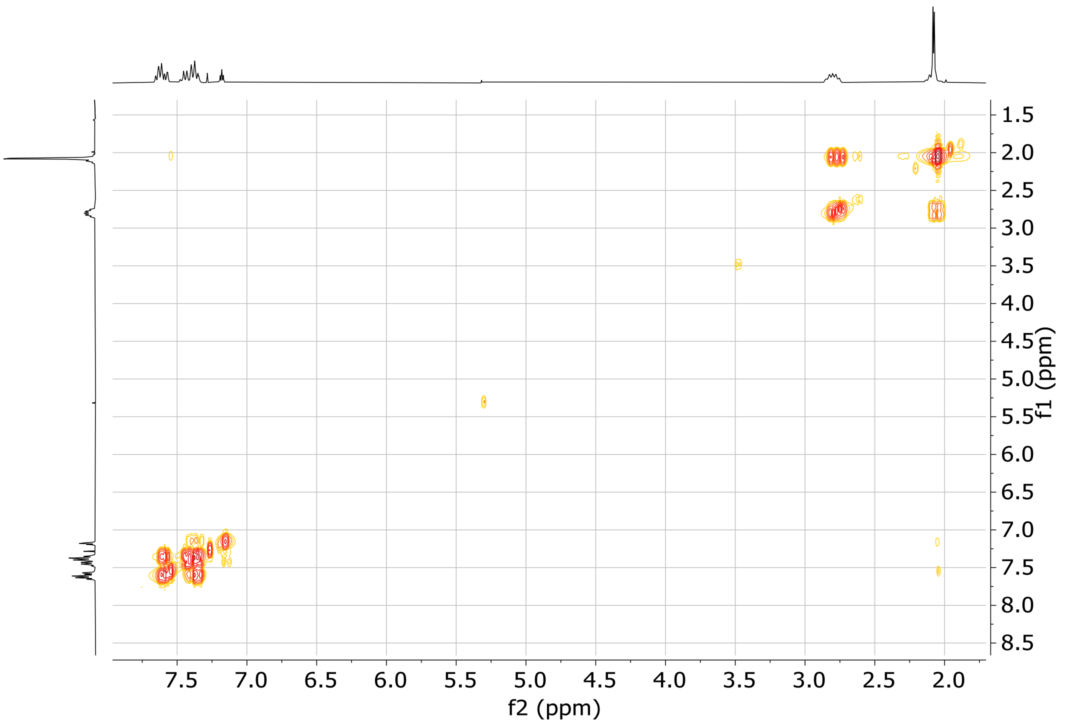

Figure S67. ^{1}H-^{1}H COSY spectrum of [$PdCl_2$(**DTE°-COCF$_3$**)$_2$] in $CDCl_3$ recorded at 25 °C.

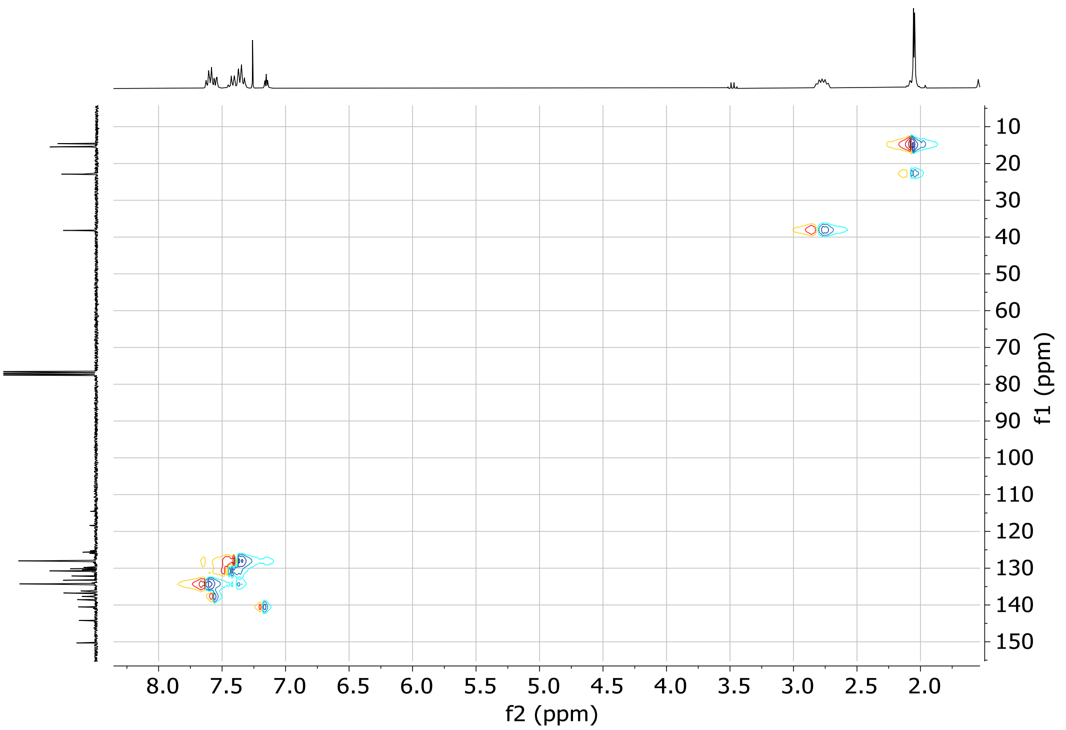

Figure S68. ^{1}H-^{13}C HSQC spectrum of [PdCl$_2$(**DTE°-COCF$_3$**)$_2$] in CDCl$_3$ recorded at 25 °C.

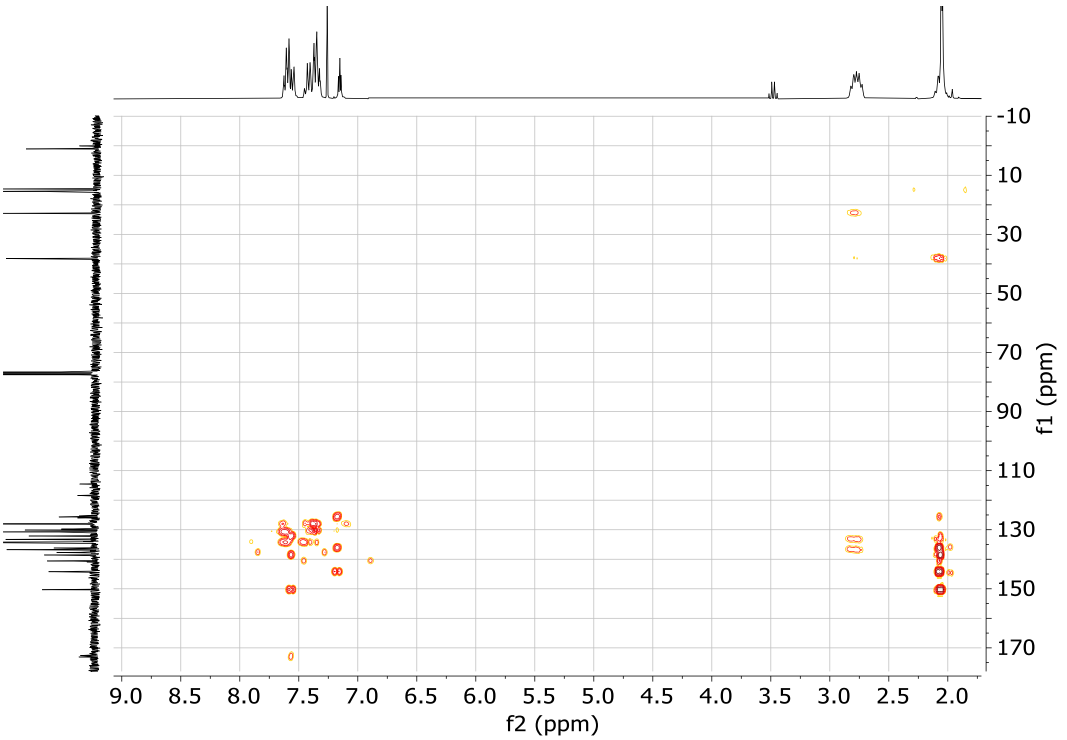

Figure S69. ^{1}H-^{13}C HMBC spectrum of [PdCl$_2$(**DTE°-COCF$_3$**)$_2$] in CDCl$_3$ recorded at 25 °C.

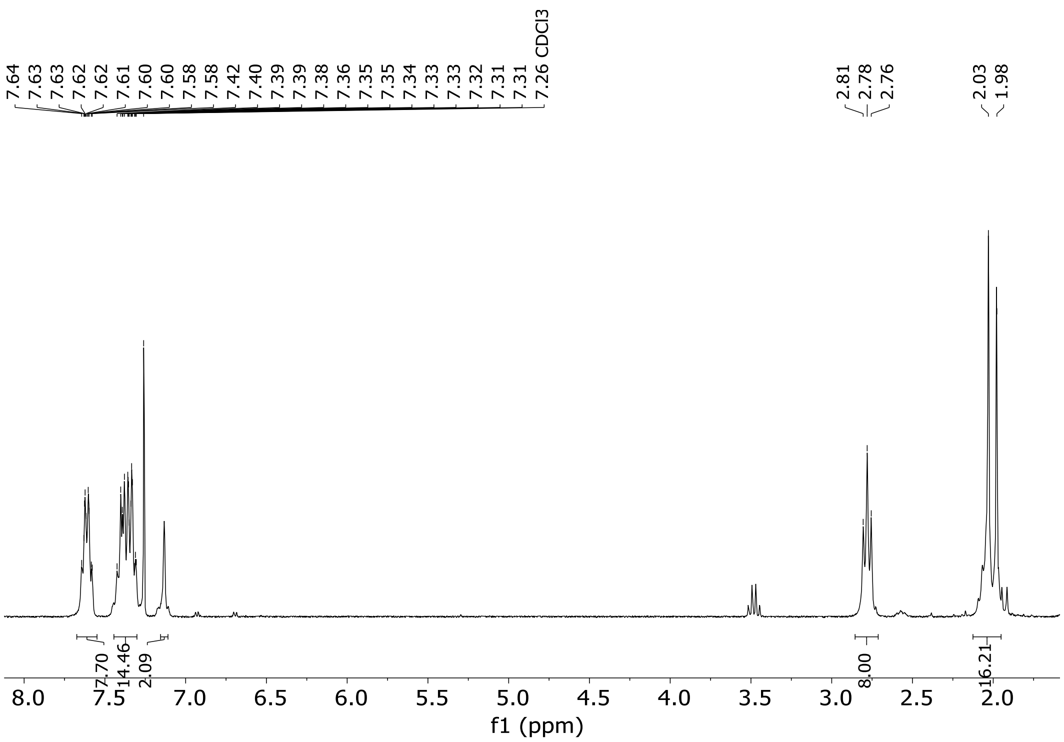

Figure S70. ^{1}H NMR spectrum (300 MHz) of [$PdCl_2$(**DTE°-C_6F_5**)$_2$] in $CDCl_3$ recorded at 25 °C.

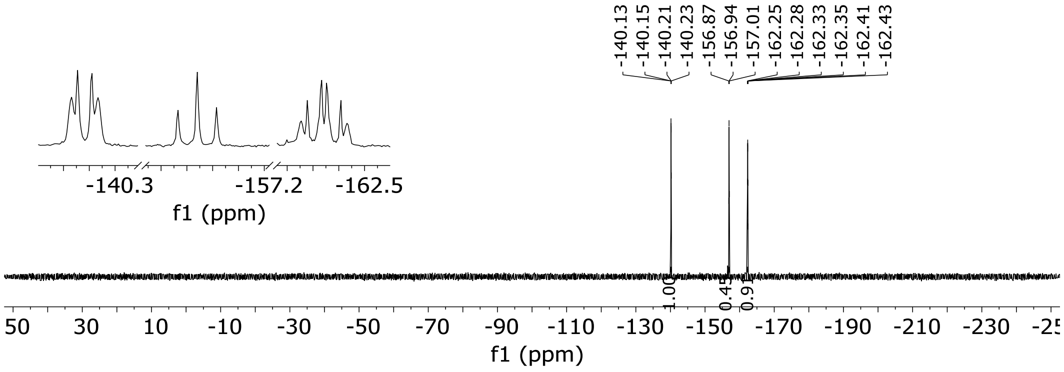

Figure S71. ^{19}F NMR spectrum (282 MHz) of [$PdCl_2$(**DTE°-C_6F_5**)$_2$] in $CDCl_3$ recorded at 25 °C.

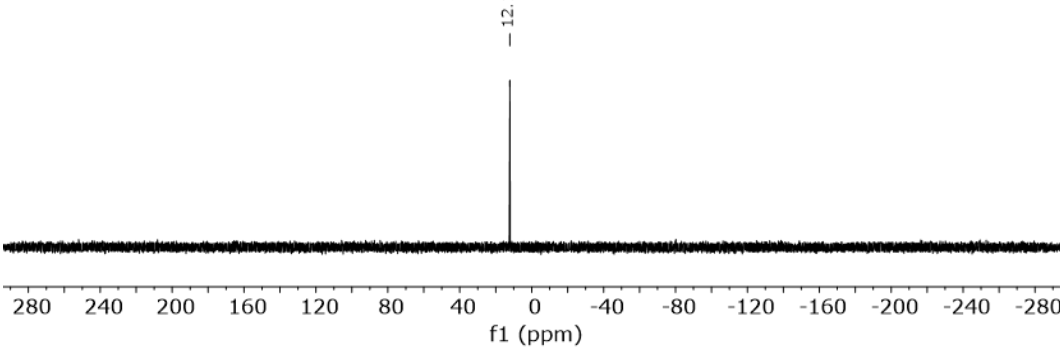

Figure S72. ^{31}P{^{1}H} NMR spectrum (122 MHz) of [$PdCl_2$(**DTE°-C_6F_5**)$_2$] in $CDCl_3$ recorded at 25 °C.

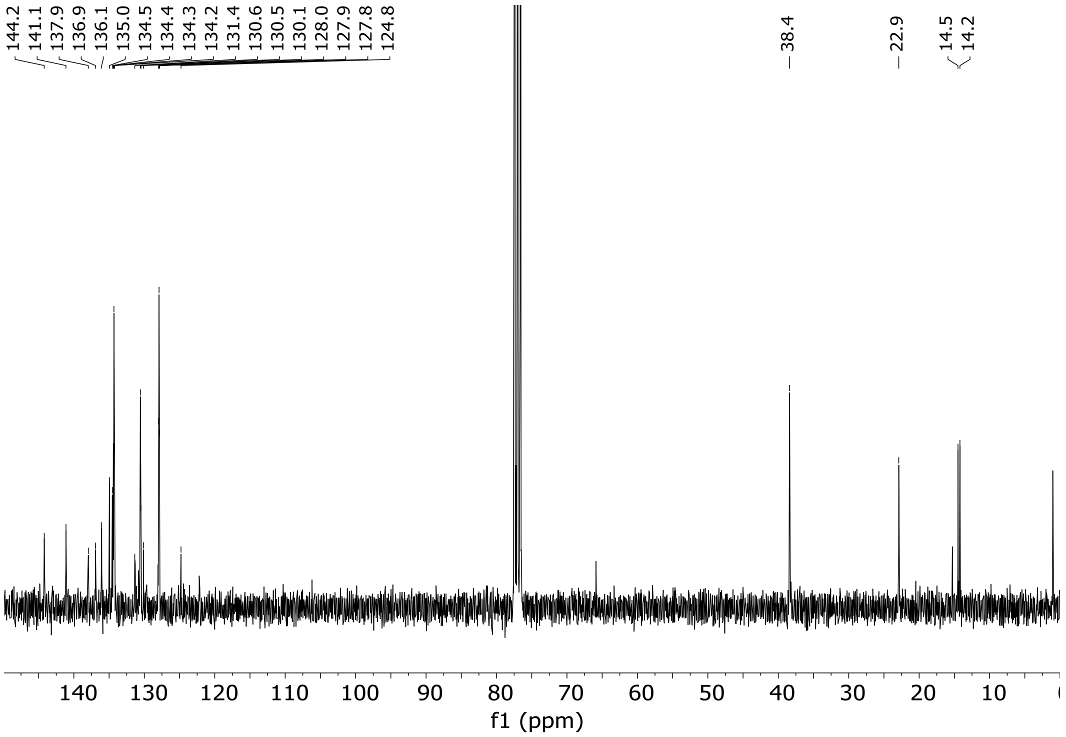

Figure S73. $^{13}C\{^{1}H\}$ NMR spectrum (75 MHz) of [PdCl$_2$(**DTE°-C$_6$F$_5$**)$_2$] in CDCl$_3$ recorded at 25 °C.

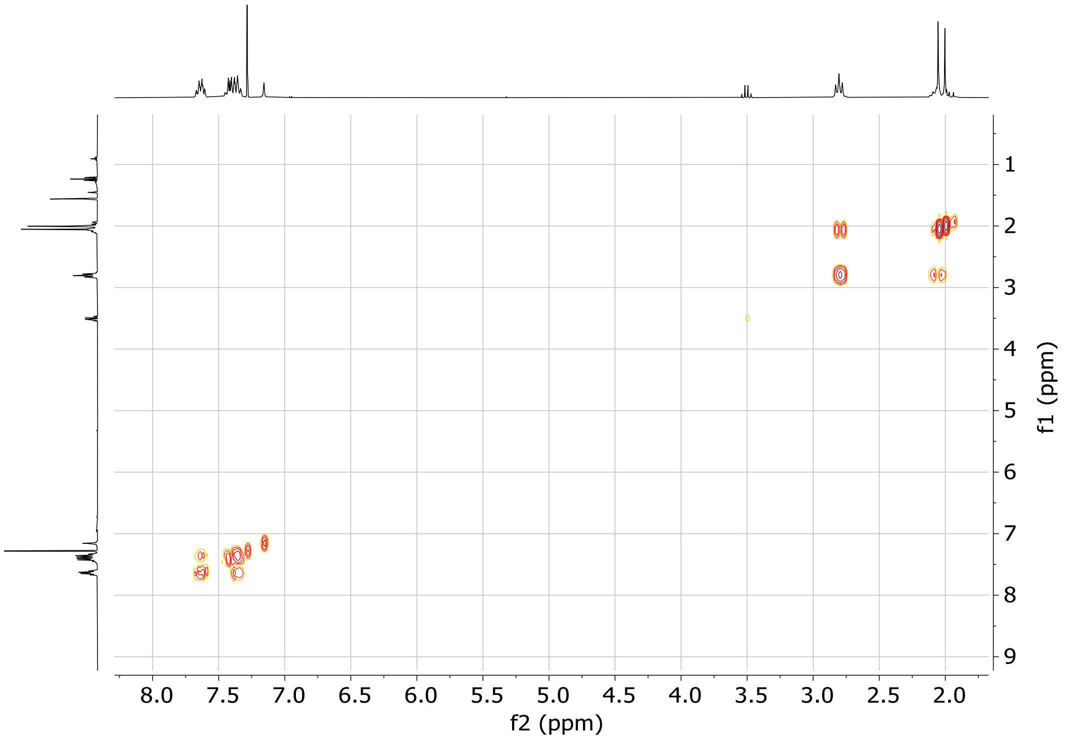

Figure S74. ^{1}H-^{1}H COSY spectrum of [PdCl$_2$(**DTE°-C$_6$F$_5$**)$_2$] in CDCl$_3$ recorded at 25 °C.

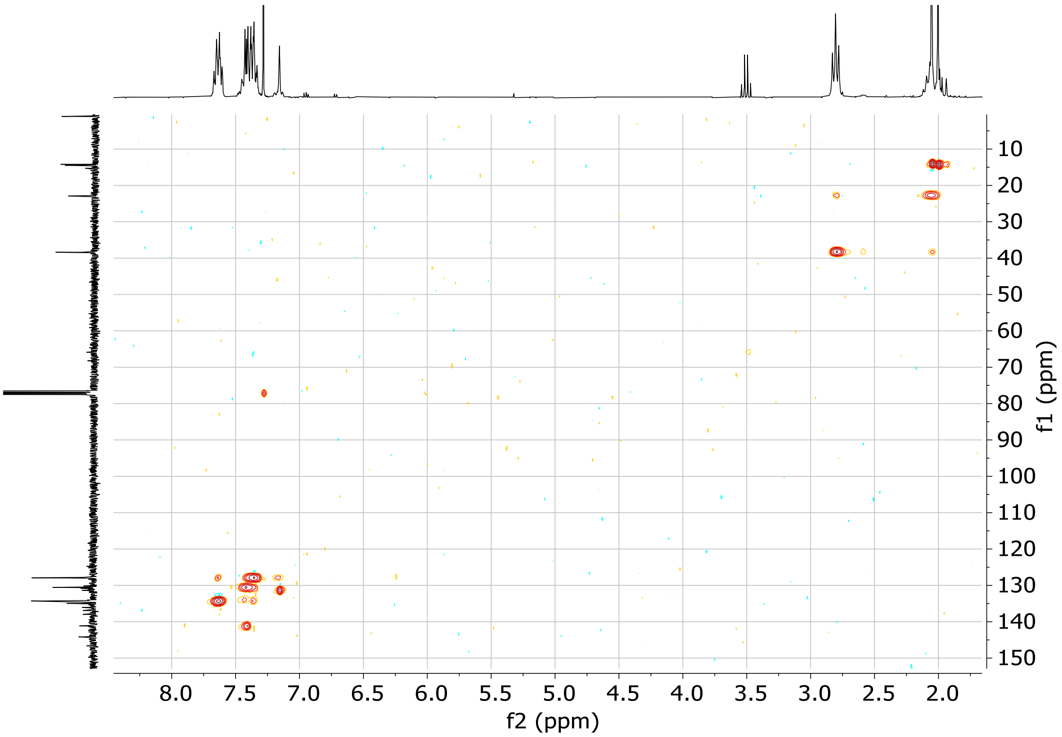

Figure S75. ^{1}H-^{13}C HSQC spectrum of [PdCl$_2$(**DTE°-C$_6$F$_5$**)$_2$] in $CDCl_3$ recorded at 25 °C.

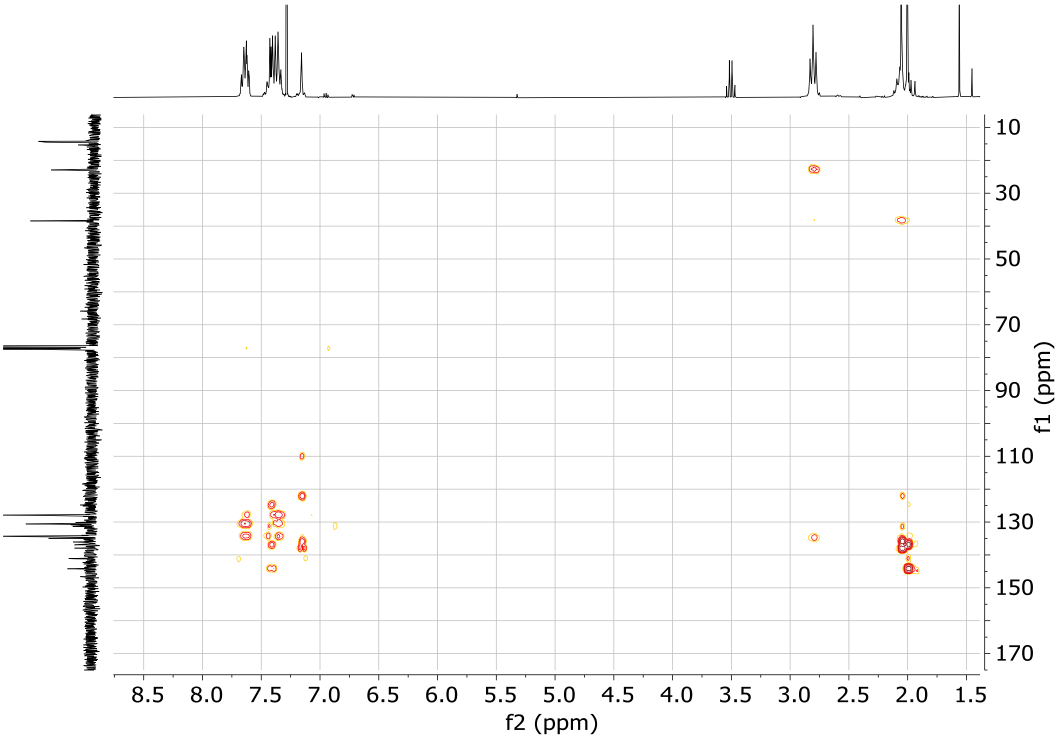

Figure S76. ^{1}H-^{13}C HMBC spectrum of [PdCl$_2$(**DTE°-C$_6$F$_5$**)$_2$] in $CDCl_3$ recorded at 25 °C.

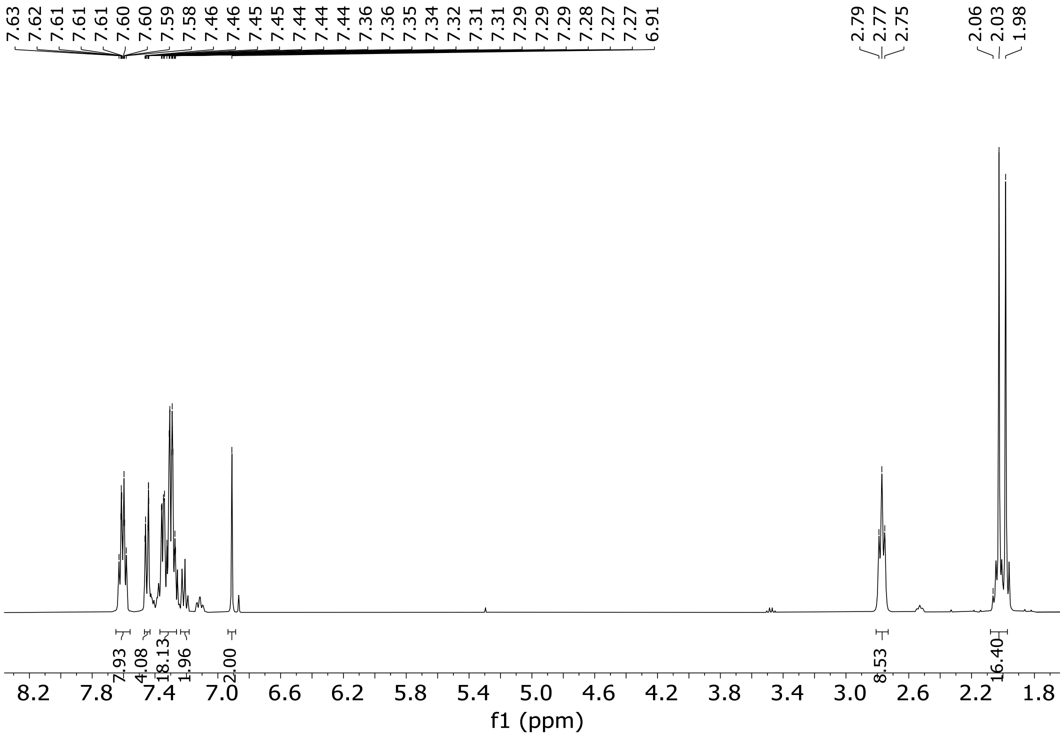

Figure S77. ^{1}H NMR spectrum (400 MHz) of [PdCl$_2$(**DTE°-Ph**)$_2$] in CDCl$_3$ recorded at 25 °C.

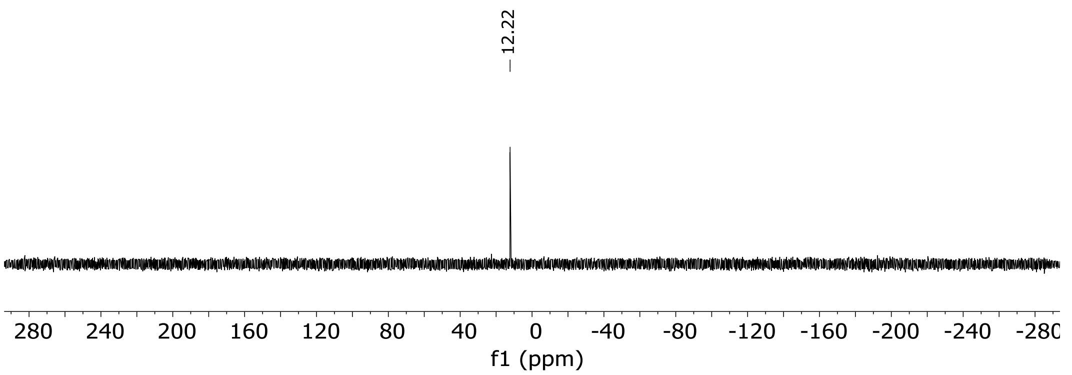

Figure S78. $^{31}P\{^{1}H\}$ NMR spectrum (122 MHz) of [PdCl$_2$(**DTE°-Ph**)$_2$] in CDCl$_3$ recorded at 25 °C.

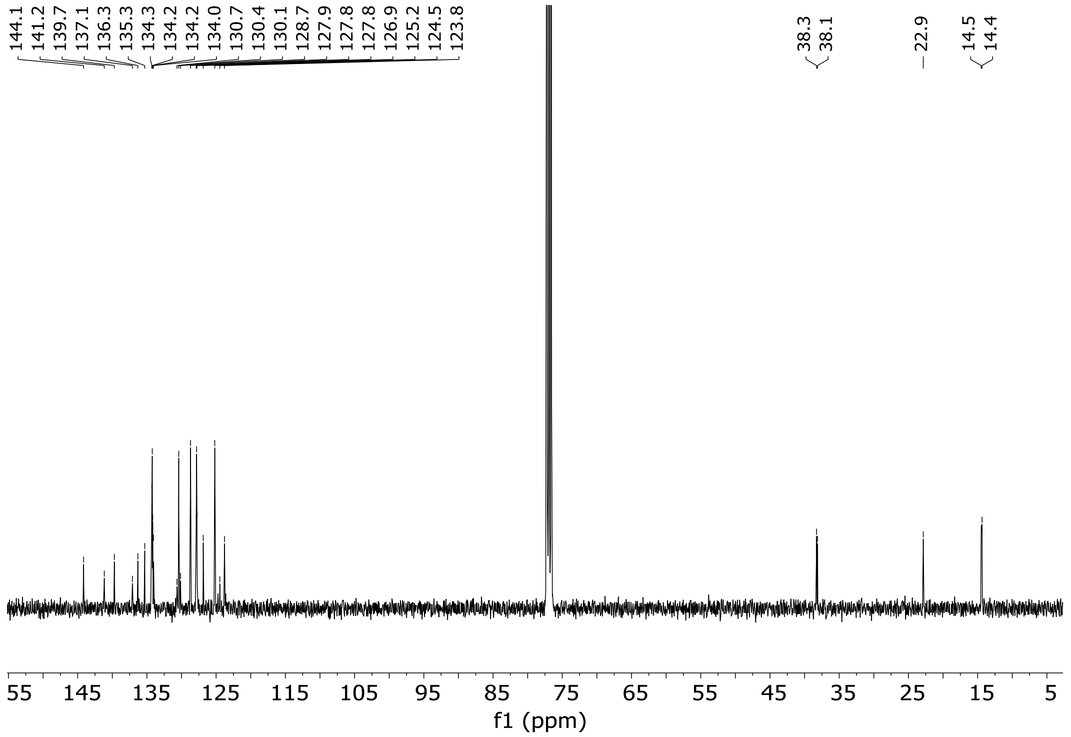

Figure S79. $^{13}C\{^{1}H\}$ NMR spectrum (101 MHz) of [PdCl$_2$(**DTE°-Ph**)$_2$] in $CDCl_3$ recorded at 25 °C.

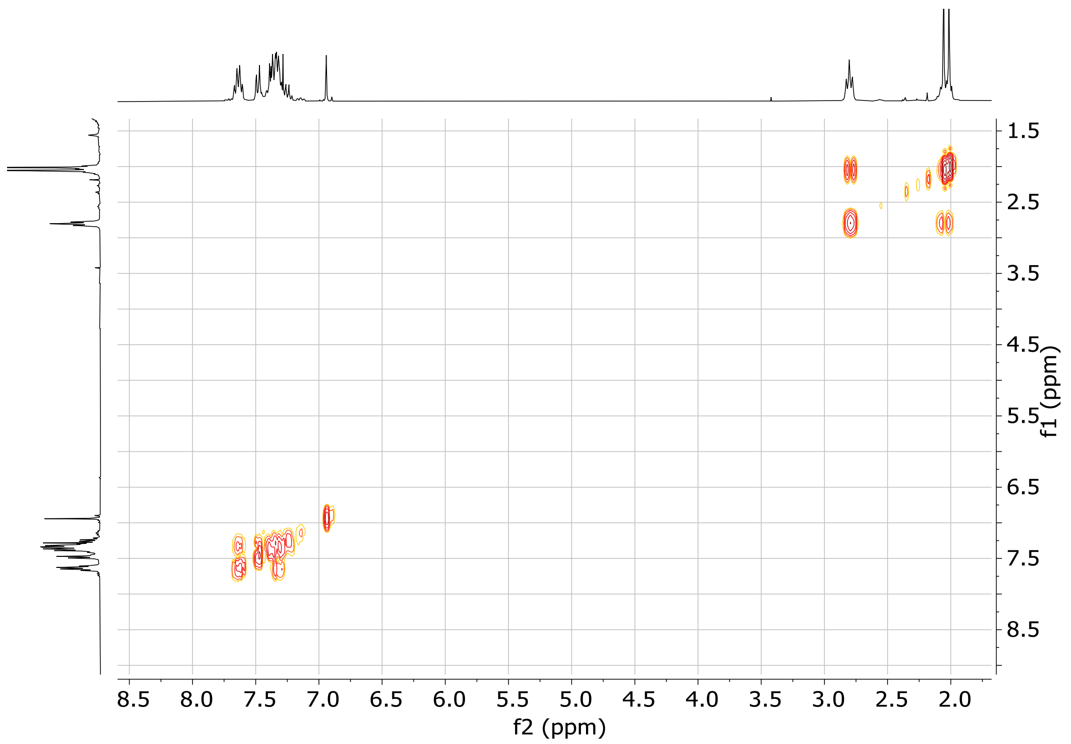

Figure S80. ^{1}H-^{1}H COSY spectrum of [PdCl$_2$(**DTE°-Ph**)$_2$] in $CDCl_3$ recorded at 25 °C.

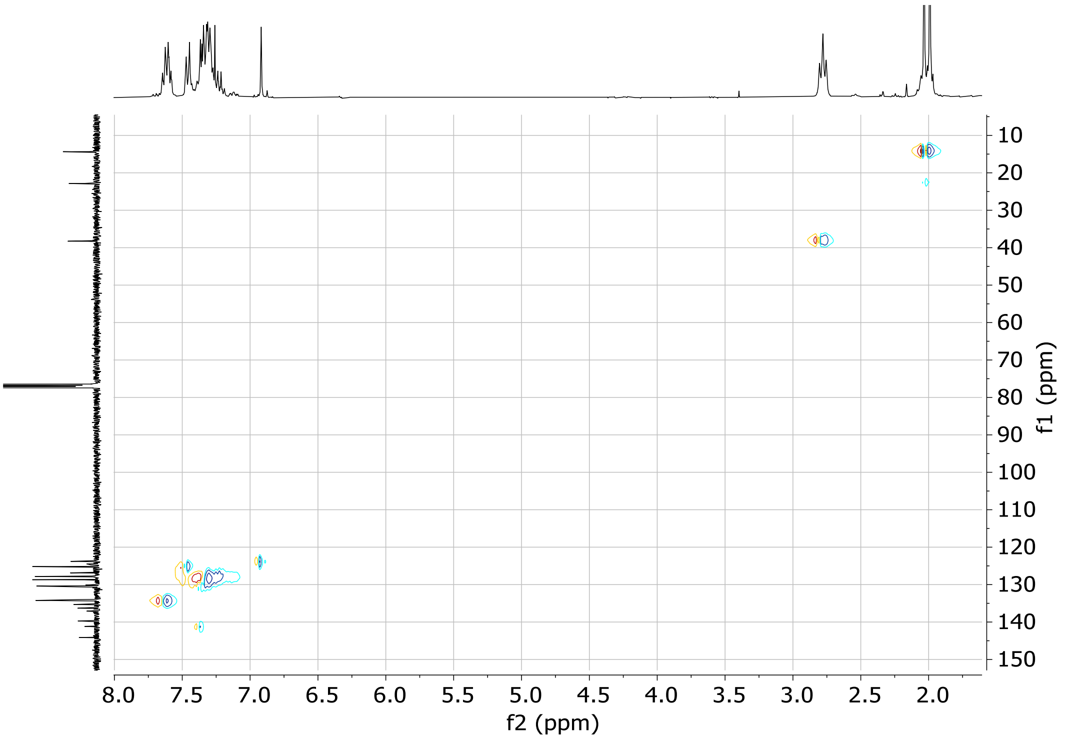

Figure S81. ^{1}H-^{13}C HSQC spectrum of [$PdCl_2$(**DTE°-Ph**)$_2$] in $CDCl_3$ recorded at 25 °C.

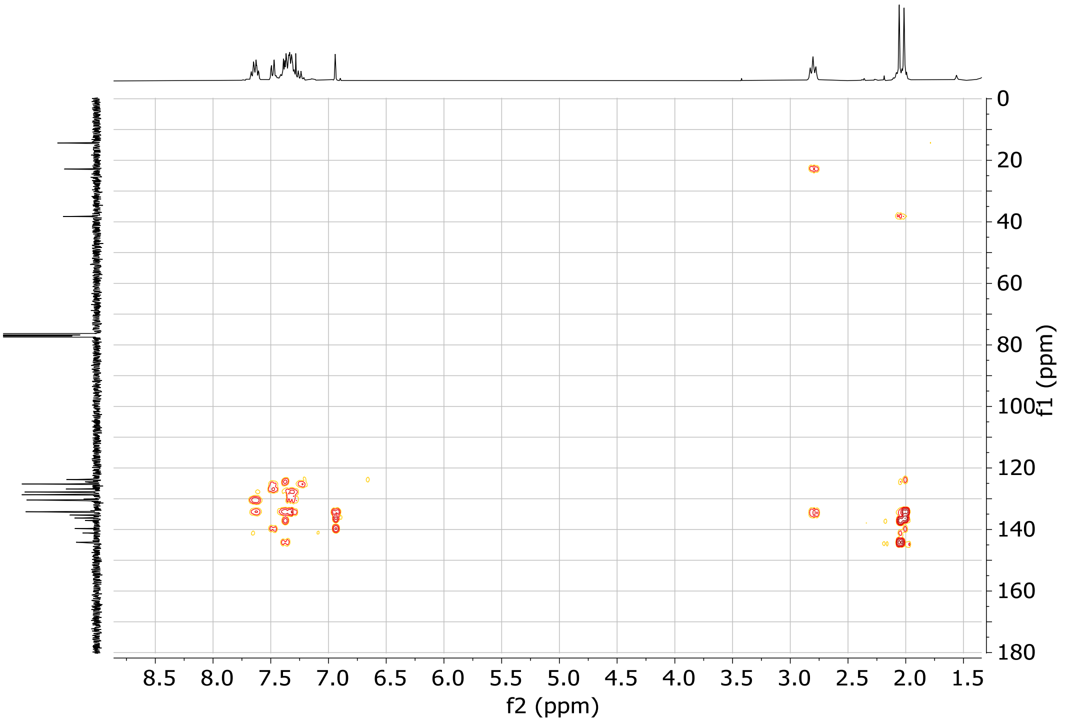

Figure S82. ^{1}H-^{13}C HMBC spectrum of [$PdCl_2$(**DTE°-Ph**)$_2$] in $CDCl_3$ recorded at 25 °C.

6 Mass spectra of novel free ligands and complexes

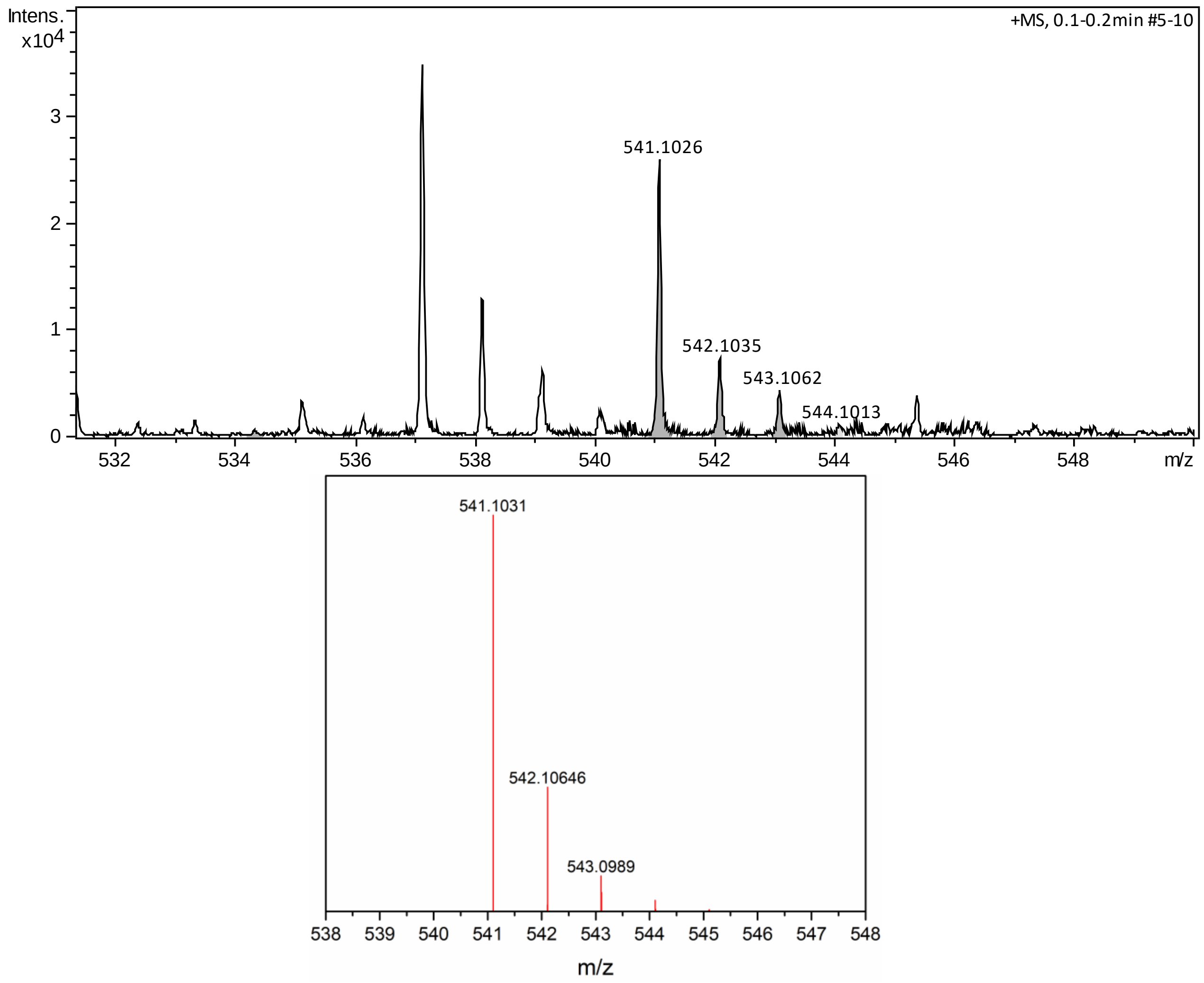

Figure S83. HRMS (ESI-TOF) spectrum of **DTE-COCF₃** in CH_3CN and simulated isotope distribution (in red) (calculated for $[M+H]^+$ 541.1031; found 541.1026).

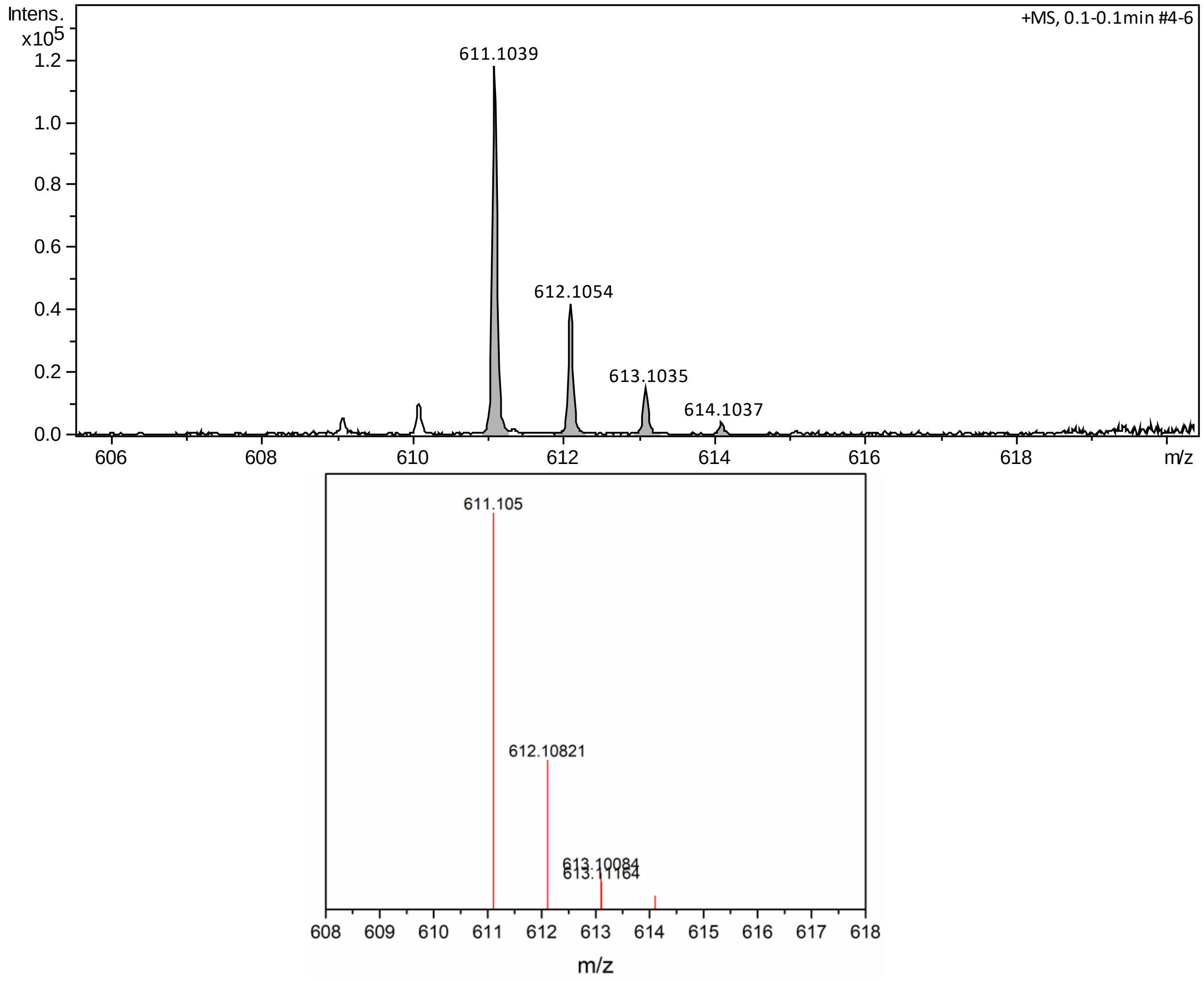

Figure S84. HRMS (ESI-TOF) spectrum of **DTE-C_6F_5** in CH_3CN and simulated isotope distribution (in red) (calculated for $[M+H]^+$ 611.1050; found 611.1039).

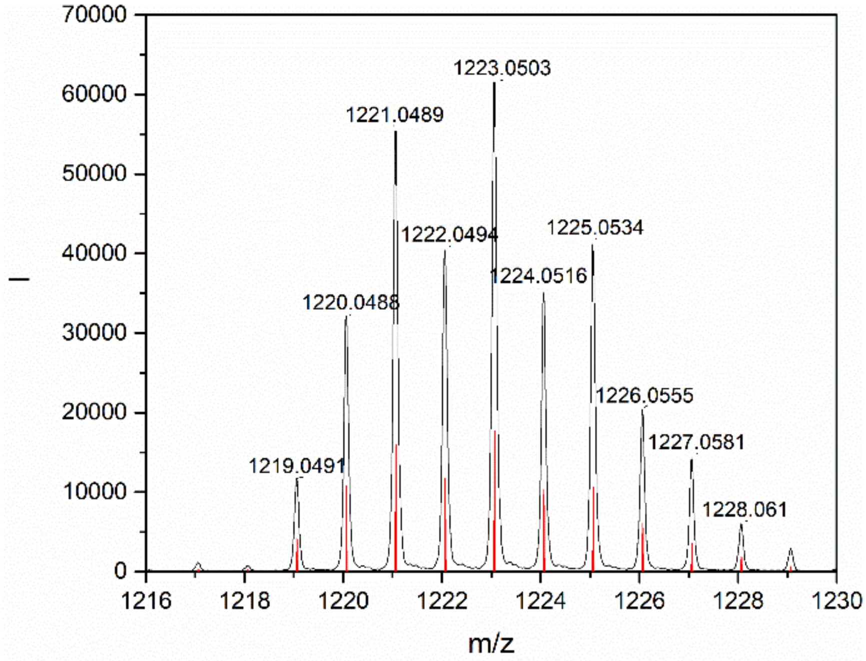

Figure S85. HRMS (ESI-TOF) spectrum of $[PdCl_2($**DTE-$COCF_3$**$)_2]$ in CH_3CN and simulated isotope distribution (in red) (calculated for $[M-Cl]^+$ 1221.0640, found 1221.0600)

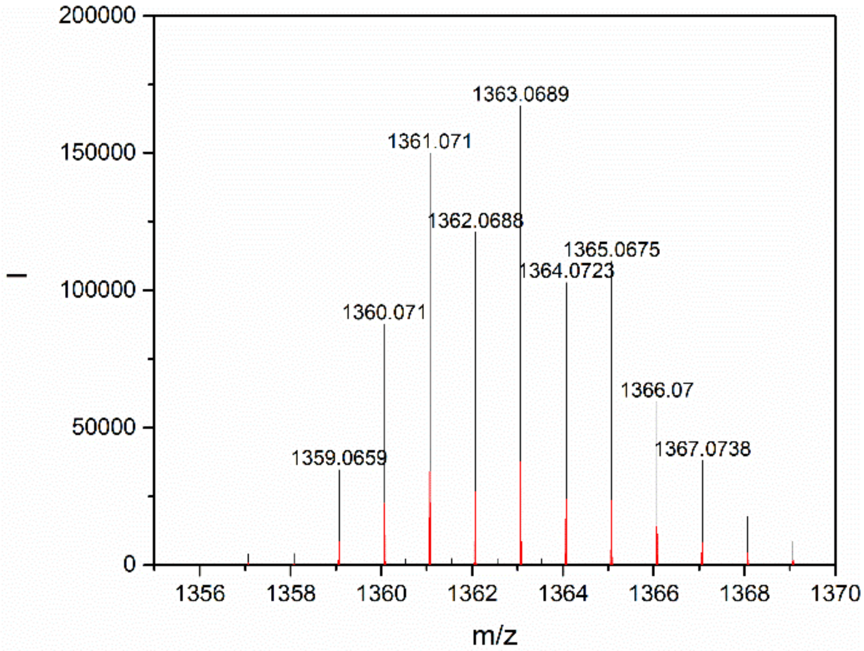

Figure S86. HRMS (ESI-TOF) spectrum of [$PdCl_2$(**DTE-C_6F_5**)$_2$] in CH_3CN and simulated isotope distribution (in red) ([M-Cl]$^+$ 1361.0678, found 1361.0710).

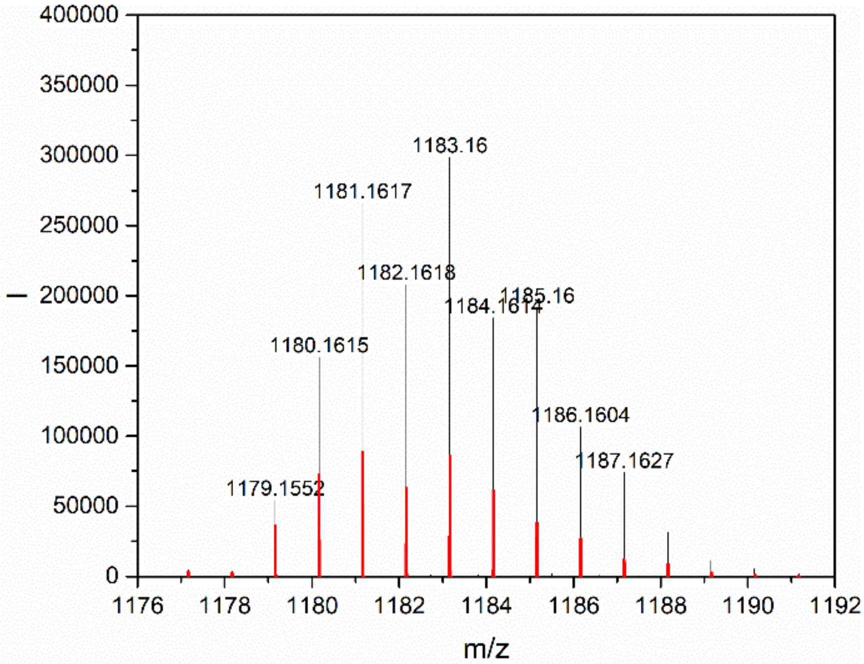

Figure S87. HRMS (ESI-TOF) spectrum of [$PdCl_2$(**DTE-Ph**)$_2$] in CH_3CN and simulated isotope distribution (in red) (calculated for [M-Cl]$^+$ 1181.1620, found 1181.1617).

7 References

(1) Coulson, D.R., Satek, L.C. and Grim, S.O. (1972). Tetrakis(triphenylphosphine)palladium(0). In *Inorganic Syntheses*, F.A. Cotton (Ed.). John Wiley & Sons, Ltd, 1972; pp 121–124. DOI: 10.1002/9780470132449.ch23.

(2) Negishi, E.; Mohan, S. trans-Dichlorobis(triphenylphosphine)palladium(II). In *Encyclopedia of Reagents for Organic Synthesis (EROS)*; John Wiley & Sons, Ltd, 2008. DOI: 10.1002/047084289X.rn00920.

(3) Tsuji, J.; Guo, H.; Ma, S.; Roman, D. S. Bis(benzonitrile)dichloropalladium(II). In *Encyclopedia of Reagents for Organic Synthesis (EROS)*; John Wiley & Sons, Ltd, 2015; pp 1–27. DOI: 10.1002/047084289X.rb101.pub3.

(4) Harris R. K; Becker E. D.; Cabral de Menezes S. M.; Goodfellow R.; Granger P. NMR Nomenclature: Nuclear Spin Properties and Conventions for Chemical Shifts: IUPAC Recommendations 2001. *Solid State Nucl. Magn. Reson.* **2002**, *22* (4), 458–483. DOI: 10.1006/snmr.2002.0063.

(5) Bianchini, G.; Strukul, G.; Wass, D. F.; Scarso, A. Photomodulable phosphines incorporating diarylethene moieties. *RSC Adv.* **2015**, *5* (14), 10795–10798. DOI: 10.1039/C4RA16127K.

(6) Sánchez, R. S.; Gras-Charles, R.; Bourdelande, J. L.; Guirado, G.; Hernando, J. Light- and Redox-Controlled Fluorescent Switch Based on a Perylenediimide–Dithienylethene Dyad. *J. Phys. Chem. C* **2012**, *116* (12), 7164–7172. DOI: 10.1021/jp300815p.

(7) Kobatake, S.; Uchida, K.; Tsuchida, E.; Irie, M. Single-crystalline photochromism of diarylethenes: reactivity-structure relationship. *Chem. Commun.* **2002** (23), 2804–2805. DOI: 10.1039/b208419h.

8 Cartesian coordinates

DTE^{o}-COCF$_3$

C	2.759474000	1.146499000	-0.626776000
C	2.330436000	-0.124591000	-1.002367000
S	3.456555000	-1.353428000	-0.519939000
C	4.540454000	-0.165924000	0.183190000
C	4.021628000	1.103704000	0.026042000
H	4.541023000	1.980872000	0.393614000
C	1.085029000	-0.516156000	-1.731234000
H	0.302608000	-0.814495000	-1.024929000
H	0.707569000	0.332691000	-2.305344000
H	1.261471000	-1.353653000	-2.413029000
C	1.735614000	4.568237000	-1.895034000
C	0.467114000	4.150027000	-1.123079000
C	0.749118000	2.712145000	-0.716607000
C	2.059692000	2.413509000	-0.884483000
C	2.839086000	3.629906000	-1.362331000
H	1.979148000	5.627741000	-1.776803000
H	-0.448903000	4.249424000	-1.716173000
H	0.316682000	4.757876000	-0.218698000
H	3.590682000	3.376159000	-2.117871000
H	3.381119000	4.082000000	-0.518352000
H	1.587967000	4.378331000	-2.964058000
C	-1.615689000	1.775484000	-0.764429000
C	-2.492160000	0.939035000	-0.121255000
S	-1.726874000	0.270930000	1.311848000
C	-0.235588000	1.126519000	1.009245000
C	-0.319648000	1.875814000	-0.150591000
H	-1.875433000	2.300345000	-1.677975000
C	0.902001000	1.008022000	1.977158000
H	1.491569000	0.101556000	1.796258000
H	1.573392000	1.863586000	1.872605000
H	0.545611000	0.971762000	3.011587000
P	-4.166195000	0.535141000	-0.726120000
C	-3.932337000	-1.165456000	-1.423994000
C	-5.045102000	-2.012456000	-1.568581000
C	-2.692776000	-1.590437000	-1.924753000
C	-4.912400000	-3.261460000	-2.176732000
C	-2.562801000	-2.838831000	-2.537727000
C	-3.670077000	-3.680461000	-2.662089000
H	-6.016487000	-1.702822000	-1.191748000
H	-1.825520000	-0.944350000	-1.836283000
H	-5.780515000	-3.908377000	-2.270096000
H	-1.592544000	-3.153535000	-2.912944000
H	-3.567892000	-4.653239000	-3.134769000
C	-5.069994000	0.197747000	0.852621000
C	-4.929200000	-0.987593000	1.594598000
C	-5.912756000	1.208078000	1.341864000
C	-5.609917000	-1.151012000	2.801916000
C	-6.590816000	1.044949000	2.552220000
C	-6.440235000	-0.134873000	3.283917000
H	-4.293419000	-1.786739000	1.226294000
H	-6.039755000	2.126399000	0.773991000
H	-5.491402000	-2.071828000	3.366396000
H	-7.239362000	1.835762000	2.918805000
H	-6.970361000	-0.265065000	4.223272000
C	5.804271000	-0.445722000	0.845731000
O	6.541197000	0.414126000	1.303171000
C	6.229047000	-1.931511000	0.984987000
F	6.318474000	-2.522273000	-0.228992000
F	7.412034000	-2.044746000	1.593510000
F	5.314202000	-2.622947000	1.702546000

DTE^{c}-COCF$_3$

C	-2.636506000	0.868282000	-0.317549000
C	-1.485089000	0.012566000	0.248517000
S	-2.265034000	-1.668849000	0.562527000
C	-3.896377000	-0.953212000	0.455368000
C	-3.916580000	0.333747000	-0.022354000
H	-4.831023000	0.886230000	-0.202583000
C	-2.289638000	4.368544000	-1.447604000
C	-0.899204000	3.789581000	-1.807754000
C	-0.962742000	2.370924000	-1.284535000
C	-2.333316000	2.028555000	-0.974453000
C	-3.237904000	3.144035000	-1.435361000
H	-2.617309000	5.148035000	-2.140092000
H	-0.071968000	4.365690000	-1.381373000
H	-0.753081000	3.775769000	-2.896435000
H	-4.118806000	3.277977000	-0.801450000
H	-3.594309000	2.927326000	-2.452373000
H	-2.253933000	4.805158000	-0.443270000
C	1.448426000	1.635979000	-1.268778000
C	2.210845000	0.606650000	-0.799063000
S	1.269132000	-0.750240000	-0.150381000
C	-0.333722000	0.001032000	-0.780546000
C	0.034818000	1.456037000	-1.122619000
H	1.882220000	2.544575000	-1.673765000
P	4.046675000	0.679307000	-0.777288000
C	4.378638000	0.984835000	1.018936000
C	5.688928000	0.787286000	1.489874000
C	3.412689000	1.492540000	1.900272000
C	6.017280000	1.071405000	2.815349000
C	3.746368000	1.782238000	3.225906000
C	5.046116000	1.569979000	3.688739000
H	6.453145000	0.397170000	0.822155000
H	2.396287000	1.661043000	1.558849000
H	7.032242000	0.904467000	3.165412000
H	2.984688000	2.169200000	3.897396000
H	5.302224000	1.792394000	4.720721000
C	4.487650000	-1.106304000	-0.970002000
C	4.485622000	-2.033865000	0.085037000
C	4.807585000	-1.547227000	-2.263833000
C	4.791082000	-3.374334000	-0.154319000
C	5.106598000	-2.890384000	-2.502607000
C	5.099508000	-3.805555000	-1.447767000
H	4.248678000	-1.710139000	1.093684000
H	4.822878000	-0.838422000	-3.088199000
H	4.784978000	-4.082844000	0.669397000
H	5.350494000	-3.218749000	-3.509093000
H	5.336623000	-4.849827000	-1.630975000
C	-0.682341000	-0.719738000	-2.104552000
H	-1.587232000	-0.287866000	-2.541981000
H	0.139958000	-0.592572000	-2.813042000
H	-0.847174000	-1.786926000	-1.947999000
C	-1.081048000	0.604006000	1.619485000
H	-0.678009000	1.613370000	1.494551000

H	-1.963091000	0.663831000	2.261753000
H	-0.330122000	-0.012413000	2.117173000
C	-5.028743000	-1.775774000	0.819537000
O	-4.950382000	-2.932555000	1.215979000
C	-6.434369000	-1.134151000	0.678429000
F	-6.679431000	-0.774979000	-0.603498000
F	-7.392360000	-1.985118000	1.060389000
F	-6.541368000	-0.015711000	1.432909000

DTE^{O}-C_6F_5

C	1.710839000	1.317954000	-0.785383000
C	1.265803000	0.137817000	-1.352811000
S	2.451436000	-1.127434000	-1.186306000
C	3.588114000	-0.057491000	-0.373907000
C	3.036951000	1.196875000	-0.256498000
H	3.550428000	2.021536000	0.218037000
C	-0.035720000	-0.158488000	-2.029869000
H	-0.744688000	-0.621255000	-1.333981000
H	-0.485726000	0.768972000	-2.391153000
H	0.093791000	-0.837300000	-2.878991000
C	0.548035000	4.892410000	-1.276425000
C	-0.647188000	4.301190000	-0.500762000
C	-0.316361000	2.819713000	-0.402592000
C	0.978487000	2.591382000	-0.730579000
C	1.701076000	3.898621000	-1.024120000
H	0.789318000	5.916330000	-0.977137000
H	-1.609600000	4.488514000	-0.990499000
H	-0.729526000	4.722965000	0.512012000
H	2.391446000	3.815318000	-1.870712000
H	2.305837000	4.194723000	-0.153826000
H	0.313791000	4.905891000	-2.346846000
C	-2.672361000	1.859242000	-0.428146000
C	-3.486658000	0.897759000	0.114490000
S	-2.595456000	-0.019137000	1.318825000
C	-1.138937000	0.907982000	1.058747000
C	-1.327063000	1.865658000	0.078349000
H	-3.014216000	2.544527000	-1.196855000
C	0.082755000	0.623054000	1.877452000
H	0.661653000	-0.207816000	1.457491000
H	0.734268000	1.500045000	1.894509000
H	-0.176677000	0.362740000	2.908681000
P	-5.205976000	0.585876000	-0.409737000
C	-5.037497000	-0.948440000	-1.434794000
C	-6.171196000	-1.746741000	-1.668415000
C	-3.838156000	-1.275473000	-2.084611000
C	-6.097598000	-2.855277000	-2.512626000
C	-3.767614000	-2.382478000	-2.933816000
C	-4.894844000	-3.178033000	-3.148286000
H	-7.112254000	-1.511092000	-1.177943000
H	-2.955598000	-0.664146000	-1.927828000
H	-6.980847000	-3.467422000	-2.673938000
H	-2.827620000	-2.623318000	-3.423352000
H	-4.839056000	-4.040970000	-3.805900000
C	-5.966636000	-0.058318000	1.149297000
C	-5.781544000	-1.367550000	1.625294000
C	-6.740077000	0.835785000	1.906221000
C	-6.351072000	-1.766873000	2.835335000
C	-7.305896000	0.436430000	3.119197000
C	-7.112179000	-0.865636000	3.585433000
H	-5.198218000	-2.077875000	1.047518000
H	-6.900565000	1.848855000	1.545710000
H	-6.199691000	-2.781741000	3.192791000
H	-7.901473000	1.139540000	3.694827000
H	-7.555742000	-1.179179000	4.526359000
C	4.924252000	-0.489864000	0.042308000
C	5.989154000	0.421332000	0.173269000
C	5.241504000	-1.827096000	0.336644000
C	7.262783000	0.035641000	0.573873000
C	6.510474000	-2.234928000	0.730177000
C	7.531522000	-1.299817000	0.854356000
F	5.812664000	1.725977000	-0.100529000
F	8.238389000	0.948414000	0.678735000
F	8.755813000	-1.678721000	1.236308000
F	6.748535000	-3.525844000	0.998352000
F	4.298602000	-2.783037000	0.240021000

DTE^{c}-C_6F_5

C	-1.594303000	1.803909000	0.135557000
C	-0.660430000	0.599183000	0.354182000
S	-1.737173000	-0.890394000	-0.017770000
C	-3.221817000	0.098229000	0.137904000
C	-2.976222000	1.438448000	0.192792000
H	-3.768162000	2.175637000	0.239872000
C	-0.557329000	5.326493000	0.456245000
C	0.741465000	4.672671000	-0.076251000
C	0.396871000	3.200244000	-0.141143000
C	-1.042566000	3.036984000	-0.018754000
C	-1.696402000	4.398630000	-0.033804000
H	-0.679183000	6.360388000	0.122235000
H	1.615420000	4.882162000	0.548629000
H	0.973688000	5.040561000	-1.085252000
H	-2.597175000	4.453618000	0.584467000
H	-1.986925000	4.663460000	-1.060458000
H	-0.541141000	5.328111000	1.552131000
C	2.642224000	2.071869000	-0.369369000
C	3.178756000	0.821199000	-0.320122000
S	1.954808000	-0.475059000	-0.219957000
C	0.568172000	0.745170000	-0.570350000
C	1.207927000	2.125075000	-0.322584000
H	3.256325000	2.966501000	-0.398661000
P	4.987948000	0.533344000	-0.299825000
C	5.219765000	-0.265126000	1.354698000
C	6.340385000	-1.084657000	1.576027000
C	4.375712000	0.029976000	2.437082000
C	6.596567000	-1.611466000	2.842615000
C	4.636353000	-0.496031000	3.704470000
C	5.744517000	-1.320169000	3.911563000
H	7.008953000	-1.324312000	0.753377000
H	3.508146000	0.666685000	2.292194000
H	7.462134000	-2.250889000	2.993384000
H	3.967480000	-0.264016000	4.528883000
H	5.943703000	-1.731587000	4.897079000
C	5.159843000	-0.889261000	-1.470074000
C	4.824998000	-2.216242000	-1.151531000
C	5.620554000	-0.597575000	-2.763340000
C	4.944790000	-3.224398000	-2.108443000
C	5.735018000	-1.606765000	-3.722847000
C	5.397720000	-2.921602000	-3.396231000
H	4.478408000	-2.465021000	-0.153409000
H	5.891726000	0.423130000	-3.021695000

H	4.682114000	-4.246431000	-1.849545000
H	6.092417000	-1.366056000	-4.720206000
H	5.490398000	-3.708720000	-4.139416000
C	0.232971000	0.641154000	-2.076102000
H	-0.538105000	1.370091000	-2.342773000
H	1.131365000	0.856501000	-2.660112000
H	-0.123788000	-0.355522000	-2.341979000
C	-0.301658000	0.545411000	1.857939000
H	0.284424000	1.426294000	2.135751000
H	-1.221097000	0.541514000	2.448777000
H	0.275148000	-0.348208000	2.102719000
C	-4.550389000	-0.523778000	0.113880000
C	-5.633375000	0.099262000	-0.535001000
C	-4.830274000	-1.766832000	0.707482000
C	-6.901973000	-0.464830000	-0.582289000
C	-6.092010000	-2.349809000	0.664383000
C	-7.136274000	-1.697905000	0.018335000
F	-5.470559000	1.279391000	-1.155794000
F	-7.898462000	0.168443000	-1.215798000
F	-8.354123000	-2.247062000	-0.021963000
F	-6.307331000	-3.532374000	1.255759000
F	-3.872323000	-2.440793000	1.363737000

DTE^{o}-Ph

C	2.812527000	0.855769000	-0.761248000
C	2.309136000	-0.377667000	-1.129013000
S	3.409687000	-1.658617000	-0.678020000
C	4.570415000	-0.529144000	0.004769000
C	4.103049000	0.752507000	-0.137288000
H	4.644068000	1.616086000	0.233020000
C	1.024433000	-0.716272000	-1.818553000
H	0.254466000	-1.013755000	-1.097279000
H	0.649988000	0.156062000	-2.359213000
H	1.151308000	-1.538048000	-2.531275000
C	1.917961000	4.341767000	-1.953495000
C	0.653194000	3.975795000	-1.149703000
C	0.877329000	2.520256000	-0.768590000
C	2.166620000	2.158362000	-0.977611000
C	2.988323000	3.344673000	-1.462388000
H	2.215598000	5.387297000	-1.832379000
H	-0.272793000	4.128285000	-1.715851000
H	0.558222000	4.580663000	-0.235512000
H	3.707273000	3.066437000	-2.240871000
H	3.573495000	3.760029000	-0.628181000
H	1.730901000	4.171277000	-3.019746000
C	-1.535280000	1.710461000	-0.752434000
C	-2.435976000	0.913790000	-0.091904000
S	-1.662683000	0.182952000	1.305629000
C	-0.137013000	0.960634000	0.964774000
C	-0.216434000	1.731907000	-0.180771000
H	-1.795235000	2.261123000	-1.650639000
C	1.025920000	0.757770000	1.886624000
H	1.567891000	-0.165203000	1.649854000
H	1.732211000	1.585226000	1.785175000
H	0.705057000	0.700200000	2.931709000
P	-4.145696000	0.607735000	-0.648980000
C	-4.033787000	-1.107418000	-1.341153000
C	-5.200331000	-1.883387000	-1.458565000
C	-2.833768000	-1.612217000	-1.863897000
C	-5.159140000	-3.141077000	-2.061826000
C	-2.795423000	-2.869151000	-2.472177000
C	-3.955807000	-3.639737000	-2.569689000
H	-6.142363000	-1.511347000	-1.064154000
H	-1.925301000	-1.022670000	-1.796268000
H	-6.068097000	-3.732236000	-2.134028000
H	-1.854583000	-3.245821000	-2.864732000
H	-3.924925000	-4.619093000	-3.038850000
C	-5.027489000	0.335253000	0.955109000
C	-4.948653000	-0.855233000	1.697941000
C	-5.785791000	1.401985000	1.462580000
C	-5.606878000	-0.969101000	2.923338000
C	-6.441091000	1.288415000	2.690986000
C	-6.352590000	0.102534000	3.423067000
H	-4.378770000	-1.696705000	1.316207000
H	-5.864672000	2.325676000	0.894630000
H	-5.536948000	-1.894682000	3.488175000
H	-7.024008000	2.122659000	3.071341000
H	-6.865621000	0.010899000	4.376372000
C	5.832840000	-0.977663000	0.605914000
C	6.950937000	-0.120506000	0.619147000
C	5.965098000	-2.254036000	1.184550000
C	8.150935000	-0.523385000	1.201767000
C	7.171329000	-2.658241000	1.755568000
C	8.269417000	-1.794780000	1.771102000
H	6.880629000	0.857670000	0.153392000
H	5.113732000	-2.929019000	1.204816000
H	9.000727000	0.153762000	1.199773000
H	7.249379000	-3.647875000	2.197076000
H	9.207679000	-2.109436000	2.218804000

DTE^{c}-Ph

C	-2.709941000	1.312333000	-0.134644000
C	-1.646772000	0.269485000	0.263220000
S	-2.537282000	-1.372071000	0.100540000
C	-4.130630000	-0.558809000	0.120174000
C	-4.040200000	0.795714000	-0.016205000
H	-4.912383000	1.440122000	-0.045739000
C	-2.084986000	4.941673000	-0.303027000
C	-0.709196000	4.374351000	-0.730541000
C	-0.882822000	2.877174000	-0.589729000
C	-2.297067000	2.567351000	-0.457744000
C	-3.100661000	3.830304000	-0.666048000
H	-2.314834000	5.899321000	-0.778165000
H	0.121824000	4.768030000	-0.136614000
H	-0.498315000	4.622658000	-1.780036000
H	-4.013885000	3.866955000	-0.064534000
H	-3.399748000	3.918857000	-1.720387000
H	-2.093343000	5.095885000	0.782083000
C	1.483694000	1.999776000	-0.630933000
C	2.159571000	0.840391000	-0.399334000
S	1.087450000	-0.563696000	-0.127033000
C	-0.419768000	0.425004000	-0.661998000
C	0.052034000	1.890736000	-0.608070000
H	1.991952000	2.947231000	-0.781312000
P	3.986860000	0.770968000	-0.323123000
C	4.271775000	0.219209000	1.421622000
C	5.475418000	-0.425740000	1.756053000
C	3.372157000	0.542307000	2.449821000
C	5.760195000	-0.757270000	3.081335000
C	3.661103000	0.212411000	3.775996000

C	4.853051000	-0.440785000	4.096447000
H	6.188187000	-0.683278000	0.977141000
H	2.439798000	1.047820000	2.216956000
H	6.691146000	-1.264468000	3.320051000
H	2.949094000	0.462584000	4.557886000
H	5.074590000	-0.700115000	5.127892000
C	4.353691000	-0.758585000	-1.298523000
C	4.167366000	-2.064716000	-0.814835000
C	4.810912000	-0.581614000	-2.613664000
C	4.428764000	-3.165079000	-1.631714000
C	5.067227000	-1.683863000	-3.433183000
C	4.876839000	-2.977146000	-2.942866000
H	3.826064000	-2.223659000	0.203265000
H	4.968093000	0.422702000	-2.999357000
H	4.280324000	-4.169784000	-1.245512000
H	5.420220000	-1.531334000	-4.449339000
H	5.080116000	-3.835834000	-3.576737000
C	-0.702870000	0.070290000	-2.139807000
H	-1.544995000	0.659202000	-2.515426000
H	0.179733000	0.304045000	-2.740673000
H	-0.936738000	-0.988700000	-2.263500000
C	-1.321530000	0.475474000	1.761399000
H	-0.846333000	1.449209000	1.912033000
H	-2.249018000	0.449804000	2.338957000
H	-0.652296000	-0.299986000	2.138280000
C	-5.359119000	-1.352401000	0.241871000
C	-6.578795000	-0.857213000	-0.264703000
C	-5.358798000	-2.619016000	0.857816000
C	-7.752950000	-1.594901000	-0.141708000
C	-6.536787000	-3.355778000	0.976209000
C	-7.739331000	-2.847941000	0.480117000
H	-6.598659000	0.099591000	-0.776488000
H	-4.435715000	-3.020263000	1.265044000
H	-8.680518000	-1.196004000	-0.542674000
H	-6.513526000	-4.327800000	1.460682000
H	-8.655870000	-3.423798000	0.570125000

[PdCl$_2$(**DTE^{o}-COCF$_3$**)$_2$]

Pd	-0.061203000	-1.184500000	0.659183000
Cl	-0.642077000	-1.189271000	2.950424000
S	-4.926600000	-2.531676000	0.839954000
S	-8.061580000	0.660969000	-2.682137000
P	-1.795047000	-2.768581000	0.303599000
F	-12.498098000	2.776474000	-1.128824000
F	-10.695421000	3.966002000	-0.846071000
F	-11.079200000	2.246956000	0.436198000
O	-11.063608000	1.273202000	-2.738561000
C	-1.755246000	-4.298367000	1.308326000
C	-0.660572000	-4.551012000	2.146810000
H	0.140074000	-3.824384000	2.225011000
C	-0.605642000	-5.731713000	2.890848000
H	0.245895000	-5.918545000	3.538608000
C	-1.640869000	-6.663927000	2.804869000
H	-1.598823000	-7.579476000	3.387957000
C	-2.728832000	-6.423750000	1.960193000
H	-3.530713000	-7.151855000	1.880157000
C	-2.783229000	-5.251822000	1.207304000
H	-3.614229000	-5.097943000	0.525660000
C	-2.052433000	-3.370193000	-1.410400000
C	-1.507391000	-4.590411000	-1.835121000
H	-0.966455000	-5.222119000	-1.137461000
C	-1.670234000	-5.006212000	-3.158025000
H	-1.250527000	-5.955948000	-3.477031000
C	-2.372131000	-4.208947000	-4.064908000
H	-2.500821000	-4.537335000	-5.092311000
C	-2.906711000	-2.986863000	-3.647612000
H	-3.450762000	-2.359717000	-4.348228000
C	-2.742625000	-2.565522000	-2.328929000
H	-3.150992000	-1.611581000	-2.012671000
C	-3.317841000	-1.856396000	0.674979000
C	-3.378745000	-0.487683000	0.736957000
H	-2.500749000	0.139989000	0.659173000
C	-4.697643000	0.046418000	0.896585000
C	-5.653559000	-0.948953000	0.988411000
C	-7.129379000	-0.825027000	1.209623000
H	-7.357243000	0.146634000	1.654004000
H	-7.503206000	-1.607893000	1.876874000
H	-7.682873000	-0.896948000	0.266116000
C	-4.926611000	1.497515000	0.958795000
H	-4.342404000	2.162544000	2.919685000
C	-4.454117000	3.809155000	1.451291000
H	-3.726081000	4.165812000	0.715387000
C	-5.838064000	3.679377000	0.777545000
C	-5.869081000	2.229360000	0.320363000
C	-6.864726000	1.776959000	-0.661983000
C	-8.230198000	2.170997000	-0.589405000
H	-8.616407000	2.809461000	0.195143000
C	-9.017635000	1.645227000	-1.595007000
C	-6.616417000	0.966185000	-1.766784000
C	-5.318742000	0.373424000	-2.215111000
H	-4.484754000	0.945058000	-1.801891000
H	-5.234566000	0.363080000	-3.306063000
H	-5.221653000	-0.658617000	-1.860165000
C	-10.437234000	1.797696000	-1.829794000
C	-11.197685000	2.711099000	-0.828517000
Cl	0.520699000	-1.221932000	-1.626024000
S	3.596732000	2.172843000	-0.612047000
S	8.973797000	1.651865000	1.161031000
P	1.284970000	0.743014000	0.967715000
F	12.934895000	2.252319000	-2.081269000
F	12.133012000	0.237950000	-1.873660000
F	11.019601000	1.745090000	-2.985115000
O	11.587459000	2.842639000	0.097298000
C	0.355235000	2.224499000	0.402524000
C	-0.720126000	2.071553000	-0.487528000
H	-0.991424000	1.083905000	-0.842950000
C	-1.433561000	3.187681000	-0.927763000
H	-2.268880000	3.056756000	-1.609318000
C	-1.076049000	4.465319000	-0.490674000
H	-1.632797000	5.333038000	-0.832406000
C	-0.001485000	4.625660000	0.388051000
H	0.281562000	5.617042000	0.729420000
C	0.710761000	3.512969000	0.837113000
H	1.532118000	3.649888000	1.533106000
C	1.801071000	1.142470000	2.682684000
C	0.896560000	1.748477000	3.569788000
H	-0.098795000	2.019735000	3.234759000
C	1.276935000	2.011863000	4.885182000
H	0.572826000	2.487633000	5.561678000
C	2.553155000	1.659554000	5.333280000
H	2.845546000	1.865115000	6.359063000

C	3.449518000	1.040599000	4.459710000
H	4.443088000	0.763123000	4.800244000
C	3.076504000	0.781739000	3.139519000
H	3.780423000	0.305151000	2.466486000
C	2.823410000	0.736821000	0.023808000
C	3.589267000	-0.370882000	-0.227975000
H	3.286198000	-1.369199000	0.065423000
C	4.821093000	-0.089825000	-0.907078000
C	4.957778000	1.255020000	-1.205090000
C	6.053615000	1.950105000	-1.953315000
H	6.593253000	1.230904000	-2.573794000
H	5.659613000	2.739236000	-2.601440000
H	6.778456000	2.408588000	-1.270663000
C	5.782026000	-1.150153000	-1.244609000
C	5.319455000	-2.417227000	-1.944642000
H	5.050913000	-2.177902000	-2.983963000
H	4.421594000	-2.848423000	-1.487647000
C	6.548158000	-3.347398000	-1.863022000
H	6.641211000	-4.010111000	-2.727767000
H	6.467143000	-3.976365000	-0.969453000
C	7.749871000	-2.391467000	-1.700782000
H	8.197387000	-2.135813000	-2.672835000
H	8.553735000	-2.810504000	-1.085681000
C	7.125395000	-1.152218000	-1.077366000
C	7.971802000	-0.129006000	-0.446510000
C	9.189205000	0.310136000	-1.038848000
H	9.551961000	-0.069528000	-1.985732000
C	9.848706000	1.279581000	-0.308684000
C	7.731650000	0.501051000	0.772118000
C	6.591779000	0.297586000	1.718284000
H	6.144414000	-0.685434000	1.555439000
H	6.915426000	0.371082000	2.761016000
H	5.811862000	1.049431000	1.554595000
C	11.084971000	1.973039000	-0.598983000
C	11.811323000	1.550403000	-1.906151000
C	-4.081960000	2.371221000	1.871986000
H	-3.009711000	2.166028000	1.772539000
H	-4.450198000	4.512520000	2.288484000
H	-6.656078000	3.864228000	1.489535000
H	-5.979939000	4.382031000	-0.051070000

[PdCl$_2$(**DTE^o-COCF$_3$**)(**DTE^c-COCF$_3$**)]

C	7.496451000	-1.255225000	0.414066000
C	7.059525000	0.218059000	0.273332000
S	8.523588000	1.060693000	-0.550586000
C	9.576201000	-0.315011000	-0.124908000
C	8.903949000	-1.419369000	0.334506000
H	9.392217000	-2.355370000	0.578198000
C	5.377302000	-3.965380000	1.765995000
C	4.323032000	-3.055095000	1.089408000
C	5.138810000	-1.881174000	0.595335000
C	6.546567000	-2.210393000	0.643884000
C	6.700915000	-3.662854000	1.019390000
H	5.107487000	-5.023835000	1.732034000
H	3.511112000	-2.762510000	1.761911000
H	3.858138000	-3.564308000	0.234187000
H	7.596117000	-3.865391000	1.613658000
H	6.769208000	-4.269097000	0.105104000
H	5.484383000	-3.679654000	2.818398000
C	3.404076000	-0.132480000	0.105094000
C	3.332012000	1.188915000	-0.219768000
S	4.898097000	1.943686000	-0.565668000
C	5.750389000	0.263023000	-0.544703000
C	4.727198000	-0.673070000	0.120012000
H	2.530458000	-0.718258000	0.366855000
P	1.761066000	2.116120000	-0.140129000
C	1.941968000	3.094963000	1.400656000
C	1.200551000	4.276160000	1.558497000
C	2.757675000	2.645092000	2.448434000
C	1.287231000	5.002873000	2.745519000
C	2.841575000	3.377463000	3.633519000
C	2.108580000	4.556488000	3.784368000
H	0.562374000	4.633124000	0.755847000
H	3.331138000	1.729914000	2.342032000
H	0.713011000	5.917973000	2.857744000
H	3.483196000	3.026246000	4.436544000
H	2.176252000	5.125478000	4.707135000
C	1.781482000	3.375610000	-1.469809000
C	2.706979000	4.433626000	-1.438112000
C	0.846580000	3.305932000	-2.512570000
C	2.711383000	5.389548000	-2.452604000
C	0.850429000	4.271967000	-3.521050000
C	1.784296000	5.309044000	-3.495986000
H	3.411134000	4.525862000	-0.617041000
H	0.126727000	2.496163000	-2.541963000
H	3.433608000	6.200111000	-2.423716000
H	0.122825000	4.210383000	-4.325082000
H	1.787387000	6.057587000	-4.283226000
C	5.908109000	-0.179528000	-2.017885000
H	6.358996000	-1.175148000	-2.063041000
H	4.923485000	-0.222435000	-2.490431000
H	6.536826000	0.513230000	-2.579467000
C	6.919271000	0.808750000	1.696071000
H	6.115567000	0.303644000	2.240008000
H	7.852525000	0.657643000	2.243924000
H	6.702425000	1.878112000	1.667241000
Pd	0.024485000	0.503901000	-0.224333000
Cl	-0.780864000	1.194479000	1.880745000
Cl	0.870669000	-0.178957000	-2.326479000
C	11.001710000	-0.175701000	-0.337310000
O	11.542501000	0.828915000	-0.782003000
C	11.886583000	-1.398760000	0.021689000
F	13.178812000	-1.139350000	-0.201537000
F	11.745135000	-1.738293000	1.323828000
F	11.542311000	-2.481417000	-0.713658000
S	-4.180812000	-2.131408000	1.175161000
S	-9.076214000	-1.269271000	-1.683015000
P	-1.459940000	-1.342682000	-0.192112000
F	-13.542946000	-0.759027000	0.836014000
F	-12.377793000	1.058391000	0.548567000
F	-11.728030000	-0.434965000	1.995978000
O	-11.993223000	-1.871619000	-0.971863000
C	-0.783784000	-2.668548000	0.882767000
C	0.219229000	-2.360461000	1.815602000
H	0.594033000	-1.345635000	1.893349000
C	0.727004000	-3.354036000	2.654867000
H	1.501843000	-3.104523000	3.373788000
C	0.240481000	-4.660257000	2.570423000
H	0.638407000	-5.432497000	3.222454000
C	-0.758767000	-4.973197000	1.645101000
H	-1.140314000	-5.987579000	1.574876000

C	-1.268512000	-3.985439000	0.801867000
H	-2.031465000	-4.245955000	0.075265000
C	-1.796481000	-2.157237000	-1.801371000
C	-0.905163000	-3.107874000	-2.322913000
H	-0.028736000	-3.404674000	-1.756734000
C	-1.146998000	-3.682510000	-3.570635000
H	-0.455829000	-4.423067000	-3.962645000
C	-2.267513000	-3.304210000	-4.315101000
H	-2.452298000	-3.753806000	-5.286533000
C	-3.147043000	-2.345160000	-3.807939000
H	-4.019731000	-2.045021000	-4.380989000
C	-2.913028000	-1.772035000	-2.557138000
H	-3.601845000	-1.029767000	-2.168864000
C	-3.102010000	-0.955249000	0.453138000
C	-3.721854000	0.262671000	0.353344000
H	-3.226847000	1.137264000	-0.051373000
C	-5.075214000	0.273597000	0.828339000
C	-5.464184000	-0.957743000	1.325775000
C	-6.762249000	-1.351774000	1.960532000
H	-7.276445000	-0.463935000	2.335946000
H	-6.608403000	-2.042463000	2.795417000
H	-7.428402000	-1.840764000	1.240315000
C	-5.899564000	1.490354000	0.799237000
H	-5.300168000	2.727292000	2.450503000
C	-6.399639000	3.843389000	0.897962000
H	-6.081650000	4.276663000	-0.056934000
C	-7.695512000	3.032966000	0.678708000
C	-7.182810000	1.629621000	0.391552000
C	-8.069037000	0.623207000	-0.211268000
C	-9.420871000	0.463759000	0.205081000
H	-9.865766000	1.055214000	0.995482000
C	-10.103223000	-0.524788000	-0.476858000
C	-7.741224000	-0.245201000	-1.249661000
C	-6.445127000	-0.374437000	-1.983741000
H	-5.874301000	0.552865000	-1.898532000
H	-6.602285000	-0.597010000	-3.043511000
H	-5.837517000	-1.180166000	-1.557235000
C	-11.466690000	-0.982480000	-0.319456000
C	-12.300089000	-0.270998000	0.781918000
C	-5.360210000	2.798206000	1.354594000
H	-4.344875000	3.018479000	1.005944000
H	-6.522529000	4.663982000	1.610123000
H	-8.324472000	3.023233000	1.581410000
H	-8.317500000	3.426280000	-0.132806000

[PdCl$_2$(**DTE^c-COCF$_3$**)$_2$]

C	6.854347000	-2.166313000	0.508531000
C	6.709744000	-0.671777000	0.864529000
S	8.429968000	0.038160000	0.599200000
C	9.128936000	-1.602950000	0.609388000
C	8.201414000	-2.611861000	0.545441000
H	8.465102000	-3.660955000	0.485659000
C	4.077866000	-4.611946000	0.473209000
C	3.351611000	-3.306755000	0.064828000
C	4.436756000	-2.258543000	0.148683000
C	5.729681000	-2.898707000	0.250345000
C	5.557920000	-4.386801000	0.073037000
H	3.646207000	-5.500242000	0.004881000
H	2.486145000	-3.079393000	0.694317000
H	2.979809000	-3.369089000	-0.966130000
H	6.265912000	-4.978376000	0.659962000
H	5.710795000	-4.645552000	-0.984226000
H	4.014458000	-4.741417000	1.559324000
C	3.155616000	-0.098809000	0.091755000
C	3.375502000	1.235721000	0.254328000
S	5.082554000	1.680382000	0.442324000
C	5.618408000	-0.059774000	-0.040724000
C	4.338822000	-0.901331000	0.097184000
H	2.162824000	-0.520955000	-0.012873000
P	2.011559000	2.439173000	0.384310000
C	1.979122000	2.847197000	2.170736000
C	1.422710000	4.065758000	2.590188000
C	2.445470000	1.933230000	3.125908000
C	1.349056000	4.368861000	3.949089000
C	2.368683000	2.242401000	4.484935000
C	1.822423000	3.458996000	4.898727000
H	1.053749000	4.779268000	1.859641000
H	2.873199000	0.985764000	2.814151000
H	0.921143000	5.315660000	4.265604000
H	2.740691000	1.532439000	5.217984000
H	1.765703000	3.698302000	5.956753000
C	2.567302000	3.989029000	-0.420322000
C	3.596932000	4.760732000	0.146633000
C	1.951107000	4.421743000	-1.603392000
C	4.015536000	5.934846000	-0.477464000
C	2.369777000	5.603034000	-2.219268000
C	3.403580000	6.356913000	-1.661413000
H	4.062314000	4.459434000	1.079873000
H	1.155851000	3.833774000	-2.046609000
H	4.815164000	6.521822000	-0.035415000
H	1.886280000	5.930832000	-3.134918000
H	3.729497000	7.274174000	-2.143454000
C	5.992312000	-0.014096000	-1.540937000
H	6.265614000	-1.014425000	-1.889175000
H	5.130660000	0.330474000	-2.118280000
H	6.830586000	0.659762000	-1.724658000
C	6.388520000	-0.570493000	2.374063000
H	5.417029000	-1.025973000	2.587562000
H	7.151849000	-1.106690000	2.943114000
H	6.368645000	0.467385000	2.711697000
Pd	0.082184000	1.443394000	-0.565096000
Cl	-1.106472000	1.749215000	1.440343000
Cl	1.306136000	1.098080000	-2.575325000
C	-7.759714000	-0.148636000	1.072573000
C	-6.680846000	-1.162533000	0.643836000
S	-7.656738000	-2.642880000	0.017535000
C	-9.126813000	-2.038533000	0.826700000
C	-9.024298000	-0.752840000	1.295040000
H	-9.846212000	-0.221247000	1.759133000
C	-7.068931000	3.253015000	2.344480000
C	-5.904205000	3.053737000	1.343374000
C	-6.094985000	1.632516000	0.862656000
C	-7.407532000	1.161069000	1.243568000
C	-8.186677000	2.302863000	1.847121000
H	-7.398813000	4.292851000	2.409885000
H	-4.919250000	3.222611000	1.789648000
H	-5.996630000	3.745480000	0.494915000
H	-8.876981000	1.987590000	2.634417000
H	-8.782741000	2.791702000	1.063549000
H	-6.747997000	2.938775000	3.343761000
C	-3.907033000	1.101809000	-0.257683000

C	-3.254001000	0.026842000	-0.778795000
S	-4.219065000	-1.465324000	-0.829287000
C	-5.777891000	-0.496553000	-0.418013000
C	-5.255588000	0.838790000	0.140282000
H	-3.429264000	2.063153000	-0.116996000
P	-1.612120000	0.135671000	-1.562752000
C	-0.973188000	-1.576812000	-1.547927000
C	-0.374207000	-2.155377000	-2.676286000
C	-0.979152000	-2.276142000	-0.327564000
C	0.184452000	-3.431910000	-2.590265000
C	-0.422184000	-3.552536000	-0.250932000
C	0.157516000	-4.133393000	-1.382499000
H	-0.343283000	-1.616251000	-3.616332000
H	-1.419265000	-1.827849000	0.558558000
H	0.640435000	-3.876883000	-3.469788000
H	-0.440093000	-4.090888000	0.691996000
H	0.591875000	-5.126944000	-1.320544000
C	-2.089633000	0.567617000	-3.275595000
C	-2.787634000	-0.342233000	-4.086611000
C	-1.860698000	1.873735000	-3.731615000
C	-3.238788000	0.053560000	-5.346201000
C	-2.320043000	2.264499000	-4.989788000
C	-3.006560000	1.355399000	-5.798477000
H	-2.979602000	-1.352092000	-3.739662000
H	-1.318767000	2.576372000	-3.107343000
H	-3.773488000	-0.655294000	-5.971666000
H	-2.136488000	3.276497000	-5.338867000
H	-3.360322000	1.660200000	-6.779240000
C	-6.490918000	-0.196709000	-1.757875000
H	-7.378889000	0.416904000	-1.579828000
H	-5.813470000	0.355823000	-2.414132000
H	-6.797019000	-1.113887000	-2.263276000
C	-5.917163000	-1.607813000	1.912827000
H	-5.369038000	-0.764407000	2.342835000
H	-6.632121000	-1.969809000	2.655712000
H	-5.208252000	-2.408836000	1.695391000
C	-10.285024000	-2.905259000	0.877861000
O	-10.329277000	-4.034476000	0.405723000
C	10.570949000	-1.731401000	0.633043000
O	11.351506000	-0.788249000	0.661372000
C	11.147664000	-3.171989000	0.606377000
F	12.483549000	-3.155600000	0.656121000
F	10.697299000	-3.898893000	1.655058000
F	10.777505000	-3.823621000	-0.520540000
C	-11.551359000	-2.342672000	1.577127000
F	-12.539276000	-3.243461000	1.578330000
F	-11.291178000	-2.002693000	2.860561000
F	-11.999375000	-1.228898000	0.951937000

[PdCl$_2$(**DTE^{o}-C$_6$F$_5$**)$_2$]

C	5.205534000	-2.190946000	-1.849110000
C	4.043159000	-2.206101000	-1.097864000
S	4.116420000	-3.428369000	0.141493000
C	5.729884000	-3.935056000	-0.344291000
C	6.144963000	-3.182242000	-1.418326000
H	7.104732000	-3.319320000	-1.896998000
C	2.808994000	-1.372690000	-1.244260000
H	2.828424000	-0.493847000	-0.593101000
H	2.725133000	-1.010615000	-2.271624000
H	1.905098000	-1.937189000	-0.998317000
C	5.878928000	-0.791226000	-5.308541000
C	5.708511000	0.508748000	-4.495424000
C	5.283017000	0.018364000	-3.120252000
C	5.490146000	-1.314255000	-2.994047000
C	6.144442000	-1.879324000	-4.246711000
H	6.668148000	-0.724711000	-6.062834000
H	4.983647000	1.200183000	-4.940357000
H	6.653356000	1.065778000	-4.407706000
H	5.740259000	-2.858972000	-4.524229000
H	7.221890000	-2.020990000	-4.073911000
H	4.941086000	-1.018088000	-5.827731000
C	3.796377000	1.954171000	-2.421841000
C	3.456859000	2.761234000	-1.368679000
S	4.377934000	2.320435000	0.056259000
C	5.231036000	1.061436000	-0.801648000
C	4.798461000	0.972798000	-2.112300000
H	3.321005000	2.021734000	-3.392595000
C	6.278694000	0.260295000	-0.093268000
H	5.832407000	-0.565697000	0.472770000
H	6.968214000	-0.177002000	-0.819184000
H	6.853344000	0.876282000	0.605662000
P	2.093542000	3.943947000	-1.239744000
C	2.868155000	5.586739000	-0.998130000
C	2.026184000	6.678093000	-0.730993000
C	4.246397000	5.793933000	-1.143905000
C	2.559150000	7.960357000	-0.614526000
C	4.776339000	7.080757000	-1.018094000
C	3.935804000	8.163343000	-0.753909000
H	0.959263000	6.521739000	-0.607615000
H	4.906054000	4.961321000	-1.364701000
H	1.901784000	8.799580000	-0.406292000
H	5.845776000	7.233965000	-1.131592000
H	4.350232000	9.162767000	-0.656252000
C	1.389618000	4.081663000	-2.925012000
C	2.209591000	4.469833000	-3.998089000
C	0.020804000	3.871160000	-3.136921000
C	1.666450000	4.616958000	-5.273614000
C	-0.519804000	4.032233000	-4.414661000
C	0.301168000	4.397790000	-5.483017000
H	3.266776000	4.658163000	-3.835158000
H	-0.614799000	3.574016000	-2.310652000
H	2.306262000	4.909213000	-6.101273000
H	-1.581807000	3.867940000	-4.572654000
H	-0.120959000	4.517781000	-6.476764000
Pd	0.702581000	3.030168000	0.439570000
Cl	0.336920000	1.185208000	-1.012933000
Cl	1.121163000	4.813209000	1.912836000
C	-6.608150000	-1.155274000	1.355790000
C	-5.529675000	-1.897637000	0.909300000
S	-5.955138000	-2.844962000	-0.488377000
C	-7.619177000	-2.273144000	-0.462485000
C	-7.789991000	-1.396737000	0.583809000
H	-8.731327000	-0.907444000	0.793531000
C	-4.134938000	-1.967035000	1.450850000
H	-3.458148000	-1.304559000	0.899278000
H	-4.129830000	-1.650037000	2.496261000
H	-3.728549000	-2.981944000	1.392837000
C	-7.168915000	0.559205000	4.696831000
C	-6.071118000	1.453403000	4.081141000
C	-5.697461000	0.727108000	2.800108000
C	-6.607127000	-0.228076000	2.496669000

C	-7.750302000	-0.225349000	3.500052000
H	-7.925311000	1.126965000	5.245966000
H	-5.213747000	1.602523000	4.747919000
H	-6.447698000	2.457256000	3.834556000
H	-8.076828000	-1.236564000	3.766508000
H	-8.625054000	0.283577000	3.068335000
H	-6.709503000	-0.146047000	5.398399000
C	-3.220868000	1.261614000	2.603599000
C	-2.229750000	1.593031000	1.709697000
S	-2.902911000	1.799586000	0.109123000
C	-4.509051000	1.423332000	0.663093000
C	-4.520088000	1.133075000	2.017595000
H	-3.029625000	1.094334000	3.657348000
C	-5.652229000	1.427525000	-0.304675000
H	-5.707195000	0.483747000	-0.859219000
H	-6.594996000	1.547045000	0.234415000
H	-5.561672000	2.240275000	-1.032379000
P	-0.474559000	1.816784000	2.085293000
C	-0.525521000	2.484660000	3.792583000
C	0.234420000	1.925465000	4.828428000
C	-1.324993000	3.610799000	4.051761000
C	0.186065000	2.480032000	6.110305000
C	-1.373691000	4.155733000	5.332852000
C	-0.617445000	3.591829000	6.365516000
H	0.857235000	1.056275000	4.646380000
H	-1.913585000	4.054236000	3.254949000
H	0.773964000	2.035356000	6.908164000
H	-1.998321000	5.023467000	5.524407000
H	-0.655659000	4.018855000	7.363624000
C	0.276049000	0.151311000	2.242959000
C	1.676751000	0.055825000	2.212164000
C	-0.495266000	-1.006206000	2.403187000
C	2.296230000	-1.184878000	2.355293000
C	0.130220000	-2.248715000	2.530538000
C	1.523633000	-2.340106000	2.510252000
H	2.279407000	0.947177000	2.063235000
H	-1.577763000	-0.942457000	2.420350000
H	3.379385000	-1.252594000	2.328768000
H	-0.473958000	-3.144251000	2.644715000
H	2.007461000	-3.307303000	2.608240000
C	6.480399000	-4.976130000	0.361263000
C	7.887000000	-5.028161000	0.327126000
C	5.863742000	-5.989666000	1.115557000
C	8.618618000	-6.016983000	0.974025000
C	6.577040000	-6.981414000	1.778162000
C	7.965026000	-7.003526000	1.704682000
C	-8.630768000	-2.737875000	-1.414333000
C	-8.314842000	-3.216271000	-2.697586000
C	-10.002052000	-2.733912000	-1.096549000
C	-9.276995000	-3.656172000	-3.598923000
C	-10.980431000	-3.160698000	-1.986604000
C	-10.621587000	-3.626112000	-3.247164000
F	8.592200000	-4.096194000	-0.338514000
F	9.957100000	-6.014817000	0.905159000
F	8.662538000	-7.956918000	2.331892000
F	5.928573000	-7.921938000	2.478455000
F	4.522463000	-6.030198000	1.225924000
F	-10.424273000	-2.318132000	0.110575000
F	-12.271388000	-3.135447000	-1.627615000
F	-11.557275000	-4.041460000	-4.107666000
F	-8.911336000	-4.099445000	-4.809174000
F	-7.032544000	-3.269045000	-3.103239000

[$PdCl_2$(**DTE^o^-C_6F_5**)(**DTE^c^-C_6F_5**)]

C	-7.241439000	0.599706000	-0.373321000
C	-6.297781000	-0.333423000	0.408945000
S	-6.979302000	-2.050359000	0.089624000
C	-8.537170000	-1.367610000	-0.467371000
C	-8.498435000	-0.018590000	-0.663106000
H	-9.343699000	0.540391000	-1.044477000
C	-6.831455000	4.257746000	-0.614135000
C	-5.358063000	3.786992000	-0.539218000
C	-5.473650000	2.291150000	-0.345665000
C	-6.832480000	1.869059000	-0.640108000
C	-7.605506000	3.045338000	-1.188551000
H	-6.956822000	5.163245000	-1.213731000
H	-4.788486000	4.279589000	0.255336000
H	-4.835914000	3.993801000	-1.483543000
H	-8.663709000	3.035751000	-0.911526000
H	-7.554163000	3.047177000	-2.286604000
H	-7.196862000	4.471902000	0.396702000
C	-3.165790000	1.614062000	0.403961000
C	-2.530329000	0.522028000	0.907023000
S	-3.531377000	-0.950628000	0.954673000
C	-4.854463000	-0.116015000	-0.094812000
C	-4.521905000	1.384930000	-0.000142000
H	-2.687142000	2.584197000	0.332085000
P	-0.852846000	0.590393000	1.595952000
C	-1.136228000	0.618123000	3.408793000
C	-0.102203000	0.225024000	4.273992000
C	-2.347619000	1.076750000	3.944500000
C	-0.288218000	0.273333000	5.654452000
C	-2.526553000	1.127683000	5.328656000
C	-1.500502000	0.724428000	6.185005000
H	0.843186000	-0.123698000	3.870861000
H	-3.154296000	1.385184000	3.287436000
H	0.515692000	-0.037396000	6.315518000
H	-3.471948000	1.476674000	5.734120000
H	-1.643342000	0.761201000	7.261243000
C	-0.089757000	-1.053938000	1.290543000
C	-0.492999000	-2.178707000	2.031243000
C	0.899478000	-1.198156000	0.305864000
C	0.078446000	-3.426094000	1.780416000
C	1.468507000	-2.448959000	0.061231000
C	1.060717000	-3.563565000	0.795665000
H	-1.242382000	-2.082534000	2.810596000
H	1.217877000	-0.340828000	-0.275359000
H	-0.241958000	-4.288759000	2.357293000
H	2.231158000	-2.548922000	-0.704582000
H	1.508322000	-4.534786000	0.604565000
C	-4.624238000	-0.561332000	-1.557062000
H	-5.339803000	-0.063802000	-2.218303000
H	-3.613917000	-0.279166000	-1.863957000
H	-4.735758000	-1.640898000	-1.672752000
C	-6.481556000	-0.035471000	1.915915000
H	-6.146139000	0.981054000	2.141118000
H	-7.540903000	-0.114641000	2.172728000
H	-5.918442000	-0.734138000	2.537186000
Pd	0.478035000	2.316248000	0.656137000
Cl	1.107015000	3.087131000	2.794515000
Cl	-0.261012000	1.650879000	-1.490451000

C	-9.683307000	-2.237841000	-0.753492000
C	-9.991376000	-3.379831000	0.005881000
C	-10.541788000	-1.976838000	-1.838276000
C	-11.072480000	-4.203947000	-0.286896000
C	-11.629507000	-2.785807000	-2.142262000
C	-11.899063000	-3.907974000	-1.364982000
F	-9.245385000	-3.712501000	1.071393000
F	-11.327270000	-5.275432000	0.475616000
F	-12.944305000	-4.690387000	-1.650051000
F	-12.413689000	-2.495705000	-3.189154000
F	-10.326505000	-0.922321000	-2.641896000
C	6.791732000	-0.952200000	-1.611158000
C	5.453680000	-1.278697000	-1.742104000
S	5.174073000	-2.947995000	-1.333036000
C	6.874306000	-3.226002000	-0.975814000
C	7.584561000	-2.067506000	-1.189749000
H	8.653100000	-1.995954000	-1.040561000
C	4.304689000	-0.426703000	-2.189895000
H	3.755243000	-0.013742000	-1.336009000
H	4.675764000	0.414574000	-2.779831000
H	3.596070000	-0.993443000	-2.802403000
C	8.726646000	2.000546000	-3.018276000
C	7.863380000	2.689413000	-1.938511000
C	6.959224000	1.574954000	-1.443753000
C	7.392238000	0.365730000	-1.868939000
C	8.688352000	0.499555000	-2.652967000
H	9.742233000	2.402260000	-3.074672000
H	7.300442000	3.548850000	-2.321760000
H	8.471337000	3.066212000	-1.102688000
H	8.717854000	-0.156545000	-3.529819000
H	9.540798000	0.214891000	-2.018318000
H	8.259066000	2.142352000	-3.999001000
C	4.778766000	2.774356000	-0.936756000
C	3.719783000	2.814611000	-0.061475000
S	4.007045000	1.726021000	1.282841000
C	5.537395000	1.215705000	0.629477000
C	5.806739000	1.847619000	-0.571718000
H	4.809379000	3.349865000	-1.853860000
C	6.357532000	0.197379000	1.360069000
H	5.988037000	-0.817581000	1.173441000
H	7.394262000	0.237803000	1.018164000
H	6.340497000	0.367639000	2.441348000
P	2.157608000	3.712723000	-0.255177000
C	2.355403000	5.340373000	0.553972000
C	1.204920000	6.040480000	0.946643000
C	3.619674000	5.915425000	0.736452000
C	1.320535000	7.313271000	1.505179000
C	3.730288000	7.184967000	1.306928000
C	2.583107000	7.884869000	1.688557000
H	0.226006000	5.586704000	0.821827000
H	4.512178000	5.373441000	0.438335000
H	0.427557000	7.852578000	1.807605000
H	4.712377000	7.625695000	1.452536000
H	2.672359000	8.872320000	2.132465000
C	2.114395000	4.089318000	-2.050688000
C	1.856202000	5.387930000	-2.512959000
C	2.307364000	3.052654000	-2.981169000
C	1.802752000	5.646910000	-3.884918000
C	2.264452000	3.319827000	-4.347952000
C	2.009869000	4.617002000	-4.803535000
H	1.705526000	6.201481000	-1.811498000
H	2.497054000	2.042560000	-2.637137000
H	1.606995000	6.657731000	-4.231018000
H	2.423012000	2.512415000	-5.057015000
H	1.973426000	4.822097000	-5.869689000
C	7.380455000	-4.507958000	-0.480020000
C	8.557974000	-4.592060000	0.286789000
C	6.736329000	-5.733198000	-0.723577000
C	9.062061000	-5.798145000	0.758712000
C	7.218436000	-6.948664000	-0.253015000
C	8.392198000	-6.987155000	0.490910000
F	9.248932000	-3.483717000	0.607020000
F	10.189089000	-5.816580000	1.483461000
F	8.870161000	-8.150738000	0.944445000
F	6.558551000	-8.083057000	-0.522510000
F	5.595796000	-5.767218000	-1.437831000

[PdCl$_2$(**DTE^c-C$_6$F$_5$**)$_2$]

C	-6.781029000	-2.169103000	-0.437751000
C	-6.684611000	-0.661313000	-0.743637000
S	-8.421511000	-0.020869000	-0.449801000
C	-9.085706000	-1.680916000	-0.530729000
C	-8.126635000	-2.649983000	-0.503151000
H	-8.363547000	-3.706574000	-0.496927000
C	-3.925038000	-4.506255000	-0.535357000
C	-3.240046000	-3.196761000	-0.073925000
C	-4.359232000	-2.181696000	-0.103100000
C	-5.634934000	-2.866829000	-0.214504000
C	-5.404654000	-4.355789000	-0.100115000
H	-3.450647000	-5.400224000	-0.121384000
H	-2.384807000	-2.913368000	-0.694851000
H	-2.861190000	-3.294246000	0.951910000
H	-6.096579000	-4.949483000	-0.704640000
H	-5.527672000	-4.673055000	0.945124000
H	-3.876632000	-4.577445000	-1.627919000
C	-3.145152000	0.015984000	0.016837000
C	-3.406231000	1.345816000	-0.106554000
S	-5.137884000	1.734804000	-0.274848000
C	-5.609209000	-0.035458000	0.171330000
C	-4.301503000	-0.827959000	-0.003521000
H	-2.137705000	-0.376224000	0.100818000
P	-2.086747000	2.588212000	-0.225651000
C	-2.108752000	3.081247000	-1.992335000
C	-1.549606000	4.312585000	-2.369017000
C	-2.624606000	2.226093000	-2.975435000
C	-1.520938000	4.686261000	-3.711767000
C	-2.594167000	2.605902000	-4.318698000
C	-2.044711000	3.834794000	-4.689108000
H	-1.142962000	4.981548000	-1.616254000
H	-3.053354000	1.268923000	-2.696834000
H	-1.089393000	5.642157000	-3.994504000
H	-3.004019000	1.940510000	-5.073178000
H	-2.023636000	4.128941000	-5.734576000
C	-2.660918000	4.099071000	0.642807000
C	-3.730961000	4.850930000	0.126053000
C	-2.021327000	4.526318000	1.815007000
C	-4.166333000	5.997298000	0.789120000
C	-2.456496000	5.680463000	2.470242000
C	-3.530610000	6.413127000	1.962761000
H	-4.214539000	4.556182000	-0.800138000
H	-1.194668000	3.954673000	2.219910000

H	-4.997313000	6.568197000	0.385272000
H	-1.953824000	6.003610000	3.377275000
H	-3.869112000	7.309111000	2.475318000
C	-5.976223000	-0.044966000	1.672462000
H	-6.210849000	-1.063637000	1.995098000
H	-5.123476000	0.315287000	2.253561000
H	-6.836437000	0.593431000	1.881861000
C	-6.383074000	-0.504710000	-2.252989000
H	-5.396957000	-0.918048000	-2.483745000
H	-7.131486000	-1.054339000	-2.829328000
H	-6.403852000	0.541959000	-2.561632000
Pd	-0.101968000	1.601272000	0.621402000
Cl	0.944542000	1.831018000	-1.475004000
Cl	-1.216872000	1.241318000	2.690703000
C	7.838833000	0.674134000	-1.081883000
C	6.867387000	-0.445755000	-0.666254000
S	7.983690000	-1.805672000	-0.017219000
C	9.405044000	-1.066691000	-0.813212000
C	9.169598000	0.190382000	-1.285780000
H	9.939957000	0.792074000	-1.752035000
C	6.793136000	3.953936000	-2.409485000
C	5.642591000	3.639824000	-1.421945000
C	5.977363000	2.252908000	-0.919388000
C	7.348413000	1.928834000	-1.270751000
C	8.003748000	3.148158000	-1.876153000
H	6.999946000	5.024242000	-2.492556000
H	4.650765000	3.699304000	-1.881006000
H	5.650486000	4.346495000	-0.580436000
H	8.737792000	2.905730000	-2.650150000
H	8.526849000	3.718862000	-1.095677000
H	6.526540000	3.585564000	-3.406542000
C	3.838917000	1.497925000	0.174417000
C	3.296790000	0.364424000	0.694080000
S	4.423291000	-1.020705000	0.752718000
C	5.874469000	0.116584000	0.375077000
C	5.215671000	1.382414000	-0.205275000
H	3.263678000	2.404080000	0.028831000
P	1.669694000	0.308258000	1.496925000
C	1.108336000	-1.425286000	1.353153000
C	0.428049000	-2.061607000	2.403125000
C	1.245586000	-2.080346000	0.117070000
C	-0.077384000	-3.350360000	2.226184000
C	0.737479000	-3.368855000	-0.051814000
C	0.080037000	-4.007424000	1.002972000
H	0.293649000	-1.555359000	3.352526000
H	1.747787000	-1.587512000	-0.709879000
H	-0.595225000	-3.839712000	3.045912000
H	0.856303000	-3.871521000	-1.007123000
H	-0.315308000	-5.010267000	0.869761000
C	2.142645000	0.614209000	3.239411000
C	2.779337000	-0.368834000	4.012822000
C	1.984812000	1.909540000	3.754186000
C	3.241311000	-0.056548000	5.292641000
C	2.454392000	2.216945000	5.030895000
C	3.080842000	1.234179000	5.802299000
H	2.915583000	-1.371910000	3.623149000
H	1.489015000	2.669804000	3.159067000
H	3.729163000	-0.822726000	5.888251000
H	2.325293000	3.220988000	5.424668000
H	3.442476000	1.473624000	6.798258000
C	6.523932000	0.496149000	1.725409000
H	7.338677000	1.208220000	1.563908000
H	5.774006000	0.966707000	2.366665000
H	6.923111000	-0.378738000	2.241567000
C	6.186677000	-0.979870000	-1.948549000
H	5.556391000	-0.203326000	-2.391268000
H	6.954956000	-1.259194000	-2.673912000
H	5.567595000	-1.854615000	-1.741942000
C	10.696844000	-1.761539000	-0.850383000
C	11.904805000	-1.059326000	-0.681311000
C	10.818588000	-3.148282000	-1.042863000
C	13.143907000	-1.687027000	-0.709801000
C	12.049762000	-3.794179000	-1.069187000
C	13.220691000	-3.062804000	-0.903618000
C	-10.534082000	-1.915652000	-0.537524000
C	-11.445389000	-1.084332000	-1.211369000
C	-11.098536000	-3.001408000	0.158379000
C	-12.817260000	-1.311053000	-1.197212000
C	-12.465800000	-3.246971000	0.178471000
C	-13.333840000	-2.398380000	-0.501633000
F	9.728395000	-3.909292000	-1.228495000
F	12.111428000	-5.117555000	-1.267097000
F	14.408697000	-3.674051000	-0.933627000
F	14.265412000	-0.974069000	-0.539593000
F	11.899211000	0.267007000	-0.469396000
F	-11.012099000	-0.028583000	-1.918501000
F	-13.641195000	-0.492247000	-1.864176000
F	-14.650202000	-2.628446000	-0.488779000
F	-12.952990000	-4.292643000	0.859823000
F	-10.320021000	-3.846555000	0.853718000

[PdCl$_2$(**DTE^o-Ph**)$_2$]

C	5.914833000	-1.584349000	-1.505057000
C	5.223992000	-1.812995000	-0.329509000
S	6.174829000	-2.784059000	0.768493000
C	7.514501000	-2.898015000	-0.364009000
C	7.202497000	-2.220789000	-1.515852000
H	7.891124000	-2.134661000	-2.349192000
C	3.847299000	-1.379518000	0.064438000
H	3.853830000	-0.453265000	0.649007000
H	3.250316000	-1.196503000	-0.831689000
H	3.339081000	-2.139062000	0.665234000
C	4.619160000	-0.452781000	-4.891249000
C	4.442268000	0.835288000	-4.057986000
C	4.821423000	0.407732000	-2.649679000
C	5.423446000	-0.805271000	-2.650558000
C	5.598072000	-1.321269000	-4.070790000
H	4.968182000	-0.257609000	-5.909189000
H	3.426196000	1.243777000	-4.113720000
H	5.115851000	1.638167000	-4.392381000
H	5.392945000	-2.394309000	-4.153936000
H	6.638995000	-1.175156000	-4.395788000
H	3.657020000	-0.971613000	-4.967804000
C	3.308552000	1.908944000	-1.271096000
C	3.252766000	2.685180000	-0.138203000
S	4.803626000	2.699273000	0.663257000
C	5.512148000	1.653797000	-0.540489000
C	4.584763000	1.303127000	-1.507002000
H	2.447824000	1.738936000	-1.907962000
C	6.950521000	1.253931000	-0.425483000
H	7.072842000	0.403069000	0.254555000

H	7.330178000	0.948405000	-1.403331000
H	7.570238000	2.075589000	-0.052454000
P	1.714987000	3.365967000	0.533424000
C	2.140368000	4.257226000	2.074687000
C	1.465788000	3.969725000	3.268566000
C	3.091228000	5.291949000	2.043317000
C	1.754643000	4.697341000	4.425033000
C	3.384127000	6.006299000	3.204323000
C	2.716375000	5.709026000	4.396222000
H	0.730224000	3.174093000	3.295183000
H	3.593133000	5.545820000	1.114059000
H	1.228807000	4.469492000	5.347721000
H	4.125789000	6.799293000	3.175904000
H	2.942318000	6.270060000	5.298636000
C	1.226368000	4.730941000	-0.589150000
C	1.980542000	5.103773000	-1.708033000
C	0.048357000	5.429819000	-0.279437000
C	1.561307000	6.171299000	-2.507063000
C	-0.361989000	6.496064000	-1.076479000
C	0.393459000	6.867967000	-2.193576000
H	2.891539000	4.569291000	-1.955859000
H	-0.546895000	5.139278000	0.581968000
H	2.152249000	6.457092000	-3.372697000
H	-1.274087000	7.032326000	-0.830761000
H	0.070100000	7.696908000	-2.816790000
Pd	0.341764000	1.444771000	0.632541000
Cl	-0.988796000	2.216583000	-1.164186000
Cl	1.694075000	0.671736000	2.412786000
C	-7.436424000	-2.062665000	0.209549000
C	-6.839482000	-1.786238000	-1.006319000
S	-7.957437000	-0.974451000	-2.076026000
C	-9.250162000	-1.013947000	-0.885705000
C	-8.807169000	-1.635598000	0.254223000
H	-9.428052000	-1.754064000	1.135147000
C	-5.451605000	-2.093840000	-1.478455000
H	-4.785748000	-1.230645000	-1.363732000
H	-5.034039000	-2.913389000	-0.888657000
H	-5.438964000	-2.385134000	-2.533968000
C	-6.393939000	-4.500379000	2.927092000
C	-5.295723000	-3.422981000	3.055341000
C	-5.575077000	-2.489944000	1.888996000
C	-6.795106000	-2.728090000	1.352800000
C	-7.525350000	-3.810800000	2.133413000
H	-6.724136000	-4.892453000	3.893240000
H	-4.281120000	-3.838023000	3.030876000
H	-5.376546000	-2.862971000	3.998914000
H	-8.074741000	-4.499178000	1.481822000
H	-8.267910000	-3.352339000	2.803485000
H	-6.007525000	-5.342461000	2.341976000
C	-3.230979000	-1.736333000	1.267359000
C	-2.486793000	-0.649369000	0.872500000
S	-3.484301000	0.787233000	0.838440000
C	-4.895251000	-0.100691000	1.341467000
C	-4.610598000	-1.445137000	1.512821000
H	-2.813479000	-2.731711000	1.364007000
C	-6.202204000	0.609901000	1.514243000
H	-6.723730000	0.719138000	0.556497000
H	-6.853237000	0.034745000	2.176952000
H	-6.066134000	1.609184000	1.939803000
P	-0.735153000	-0.647149000	0.423802000
C	-0.071158000	-2.041363000	1.414019000
C	0.683077000	-3.065224000	0.825245000
C	-0.287722000	-2.054728000	2.802177000
C	1.209483000	-4.091566000	1.614532000
C	0.232257000	-3.084711000	3.583166000
C	0.984947000	-4.104314000	2.991644000
H	0.863459000	-3.070354000	-0.243950000
H	-0.866306000	-1.264431000	3.269315000
H	1.791436000	-4.880796000	1.147262000
H	0.054814000	-3.088024000	4.654751000
H	1.393555000	-4.903884000	3.602970000
C	-0.615596000	-1.227282000	-1.312495000
C	0.549321000	-0.924790000	-2.034667000
C	-1.617295000	-2.004833000	-1.906801000
C	0.715737000	-1.407398000	-3.332157000
C	-1.453395000	-2.473481000	-3.212319000
C	-0.287925000	-2.179574000	-3.924348000
H	1.320171000	-0.308795000	-1.583011000
H	-2.522501000	-2.241055000	-1.357549000
H	1.624192000	-1.173588000	-3.879774000
H	-2.237880000	-3.068715000	-3.670995000
H	-0.163240000	-2.547868000	-4.938684000
C	8.742418000	-3.638123000	-0.047436000
C	9.513840000	-4.199169000	-1.084390000
C	9.186043000	-3.805444000	1.277978000
C	10.691773000	-4.888721000	-0.803688000
C	10.358777000	-4.506887000	1.555791000
C	11.120315000	-5.048796000	0.517493000
H	9.174640000	-4.108429000	-2.111814000
H	8.620228000	-3.368484000	2.096272000
H	11.270365000	-5.314013000	-1.619187000
H	10.682041000	-4.621621000	2.586702000
H	12.035287000	-5.592324000	0.734878000
C	-10.578927000	-0.451611000	-1.158898000
C	-10.764347000	0.599476000	-2.076666000
C	-11.710089000	-0.959996000	-0.490157000
C	-12.034363000	1.122926000	-2.316596000
C	-12.975843000	-0.426042000	-0.723552000
C	-13.146291000	0.616452000	-1.639395000
H	-9.907838000	1.024081000	-2.593213000
H	-11.595238000	-1.789289000	0.201147000
H	-12.152414000	1.935126000	-3.028496000
H	-13.834298000	-0.834518000	-0.197385000
H	-14.134715000	1.026961000	-1.824577000

[PdCl$_2$(**DTE^o-Ph**)(**DTE^c-Ph**)]

C	7.512552000	0.025685000	-0.202578000
C	6.408326000	-1.039199000	-0.357158000
S	7.107409000	-2.547711000	0.507521000
C	8.763493000	-1.875736000	0.433417000
C	8.789792000	-0.561486000	0.066502000
H	9.710397000	0.007470000	-0.004448000
C	7.231216000	3.497880000	-1.417438000
C	5.765313000	3.188306000	-1.026767000
C	5.808996000	1.735269000	-0.607270000
C	7.190301000	1.331047000	-0.410934000
C	8.083288000	2.542980000	-0.544931000
H	7.493840000	4.549858000	-1.276572000
H	5.053535000	3.380458000	-1.836018000
H	5.452937000	3.804995000	-0.172690000
H	9.059919000	2.315243000	-0.982539000

H	8.263548000	2.990190000	0.443092000
H	7.385299000	3.252898000	-2.474580000
C	3.383156000	1.053010000	-0.607126000
C	2.636419000	-0.077603000	-0.496695000
S	3.577199000	-1.542268000	-0.105024000
C	5.100982000	-0.496676000	0.260276000
C	4.786505000	0.868366000	-0.381136000
H	2.949447000	2.012619000	-0.862080000
P	0.865907000	-0.169930000	-0.866631000
C	0.833428000	-0.920871000	-2.542481000
C	-0.322211000	-1.568754000	-3.010941000
C	1.943944000	-0.800389000	-3.392424000
C	-0.354309000	-2.103815000	-4.298213000
C	1.902416000	-1.329978000	-4.683437000
C	0.756298000	-1.985308000	-5.137802000
H	-1.193439000	-1.654000000	-2.374133000
H	2.843352000	-0.300904000	-3.048384000
H	-1.251362000	-2.608432000	-4.645435000
H	2.770797000	-1.235048000	-5.329048000
H	0.727562000	-2.401158000	-6.140959000
C	0.203067000	-1.444211000	0.285064000
C	0.132447000	-2.806078000	-0.045473000
C	-0.228969000	-1.022488000	1.554111000
C	-0.390590000	-3.723099000	0.869005000
C	-0.738799000	-1.944786000	2.467375000
C	-0.832941000	-3.295404000	2.122513000
H	0.474341000	-3.156530000	-1.013035000
H	-0.161974000	0.024439000	1.828865000
H	-0.451714000	-4.772986000	0.597671000
H	-1.070875000	-1.603360000	3.442869000
H	-1.247172000	-4.010417000	2.827509000
C	5.163873000	-0.299284000	1.791760000
H	6.007766000	0.345862000	2.053404000
H	4.242263000	0.181236000	2.130266000
H	5.275823000	-1.249245000	2.317627000
C	6.289112000	-1.378532000	-1.861832000
H	5.940674000	-0.503234000	-2.417666000
H	7.271828000	-1.663184000	-2.246020000
H	5.593325000	-2.201251000	-2.035663000
Pd	-0.412989000	1.810579000	-0.504296000
Cl	0.987319000	2.472302000	1.281914000
Cl	-1.869216000	1.212300000	-2.275148000
C	9.914463000	-2.718285000	0.778456000
C	9.854616000	-4.121759000	0.675903000
C	11.117319000	-2.133023000	1.226296000
C	10.960298000	-4.909226000	0.994905000
C	12.219387000	-2.922957000	1.541203000
C	12.148096000	-4.315342000	1.426607000
H	8.944675000	-4.598584000	0.324311000
H	11.179180000	-1.056437000	1.348293000
H	10.892425000	-5.989429000	0.902082000
H	13.134464000	-2.451216000	1.888135000
H	13.008060000	-4.929498000	1.677696000
C	-5.372105000	-2.480963000	1.209860000
C	-4.732207000	-2.376583000	-0.009942000
S	-5.654017000	-3.175614000	-1.261717000
C	-6.910896000	-3.668062000	-0.133939000
C	-6.594261000	-3.230271000	1.127423000
H	-7.238139000	-3.394941000	1.984329000
C	-3.419946000	-1.738721000	-0.341457000
H	-3.540725000	-0.761382000	-0.821914000

H	-2.839011000	-1.588558000	0.569525000
H	-2.834336000	-2.370436000	-1.016307000
C	-3.929072000	-1.955544000	4.686651000
C	-3.959629000	-0.507838000	4.146716000
C	-4.423326000	-0.666221000	2.708753000
C	-4.879460000	-1.918165000	2.474781000
C	-4.857339000	-2.754280000	3.743794000
H	-4.225725000	-2.022952000	5.737092000
H	-2.986430000	-0.006866000	4.216903000
H	-4.671185000	0.124553000	4.697703000
H	-4.503567000	-3.775256000	3.561415000
H	-5.875099000	-2.843947000	4.151659000
H	-2.912029000	-2.352729000	4.606617000
C	-3.238762000	1.292525000	1.603804000
C	-3.400311000	2.317531000	0.702627000
S	-5.020818000	2.303322000	0.052326000
C	-5.465067000	0.913907000	1.009108000
C	-4.403882000	0.479568000	1.785358000
H	-2.297909000	1.090277000	2.102563000
C	-6.850935000	0.355028000	0.913071000
H	-6.941710000	-0.338848000	0.069531000
H	-7.089103000	-0.203126000	1.821774000
H	-7.598347000	1.144107000	0.783117000
P	-2.049584000	3.381949000	0.137038000
C	-2.731463000	4.517400000	-1.126551000
C	-2.146464000	4.603201000	-2.396764000
C	-3.801999000	5.365456000	-0.793686000
C	-2.641601000	5.514276000	-3.332095000
C	-4.300444000	6.263400000	-1.736654000
C	-3.721202000	6.337735000	-3.006927000
H	-1.317806000	3.954271000	-2.655209000
H	-4.237405000	5.333247000	0.200806000
H	-2.184097000	5.576060000	-4.315210000
H	-5.133730000	6.909584000	-1.476576000
H	-4.107501000	7.041762000	-3.738514000
C	-1.681230000	4.519884000	1.527873000
C	-2.311251000	4.426788000	2.774466000
C	-0.726598000	5.525547000	1.304721000
C	-1.991156000	5.335631000	3.787110000
C	-0.414348000	6.430844000	2.316157000
C	-1.045349000	6.336382000	3.561122000
H	-3.052148000	3.655776000	2.956798000
H	-0.229930000	5.600312000	0.341307000
H	-2.486800000	5.259877000	4.750816000
H	0.324242000	7.206547000	2.135656000
H	-0.798921000	7.040906000	4.350368000
C	-8.086524000	-4.435973000	-0.563098000
C	-8.758603000	-5.268321000	0.354041000
C	-8.578479000	-4.364160000	-1.880143000
C	-9.887230000	-5.989024000	-0.031125000
C	-9.700890000	-5.096555000	-2.265201000
C	-10.364107000	-5.909703000	-1.342992000
H	-8.381031000	-5.362252000	1.367636000
H	-8.091417000	-3.717115000	-2.604534000
H	-10.388675000	-6.625100000	0.693218000
H	-10.062489000	-5.023419000	-3.287119000
H	-11.240253000	-6.477346000	-1.642914000

[$PdCl_2$(**DTE^c-Ph**)$_2$]

C	-6.781414000	-2.448113000	-0.515268000

C	-6.680707000	-0.936717000	-0.808150000
S	-8.425308000	-0.305582000	-0.543190000
C	-9.089784000	-1.961952000	-0.659716000
C	-8.125352000	-2.926952000	-0.625406000
H	-8.356509000	-3.985876000	-0.662385000
C	-3.912517000	-4.773463000	-0.578060000
C	-3.244555000	-3.466754000	-0.085309000
C	-4.366616000	-2.455378000	-0.131379000
C	-5.636201000	-3.143692000	-0.277177000
C	-5.402918000	-4.634032000	-0.177123000
H	-3.443682000	-5.670429000	-0.163910000
H	-2.374787000	-3.173613000	-0.680905000
H	-2.891359000	-3.574404000	0.948758000
H	-6.075989000	-5.221581000	-0.808753000
H	-5.550885000	-4.969811000	0.859232000
H	-3.837518000	-4.831057000	-1.669983000
C	-3.158772000	-0.257410000	0.038968000
C	-3.416338000	1.074418000	-0.075751000
S	-5.146586000	1.458347000	-0.294827000
C	-5.625588000	-0.314313000	0.132918000
C	-4.312685000	-1.102092000	-0.018490000
H	-2.153774000	-0.650978000	0.142858000
P	-2.095660000	2.317145000	-0.146505000
C	-2.144952000	2.906900000	-1.882995000
C	-1.653903000	4.183001000	-2.199127000
C	-2.608369000	2.070842000	-2.908362000
C	-1.640541000	4.619311000	-3.523689000
C	-2.595348000	2.513523000	-4.231943000
C	-2.112960000	3.786900000	-4.542216000
H	-1.288987000	4.838668000	-1.414092000
H	-2.983198000	1.079160000	-2.675894000
H	-1.262138000	5.609922000	-3.759243000
H	-2.964682000	1.862237000	-5.018961000
H	-2.104625000	4.129846000	-5.572899000
C	-2.632327000	3.785788000	0.814948000
C	-3.742197000	4.542844000	0.400271000
C	-1.913972000	4.184784000	1.951083000
C	-4.139591000	5.662694000	1.129184000
C	-2.310869000	5.313017000	2.672464000
C	-3.425693000	6.048570000	2.267576000
H	-4.286523000	4.275535000	-0.499723000
H	-1.054194000	3.611537000	2.276689000
H	-5.002147000	6.236834000	0.803672000
H	-1.746968000	5.613082000	3.550996000
H	-3.734854000	6.923988000	2.831654000
C	-6.025084000	-0.333333000	1.625461000
H	-6.264356000	-1.354422000	1.937020000
H	-5.185800000	0.025145000	2.227015000
H	-6.890948000	0.302014000	1.820501000
C	-6.347117000	-0.769480000	-2.309370000
H	-5.352972000	-1.174031000	-2.520528000
H	-7.078976000	-1.320862000	-2.904993000
H	-6.367885000	0.278962000	-2.612054000
Pd	-0.099527000	1.276982000	0.607791000
Cl	0.870058000	1.533615000	-1.522561000
Cl	-1.153164000	0.882457000	2.703716000
C	7.810953000	0.321394000	-1.298868000
C	6.848025000	-0.799498000	-0.863167000
S	7.980809000	-2.147128000	-0.219260000
C	9.389840000	-1.418618000	-1.045174000
C	9.136538000	-0.166955000	-1.526757000
H	9.888409000	0.427573000	-2.034125000
C	6.725828000	3.585769000	-2.637571000
C	5.594853000	3.274607000	-1.627015000
C	5.942946000	1.891747000	-1.121795000
C	7.309355000	1.571921000	-1.491663000
C	7.949115000	2.788624000	-2.120408000
H	6.926052000	4.656416000	-2.733246000
H	4.594255000	3.329071000	-2.067362000
H	5.616758000	3.986508000	-0.790044000
H	8.667069000	2.541401000	-2.908176000
H	8.487441000	3.369633000	-1.357845000
H	6.442339000	3.208262000	-3.626593000
C	3.823547000	1.139317000	0.012650000
C	3.291611000	0.008187000	0.548554000
S	4.423548000	-1.376436000	0.588264000
C	5.865526000	-0.235415000	0.187299000
C	5.193242000	1.024050000	-0.390882000
H	3.245065000	2.044903000	-0.123662000
P	1.689911000	-0.048508000	1.397142000
C	1.115439000	-1.777525000	1.244500000
C	0.450798000	-2.421343000	2.299998000
C	1.230612000	-2.422507000	0.001003000
C	-0.060145000	-3.707592000	2.120937000
C	0.716610000	-3.708475000	-0.170258000
C	0.075823000	-4.354835000	0.889955000
H	0.333157000	-1.922297000	3.255391000
H	1.721425000	-1.924368000	-0.829565000
H	-0.565553000	-4.202668000	2.945009000
H	0.818331000	-4.203245000	-1.131660000
H	-0.323562000	-5.355856000	0.755026000
C	2.212929000	0.226697000	3.131248000
C	2.851006000	-0.775369000	3.878497000
C	2.091900000	1.518819000	3.664011000
C	3.350553000	-0.485335000	5.149522000
C	2.598684000	1.804213000	4.931520000
C	3.226483000	0.802245000	5.676739000
H	2.959425000	-1.776465000	3.475463000
H	1.595146000	2.294597000	3.089995000
H	3.839485000	-1.266567000	5.724354000
H	2.497372000	2.806151000	5.338622000
H	3.617361000	1.024330000	6.665662000
C	6.530793000	0.154372000	1.526855000
H	7.340182000	0.869184000	1.350840000
H	5.787163000	0.625278000	2.175240000
H	6.940675000	-0.715571000	2.043052000
C	6.152534000	-1.342815000	-2.133364000
H	5.510948000	-0.571997000	-2.569871000
H	6.912211000	-1.620381000	-2.868386000
H	5.541326000	-2.220035000	-1.914077000
C	10.658540000	-2.151960000	-1.120738000
C	11.873177000	-1.457235000	-1.300157000
C	10.704016000	-3.555739000	-1.014861000
C	13.080611000	-2.144425000	-1.385740000
C	11.916267000	-4.239764000	-1.097953000
C	13.109622000	-3.539512000	-1.285681000
H	11.870640000	-0.373209000	-1.351130000
H	9.783660000	-4.117598000	-0.887632000
H	14.004497000	-1.588970000	-1.520506000
H	11.925771000	-5.323145000	-1.018919000
H	14.053744000	-4.072876000	-1.347881000
C	-10.540060000	-2.168950000	-0.746273000

C	-11.398661000	-1.165148000	-1.235166000
C	-11.110436000	-3.391399000	-0.333274000
C	-12.773600000	-1.380826000	-1.321462000
C	-12.483169000	-3.603230000	-0.423966000
C	-13.322902000	-2.599975000	-0.919246000
H	-10.986493000	-0.217764000	-1.569210000
H	-10.476276000	-4.168794000	0.080633000
H	-13.415392000	-0.593858000	-1.707280000
H	-12.901325000	-4.550935000	-0.096448000
H	-14.394277000	-2.766343000	-0.984260000

PhI

C	0.000000000	0.000000000	-0.579023000
C	0.000000000	1.218778000	-1.260023000
C	0.000000000	-1.218778000	-1.260023000
C	0.000000000	1.208622000	-2.658171000
C	0.000000000	-1.208622000	-2.658171000
C	0.000000000	0.000000000	-3.357848000
H	0.000000000	2.157824000	-0.717789000
H	0.000000000	-2.157824000	-0.717789000
H	0.000000000	2.152414000	-3.196150000
H	0.000000000	-2.152414000	-3.196150000
H	0.000000000	0.000000000	-4.443889000
I	0.000000000	0.000000000	1.564365000

0 [Pd(**DTE°-COCF$_3$**)$_2$]

Pd	-0.083484000	2.249482000	0.539332000
S	4.961586000	2.411204000	0.881997000
S	7.556792000	-1.511742000	-2.342721000
P	1.956756000	3.231076000	0.209455000
F	11.531232000	-4.212687000	-0.478576000
F	9.545334000	-5.020709000	-0.095870000
F	10.230241000	-3.256594000	0.983435000
O	10.403212000	-2.644497000	-2.259922000
C	2.522114000	4.784745000	1.024381000
C	1.843178000	5.191278000	2.183031000
H	0.993117000	4.611431000	2.535041000
C	2.252149000	6.330093000	2.880602000
H	1.719186000	6.634790000	3.776916000
C	3.338468000	7.077876000	2.420914000
H	3.654364000	7.966783000	2.959756000
C	4.015462000	6.685068000	1.262403000
H	4.858341000	7.266839000	0.899861000
C	3.611241000	5.545017000	0.566367000
H	4.137781000	5.254906000	-0.337912000
C	2.293213000	3.513348000	-1.586838000
C	1.977028000	4.739342000	-2.193720000
H	1.626400000	5.571720000	-1.590095000
C	2.122978000	4.902584000	-3.572892000
H	1.883543000	5.859718000	-4.028138000
C	2.576048000	3.843664000	-4.364468000
H	2.689694000	3.973841000	-5.436937000
C	2.880995000	2.617122000	-3.768435000
H	3.233205000	1.787088000	-4.375210000
C	2.737855000	2.451283000	-2.390061000
H	2.965951000	1.492174000	-1.937618000
C	3.259472000	2.032417000	0.673907000
C	3.066131000	0.681080000	0.814069000
H	2.092145000	0.221354000	0.707266000
C	4.261032000	-0.073578000	1.063329000
C	5.379959000	0.735071000	1.147390000
C	6.797688000	0.357058000	1.450163000
H	6.824476000	-0.606983000	1.963803000
H	7.285749000	1.102073000	2.086611000
H	7.392228000	0.259818000	0.534186000
C	4.229843000	-1.534794000	1.223926000
H	3.472639000	-1.937959000	3.198106000
C	3.365646000	-3.694295000	1.861812000
H	2.608656000	-3.987718000	1.127828000
C	4.766914000	-3.846048000	1.229688000
C	5.053923000	-2.458814000	0.677322000
C	6.138555000	-2.253993000	-0.293669000
C	7.414038000	-2.866732000	-0.144500000
H	7.665138000	-3.502786000	0.695113000
C	8.303944000	-2.559731000	-1.155834000
C	6.060092000	-1.496585000	-1.460036000
C	4.893262000	-0.727605000	-1.990690000
H	3.965000000	-1.127441000	-1.578614000
H	4.842554000	-0.772231000	-3.082985000
H	4.959554000	0.326524000	-1.699523000
C	9.680804000	-2.967703000	-1.328506000
C	10.263671000	-3.881450000	-0.215271000
S	-3.412381000	-1.429581000	-0.986791000
S	-8.400051000	-2.126614000	1.553874000
P	-1.150071000	0.223661000	0.516792000
F	-12.568360000	-3.255005000	-1.259446000
F	-12.150988000	-1.141209000	-0.940919000
F	-10.916416000	-2.300793000	-2.310851000
O	-10.856863000	-3.733580000	0.677050000
C	-0.144405000	-0.963247000	-0.493485000
C	0.485039000	-0.479979000	-1.653742000
H	0.377782000	0.566302000	-1.926751000
C	1.267085000	-1.325298000	-2.440329000
H	1.752014000	-0.935119000	-3.330648000
C	1.439195000	-2.664174000	-2.074362000
H	2.059404000	-3.319584000	-2.679154000
C	0.814146000	-3.153646000	-0.925917000
H	0.936871000	-4.194423000	-0.640397000
C	0.026672000	-2.309229000	-0.136945000
H	-0.446411000	-2.702324000	0.757396000
C	-1.237926000	-0.631579000	2.153118000
C	-0.125963000	-0.497897000	3.004013000
H	0.720930000	0.107242000	2.691223000
C	-0.103053000	-1.133893000	4.245189000
H	0.767317000	-1.029418000	4.887038000
C	-1.199098000	-1.895022000	4.663436000
H	-1.185126000	-2.384286000	5.633244000
C	-2.314101000	-2.017945000	3.832155000
H	-3.171868000	-2.603170000	4.152249000
C	-2.332963000	-1.393992000	2.581356000
H	-3.202344000	-1.507204000	1.943081000
C	-2.809119000	0.021550000	-0.204413000
C	-3.778453000	0.991891000	-0.207683000
H	-3.614910000	1.977555000	0.215683000
C	-5.024669000	0.585068000	-0.792569000
C	-4.974645000	-0.707987000	-1.282348000
C	-6.032420000	-1.484061000	-2.005857000
H	-6.767870000	-0.800533000	-2.436770000
H	-5.606169000	-2.087850000	-2.813398000
H	-6.567107000	-2.161267000	-1.329465000

C	-6.190005000	1.479727000	-0.857326000
C	-6.066015000	2.882446000	-1.427518000
H	-5.911548000	2.819709000	-2.514744000
H	-5.203190000	3.425312000	-1.024694000
C	-7.416176000	3.541121000	-1.071087000
H	-7.752033000	4.261620000	-1.821948000
H	-7.317795000	4.074859000	-0.119317000
C	-8.392608000	2.358228000	-0.888162000
H	-8.930600000	2.128305000	-1.820037000
H	-9.155001000	2.544305000	-0.123768000
C	-7.472183000	1.203830000	-0.524039000
C	-8.009511000	-0.029698000	0.068166000
C	-9.187657000	-0.657514000	-0.423446000
H	-9.750124000	-0.273924000	-1.265463000
C	-9.531754000	-1.810203000	0.256278000
C	-7.477497000	-0.707661000	1.162455000
C	-6.276831000	-0.354340000	1.980363000
H	-6.083592000	0.718887000	1.913896000
H	-6.408427000	-0.626243000	3.031882000
H	-5.385802000	-0.870284000	1.606723000
C	-10.632833000	-2.720631000	0.030832000
C	-11.588426000	-2.354446000	-1.138426000
C	3.222021000	-2.185759000	2.156140000
H	2.203120000	-1.820347000	1.989425000
H	3.226803000	-4.317237000	2.749720000
H	5.525775000	-4.114992000	1.979551000
H	4.805549000	-4.620006000	0.454935000

0 [Pd(**DTE^{o}-COCF$_3$**)(**DTE^{c}-COCF$_3$**)]

C	-6.462111000	-1.967294000	0.035290000
C	-6.159461000	-0.690218000	-0.775028000
S	-7.835801000	-0.147332000	-1.429277000
C	-8.515437000	-1.761352000	-1.081443000
C	-7.701715000	-2.558032000	-0.312742000
H	-7.991534000	-3.541607000	0.037397000
C	-3.938942000	-3.898206000	1.928987000
C	-3.389687000	-2.456070000	2.061493000
C	-4.358350000	-1.636342000	1.238028000
C	-5.526434000	-2.422397000	0.925042000
C	-5.457940000	-3.729503000	1.675160000
H	-3.723754000	-4.516769000	2.804048000
H	-2.357173000	-2.365236000	1.710866000
H	-3.409743000	-2.121504000	3.107778000
H	-5.906850000	-4.565344000	1.131143000
H	-5.997281000	-3.630992000	2.627858000
H	-3.479364000	-4.376854000	1.058097000
C	-3.145081000	0.540513000	0.937932000
C	-3.241829000	1.691910000	0.210821000
S	-4.741161000	1.828337000	-0.725267000
C	-5.472602000	0.333073000	0.157010000
C	-4.260180000	-0.341454000	0.820011000
H	-2.253709000	0.288706000	1.501362000
P	-1.902586000	2.958038000	0.185950000
C	-2.290906000	3.893822000	-1.355037000
C	-2.744145000	5.220246000	-1.369169000
C	-2.068975000	3.231961000	-2.576411000
C	-2.981492000	5.868704000	-2.584394000
C	-2.320066000	3.877813000	-3.785879000
C	-2.775394000	5.200098000	-3.792256000
H	-2.917086000	5.750388000	-0.438481000
H	-1.701667000	2.208573000	-2.575668000
H	-3.332313000	6.896927000	-2.583291000
H	-2.154193000	3.352372000	-4.722179000
H	-2.964524000	5.706326000	-4.734632000
C	-2.393110000	4.073071000	1.566555000
C	-3.727104000	4.438328000	1.817010000
C	-1.379670000	4.552572000	2.410178000
C	-4.035807000	5.271931000	2.891977000
C	-1.691952000	5.391997000	3.482326000
C	-3.019382000	5.750580000	3.725045000
H	-4.522654000	4.075943000	1.173321000
H	-0.348555000	4.259636000	2.228656000
H	-5.069731000	5.547890000	3.079284000
H	-0.899732000	5.758076000	4.129238000
H	-3.263764000	6.398379000	4.562218000
C	-6.384792000	0.872613000	1.283814000
H	-6.797850000	0.040066000	1.860891000
H	-5.796547000	1.501941000	1.956738000
H	-7.211471000	1.461931000	0.884058000
C	-5.287887000	-1.094678000	-1.986884000
H	-4.317404000	-1.465996000	-1.645893000
H	-5.787918000	-1.892322000	-2.541714000
H	-5.121880000	-0.253350000	-2.662368000
Pd	0.051666000	1.775661000	0.335493000
C	-9.838295000	-2.066507000	-1.575326000
O	-10.535305000	-1.295521000	-2.225847000
C	-10.401591000	-3.474289000	-1.245884000
F	-11.625902000	-3.635109000	-1.759575000
F	-9.606265000	-4.448387000	-1.746273000
F	-10.480424000	-3.671660000	0.091437000
S	3.541745000	-2.045357000	-0.347797000
S	8.852385000	-1.116908000	1.552429000
P	1.151206000	-0.205015000	0.671685000
F	12.965190000	-2.535736000	-1.210677000
F	12.126828000	-0.574393000	-1.650063000
F	11.079493000	-2.374700000	-2.289224000
O	11.515098000	-2.520263000	0.981126000
C	0.159253000	-1.668689000	0.128316000
C	-0.785326000	-1.499151000	-0.895802000
H	-0.944601000	-0.510206000	-1.316847000
C	-1.526139000	-2.586819000	-1.363305000
H	-2.251803000	-2.441939000	-2.158223000
C	-1.338708000	-3.853502000	-0.804942000
H	-1.918286000	-4.698664000	-1.165044000
C	-0.408160000	-4.029686000	0.223265000
H	-0.263786000	-5.011230000	0.665743000
C	0.337689000	-2.944858000	0.688113000
H	1.050220000	-3.092718000	1.493998000
C	1.457112000	-0.567224000	2.456950000
C	0.355929000	-0.828343000	3.291046000
H	-0.643625000	-0.888057000	2.868466000
C	0.537638000	-1.024797000	4.659434000
H	-0.320844000	-1.235368000	5.291374000
C	1.818905000	-0.951474000	5.216334000
H	1.959376000	-1.103452000	6.282722000
C	2.915249000	-0.681408000	4.395357000
H	3.914026000	-0.622436000	4.819253000
C	2.736655000	-0.490168000	3.022471000
H	3.595227000	-0.278620000	2.393405000
C	2.765526000	-0.488564000	-0.118743000
C	3.574334000	0.511666000	-0.595252000

H	3.285130000	1.556812000	-0.556063000
C	4.832803000	0.061134000	-1.120588000
C	4.953745000	-1.316230000	-1.072527000
C	6.070217000	-2.183864000	-1.567499000
H	6.664438000	-1.641515000	-2.306699000
H	5.690538000	-3.099457000	-2.032191000
H	6.742826000	-2.477232000	-0.753080000
C	5.833987000	0.994569000	-1.657321000
H	5.212220000	1.562878000	-3.634709000
C	6.683306000	2.957149000	-2.765606000
H	6.576203000	3.790829000	-2.062738000
C	7.851483000	2.057388000	-2.308062000
C	7.167645000	1.023607000	-1.425909000
C	7.959921000	0.188717000	-0.511491000
C	9.201371000	-0.386378000	-0.903128000
H	9.621058000	-0.258474000	-1.893017000
C	9.808827000	-1.134466000	0.086575000
C	7.646137000	-0.108829000	0.812598000
C	6.456492000	0.323505000	1.608095000
H	6.039938000	1.242037000	1.188775000
H	6.712392000	0.496692000	2.657721000
H	5.672904000	-0.441312000	1.572493000
C	11.049834000	-1.877183000	0.051861000
C	11.825324000	-1.843388000	-1.294209000
C	5.437149000	2.051379000	-2.675146000
H	4.530608000	2.595330000	-2.386341000
H	6.833086000	3.380775000	-3.762507000
H	8.336172000	1.559211000	-3.161000000
H	8.636853000	2.607598000	-1.778361000

0 [Pd(**DTE^{c}-COCF$_3$**)$_2$]

C	-5.867033000	-1.507421000	0.942644000
C	-4.939774000	-1.531478000	-0.289751000
S	-5.862227000	-2.583512000	-1.544676000
C	-6.911773000	-3.232815000	-0.255332000
C	-6.808849000	-2.567305000	0.941472000
H	-7.419643000	-2.799700000	1.805747000
C	-5.185859000	0.383232000	4.045764000
C	-4.538549000	1.297534000	2.977450000
C	-4.721173000	0.519038000	1.693972000
C	-5.667237000	-0.552527000	1.901652000
C	-6.264323000	-0.424453000	3.280997000
H	-5.597644000	0.941395000	4.890419000
H	-3.489550000	1.522648000	3.191105000
H	-5.070840000	2.256028000	2.907741000
H	-6.499901000	-1.387674000	3.742160000
H	-7.200381000	0.148637000	3.221352000
H	-4.428791000	-0.306160000	4.434907000
C	-3.114706000	1.630146000	0.114493000
C	-2.587604000	1.444588000	-1.130411000
S	-3.328393000	0.109066000	-2.033725000
C	-4.680579000	-0.076202000	-0.739057000
C	-4.157983000	0.720374000	0.468848000
H	-2.716923000	2.366254000	0.804834000
P	-1.216671000	2.488340000	-1.777379000
C	-0.216946000	1.267811000	-2.736781000
C	0.320091000	1.564717000	-3.997989000
C	0.120456000	0.048822000	-2.121687000
C	1.161690000	0.650606000	-4.639033000
C	0.951304000	-0.864777000	-2.768506000
C	1.474359000	-0.567045000	-4.030771000
H	0.078470000	2.502742000	-4.488122000
H	-0.272538000	-0.186931000	-1.137237000
H	1.568173000	0.891245000	-5.617359000
H	1.189525000	-1.808596000	-2.286827000
H	2.120954000	-1.279386000	-4.534620000
C	-2.070410000	3.509960000	-3.050989000
C	-2.686338000	2.970762000	-4.193433000
C	-2.131468000	4.893520000	-2.827306000
C	-3.352820000	3.804904000	-5.090550000
C	-2.800980000	5.726960000	-3.726890000
C	-3.412706000	5.183051000	-4.857774000
H	-2.638528000	1.904242000	-4.387807000
H	-1.653383000	5.313678000	-1.945613000
H	-3.825794000	3.379903000	-5.971392000
H	-2.841674000	6.797083000	-3.544336000
H	-3.932830000	5.829500000	-5.559107000
C	-5.933457000	0.651023000	-1.280015000
H	-6.737633000	0.614444000	-0.539244000
H	-5.688853000	1.698348000	-1.475187000
H	-6.292230000	0.197256000	-2.205248000
C	-3.650247000	-2.290032000	0.103887000
H	-3.106989000	-1.737221000	0.875382000
H	-3.915908000	-3.271656000	0.503858000
H	-2.989628000	-2.431174000	-0.753574000
Pd	0.000007000	3.246442000	0.000006000
C	5.867002000	-1.507397000	-0.942716000
C	4.940043000	-1.531226000	0.289915000
S	5.862902000	-2.582830000	1.544891000
C	6.912259000	-3.232316000	0.255492000
C	6.808971000	-2.567150000	-0.941470000
H	7.419605000	-2.799685000	-1.805819000
C	5.184796000	0.382230000	-4.046200000
C	4.537466000	1.296680000	-2.978026000
C	4.720612000	0.518659000	-1.694328000
C	5.666841000	-0.552786000	-1.901924000
C	6.263618000	-0.424960000	-3.281423000
H	5.596256000	0.940258000	-4.891102000
H	3.488356000	1.521439000	-3.191527000
H	5.069508000	2.255343000	-2.908743000
H	6.499372000	-1.388242000	-3.742368000
H	7.199529000	0.148404000	-3.222111000
H	4.427828000	-0.307470000	-4.434989000
C	3.114358000	1.629922000	-0.114725000
C	2.587638000	1.444626000	1.130385000
S	3.329086000	0.109660000	2.033958000
C	4.680843000	-0.075852000	0.738883000
C	4.157731000	0.720273000	-0.469109000
H	2.716213000	2.365740000	-0.805166000
P	1.216645000	2.488274000	1.777385000
C	0.216669000	1.267684000	2.736470000
C	-0.321369000	1.565039000	3.997165000
C	-0.120098000	0.048404000	2.121650000
C	-1.163314000	0.651089000	4.637962000
C	-0.951349000	-0.865024000	2.768220000
C	-1.475392000	-0.566853000	4.029961000
H	-0.080288000	2.503322000	4.487073000
H	0.273665000	-0.187733000	1.137602000
H	-1.570543000	0.892076000	5.615893000
H	-1.189120000	-1.809043000	2.286712000
H	-2.122307000	-1.279053000	4.533600000

C	2.070280000	3.509561000	3.051343000
C	2.685168000	2.970143000	4.194243000
C	2.132448000	4.893030000	2.827427000
C	3.351731000	3.803987000	5.091580000
C	2.802050000	5.726169000	3.727222000
C	3.412745000	5.182043000	4.858560000
H	2.636436000	1.903701000	4.388829000
H	1.655150000	5.313356000	1.945386000
H	3.823895000	3.378825000	5.972777000
H	2.843606000	6.796227000	3.544484000
H	3.932927000	5.828259000	5.560066000
C	5.933834000	0.651656000	1.279186000
H	6.737764000	0.614922000	0.538156000
H	5.689201000	1.699019000	1.474111000
H	6.292963000	0.198206000	2.204438000
C	3.650467000	-2.289985000	-0.103181000
H	3.106963000	-1.737405000	-0.874667000
H	3.916093000	-3.271690000	-0.502973000
H	2.990100000	-2.430948000	0.754504000
C	7.790116000	-4.334732000	0.579380000
O	7.861409000	-4.868126000	1.680466000
C	-7.789403000	-4.335441000	-0.579106000
O	-7.860438000	-4.869104000	-1.680082000
C	-8.707413000	-4.857230000	0.558066000
F	-9.445525000	-5.892475000	0.143937000
F	-7.983259000	-5.260504000	1.627902000
F	-9.548972000	-3.888153000	0.988539000
C	8.707963000	-4.856703000	-0.557843000
F	9.446462000	-5.891594000	-0.143520000
F	7.983628000	-5.260570000	-1.627325000
F	9.549149000	-3.887568000	-0.988909000

1 [Pd(**DTE^{o}-COCF$_3$**)$_2$]·**PhI**

Pd	-0.241699000	1.560811000	1.068463000
S	4.505321000	2.399895000	-0.650597000
S	7.781285000	-1.873024000	-2.632086000
P	1.331927000	2.117657000	-0.555171000
F	12.620980000	-2.660286000	-1.046123000
F	11.042150000	-3.597749000	0.125034000
F	11.365289000	-1.447092000	0.255757000
O	10.806294000	-2.269431000	-2.905853000
C	1.549194000	3.880339000	-1.041877000
C	1.983903000	4.781444000	-0.053038000
H	2.245000000	4.412655000	0.935145000
C	2.075891000	6.144994000	-0.328778000
H	2.419552000	6.828706000	0.442595000
C	1.719428000	6.631078000	-1.591193000
H	1.784271000	7.694314000	-1.804314000
C	1.281975000	5.743328000	-2.576381000
H	1.008976000	6.113168000	-3.560978000
C	1.198794000	4.374678000	-2.306242000
H	0.861456000	3.695507000	-3.082741000
C	0.971147000	1.246508000	-2.143187000
C	-0.376308000	1.119806000	-2.523140000
H	-1.150546000	1.587725000	-1.922082000
C	-0.728935000	0.374468000	-3.648469000
H	-1.775688000	0.278627000	-3.922990000
C	0.260039000	-0.263917000	-4.403113000
H	-0.014395000	-0.859408000	-5.269291000
C	1.602404000	-0.135917000	-4.037071000
H	2.376494000	-0.627461000	-4.620158000
C	1.957193000	0.618911000	-2.916297000
H	3.001540000	0.699352000	-2.635658000
C	3.027658000	1.603570000	-0.139286000
C	3.329800000	0.477650000	0.583337000
H	2.566148000	-0.174159000	0.988775000
C	4.734038000	0.214541000	0.717745000
C	5.508269000	1.193696000	0.119856000
C	6.999456000	1.334352000	0.091685000
H	7.444561000	0.758025000	0.906231000
H	7.305772000	2.379776000	0.199880000
H	7.422189000	0.959217000	-0.847866000
C	5.227097000	-0.966715000	1.440166000
H	4.987188000	-0.576606000	3.542798000
C	5.234639000	-2.723822000	3.092162000
H	4.510189000	-3.480170000	2.773890000
C	6.491496000	-2.824159000	2.201367000
C	6.211922000	-1.826497000	1.087410000
C	7.004173000	-1.866719000	-0.150349000
C	8.410610000	-2.085361000	-0.133508000
H	8.973770000	-2.210469000	0.782823000
C	8.990077000	-2.104122000	-1.387366000
C	6.514004000	-1.751177000	-1.449465000
C	5.102950000	-1.550106000	-1.898317000
H	4.411971000	-1.885054000	-1.123875000
H	4.891310000	-2.096678000	-2.822418000
H	4.903390000	-0.488720000	-2.081160000
C	10.376136000	-2.280263000	-1.761874000
C	11.374368000	-2.500668000	-0.591864000
S	-2.713519000	-2.774495000	0.044174000
S	-8.126555000	-2.183016000	1.361117000
P	-0.636897000	-0.733421000	1.348009000
F	-11.794099000	-4.074753000	-1.725944000
F	-11.150361000	-2.031470000	-2.119927000
F	-9.852900000	-3.702321000	-2.640506000
O	-10.569862000	-3.862013000	0.588754000
C	0.562297000	-2.013365000	0.771107000
C	0.892178000	-1.997380000	-0.596591000
H	0.413011000	-1.284477000	-1.260155000
C	1.830244000	-2.890358000	-1.109260000
H	2.071576000	-2.861936000	-2.167761000
C	2.464275000	-3.806963000	-0.263175000
H	3.202921000	-4.497078000	-0.660670000
C	2.141764000	-3.829435000	1.094511000
H	2.617800000	-4.547827000	1.755700000
C	1.195571000	-2.938661000	1.612517000
H	0.951494000	-2.977597000	2.668837000
C	-1.012489000	-1.177686000	3.091993000
C	-0.020074000	-0.900873000	4.050803000
H	0.950556000	-0.529935000	3.730867000
C	-0.275239000	-1.081531000	5.409422000
H	0.504120000	-0.870910000	6.136432000
C	-1.536263000	-1.512055000	5.834640000
H	-1.740664000	-1.639944000	6.893810000
C	-2.533645000	-1.768325000	4.891242000
H	-3.517357000	-2.098367000	5.214009000
C	-2.274169000	-1.607096000	3.527276000
H	-3.061376000	-1.808263000	2.808506000
C	-2.119879000	-1.157063000	0.384743000
C	-2.922677000	-0.229684000	-0.230079000
H	-2.732675000	0.835957000	-0.168724000

C	-4.035084000	-0.782360000	-0.943789000
C	-4.052621000	-2.164076000	-0.898904000
C	-5.026491000	-3.111756000	-1.528733000
H	-5.564187000	-2.609198000	-2.336307000
H	-4.523527000	-3.992022000	-1.941719000
H	-5.770289000	-3.461894000	-0.803313000
C	-5.018915000	0.079942000	-1.613992000
C	-4.580557000	1.148100000	-2.601008000
H	-4.240666000	0.667073000	-3.530357000
H	-3.737467000	1.742575000	-2.230473000
C	-5.859290000	1.985655000	-2.817640000
H	-5.937961000	2.393888000	-3.828997000
H	-5.859222000	2.828470000	-2.116967000
C	-7.019743000	1.030178000	-2.457972000
H	-7.402194000	0.504851000	-3.345850000
H	-7.874215000	1.544150000	-2.004568000
C	-6.366324000	0.038631000	-1.508269000
C	-7.173757000	-0.841320000	-0.650550000
C	-8.309703000	-1.544653000	-1.138225000
H	-8.641860000	-1.481325000	-2.166891000
C	-8.933485000	-2.327596000	-0.185704000
C	-6.959300000	-1.075887000	0.705923000
C	-5.904282000	-0.498712000	1.595621000
H	-5.559697000	0.456341000	1.191961000
H	-6.276388000	-0.339302000	2.611983000
H	-5.034436000	-1.162670000	1.654528000
C	-10.086559000	-3.191858000	-0.311827000
C	-10.738253000	-3.255613000	-1.720998000
C	4.657801000	-1.321958000	2.804160000
H	3.561889000	-1.307160000	2.813818000
H	5.445993000	-2.890029000	4.152103000
H	7.400632000	-2.531616000	2.747478000
H	6.667473000	-3.836445000	1.820743000
C	-1.374766000	3.444693000	1.674808000
C	-0.542432000	4.417377000	2.299096000
C	-1.930759000	2.375523000	2.442945000
C	-0.207995000	4.273360000	3.632736000
C	-1.585105000	2.285793000	3.823694000
C	-0.729414000	3.202731000	4.400887000
H	-0.162214000	5.248658000	1.716662000
H	-2.781015000	1.810326000	2.077315000
H	0.453136000	5.000745000	4.096160000
H	-2.024499000	1.487610000	4.414829000
H	-0.477457000	3.123176000	5.454578000
I	-2.448960000	4.082754000	-0.126859000

1 [Pd(**DTE^{o}-COCF$_3$**)(**DTE^{c}-COCF$_3$**)]·**PhI**

C	5.878329000	2.926747000	0.325862000
C	5.808240000	1.667376000	-0.563121000
S	7.523893000	1.536981000	-1.319377000
C	7.862597000	3.236206000	-0.887821000
C	6.942488000	3.790961000	-0.031171000
H	7.035148000	4.792223000	0.372164000
C	3.099607000	4.147928000	2.433898000
C	2.897555000	2.616073000	2.515569000
C	3.960212000	2.078235000	1.583612000
C	4.918585000	3.114195000	1.284150000
C	4.605939000	4.331900000	2.118847000
H	2.791439000	4.667938000	3.344595000
H	1.888640000	2.298111000	2.240708000
H	3.078667000	2.252160000	3.536116000
H	4.837294000	5.274167000	1.614400000
H	5.195482000	4.302003000	3.046144000
H	2.508162000	4.546453000	1.601582000
C	3.180355000	-0.259425000	1.113819000
C	3.472462000	-1.312759000	0.298391000
S	4.962710000	-1.094618000	-0.641536000
C	5.399358000	0.464977000	0.316481000
C	4.102007000	0.827876000	1.057566000
H	2.274466000	-0.222588000	1.704075000
P	2.403533000	-2.814315000	0.252197000
C	2.602075000	-3.413934000	-1.478002000
C	2.609967000	-4.791100000	-1.755711000
C	2.555081000	-2.506386000	-2.549905000
C	2.589931000	-5.247743000	-3.074766000
C	2.541729000	-2.966222000	-3.867068000
C	2.558547000	-4.337769000	-4.134851000
H	2.640919000	-5.509930000	-0.941994000
H	2.523286000	-1.439089000	-2.358223000
H	2.603157000	-6.315895000	-3.273314000
H	2.516624000	-2.250737000	-4.684555000
H	2.548669000	-4.694609000	-5.160783000
C	3.431008000	-3.979054000	1.254110000
C	4.688022000	-4.453568000	0.843944000
C	2.927410000	-4.369012000	2.504544000
C	5.423773000	-5.301071000	1.672540000
C	3.667533000	-5.214651000	3.334989000
C	4.916344000	-5.681057000	2.919486000
H	5.088184000	-4.169137000	-0.124061000
H	1.952730000	-4.005560000	2.822345000
H	6.394142000	-5.664528000	1.345800000
H	3.267258000	-5.510004000	4.300950000
H	5.492744000	-6.340810000	3.562012000
C	6.454139000	0.068052000	1.375262000
H	6.704985000	0.931931000	1.997843000
H	6.045406000	-0.716186000	2.017811000
H	7.369218000	-0.302215000	0.910154000
C	4.806887000	1.953331000	-1.706332000
H	3.800671000	2.092169000	-1.303601000
H	5.101435000	2.868850000	-2.225516000
H	4.779540000	1.136597000	-2.430109000
Pd	0.214531000	-2.399108000	0.783118000
C	9.058115000	3.852326000	-1.415391000
O	9.858147000	3.299164000	-2.161979000
C	9.341514000	5.314840000	-0.980282000
F	10.458020000	5.779659000	-1.551395000
F	8.323017000	6.138290000	-1.322604000
F	9.490372000	5.403358000	0.362589000
S	-3.129424000	0.170448000	-1.062895000
S	-6.913554000	4.126215000	-0.243550000
P	-0.795890000	-0.327308000	0.973284000
F	-11.458211000	3.890398000	-2.662399000
F	-11.342374000	2.906433000	-0.723019000
F	-10.362442000	2.019391000	-2.454940000
O	-9.205592000	5.090078000	-2.032517000
C	-0.018242000	0.841878000	-0.246563000
C	0.396147000	0.295433000	-1.473224000
H	0.262297000	-0.766631000	-1.658542000
C	0.991731000	1.097741000	-2.447164000
H	1.304829000	0.656402000	-3.389362000
C	1.200796000	2.457672000	-2.202224000

H	1.679848000	3.080630000	-2.952088000
C	0.799415000	3.009162000	-0.983288000
H	0.961828000	4.064738000	-0.782986000
C	0.186062000	2.209895000	-0.014553000
H	-0.121310000	2.661512000	0.922002000
C	-0.676664000	0.551253000	2.594513000
C	0.218070000	0.043972000	3.550085000
H	0.773563000	-0.864254000	3.329390000
C	0.395480000	0.692966000	4.774765000
H	1.094936000	0.290176000	5.501970000
C	-0.328504000	1.851743000	5.062661000
H	-0.191993000	2.357791000	6.014220000
C	-1.239086000	2.353250000	4.127080000
H	-1.813186000	3.248106000	4.351142000
C	-1.416164000	1.706259000	2.903495000
H	-2.134080000	2.096698000	2.188967000
C	-2.570528000	-0.105535000	0.573619000
C	-3.638881000	-0.326429000	1.403996000
H	-3.528383000	-0.524040000	2.464435000
C	-4.918454000	-0.234786000	0.756367000
C	-4.806656000	0.003957000	-0.599985000
C	-5.885394000	0.146184000	-1.630232000
H	-6.814216000	-0.294150000	-1.259779000
H	-5.616205000	-0.355190000	-2.565856000
H	-6.088453000	1.198367000	-1.863014000
C	-6.182334000	-0.354686000	1.498552000
H	-6.643710000	-2.439094000	1.750382000
C	-7.741698000	-1.142073000	3.155724000
H	-7.452452000	-0.689362000	4.110612000
C	-8.407267000	-0.066659000	2.267978000
C	-7.240514000	0.487415000	1.465832000
C	-7.354875000	1.761713000	0.741213000
C	-8.497456000	2.092363000	-0.038684000
H	-9.343647000	1.426337000	-0.152670000
C	-8.417677000	3.328739000	-0.650875000
C	-6.410240000	2.785748000	0.739523000
C	-5.099097000	2.845805000	1.457772000
H	-5.119712000	2.182266000	2.325162000
H	-4.870824000	3.861946000	1.793490000
H	-4.282634000	2.511782000	0.807527000
C	-9.358988000	3.986300000	-1.530303000
C	-10.656013000	3.193602000	-1.852017000
C	-6.470295000	-1.555562000	2.382565000
H	-5.628730000	-1.805074000	3.039196000
H	-8.402185000	-1.985427000	3.375385000
H	-9.153912000	-0.505997000	1.589745000
H	-8.925080000	0.706774000	2.845758000
C	-1.831854000	-3.845677000	-0.722884000
C	-0.913372000	-4.518828000	0.105399000
C	-3.085765000	-3.445564000	-0.255981000
C	-1.266406000	-4.729374000	1.452650000
C	-3.426475000	-3.698700000	1.075736000
C	-2.512989000	-4.314618000	1.934852000
H	-0.013917000	-4.960362000	-0.308218000
H	-3.779888000	-2.927910000	-0.908007000
H	-0.571959000	-5.253503000	2.102664000
H	-4.400277000	-3.390155000	1.440639000
H	-2.774907000	-4.491649000	2.973908000
I	-1.279520000	-3.463695000	-2.749377000

1 [Pd(**DTE^{c}-COCF$_3$**)$_2$]·**PhI**

C	6.232804000	-1.777405000	-0.928220000
C	5.133993000	-2.287108000	0.027766000
S	5.936607000	-3.726483000	0.932148000
C	7.216796000	-3.814474000	-0.307392000
C	7.243704000	-2.743354000	-1.165903000
H	7.994584000	-2.618628000	-1.936923000
C	5.872007000	1.142338000	-3.164079000
C	5.014707000	1.575291000	-1.949813000
C	5.062688000	0.366926000	-1.040959000
C	6.108155000	-0.530383000	-1.476032000
C	6.888772000	0.124241000	-2.588928000
H	6.356189000	1.984843000	-3.664235000
H	3.994580000	1.863636000	-2.221513000
H	5.466252000	2.438680000	-1.442397000
H	7.265869000	-0.585650000	-3.330319000
H	7.756402000	0.650434000	-2.166460000
H	5.235073000	0.637424000	-3.899397000
C	3.141509000	0.753735000	0.525448000
C	2.436312000	0.085709000	1.483255000
S	3.142477000	-1.470888000	1.951650000
C	4.692819000	-1.121879000	0.941792000
C	4.305458000	0.073077000	0.053114000
H	2.793111000	1.687228000	0.096200000
P	0.831655000	0.691783000	2.130107000
C	-0.066391000	-0.905268000	2.368208000
C	-0.249625000	-1.533339000	3.609199000
C	-0.566893000	-1.523578000	1.210433000
C	-0.930256000	-2.752124000	3.686651000
C	-1.237036000	-2.743260000	1.288993000
C	-1.425665000	-3.358991000	2.530147000
H	0.139461000	-1.081236000	4.515299000
H	-0.423338000	-1.051467000	0.245479000
H	-1.069001000	-3.227746000	4.653441000
H	-1.612936000	-3.208887000	0.382439000
H	-1.953077000	-4.306335000	2.594735000
C	1.204264000	1.264382000	3.835463000
C	2.510669000	1.487549000	4.292557000
C	0.120783000	1.653509000	4.643402000
C	2.728872000	2.083917000	5.537347000
C	0.342207000	2.235010000	5.891025000
C	1.648189000	2.457071000	6.339815000
H	3.360011000	1.203632000	3.678816000
H	-0.897677000	1.504961000	4.292791000
H	3.746090000	2.251123000	5.880609000
H	-0.503912000	2.525502000	6.507490000
H	1.820999000	2.919426000	7.307409000
C	5.785388000	-0.644940000	1.926288000
H	6.698094000	-0.390102000	1.379638000
H	5.437257000	0.248782000	2.450962000
H	6.024615000	-1.412734000	2.663750000
C	3.983084000	-2.855706000	-0.835091000
H	3.507148000	-2.056405000	-1.408740000
H	4.384924000	-3.593437000	-1.534223000
H	3.220619000	-3.338271000	-0.220988000
Pd	0.100845000	2.394489000	0.686424000
C	-6.416750000	-1.974096000	0.485657000
C	-5.699597000	-1.529045000	-0.807048000
S	-7.025267000	-1.636879000	-2.134394000
C	-8.028411000	-2.663228000	-1.073067000
C	-7.596447000	-2.717938000	0.228874000
H	-8.128441000	-3.247591000	1.009960000

C	-4.825565000	-2.081212000	3.813547000
C	-4.112521000	-0.866849000	3.171396000
C	-4.678766000	-0.832073000	1.770130000
C	-5.845527000	-1.681852000	1.692954000
C	-6.184557000	-2.181207000	3.075194000
H	-4.939912000	-1.984787000	4.896221000
H	-3.023041000	-0.955399000	3.187949000
H	-4.373329000	0.062027000	3.697322000
H	-6.613684000	-3.187015000	3.079530000
H	-6.919920000	-1.507387000	3.537209000
H	-4.239833000	-2.985675000	3.615814000
C	-3.022452000	0.576246000	0.522672000
C	-2.709476000	0.911304000	-0.760961000
S	-3.953274000	0.467298000	-1.948987000
C	-5.120650000	-0.113521000	-0.589439000
C	-4.245660000	-0.146043000	0.675688000
H	-2.350616000	0.773290000	1.349491000
P	-1.107956000	1.690019000	-1.185831000
C	-0.178779000	0.380860000	-2.100267000
C	1.152625000	0.675036000	-2.447978000
C	-0.679794000	-0.902685000	-2.364956000
C	1.953993000	-0.284730000	-3.065480000
C	0.133152000	-1.868638000	-2.964518000
C	1.447538000	-1.562927000	-3.320569000
H	1.563018000	1.656375000	-2.227682000
H	-1.697133000	-1.163148000	-2.096068000
H	2.978246000	-0.040967000	-3.330601000
H	-0.266834000	-2.860909000	-3.153380000
H	2.076940000	-2.315676000	-3.786229000
C	-1.610479000	2.895014000	-2.486880000
C	-1.119155000	2.882998000	-3.799235000
C	-2.474555000	3.930704000	-2.089404000
C	-1.480858000	3.892861000	-4.695177000
C	-2.841265000	4.931185000	-2.988031000
C	-2.339246000	4.918421000	-4.293612000
H	-0.457093000	2.089140000	-4.128890000
H	-2.857546000	3.952910000	-1.072343000
H	-1.094779000	3.871794000	-5.710607000
H	-3.514035000	5.722482000	-2.669105000
H	-2.619007000	5.701256000	-4.992792000
C	-6.191031000	0.985565000	-0.405514000
H	-6.872899000	0.714319000	0.405795000
H	-5.702128000	1.928345000	-0.146531000
H	-6.775159000	1.133194000	-1.315483000
C	-4.619173000	-2.588354000	-1.133380000
H	-3.850234000	-2.598810000	-0.355098000
H	-5.082173000	-3.577319000	-1.173460000
H	-4.141731000	-2.389438000	-2.094676000
C	-9.201486000	-3.292701000	-1.638694000
O	-9.557727000	-3.180004000	-2.805567000
C	8.122345000	-4.942040000	-0.276882000
O	8.066979000	-5.857832000	0.535241000
C	9.246047000	-4.966743000	-1.346819000
F	9.995035000	-6.068436000	-1.232502000
F	8.733194000	-4.936511000	-2.598874000
F	10.059370000	-3.891827000	-1.222649000
C	-10.061344000	-4.159675000	-0.680505000
F	-11.101807000	-4.699646000	-1.323554000
F	-9.332364000	-5.165360000	-0.143184000
F	-10.541394000	-3.421819000	0.348033000
C	0.597854000	4.592885000	0.404623000
C	-0.566426000	5.413205000	0.452951000
C	1.244768000	4.199393000	1.615853000
C	-1.102488000	5.765624000	1.677768000
C	0.682784000	4.623040000	2.855384000
C	-0.474704000	5.374808000	2.886392000
H	-1.029548000	5.738634000	-0.471502000
H	2.261196000	3.820422000	1.603521000
H	-2.006760000	6.367659000	1.707521000
H	1.195257000	4.352930000	3.773719000
H	-0.891462000	5.693813000	3.837472000
I	1.812045000	4.722949000	-1.415867000

TS1 [Pd(**DTE^{o}-COCF$_3$**)$_2$]·**PhI**

Pd	-0.134768000	2.012621000	0.584561000
S	4.512281000	2.047059000	-1.499964000
S	7.414080000	-2.869905000	-2.158850000
P	1.316171000	2.068250000	-1.275992000
F	12.171608000	-3.713540000	-0.363721000
F	10.525453000	-4.093137000	1.010891000
F	11.085799000	-2.045618000	0.521137000
O	10.371918000	-3.665989000	-2.278315000
C	1.644988000	3.528824000	-2.355502000
C	2.394364000	4.603326000	-1.843471000
H	2.831618000	4.533920000	-0.853473000
C	2.582738000	5.762421000	-2.596622000
H	3.174228000	6.577211000	-2.187796000
C	2.011206000	5.876613000	-3.866627000
H	2.155049000	6.780607000	-4.451610000
C	1.254225000	4.820015000	-4.379101000
H	0.810423000	4.897023000	-5.368067000
C	1.072635000	3.654183000	-3.632156000
H	0.491575000	2.838933000	-4.051861000
C	0.847538000	0.752243000	-2.489811000
C	-0.520913000	0.540968000	-2.726009000
H	-1.253803000	1.169634000	-2.229530000
C	-0.948029000	-0.497264000	-3.555869000
H	-2.010546000	-0.657352000	-3.715208000
C	-0.010318000	-1.344974000	-4.152326000
H	-0.340593000	-2.166199000	-4.782175000
C	1.353972000	-1.136229000	-3.930349000
H	2.088107000	-1.791325000	-4.391563000
C	1.781399000	-0.091258000	-3.108521000
H	2.842827000	0.048669000	-2.934120000
C	2.994881000	1.575433000	-0.749467000
C	3.239858000	0.692392000	0.271908000
H	2.445695000	0.257051000	0.865684000
C	4.623621000	0.358611000	0.457039000
C	5.447625000	1.045333000	-0.416410000
C	6.943067000	1.044035000	-0.505045000
H	7.371981000	0.683980000	0.433118000
H	7.333630000	2.047854000	-0.701235000
H	7.300242000	0.385677000	-1.305841000
C	5.050835000	-0.598874000	1.486634000
H	4.914743000	0.414078000	3.379809000
C	4.990757000	-1.782472000	3.589442000
H	4.198969000	-2.535852000	3.534287000
C	6.194739000	-2.252509000	2.745427000
C	5.950775000	-1.606565000	1.389310000
C	6.697829000	-2.078556000	0.213768000
C	8.072117000	-2.440588000	0.303229000

H	8.640212000	-2.367982000	1.222014000
C	8.617719000	-2.875867000	-0.888634000
C	6.194656000	-2.278524000	-1.070792000
C	4.805678000	-2.059149000	-1.576056000
H	4.098674000	-2.075600000	-0.747041000
H	4.515660000	-2.824835000	-2.302051000
H	4.718464000	-1.082428000	-2.064454000
C	9.967396000	-3.304541000	-1.183024000
C	10.960386000	-3.292171000	0.012213000
S	-2.696600000	-2.409926000	-0.027084000
S	-8.054060000	-2.284985000	1.612846000
P	-0.752243000	-0.194923000	1.210892000
F	-11.722135000	-4.488505000	-1.261270000
F	-11.269041000	-2.401097000	-1.688041000
F	-9.872047000	-3.963632000	-2.284324000
O	-10.384839000	-4.171680000	0.980198000
C	0.406022000	-1.617591000	0.972499000
C	0.685418000	-2.040470000	-0.339487000
H	0.195525000	-1.563105000	-1.179675000
C	1.576673000	-3.086198000	-0.572860000
H	1.772840000	-3.399776000	-1.593895000
C	2.216571000	-3.719398000	0.496748000
H	2.915572000	-4.530439000	0.313798000
C	1.953793000	-3.298747000	1.801413000
H	2.436650000	-3.791737000	2.640006000
C	1.056247000	-2.254100000	2.040946000
H	0.857016000	-1.951824000	3.063563000
C	-1.242331000	-0.368479000	2.984218000
C	-0.427923000	0.269019000	3.936273000
H	0.447171000	0.824195000	3.607545000
C	-0.733191000	0.197373000	5.295958000
H	-0.090204000	0.690616000	6.019852000
C	-1.870633000	-0.495691000	5.721578000
H	-2.117438000	-0.542007000	6.778457000
C	-2.690209000	-1.124796000	4.781683000
H	-3.575633000	-1.665824000	5.104958000
C	-2.375360000	-1.068773000	3.421535000
H	-3.015802000	-1.574072000	2.707144000
C	-2.211426000	-0.745890000	0.267195000
C	-3.124136000	0.106848000	-0.298144000
H	-3.019369000	1.183356000	-0.251725000
C	-4.235937000	-0.539001000	-0.931031000
C	-4.136898000	-1.917643000	-0.883657000
C	-5.061216000	-2.946925000	-1.458419000
H	-5.682814000	-2.495811000	-2.235738000
H	-4.509123000	-3.783929000	-1.898098000
H	-5.732645000	-3.354975000	-0.693621000
C	-5.331668000	0.234009000	-1.533496000
C	-5.050685000	1.344117000	-2.531550000
H	-4.718505000	0.904479000	-3.483854000
H	-4.245845000	2.010994000	-2.200870000
C	-6.411485000	2.061669000	-2.668136000
H	-6.585023000	2.463490000	-3.670243000
H	-6.448986000	2.899837000	-1.963222000
C	-7.457136000	1.003535000	-2.249655000
H	-7.844301000	0.449367000	-3.117783000
H	-8.325491000	1.436585000	-1.741080000
C	-6.661729000	0.071444000	-1.349262000
C	-7.337902000	-0.874715000	-0.449657000
C	-8.434613000	-1.675761000	-0.872016000
H	-8.830001000	-1.645811000	-1.879773000
C	-8.932689000	-2.505535000	0.114614000
C	-7.027943000	-1.082262000	0.892742000
C	-5.980377000	-0.408585000	1.720677000
H	-5.741919000	0.569535000	1.296293000
H	-6.307111000	-0.274739000	2.756174000
H	-5.054166000	-0.992708000	1.733215000
C	-10.013128000	-3.465256000	0.054421000
C	-10.737116000	-3.586898000	-1.315049000
C	4.506832000	-0.488565000	2.901686000
H	3.415872000	-0.383950000	2.919395000
H	5.238696000	-1.638079000	4.644647000
H	7.147705000	-1.895776000	3.163757000
H	6.271881000	-3.343635000	2.679704000
C	-0.217852000	4.130772000	1.463874000
C	0.182432000	5.215076000	0.659305000
C	0.483770000	3.808758000	2.642253000
C	1.340957000	5.908594000	0.990341000
C	1.664737000	4.505837000	2.936414000
C	2.094262000	5.551045000	2.119464000
H	-0.389962000	5.480981000	-0.222576000
H	0.101514000	3.062057000	3.327675000
H	1.666498000	6.725247000	0.352095000
H	2.221233000	4.247700000	3.833732000
H	2.992876000	6.107030000	2.370462000
I	-2.435798000	3.604234000	1.442635000

TS1 [Pd(**DTE^{o}-COCF$_3$**)(**DTE^{c}-COCF$_3$**)]·**PhI**

C	5.545231000	3.180579000	0.579883000
C	5.556429000	2.056515000	-0.476367000
S	7.293694000	2.110344000	-1.191989000
C	7.534731000	3.748728000	-0.526314000
C	6.569891000	4.137255000	0.371351000
H	6.603153000	5.076215000	0.911141000
C	2.684931000	3.951166000	2.796222000
C	2.569052000	2.414164000	2.663134000
C	3.656796000	2.071578000	1.670505000
C	4.562092000	3.186552000	1.532360000
C	4.179598000	4.258140000	2.524161000
H	2.347063000	4.321249000	3.767624000
H	1.577674000	2.079351000	2.348440000
H	2.777910000	1.924243000	3.623720000
H	4.359990000	5.272519000	2.157546000
H	4.767331000	4.131786000	3.444412000
H	2.073281000	4.430136000	2.022780000
C	3.021893000	-0.231170000	0.898459000
C	3.382673000	-1.148018000	-0.044039000
S	4.829906000	-0.697538000	-0.967298000
C	5.186961000	0.724221000	0.212602000
C	3.868521000	0.914769000	0.980667000
H	2.134482000	-0.340082000	1.506964000
P	2.480731000	-2.737555000	-0.226317000
C	2.871019000	-3.261675000	-1.943288000
C	3.158921000	-4.601602000	-2.244663000
C	2.700228000	-2.346793000	-2.996524000
C	3.297415000	-5.011703000	-3.572254000
C	2.844705000	-2.759669000	-4.321371000
C	3.144461000	-4.092942000	-4.613856000
H	3.278501000	-5.327202000	-1.446002000
H	2.454375000	-1.311481000	-2.784071000
H	3.526729000	-6.050887000	-3.790903000

H	2.720665000	-2.038333000	-5.124415000
H	3.255056000	-4.414135000	-5.645584000
C	3.525333000	-3.814278000	0.849494000
C	4.879657000	-4.070925000	0.575317000
C	2.947495000	-4.349935000	2.010015000
C	5.636432000	-4.852687000	1.448095000
C	3.709217000	-5.130082000	2.884188000
C	5.053398000	-5.382333000	2.604099000
H	5.340449000	-3.665814000	-0.319803000
H	1.900075000	-4.157329000	2.224756000
H	6.681895000	-5.047981000	1.226505000
H	3.250481000	-5.541409000	3.779094000
H	5.646294000	-5.990787000	3.281341000
C	6.245226000	0.219038000	1.221699000
H	6.447572000	0.991503000	1.969593000
H	5.863943000	-0.668762000	1.733181000
H	7.182047000	-0.038593000	0.725177000
C	4.570280000	2.456578000	-1.597212000
H	3.549874000	2.485653000	-1.208888000
H	4.829025000	3.450970000	-1.969802000
H	4.600765000	1.753843000	-2.431950000
Pd	0.150427000	-2.710414000	0.123484000
C	8.710627000	4.484684000	-0.931849000
O	9.562490000	4.065607000	-1.707448000
C	8.891975000	5.903060000	-0.329164000
F	10.006556000	6.482145000	-0.788431000
F	7.845682000	6.703578000	-0.639518000
F	8.971267000	5.856088000	1.021751000
S	-3.245714000	-0.098327000	-1.423685000
S	-6.619473000	4.241070000	-0.477369000
P	-0.804283000	-0.571968000	0.483098000
F	-11.404348000	4.405295000	-2.384358000
F	-11.160877000	3.493894000	-0.421317000
F	-10.505800000	2.429109000	-2.205700000
O	-8.979006000	5.369170000	-2.072773000
C	-0.050542000	0.676548000	-0.662965000
C	0.255876000	0.223556000	-1.957509000
H	0.076450000	-0.816107000	-2.218377000
C	0.805775000	1.089102000	-2.904650000
H	1.034045000	0.720461000	-3.900814000
C	1.076192000	2.416689000	-2.565300000
H	1.518725000	3.088429000	-3.295127000
C	0.787567000	2.872729000	-1.276584000
H	1.004796000	3.901237000	-1.001450000
C	0.223818000	2.011685000	-0.332200000
H	0.008523000	2.389589000	0.659921000
C	-0.638897000	0.128025000	2.184356000
C	0.119094000	-0.591506000	3.121116000
H	0.567003000	-1.538072000	2.829060000
C	0.297813000	-0.103843000	4.418525000
H	0.890266000	-0.671261000	5.130537000
C	-0.288017000	1.105426000	4.797018000
H	-0.149634000	1.486671000	5.804727000
C	-1.064704000	1.819804000	3.879030000
H	-1.534023000	2.754582000	4.172559000
C	-1.245557000	1.332254000	2.584634000
H	-1.869912000	1.882070000	1.887797000
C	-2.585771000	-0.294363000	0.188454000
C	-3.602585000	-0.390179000	1.103480000
H	-3.429689000	-0.522019000	2.164797000
C	-4.917096000	-0.249973000	0.542024000
C	-4.892223000	-0.102727000	-0.830636000
C	-6.033939000	0.077775000	-1.783125000
H	-6.962324000	-0.262553000	-1.318075000
H	-5.882611000	-0.489364000	-2.707398000
H	-6.167560000	1.131521000	-2.056211000
C	-6.124672000	-0.229123000	1.381419000
H	-6.707049000	-2.261863000	1.775786000
C	-7.612864000	-0.818611000	3.181134000
H	-7.225455000	-0.357858000	4.096500000
C	-8.253112000	0.275336000	2.296710000
C	-7.109939000	0.697609000	1.387321000
C	-7.174171000	1.952118000	0.623384000
C	-8.352647000	2.368437000	-0.056120000
H	-9.262721000	1.781509000	-0.067298000
C	-8.222145000	3.577410000	-0.712676000
C	-6.146583000	2.885752000	0.500726000
C	-4.777652000	2.851579000	1.104331000
H	-4.781185000	2.219354000	1.995065000
H	-4.436869000	3.853543000	1.382479000
H	-4.049117000	2.428769000	0.403472000
C	-9.185913000	4.304211000	-1.509640000
C	-10.590974000	3.653034000	-1.637048000
C	-6.432686000	-1.361333000	2.344843000
H	-5.564323000	-1.634506000	2.955703000
H	-8.320526000	-1.597958000	3.477006000
H	-9.083003000	-0.123956000	1.694926000
H	-8.660128000	1.111479000	2.875956000
C	-1.500843000	-4.085183000	0.381120000
C	-0.807389000	-4.921345000	1.282335000
C	-2.803635000	-3.635354000	0.670905000
C	-1.366695000	-5.154733000	2.551010000
C	-3.343027000	-3.902138000	1.923584000
C	-2.621292000	-4.644367000	2.875533000
H	0.121173000	-5.401900000	0.996270000
H	-3.350733000	-3.047013000	-0.056032000
H	-0.824367000	-5.771758000	3.263364000
H	-4.331348000	-3.520584000	2.163911000
H	-3.058533000	-4.849355000	3.848928000
I	-0.968569000	-4.386961000	-1.895184000

TS1 [Pd(**DTE^c-COCF$_3$**)$_2$]·**PhI**

C	6.724757000	-1.397836000	-0.720981000
C	5.466068000	-2.060352000	-0.125785000
S	6.109488000	-3.626054000	0.693707000
C	7.633654000	-3.503090000	-0.226149000
C	7.802816000	-2.305519000	-0.876293000
H	8.699194000	-2.049783000	-1.428483000
C	6.771496000	1.868333000	-2.459956000
C	5.654718000	2.090279000	-1.410225000
C	5.534193000	0.739905000	-0.738277000
C	6.674675000	-0.078000000	-1.077583000
C	7.662402000	0.751684000	-1.860482000
H	7.331576000	2.779984000	-2.683032000
H	4.713423000	2.435039000	-1.849850000
H	5.958535000	2.845132000	-0.671910000
H	8.210926000	0.178090000	-2.612913000
H	8.402816000	1.182968000	-1.171942000
H	6.327359000	1.510477000	-3.395809000
C	3.346925000	0.903984000	0.487460000
C	2.480625000	0.105052000	1.173366000

S	3.070077000	-1.555985000	1.409538000
C	4.793197000	-1.060015000	0.841562000
C	4.573464000	0.275135000	0.109821000
H	3.099826000	1.921476000	0.208163000
P	0.843313000	0.692011000	1.759683000
C	-0.084520000	-0.900458000	1.862664000
C	-0.635073000	-1.394101000	3.053902000
C	-0.280709000	-1.618054000	0.669879000
C	-1.370021000	-2.583867000	3.049405000
C	-1.004670000	-2.808279000	0.670645000
C	-1.556875000	-3.292846000	1.861188000
H	-0.489502000	-0.860046000	3.987344000
H	0.139653000	-1.248426000	-0.258847000
H	-1.790777000	-2.956911000	3.978970000
H	-1.144924000	-3.350982000	-0.259775000
H	-2.128711000	-4.216331000	1.860514000
C	1.136265000	1.111496000	3.533454000
C	2.183018000	0.578407000	4.301376000
C	0.229898000	1.997468000	4.137177000
C	2.321503000	0.935037000	5.644299000
C	0.362707000	2.342121000	5.483367000
C	1.412702000	1.814347000	6.238586000
H	2.885951000	-0.120689000	3.861606000
H	-0.580607000	2.416775000	3.548014000
H	3.137862000	0.518648000	6.227710000
H	-0.347367000	3.027529000	5.937358000
H	1.523488000	2.087592000	7.284115000
C	5.624883000	-0.760457000	2.112248000
H	6.627795000	-0.424477000	1.832280000
H	5.141255000	0.036284000	2.683489000
H	5.718371000	-1.642088000	2.748152000
C	4.555409000	-2.485410000	-1.299843000
H	4.196249000	-1.603690000	-1.834138000
H	5.125860000	-3.106299000	-1.995265000
H	3.689317000	-3.053782000	-0.955719000
Pd	-0.001894000	2.424908000	0.384539000
C	-6.362498000	-1.999428000	0.634801000
C	-5.570550000	-1.888621000	-0.684523000
S	-6.833871000	-2.292153000	-2.017640000
C	-7.914533000	-3.018892000	-0.797735000
C	-7.547180000	-2.768030000	0.500688000
H	-8.129075000	-3.087698000	1.356883000
C	-4.978014000	-1.276696000	3.981789000
C	-4.224830000	-0.245736000	3.107960000
C	-4.683620000	-0.579470000	1.706598000
C	-5.852753000	-1.427063000	1.766642000
C	-6.285725000	-1.570700000	3.204704000
H	-5.161495000	-0.921436000	4.998956000
H	-3.139143000	-0.297708000	3.222945000
H	-4.533282000	0.778279000	3.361018000
H	-6.720693000	-2.548306000	3.430060000
H	-7.045556000	-0.810988000	3.436591000
H	-4.383631000	-2.195217000	4.046114000
C	-2.969770000	0.543485000	0.255794000
C	-2.581199000	0.594496000	-1.050630000
S	-3.702617000	-0.238638000	-2.154510000
C	-4.966842000	-0.469867000	-0.779869000
C	-4.174509000	-0.183802000	0.506854000
H	-2.376022000	0.987019000	1.047396000
P	-1.085900000	1.541655000	-1.546116000
C	0.047642000	0.359454000	-2.402275000
C	1.389332000	0.767072000	-2.512151000
C	-0.321586000	-0.894435000	-2.912087000
C	2.332599000	-0.046956000	-3.139365000
C	0.631187000	-1.720202000	-3.513952000
C	1.956836000	-1.297864000	-3.635447000
H	1.695449000	1.721237000	-2.092447000
H	-1.343826000	-1.245299000	-2.828806000
H	3.362662000	0.287867000	-3.220619000
H	0.332751000	-2.694450000	-3.890702000
H	2.694453000	-1.942710000	-4.104206000
C	-1.749409000	2.551903000	-2.943860000
C	-0.972660000	2.842389000	-4.076953000
C	-3.012486000	3.154076000	-2.817286000
C	-1.454189000	3.706274000	-5.063034000
C	-3.496491000	4.005971000	-3.810134000
C	-2.718286000	4.287637000	-4.936028000
H	0.005984000	2.388880000	-4.200114000
H	-3.620182000	2.959193000	-1.938945000
H	-0.842731000	3.916312000	-5.936432000
H	-4.481042000	4.452448000	-3.700253000
H	-3.093969000	4.953677000	-5.707613000
C	-6.019658000	0.652064000	-0.941088000
H	-6.756980000	0.593615000	-0.134948000
H	-5.524589000	1.625110000	-0.885530000
H	-6.541687000	0.579341000	-1.896421000
C	-4.506529000	-3.009846000	-0.688091000
H	-3.781511000	-2.843275000	0.112379000
H	-4.994402000	-3.973180000	-0.519759000
H	-3.971400000	-3.052695000	-1.638561000
C	-9.079193000	-3.743506000	-1.258455000
O	-9.375931000	-3.907583000	-2.435566000
C	8.557329000	-4.614611000	-0.172940000
O	8.363633000	-5.651981000	0.450066000
C	9.887597000	-4.458782000	-0.957500000
F	10.645001000	-5.554624000	-0.841680000
F	9.658875000	-4.244737000	-2.274202000
F	10.603427000	-3.404391000	-0.500671000
C	-10.012600000	-4.335417000	-0.168837000
F	-11.035960000	-4.996099000	-0.720209000
F	-9.344322000	-5.193165000	0.636402000
F	-10.519979000	-3.359860000	0.620638000
C	-0.515330000	4.639283000	0.363090000
C	-1.091870000	5.188157000	-0.797512000
C	-1.196699000	4.675197000	1.595475000
C	-2.395438000	5.668036000	-0.739495000
C	-2.519181000	5.144586000	1.621467000
C	-3.119110000	5.637071000	0.463466000
H	-0.540878000	5.191990000	-1.731402000
H	-0.703038000	4.374918000	2.512079000
H	-2.854557000	6.052408000	-1.645385000
H	-3.058774000	5.154285000	2.565312000
H	-4.134169000	6.022241000	0.495313000
I	1.802373000	4.680782000	0.494138000

2 [Pd(**DTE°-COCF$_3$**)$_2$(**Ph**)**I**]

Pd	-0.343606000	2.026645000	0.770067000
S	4.481153000	2.415087000	-0.800882000
S	7.454096000	-2.204009000	-2.324179000
P	1.313603000	2.332523000	-0.823513000
F	12.035487000	-3.673141000	-0.478780000

F	10.281194000	-4.293288000	0.653246000
F	10.937125000	-2.216386000	0.710848000
O	10.382656000	-3.089608000	-2.437422000
C	1.637257000	4.022383000	-1.468571000
C	2.302050000	4.952510000	-0.651266000
H	2.653008000	4.661244000	0.331864000
C	2.489652000	6.262729000	-1.085310000
H	2.996770000	6.973318000	-0.439533000
C	2.016156000	6.661777000	-2.338534000
H	2.158176000	7.685232000	-2.673612000
C	1.358948000	5.741808000	-3.156644000
H	0.993082000	6.043385000	-4.133974000
C	1.168296000	4.426684000	-2.726258000
H	0.654419000	3.724830000	-3.374073000
C	0.935478000	1.353804000	-2.331790000
C	-0.406174000	1.265824000	-2.737083000
H	-1.181510000	1.747561000	-2.149573000
C	-0.749580000	0.547780000	-3.883205000
H	-1.791363000	0.478094000	-4.182037000
C	0.244256000	-0.092669000	-4.629229000
H	-0.022586000	-0.660934000	-5.515570000
C	1.580597000	-0.006178000	-4.229736000
H	2.355251000	-0.506733000	-4.803358000
C	1.927959000	0.715577000	-3.086610000
H	2.966818000	0.767428000	-2.781060000
C	2.938665000	1.788919000	-0.239800000
C	3.144615000	0.748882000	0.628494000
H	2.334508000	0.215709000	1.103447000
C	4.519623000	0.395516000	0.819387000
C	5.373705000	1.239545000	0.131726000
C	6.870789000	1.254383000	0.112381000
H	7.258828000	0.698415000	0.968812000
H	7.261617000	2.276030000	0.155222000
H	7.265234000	0.784116000	-0.795861000
C	4.889935000	-0.776586000	1.623796000
H	4.691494000	-0.286625000	3.709908000
C	4.634907000	-2.457870000	3.329305000
H	3.825075000	-3.119996000	3.003054000
C	5.894120000	-2.742746000	2.484545000
C	5.768281000	-1.763413000	1.325157000
C	6.582334000	-1.965902000	0.115657000
C	7.935567000	-2.402691000	0.206441000
H	8.434477000	-2.576713000	1.151476000
C	8.556535000	-2.567848000	-1.015507000
C	6.173537000	-1.828568000	-1.209939000
C	4.843185000	-1.416747000	-1.753042000
H	4.069616000	-1.533625000	-0.996236000
H	4.565170000	-2.011729000	-2.627758000
H	4.863334000	-0.364800000	-2.057176000
C	9.911098000	-2.978750000	-1.315550000
C	10.813016000	-3.297907000	-0.091149000
S	-2.633925000	-2.514130000	-0.499143000
S	-7.756950000	-2.175352000	1.583559000
P	-0.536350000	-0.453396000	0.710025000
F	-11.598919000	-4.402711000	-1.034673000
F	-11.166711000	-2.325359000	-1.524683000
F	-9.808454000	-3.902022000	-2.169287000
O	-10.126885000	-4.063173000	1.116371000
C	0.640488000	-1.689585000	-0.018427000
C	0.629704000	-1.921491000	-1.403596000
H	-0.041897000	-1.362325000	-2.044022000
C	1.458647000	-2.892035000	-1.966529000
H	1.428722000	-3.060376000	-3.039010000
C	2.315728000	-3.642015000	-1.158402000
H	2.958358000	-4.399622000	-1.597451000
C	2.353018000	-3.401057000	0.216233000
H	3.023085000	-3.970177000	0.853679000
C	1.522680000	-2.432763000	0.784139000
H	1.555658000	-2.277023000	1.856251000
C	-0.620952000	-1.029116000	2.456659000
C	0.284483000	-0.444519000	3.357996000
H	0.949712000	0.346283000	3.022627000
C	0.325840000	-0.859862000	4.688880000
H	1.034236000	-0.402955000	5.373903000
C	-0.554251000	-1.847658000	5.139296000
H	-0.532122000	-2.164685000	6.177937000
C	-1.467253000	-2.421084000	4.251340000
H	-2.158991000	-3.183742000	4.597614000
C	-1.497093000	-2.020172000	2.913361000
H	-2.212220000	-2.476921000	2.238519000
C	-2.084628000	-0.886731000	-0.125553000
C	-2.986584000	0.029109000	-0.606919000
H	-2.854860000	1.096779000	-0.485295000
C	-4.138856000	-0.544498000	-1.232622000
C	-4.089134000	-1.926271000	-1.259268000
C	-5.082947000	-2.890928000	-1.829087000
H	-5.715623000	-2.382717000	-2.560820000
H	-4.590105000	-3.734991000	-2.321939000
H	-5.738793000	-3.295040000	-1.049227000
C	-5.237989000	0.282617000	-1.751820000
C	-4.990114000	1.386667000	-2.763332000
H	-4.732939000	0.941464000	-3.736185000
H	-4.146784000	2.028886000	-2.482342000
C	-6.336829000	2.143115000	-2.809063000
H	-6.568019000	2.544242000	-3.799710000
H	-6.300232000	2.985290000	-2.109564000
C	-7.383424000	1.119352000	-2.311385000
H	-7.854117000	0.579076000	-3.146295000
H	-8.195101000	1.581287000	-1.739086000
C	-6.554643000	0.159933000	-1.473769000
C	-7.180359000	-0.776521000	-0.528213000
C	-8.303712000	-1.576288000	-0.872815000
H	-8.765165000	-1.550305000	-1.852098000
C	-8.735444000	-2.401382000	0.148962000
C	-6.777506000	-0.980019000	0.790407000
C	-5.675743000	-0.302158000	1.541820000
H	-5.451347000	0.664745000	1.087090000
H	-5.941806000	-0.142356000	2.590907000
H	-4.753402000	-0.892831000	1.518833000
C	-9.814499000	-3.363590000	0.163777000
C	-10.616544000	-3.505114000	-1.159676000
C	4.263376000	-1.000256000	2.990718000
H	3.181475000	-0.829648000	2.992092000
H	4.788330000	-2.624010000	4.398996000
H	6.815207000	-2.538774000	3.050455000
H	5.958601000	-3.782277000	2.144776000
C	-0.307906000	4.044125000	1.026202000
C	-1.020436000	4.909847000	0.185260000
C	0.430962000	4.586672000	2.086472000
C	-0.985011000	6.292666000	0.392338000
C	0.467031000	5.970947000	2.293387000
C	-0.237115000	6.828618000	1.444550000

H	-1.597640000	4.514291000	-0.646769000
H	0.983730000	3.935525000	2.759693000
H	-1.538856000	6.950266000	-0.273492000
H	1.047555000	6.375819000	3.119220000
H	-0.205366000	7.903294000	1.602147000
I	-2.473850000	2.112015000	2.549407000

2 [Pd(**DTE**^{o}-**COCF$_3$**)(**DTE**^{c}-**COCF$_3$**)(**Ph**)**I**]

C	5.343246000	3.316404000	0.681905000
C	5.293300000	2.325674000	-0.499000000
S	7.004080000	2.435726000	-1.270519000
C	7.306909000	3.974431000	-0.420168000
C	6.382786000	4.272036000	0.550896000
H	6.456714000	5.138850000	1.196758000
C	2.592060000	3.855647000	3.099851000
C	2.446929000	2.345712000	2.794045000
C	3.480539000	2.112438000	1.715524000
C	4.397341000	3.226232000	1.666588000
C	4.076776000	4.177506000	2.793896000
H	2.306894000	4.112303000	4.123252000
H	1.436314000	2.057965000	2.492410000
H	2.695082000	1.744406000	3.679243000
H	4.255248000	5.226056000	2.539831000
H	4.704681000	3.938784000	3.663895000
H	1.952013000	4.428224000	2.418630000
C	2.807715000	-0.106563000	0.752399000
C	3.111839000	-0.916330000	-0.299086000
S	4.479969000	-0.341426000	-1.275211000
C	4.922770000	0.927315000	0.043043000
C	3.647390000	1.039571000	0.892715000
H	1.988944000	-0.302559000	1.430488000
P	2.323927000	-2.561233000	-0.521211000
C	2.867252000	-3.073060000	-2.197848000
C	3.415098000	-4.343471000	-2.432166000
C	2.642725000	-2.212190000	-3.285670000
C	3.743309000	-4.737739000	-3.730420000
C	2.975679000	-2.610586000	-4.580942000
C	3.525099000	-3.874724000	-4.806687000
H	3.585741000	-5.025030000	-1.606965000
H	2.221522000	-1.226357000	-3.125803000
H	4.170280000	-5.722358000	-3.897750000
H	2.806771000	-1.930361000	-5.410853000
H	3.783022000	-4.184755000	-5.815265000
C	3.376942000	-3.500551000	0.664118000
C	4.768623000	-3.591905000	0.491930000
C	2.790181000	-4.038462000	1.818597000
C	5.554860000	-4.217300000	1.459692000
C	3.581568000	-4.659226000	2.787617000
C	4.963592000	-4.749781000	2.609566000
H	5.237503000	-3.176638000	-0.394137000
H	1.714207000	-3.986574000	1.951302000
H	6.629143000	-4.287505000	1.315225000
H	3.114986000	-5.078069000	3.674681000
H	5.578647000	-5.235565000	3.361732000
C	6.015119000	0.286764000	0.933129000
H	6.268285000	0.961262000	1.756494000
H	5.639832000	-0.649667000	1.354088000
H	6.922108000	0.075544000	0.364811000
C	4.277246000	2.871665000	-1.526432000
H	3.274211000	2.874877000	-1.095246000
H	4.545429000	3.896861000	-1.793642000
H	4.258727000	2.268404000	-2.435969000
Pd	-0.062254000	-2.939623000	-0.072948000
C	8.488458000	4.729968000	-0.774092000
O	9.301614000	4.391868000	-1.625916000
C	8.728641000	6.055866000	-0.004490000
F	9.842056000	6.662971000	-0.427865000
F	7.694142000	6.912111000	-0.171637000
F	8.852034000	5.833840000	1.325325000
S	-3.232156000	-0.089065000	-1.528549000
S	-6.214030000	4.331728000	-0.352520000
P	-0.853424000	-0.802066000	0.357659000
F	-10.875598000	4.959512000	-2.456451000
F	-10.801679000	3.935924000	-0.535140000
F	-10.133225000	2.915330000	-2.340304000
O	-8.404575000	5.718784000	-1.980823000
C	-0.100436000	0.492197000	-0.713872000
C	0.171431000	0.135040000	-2.042650000
H	0.018578000	-0.890044000	-2.368923000
C	0.640364000	1.089400000	-2.947526000
H	0.847689000	0.801104000	-3.973910000
C	0.848174000	2.405091000	-2.529866000
H	1.221989000	3.146489000	-3.229656000
C	0.583801000	2.763525000	-1.205060000
H	0.751684000	3.783879000	-0.872686000
C	0.109536000	1.814347000	-0.299043000
H	-0.089162000	2.110840000	0.723808000
C	-0.642440000	-0.301937000	2.111507000
C	0.162392000	-1.095053000	2.944701000
H	0.624716000	-1.993965000	2.547276000
C	0.372290000	-0.734598000	4.277072000
H	0.999944000	-1.355612000	4.909288000
C	-0.228945000	0.415569000	4.792513000
H	-0.067476000	0.695714000	5.829330000
C	-1.047709000	1.200735000	3.975395000
H	-1.525843000	2.090125000	4.375199000
C	-1.259458000	0.844459000	2.643326000
H	-1.914142000	1.449396000	2.025213000
C	-2.618715000	-0.482836000	0.063399000
C	-3.651106000	-0.597472000	0.956810000
H	-3.505653000	-0.851678000	1.999105000
C	-4.940769000	-0.316468000	0.397234000
C	-4.880029000	-0.048814000	-0.957157000
C	-5.992201000	0.278076000	-1.905503000
H	-6.946744000	-0.040688000	-1.480500000
H	-5.859230000	-0.222743000	-2.869884000
H	-6.056000000	1.356205000	-2.094249000
C	-6.158110000	-0.266275000	1.221687000
H	-6.945727000	-2.252617000	1.429706000
C	-7.720721000	-0.854211000	2.953051000
H	-7.300412000	-0.505535000	3.902995000
C	-8.244247000	0.361818000	2.155543000
C	-7.053892000	0.744593000	1.291343000
C	-6.993173000	2.046625000	0.610156000
C	-8.106974000	2.589318000	-0.089176000
H	-9.059073000	2.078863000	-0.164229000
C	-7.853361000	3.812453000	-0.679654000
C	-5.890401000	2.897006000	0.571854000
C	-4.550093000	2.717758000	1.213878000
H	-4.629087000	2.016621000	2.047308000
H	-4.154283000	3.667439000	1.586905000

H	-3.825445000	2.306876000	0.501861000
C	-8.719305000	4.648277000	-1.482308000
C	-10.159406000	4.112060000	-1.711761000
C	-6.589892000	-1.440732000	2.079960000
H	-5.763110000	-1.861432000	2.662738000
H	-8.502891000	-1.582342000	3.184810000
H	-9.098503000	0.091219000	1.517251000
H	-8.580040000	1.184395000	2.796513000
C	-1.957646000	-3.490918000	0.437491000
C	-2.270985000	-3.715466000	1.784393000
C	-2.955163000	-3.670113000	-0.531084000
C	-3.560998000	-4.115095000	2.157589000
C	-4.242769000	-4.065663000	-0.157862000
C	-4.550420000	-4.290546000	1.187776000
H	-1.517643000	-3.573799000	2.555557000
H	-2.735808000	-3.497649000	-1.580950000
H	-3.788170000	-4.287200000	3.207250000
H	-5.006403000	-4.198083000	-0.920601000
H	-5.551883000	-4.596800000	1.476453000
I	0.328313000	-5.607050000	-0.663396000

2 [Pd(**DTE^{c}-COCF$_3$**)$_2$(**Ph**)**I**]

C	7.036532000	-0.992411000	-0.653370000
C	5.789906000	-1.829156000	-0.302607000
S	6.485524000	-3.455123000	0.335330000
C	8.071515000	-3.080239000	-0.389086000
C	8.198834000	-1.787541000	-0.830889000
H	9.117198000	-1.386494000	-1.242467000
C	6.958154000	2.494121000	-1.876549000
C	5.727976000	2.467933000	-0.936223000
C	5.666031000	1.026901000	-0.481003000
C	6.906761000	0.359202000	-0.807905000
C	7.885799000	1.372811000	-1.345607000
H	7.449875000	3.469605000	-1.903781000
H	4.806721000	2.803295000	-1.423167000
H	5.886064000	3.120974000	-0.067506000
H	8.560323000	0.964719000	-2.103421000
H	8.507173000	1.751236000	-0.521777000
H	6.643477000	2.247924000	-2.896985000
C	3.356052000	0.826345000	0.483824000
C	2.503213000	-0.136626000	0.932875000
S	3.231335000	-1.759638000	1.019281000
C	4.944854000	-1.046131000	0.727398000
C	4.673993000	0.363855000	0.176255000
H	3.049197000	1.853993000	0.333619000
P	0.748119000	0.193283000	1.318925000
C	-0.058507000	-1.390905000	0.817979000
C	-0.984873000	-2.031162000	1.656379000
C	0.231430000	-1.958947000	-0.433334000
C	-1.609817000	-3.209893000	1.248604000
C	-0.399627000	-3.135268000	-0.839162000
C	-1.322297000	-3.763855000	-0.001375000
H	-1.209282000	-1.625274000	2.635773000
H	0.960048000	-1.497801000	-1.088167000
H	-2.321615000	-3.694521000	1.910764000
H	-0.163242000	-3.558002000	-1.811085000
H	-1.813824000	-4.678960000	-0.318419000
C	0.585051000	0.145049000	3.151403000
C	1.450747000	-0.580437000	3.981236000
C	-0.503105000	0.826031000	3.721123000
C	1.229450000	-0.620157000	5.359531000
C	-0.732806000	0.767167000	5.095973000
C	0.136270000	0.046060000	5.918578000
H	2.296313000	-1.114484000	3.562272000
H	-1.161437000	1.419669000	3.094449000
H	1.911982000	-1.177014000	5.995147000
H	-1.580266000	1.295589000	5.523015000
H	-0.034822000	0.007952000	6.990552000
C	5.620665000	-0.888187000	2.109741000
H	6.609652000	-0.435214000	1.993812000
H	5.011984000	-0.233094000	2.738354000
H	5.735319000	-1.849946000	2.612127000
C	5.036074000	-2.129295000	-1.619613000
H	4.650171000	-1.203837000	-2.054901000
H	5.724861000	-2.587207000	-2.333728000
H	4.198077000	-2.809941000	-1.459223000
Pd	0.006509000	2.397664000	0.479112000
C	-6.481993000	-1.891913000	0.618747000
C	-5.702824000	-1.714070000	-0.700093000
S	-7.002562000	-1.926429000	-2.041701000
C	-8.105884000	-2.693588000	-0.870890000
C	-7.712313000	-2.578376000	0.437728000
H	-8.302839000	-2.938743000	1.271307000
C	-5.025063000	-1.533764000	3.996341000
C	-4.216686000	-0.483302000	3.197475000
C	-4.706447000	-0.671613000	1.779100000
C	-5.925813000	-1.451527000	1.786225000
C	-6.353341000	-1.686495000	3.213920000
H	-5.180283000	-1.246687000	5.039198000
H	-3.134796000	-0.600694000	3.300797000
H	-4.461987000	0.533193000	3.535453000
H	-6.843623000	-2.652030000	3.365634000
H	-7.064755000	-0.905113000	3.516165000
H	-4.488557000	-2.489278000	3.986742000
C	-2.963721000	0.500325000	0.401038000
C	-2.598844000	0.643872000	-0.904072000
S	-3.757283000	-0.060759000	-2.054429000
C	-5.018251000	-0.329962000	-0.682800000
C	-4.194617000	-0.198431000	0.610027000
H	-2.351468000	0.864446000	1.216943000
P	-1.143130000	1.632412000	-1.390268000
C	-0.025559000	0.619516000	-2.435900000
C	1.343556000	0.928407000	-2.384933000
C	-0.465594000	-0.412452000	-3.277656000
C	2.260727000	0.203796000	-3.147060000
C	0.455986000	-1.143557000	-4.028011000
C	1.819364000	-0.842398000	-3.960155000
H	1.692482000	1.723208000	-1.733338000
H	-1.515602000	-0.673514000	-3.337465000
H	3.316455000	0.451201000	-3.095595000
H	0.107023000	-1.953011000	-4.662350000
H	2.533856000	-1.419399000	-4.539965000
C	-1.868928000	2.836756000	-2.571301000
C	-1.226964000	3.147743000	-3.777309000
C	-3.052343000	3.507306000	-2.223735000
C	-1.762835000	4.121217000	-4.623742000
C	-3.582092000	4.477257000	-3.071513000
C	-2.937505000	4.787870000	-4.272923000
H	-0.313397000	2.636393000	-4.061668000
H	-3.552053000	3.280601000	-1.287704000
H	-1.259617000	4.354727000	-5.557666000

H	-4.495951000	4.993292000	-2.792236000
H	-3.351075000	5.546145000	-4.931584000
C	-6.003521000	0.861021000	-0.736335000
H	-6.728446000	0.783632000	0.079319000
H	-5.450022000	1.796325000	-0.620344000
H	-6.545169000	0.891902000	-1.682859000
C	-4.707987000	-2.889990000	-0.819042000
H	-3.963402000	-2.831382000	-0.022946000
H	-5.248709000	-3.834738000	-0.722264000
H	-4.186687000	-2.882837000	-1.777973000
C	-9.312540000	-3.318104000	-1.375947000
O	-9.629393000	-3.357694000	-2.557306000
C	9.078120000	-4.120967000	-0.407006000
O	8.915143000	-5.252003000	0.033123000
C	10.453678000	-3.754979000	-1.025695000
F	11.288882000	-4.798234000	-0.991184000
F	10.319637000	-3.366153000	-2.314826000
F	11.033426000	-2.730779000	-0.357665000
C	-10.254282000	-3.973354000	-0.330610000
F	-11.319165000	-4.523215000	-0.922156000
F	-9.611983000	-4.940499000	0.363497000
F	-10.695421000	-3.061860000	0.567123000
C	-0.698117000	4.249168000	0.015771000
C	-0.110008000	5.075180000	-0.950620000
C	-1.826154000	4.713524000	0.706746000
C	-0.644678000	6.337951000	-1.227734000
C	-2.362049000	5.976664000	0.428488000
C	-1.774972000	6.791567000	-0.542333000
H	0.761697000	4.736404000	-1.504737000
H	-2.297154000	4.096491000	1.468785000
H	-0.178965000	6.963728000	-1.985365000
H	-3.238815000	6.320411000	0.972628000
H	-2.193137000	7.769974000	-0.762677000
I	1.563462000	3.690307000	2.356527000

3 [Pd(**DTE^{o}-COCF$_3$**)(**Ph**)**I**]

Pd	3.189422000	0.987559000	0.149111000
S	-0.754063000	-1.417469000	1.671046000
S	-5.504563000	-1.548380000	-1.481938000
P	2.051393000	-0.988878000	0.311279000
F	-10.073351000	-0.397986000	0.607978000
F	-8.919588000	1.271761000	-0.181912000
F	-8.308451000	0.306235000	1.673094000
O	-8.441524000	-1.971969000	-0.722562000
C	2.532280000	-2.067042000	1.708186000
C	3.134919000	-1.476036000	2.830482000
H	3.351784000	-0.410792000	2.830313000
C	3.459111000	-2.254430000	3.941324000
H	3.928593000	-1.792057000	4.804565000
C	3.186929000	-3.625321000	3.938094000
H	3.444213000	-4.231505000	4.801904000
C	2.587433000	-4.217294000	2.823887000
H	2.374798000	-5.282304000	2.819625000
C	2.259252000	-3.443715000	1.709096000
H	1.797127000	-3.912347000	0.846155000
C	1.992524000	-2.066555000	-1.166774000
C	3.116626000	-2.843943000	-1.493884000
H	3.991221000	-2.840016000	-0.851228000
C	3.111966000	-3.622175000	-2.650706000
H	3.984490000	-4.220353000	-2.896419000
C	1.995615000	-3.626234000	-3.492284000
H	1.996526000	-4.232369000	-4.393627000
C	0.880480000	-2.848395000	-3.173543000
H	0.009457000	-2.848342000	-3.822631000
C	0.876556000	-2.068673000	-2.015206000
H	0.007515000	-1.465279000	-1.775256000
C	0.347775000	-0.501162000	0.666780000
C	-0.269579000	0.628003000	0.184211000
H	0.229157000	1.339394000	-0.466308000
C	-1.638187000	0.765154000	0.587723000
C	-2.041800000	-0.266478000	1.418839000
C	-3.363790000	-0.472240000	2.091782000
H	-3.897285000	0.478643000	2.159637000
H	-3.242067000	-0.875383000	3.101990000
H	-3.995694000	-1.167724000	1.527509000
C	-2.464938000	1.902383000	0.157228000
H	-1.934052000	3.597472000	1.368509000
C	-2.984193000	4.163558000	-0.487504000
H	-2.629036000	4.296132000	-1.515383000
C	-4.263357000	3.298586000	-0.501819000
C	-3.729464000	1.885511000	-0.324468000
C	-4.579751000	0.724682000	-0.624802000
C	-5.945361000	0.669527000	-0.227685000
H	-6.427985000	1.464482000	0.326861000
C	-6.587904000	-0.495581000	-0.597778000
C	-4.199634000	-0.410637000	-1.336167000
C	-2.872837000	-0.725771000	-1.950498000
H	-2.321030000	0.197773000	-2.139588000
H	-2.983771000	-1.270171000	-2.893132000
H	-2.269578000	-1.342479000	-1.274809000
C	-7.951672000	-0.914710000	-0.354891000
C	-8.835416000	0.079068000	0.448728000
C	-1.955455000	3.326753000	0.302685000
H	-0.930827000	3.447654000	-0.067421000
H	-3.143703000	5.157539000	-0.061025000
H	-4.933141000	3.551219000	0.333629000
H	-4.849820000	3.412631000	-1.419910000
I	4.154324000	3.486636000	0.078645000
C	4.717995000	-0.019807000	-0.590855000
C	5.585461000	-0.724108000	0.249702000
C	4.919033000	-0.009366000	-1.975341000
C	6.662567000	-1.426337000	-0.306735000
C	5.998926000	-0.714701000	-2.517720000
C	6.868854000	-1.427153000	-1.688021000
H	5.423727000	-0.750910000	1.322339000
H	4.244659000	0.531648000	-2.631765000
H	7.333169000	-1.977184000	0.347891000
H	6.153401000	-0.706162000	-3.593577000
H	7.701598000	-1.978394000	-2.115433000

3 [Pd(**DTE^{c}-COCF$_3$**)(**Ph**)**I**]

Pd	3.068166000	0.977277000	0.309906000
C	-4.458699000	0.960711000	-0.233822000
C	-3.646062000	-0.186717000	0.398642000
S	-4.854960000	-1.624444000	0.479128000
C	-6.223735000	-0.507318000	0.242732000
C	-5.861533000	0.762157000	-0.127675000
H	-6.576160000	1.541736000	-0.362549000
C	-3.110159000	4.299180000	-1.057398000
C	-1.872918000	3.398736000	-1.295581000

C	-2.353422000	2.025096000	-0.883846000
C	-3.796446000	2.031412000	-0.762757000
C	-4.326526000	3.358638000	-1.244310000
H	-3.139330000	5.163271000	-1.725626000
H	-0.988699000	3.726506000	-0.740058000
H	-1.599014000	3.387123000	-2.359353000
H	-5.221560000	3.687241000	-0.709010000
H	-4.589163000	3.281807000	-2.308860000
H	-3.097336000	4.671099000	-0.027038000
C	-0.224986000	0.705436000	-0.645287000
C	0.183756000	-0.510910000	-0.180899000
S	-1.129401000	-1.599230000	0.298727000
C	-2.401263000	-0.446701000	-0.478559000
C	-1.645882000	0.881639000	-0.670010000
H	0.470536000	1.485135000	-0.941996000
P	1.936192000	-0.984865000	-0.044976000
C	1.984486000	-2.180542000	1.337580000
C	1.711240000	-3.544350000	1.148590000
C	2.244193000	-1.694263000	2.628910000
C	1.699688000	-4.409073000	2.243991000
C	2.228987000	-2.563724000	3.719492000
C	1.958394000	-3.921145000	3.527322000
H	1.510692000	-3.931635000	0.154949000
H	2.461901000	-0.639532000	2.776811000
H	1.488119000	-5.463525000	2.093094000
H	2.433651000	-2.182414000	4.715520000
H	1.951247000	-4.598455000	4.376452000
C	2.306060000	-1.938420000	-1.562100000
C	1.494800000	-1.833304000	-2.700873000
C	3.467956000	-2.727583000	-1.604173000
C	1.838287000	-2.519520000	-3.867328000
C	3.803238000	-3.410151000	-2.772672000
C	2.990199000	-3.308133000	-3.905131000
H	0.598737000	-1.220833000	-2.680950000
H	4.108331000	-2.806336000	-0.731547000
H	1.201560000	-2.439479000	-4.743565000
H	4.703411000	-4.017136000	-2.797623000
H	3.254786000	-3.840922000	-4.813942000
C	-2.725561000	-1.012342000	-1.881017000
H	-3.427595000	-0.351740000	-2.397993000
H	-1.807659000	-1.071512000	-2.471775000
H	-3.165350000	-2.008950000	-1.819998000
C	-3.305010000	0.218872000	1.851720000
H	-2.640132000	1.087697000	1.857740000
H	-4.224873000	0.485574000	2.377854000
H	-2.819715000	-0.595443000	2.392816000
C	-7.566316000	-1.027965000	0.409083000
O	-7.827580000	-2.185522000	0.708143000
C	-8.736788000	-0.034967000	0.179736000
F	-9.917050000	-0.630744000	0.375949000
F	-8.651624000	1.019683000	1.022170000
F	-8.717870000	0.456341000	-1.080974000
I	4.035252000	3.433068000	0.748502000
C	4.742497000	-0.003806000	-0.053698000
C	5.334650000	-0.796937000	0.933367000
C	5.326310000	0.117256000	-1.319007000
C	6.525082000	-1.477004000	0.644221000
C	6.515733000	-0.566707000	-1.593430000
C	7.114599000	-1.367080000	-0.617020000
H	4.875922000	-0.908604000	1.910313000
H	4.865570000	0.727844000	-2.089201000
H	6.982504000	-2.097121000	1.410906000
H	6.968586000	-0.471976000	-2.576940000
H	8.034369000	-1.901133000	-0.838003000

DTE^{o}-COCF$_3$

C	2.759474000	1.146499000	-0.626776000
C	2.330436000	-0.124591000	-1.002367000
S	3.456555000	-1.353428000	-0.519939000
C	4.540454000	-0.165924000	0.183190000
C	4.021628000	1.103704000	0.026042000
H	4.541023000	1.980872000	0.393614000
C	1.085029000	-0.516156000	-1.731234000
H	0.302608000	-0.814495000	-1.024929000
H	0.707569000	0.332691000	-2.305344000
H	1.261471000	-1.353653000	-2.413029000
C	1.735614000	4.568237000	-1.895034000
C	0.467114000	4.150027000	-1.123079000
C	0.749118000	2.712145000	-0.716607000
C	2.059692000	2.413509000	-0.884483000
C	2.839086000	3.629906000	-1.362331000
H	1.979148000	5.627741000	-1.776803000
H	-0.448903000	4.249424000	-1.716173000
H	0.316682000	4.757876000	-0.218698000
H	3.590682000	3.376159000	-2.117871000
H	3.381119000	4.082000000	-0.518352000
H	1.587967000	4.378331000	-2.964058000
C	-1.615689000	1.775484000	-0.764429000
C	-2.492160000	0.939035000	-0.121255000
S	-1.726874000	0.270930000	1.311848000
C	-0.235588000	1.126519000	1.009245000
C	-0.319648000	1.875814000	-0.150591000
H	-1.875433000	2.300345000	-1.677975000
C	0.902001000	1.008022000	1.977158000
H	1.491569000	0.101556000	1.796258000
H	1.573392000	1.863586000	1.872605000
H	0.545611000	0.971762000	3.011587000
P	-4.166195000	0.535141000	-0.726120000
C	-3.932337000	-1.165456000	-1.423994000
C	-5.045102000	-2.012456000	-1.568581000
C	-2.692776000	-1.590437000	-1.924753000
C	-4.912400000	-3.261460000	-2.176732000
C	-2.562801000	-2.838831000	-2.537727000
C	-3.670077000	-3.680461000	-2.662089000
H	-6.016487000	-1.702822000	-1.191748000
H	-1.825520000	-0.944350000	-1.836283000
H	-5.780515000	-3.908377000	-2.270096000
H	-1.592544000	-3.153535000	-2.912944000
H	-3.567892000	-4.653239000	-3.134769000
C	-5.069994000	0.197747000	0.852621000
C	-4.929200000	-0.987593000	1.594598000
C	-5.912756000	1.208078000	1.341864000
C	-5.609917000	-1.151012000	2.801916000
C	-6.590816000	1.044949000	2.552220000
C	-6.440235000	-0.134873000	3.283917000
H	-4.293419000	-1.786739000	1.226294000
H	-6.039755000	2.126399000	0.773991000
H	-5.491402000	-2.071828000	3.366396000
H	-7.239362000	1.835762000	2.918805000
H	-6.970361000	-0.265065000	4.223272000
C	5.804271000	-0.445722000	0.845731000

O	6.541197000	0.414126000	1.303171000
C	6.229047000	-1.931511000	0.984987000
F	6.318474000	-2.522273000	-0.228992000
F	7.412034000	-2.044746000	1.593510000
F	5.314202000	-2.622947000	1.702546000

DTE^c-COCF$_3$

C	-2.636506000	0.868282000	-0.317549000
C	-1.485089000	0.012566000	0.248517000
S	-2.265034000	-1.668849000	0.562527000
C	-3.896377000	-0.953212000	0.455368000
C	-3.916580000	0.333747000	-0.022354000
H	-4.831023000	0.886230000	-0.202583000
C	-2.289638000	4.368544000	-1.447604000
C	-0.899204000	3.789581000	-1.807754000
C	-0.962742000	2.370924000	-1.284535000
C	-2.333316000	2.028555000	-0.974453000
C	-3.237904000	3.144035000	-1.435361000
H	-2.617309000	5.148035000	-2.140092000
H	-0.071968000	4.365690000	-1.381373000
H	-0.753081000	3.775769000	-2.896435000
H	-4.118806000	3.277977000	-0.801450000
H	-3.594309000	2.927326000	-2.452373000
H	-2.253933000	4.805158000	-0.443270000
C	1.448426000	1.635979000	-1.268778000
C	2.210845000	0.606650000	-0.799063000
S	1.269132000	-0.750240000	-0.150381000
C	-0.333722000	0.001032000	-0.780546000
C	0.034818000	1.456037000	-1.122619000
H	1.882220000	2.544575000	-1.673765000
P	4.046675000	0.679307000	-0.777288000
C	4.378638000	0.984835000	1.018936000
C	5.688928000	0.787286000	1.489874000
C	3.412689000	1.492540000	1.900272000
C	6.017280000	1.071405000	2.815349000
C	3.746368000	1.782238000	3.225906000
C	5.046116000	1.569979000	3.688739000
H	6.453145000	0.397170000	0.822155000
H	2.396287000	1.661043000	1.558849000
H	7.032242000	0.904467000	3.165412000
H	2.984688000	2.169200000	3.897396000
H	5.302224000	1.792394000	4.720721000
C	4.487650000	-1.106304000	-0.970002000
C	4.485622000	-2.033865000	0.085037000
C	4.807585000	-1.547227000	-2.263833000
C	4.791082000	-3.374334000	-0.154319000
C	5.106598000	-2.890384000	-2.502607000
C	5.099508000	-3.805555000	-1.447767000
H	4.248678000	-1.710139000	1.093684000
H	4.822878000	-0.838422000	-3.088199000
H	4.784978000	-4.082844000	0.669397000
H	5.350494000	-3.218749000	-3.509093000
H	5.336623000	-4.849827000	-1.630975000
C	-0.682341000	-0.719738000	-2.104552000
H	-1.587232000	-0.287866000	-2.541981000
H	0.139958000	-0.592572000	-2.813042000
H	-0.847174000	-1.786926000	-1.947999000
C	-1.081048000	0.604006000	1.619485000
H	-0.678009000	1.613370000	1.494551000
H	-1.963091000	0.663831000	2.261753000
H	-0.330122000	-0.012413000	2.117173000
C	-5.028743000	-1.775774000	0.819537000
O	-4.950382000	-2.932555000	1.215979000
C	-6.434369000	-1.134151000	0.678429000
F	-6.679431000	-0.774979000	-0.603498000
F	-7.392360000	-1.985118000	1.060389000
F	-6.541368000	-0.015711000	1.432909000

TS3 [Pd(**DTE^o-COCF$_3$**)(**Ph**)**I**]

Pd	3.281795000	1.060855000	-0.021684000
S	-0.505011000	-1.449923000	1.693553000
S	-5.272374000	-1.743220000	-1.480775000
P	2.261728000	-0.921478000	0.314258000
F	-9.902638000	-0.759123000	0.558122000
F	-8.791852000	0.951064000	-0.206309000
F	-8.177780000	-0.003821000	1.653322000
O	-8.200231000	-2.273121000	-0.753785000
C	2.819592000	-1.865057000	1.776615000
C	3.378780000	-1.155143000	2.851703000
H	3.526650000	-0.081261000	2.772490000
C	3.747573000	-1.827583000	4.016120000
H	4.183602000	-1.274010000	4.842405000
C	3.561601000	-3.209493000	4.114650000
H	3.852830000	-3.732957000	5.020707000
C	3.003262000	-3.918344000	3.048642000
H	2.855598000	-4.991620000	3.124000000
C	2.631093000	-3.251711000	1.879697000
H	2.198345000	-3.810438000	1.056277000
C	2.204273000	-2.094939000	-1.087509000
C	3.316519000	-2.907925000	-1.363689000
H	4.191334000	-2.875114000	-0.722249000
C	3.298033000	-3.759786000	-2.467161000
H	4.159554000	-4.388144000	-2.672920000
C	2.181563000	-3.798910000	-3.307939000
H	2.172408000	-4.461979000	-4.168221000
C	1.079684000	-2.983270000	-3.041963000
H	0.209599000	-3.008931000	-3.691699000
C	1.088197000	-2.131908000	-1.935565000
H	0.229882000	-1.499666000	-1.734694000
C	0.547366000	-0.481602000	0.681830000
C	-0.125385000	0.616361000	0.203475000
H	0.337188000	1.355659000	-0.441243000
C	-1.494196000	0.690916000	0.618933000
C	-1.846874000	-0.361005000	1.446988000
C	-3.157558000	-0.631319000	2.118887000
H	-3.732376000	0.294618000	2.194818000
H	-3.018048000	-1.037885000	3.125373000
H	-3.758099000	-1.348696000	1.547574000
C	-2.366909000	1.799580000	0.205360000
H	-1.908708000	3.492073000	1.447488000
C	-2.969814000	4.048456000	-0.404812000
H	-2.616401000	4.209443000	-1.429261000
C	-4.215493000	3.135936000	-0.436360000
C	-3.628862000	1.741304000	-0.279051000
C	-4.434161000	0.553551000	-0.597028000
C	-5.800814000	0.447948000	-0.213508000
H	-6.316203000	1.221988000	0.341151000
C	-6.399539000	-0.735356000	-0.598989000
C	-4.009172000	-0.561709000	-1.314936000
C	-2.669250000	-0.824659000	-1.925346000

H	-2.143617000	0.118195000	-2.091613000
H	-2.758318000	-1.352278000	-2.879867000
H	-2.052905000	-1.438103000	-1.258553000
C	-7.751081000	-1.201651000	-0.375220000
C	-8.678535000	-0.241485000	0.419908000
C	-1.912849000	3.239677000	0.376884000
H	-0.890704000	3.406169000	0.018064000
H	-3.168390000	5.029342000	0.035463000
H	-4.896395000	3.350450000	0.400829000
H	-4.803804000	3.241722000	-1.354359000
I	2.912868000	3.760567000	-0.019549000
C	4.818182000	-0.007190000	-0.650642000
C	5.749980000	-0.557055000	0.236007000
C	5.013259000	-0.095120000	-2.034964000
C	6.901574000	-1.172586000	-0.271981000
C	6.168246000	-0.710685000	-2.528541000
C	7.111170000	-1.252714000	-1.650419000
H	5.585999000	-0.526461000	1.308329000
H	4.275697000	0.300466000	-2.727417000
H	7.626474000	-1.596942000	0.417971000
H	6.321364000	-0.773519000	-3.602786000
H	8.000663000	-1.740270000	-2.039194000

TS3 [Pd(**DTE^c-COCF$_3$**)(**Ph**)**I**]

Pd	3.267759000	1.042501000	0.144946000
C	-4.340711000	0.820715000	-0.202577000
C	-3.487951000	-0.312818000	0.402327000
S	-4.632253000	-1.804751000	0.408509000
C	-6.045536000	-0.743299000	0.179583000
C	-5.734662000	0.554398000	-0.135522000
H	-6.479354000	1.308989000	-0.358339000
C	-3.126666000	4.245584000	-0.870588000
C	-1.847358000	3.409310000	-1.120060000
C	-2.274573000	2.001375000	-0.771150000
C	-3.718448000	1.940391000	-0.675918000
C	-4.298103000	3.261751000	-1.115084000
H	-3.182536000	5.132699000	-1.506161000
H	-0.986708000	3.750994000	-0.536881000
H	-1.555636000	3.450738000	-2.178387000
H	-5.214936000	3.530259000	-0.583175000
H	-4.540307000	3.215669000	-2.186270000
H	-3.147065000	4.577863000	0.173063000
C	-0.094346000	0.770520000	-0.556029000
C	0.364122000	-0.440489000	-0.130868000
S	-0.907815000	-1.603346000	0.298094000
C	-2.219084000	-0.484683000	-0.462444000
C	-1.521547000	0.881673000	-0.591655000
H	0.564448000	1.597394000	-0.801089000
P	2.124687000	-0.891741000	-0.003028000
C	2.209562000	-1.936975000	1.495159000
C	1.949955000	-3.315575000	1.454212000
C	2.464832000	-1.311343000	2.726381000
C	1.951929000	-4.057566000	2.636656000
C	2.463672000	-2.059129000	3.903381000
C	2.209123000	-3.432577000	3.859040000
H	1.747865000	-3.810594000	0.510236000
H	2.667282000	-0.243922000	2.759498000
H	1.751306000	-5.124195000	2.600186000
H	2.665406000	-1.570049000	4.851763000
H	2.212052000	-4.014925000	4.775841000
C	2.437440000	-1.981563000	-1.437418000
C	1.638194000	-1.887830000	-2.585780000
C	3.535829000	-2.857210000	-1.421167000
C	1.928433000	-2.673041000	-3.702889000
C	3.816743000	-3.640466000	-2.539592000
C	3.014691000	-3.550379000	-3.681013000
H	0.793430000	-1.206718000	-2.610502000
H	4.169410000	-2.923642000	-0.542834000
H	1.301768000	-2.600417000	-4.586881000
H	4.666254000	-4.316682000	-2.519743000
H	3.237996000	-4.160885000	-4.551147000
C	-2.497141000	-1.013336000	-1.888909000
H	-3.221344000	-0.367232000	-2.393485000
H	-1.570067000	-1.009484000	-2.467647000
H	-2.891528000	-2.030389000	-1.869989000
C	-3.189434000	0.053581000	1.874979000
H	-2.562678000	0.948789000	1.924318000
H	-4.128920000	0.261485000	2.392868000
H	-2.678675000	-0.758382000	2.395700000
C	-7.365801000	-1.330998000	0.293026000
O	-7.579611000	-2.509798000	0.542859000
C	-8.575663000	-0.385304000	0.067680000
F	-9.731285000	-1.040987000	0.212854000
F	-8.561215000	0.642578000	0.946586000
F	-8.548456000	0.149376000	-1.175185000
I	2.938171000	3.704958000	0.577402000
C	4.863547000	-0.082943000	-0.147220000
C	5.499146000	-0.738319000	0.912035000
C	5.425362000	-0.081844000	-1.429657000
C	6.729979000	-1.368612000	0.687241000
C	6.656198000	-0.712653000	-1.638822000
C	7.307777000	-1.358523000	-0.584226000
H	5.047297000	-0.775019000	1.897932000
H	4.913913000	0.393091000	-2.262032000
H	7.226997000	-1.874664000	1.510812000
H	7.096184000	-0.706570000	-2.632597000
H	8.257393000	-1.857603000	-0.754927000

4 [Pd(**DTE^o-COCF$_3$**)(**Ph**)**I**]

Pd	3.529122000	1.099009000	-0.258587000
S	-0.418783000	-0.976142000	1.809205000
S	-5.138957000	-2.044666000	-1.149201000
P	2.305769000	-0.694416000	0.309279000
F	-9.761471000	-0.910938000	0.827538000
F	-8.784392000	0.658112000	-0.324704000
F	-8.057756000	0.155435000	1.666760000
O	-8.011550000	-2.566686000	-0.223071000
C	2.837246000	-1.472403000	1.877916000
C	3.476174000	-0.673531000	2.840054000
H	3.700034000	0.366306000	2.617999000
C	3.824911000	-1.213808000	4.077369000
H	4.321758000	-0.590968000	4.815282000
C	3.539514000	-2.551854000	4.362749000
H	3.815623000	-2.972565000	5.325296000
C	2.900070000	-3.348521000	3.410566000
H	2.674639000	-4.387944000	3.629963000
C	2.546853000	-2.814269000	2.170024000
H	2.050849000	-3.442535000	1.437738000
C	2.173137000	-2.040661000	-0.930507000
C	3.190998000	-3.002427000	-1.044374000

H	4.043521000	-2.980007000	-0.374071000
C	3.111158000	-3.989351000	-2.025632000
H	3.900413000	-4.731336000	-2.103135000
C	2.027991000	-4.018867000	-2.909096000
H	1.971260000	-4.787518000	-3.674465000
C	1.021269000	-3.056961000	-2.807003000
H	0.176969000	-3.072767000	-3.490149000
C	1.091258000	-2.070095000	-1.821695000
H	0.304325000	-1.328174000	-1.747956000
C	0.603866000	-0.176371000	0.630582000
C	-0.100653000	0.794724000	-0.033874000
H	0.341784000	1.420045000	-0.799602000
C	-1.471852000	0.901761000	0.369055000
C	-1.790567000	0.011631000	1.380701000
C	-3.086290000	-0.161087000	2.111837000
H	-3.685375000	0.748284000	2.024742000
H	-2.923592000	-0.368978000	3.173958000
H	-3.674061000	-0.986839000	1.694576000
C	-2.387132000	1.877977000	-0.240230000
H	-1.996142000	3.796909000	0.642277000
C	-3.084158000	3.937577000	-1.271442000
H	-2.742379000	3.905738000	-2.311983000
C	-4.292526000	2.989835000	-1.106719000
C	-3.648582000	1.677196000	-0.687431000
C	-4.404272000	0.417593000	-0.752278000
C	-5.754211000	0.323348000	-0.311059000
H	-6.296376000	1.163055000	0.105354000
C	-6.297796000	-0.939704000	-0.441798000
C	-3.937479000	-0.793991000	-1.255644000
C	-2.597499000	-1.104591000	-1.842504000
H	-2.123832000	-0.185174000	-2.193528000
H	-2.676229000	-1.804360000	-2.680139000
H	-1.938430000	-1.553302000	-1.091035000
C	-7.614802000	-1.419440000	-0.082598000
C	-8.577395000	-0.366298000	0.532644000
C	-1.992322000	3.340482000	-0.358431000
H	-0.978110000	3.474351000	-0.751065000
H	-3.319816000	4.977948000	-1.031576000
H	-4.976444000	3.338376000	-0.318501000
H	-4.890105000	2.890778000	-2.019512000
I	2.543423000	3.616820000	0.125335000
C	4.905648000	-0.226994000	-0.786194000
C	5.830730000	-0.736669000	0.133097000
C	5.057317000	-0.483603000	-2.156224000
C	6.931063000	-1.471031000	-0.327360000
C	6.162145000	-1.213511000	-2.606084000
C	7.097324000	-1.710645000	-1.693408000
H	5.697643000	-0.580657000	1.199294000
H	4.318754000	-0.131585000	-2.871816000
H	7.649932000	-1.862382000	0.387999000
H	6.281297000	-1.404348000	-3.669527000
H	7.946507000	-2.289735000	-2.045227000

4 [Pd(**DTE^{c}-COCF$_3$**)(**Ph**)**I**]

Pd	-3.416029000	-1.207734000	0.182672000
C	4.280280000	-0.684892000	-0.373426000
C	3.373858000	0.319718000	0.365855000
S	4.451954000	1.844581000	0.586385000
C	5.912653000	0.882700000	0.241323000
C	5.660368000	-0.373186000	-0.248616000
H	6.439490000	-1.059769000	-0.557255000
C	3.220198000	-4.038968000	-1.498854000
C	1.908756000	-3.231216000	-1.658988000
C	2.272440000	-1.864028000	-1.125025000
C	3.710810000	-1.756242000	-1.000917000
C	4.350642000	-2.984428000	-1.598657000
H	3.320790000	-4.835226000	-2.240529000
H	1.058635000	-3.678688000	-1.134575000
H	1.628890000	-3.149278000	-2.718269000
H	5.273497000	-3.281105000	-1.092732000
H	4.599792000	-2.790678000	-2.651604000
H	3.244481000	-4.499687000	-0.505245000
C	0.039042000	-0.770638000	-0.768449000
C	-0.475338000	0.340901000	-0.175571000
S	0.740721000	1.496584000	0.409983000
C	2.103681000	0.552292000	-0.482856000
C	1.469492000	-0.811540000	-0.807178000
H	-0.579340000	-1.584151000	-1.127078000
P	-2.253676000	0.704443000	-0.013737000
C	-2.366600000	1.740300000	1.490715000
C	-2.105480000	3.119465000	1.458949000
C	-2.649695000	1.113004000	2.714826000
C	-2.133718000	3.859215000	2.642175000
C	-2.674486000	1.858866000	3.893004000
C	-2.418597000	3.232090000	3.857324000
H	-1.881533000	3.616808000	0.521366000
H	-2.851400000	0.045438000	2.741115000
H	-1.931968000	4.925809000	2.611787000
H	-2.896428000	1.367551000	4.835664000
H	-2.441535000	3.812719000	4.774935000
C	-2.608765000	1.795451000	-1.444785000
C	-1.859775000	1.675118000	-2.624262000
C	-3.684289000	2.697786000	-1.394542000
C	-2.176885000	2.456913000	-3.736795000
C	-3.991490000	3.478633000	-2.507448000
C	-3.239982000	3.360005000	-3.680223000
H	-1.032078000	0.975469000	-2.678315000
H	-4.284657000	2.784716000	-0.495479000
H	-1.588812000	2.360163000	-4.644699000
H	-4.823581000	4.174772000	-2.458880000
H	-3.484617000	3.967737000	-4.546555000
C	2.363222000	1.283312000	-1.820483000
H	3.114626000	0.741959000	-2.402920000
H	1.437357000	1.321675000	-2.400184000
H	2.715825000	2.303168000	-1.659455000
C	3.081029000	-0.253405000	1.772291000
H	2.489808000	-1.170473000	1.694107000
H	4.024842000	-0.492067000	2.268602000
H	2.534777000	0.460619000	2.391137000
C	7.204979000	1.503407000	0.450882000
O	7.367425000	2.646645000	0.857167000
C	8.455890000	0.646158000	0.121647000
F	9.581369000	1.322393000	0.372395000
F	8.474899000	-0.490132000	0.855103000
F	8.466361000	0.281675000	-1.181786000
I	-2.052822000	-3.465373000	0.919712000
C	-4.988640000	-0.070819000	-0.225095000
C	-5.682719000	0.594276000	0.793347000
C	-5.511355000	-0.115435000	-1.524946000
C	-6.924996000	1.178476000	0.515250000
C	-6.755017000	0.466537000	-1.790547000

C	-7.460613000	1.116336000	-0.773401000
H	-5.262760000	0.673610000	1.791712000
H	-4.955059000	-0.586387000	-2.331227000
H	-7.464490000	1.690762000	1.307757000
H	-7.162650000	0.423367000	-2.797217000
H	-8.419092000	1.580847000	-0.987344000

Sn(CH$_2$CH)Bu$_3$

C	-3.241339000	-1.112243000	-0.072672000
Sn	-1.180453000	-0.460230000	-0.027951000
H	-3.452345000	-2.182047000	-0.147502000
C	-1.152939000	1.724910000	0.047426000
H	-1.971031000	2.097681000	-0.582153000
H	-1.387284000	2.034632000	1.073811000
C	-0.136280000	-1.150830000	-1.822821000
H	-0.448870000	-0.513501000	-2.659868000
H	-0.491997000	-2.162570000	-2.056340000
C	-0.170560000	-1.267522000	1.738469000
H	-0.890146000	-1.275590000	2.566858000
H	0.082457000	-2.316597000	1.538200000
C	1.090094000	-0.486861000	2.144639000
H	1.806213000	-0.464080000	1.311192000
H	0.832173000	0.562531000	2.346542000
C	1.793240000	-1.067837000	3.380174000
H	1.084959000	-1.087836000	4.220145000
H	2.061216000	-2.114788000	3.180831000
C	3.045695000	-0.282424000	3.779711000
H	3.530251000	-0.715596000	4.662080000
H	3.781894000	-0.273015000	2.966658000
H	2.799973000	0.760710000	4.013402000
C	1.395902000	-1.147977000	-1.697595000
H	1.704900000	-1.787350000	-0.858419000
H	1.753224000	-0.138084000	-1.451753000
C	2.111007000	-1.625519000	-2.970011000
H	1.764390000	-2.638431000	-3.218069000
H	1.811560000	-0.985080000	-3.811313000
C	3.636531000	-1.620441000	-2.836099000
H	3.963656000	-2.276471000	-2.020156000
H	4.124271000	-1.964389000	-3.755237000
H	4.010347000	-0.612522000	-2.618123000
C	0.178548000	2.348508000	-0.402814000
H	0.409274000	2.037552000	-1.431671000
H	1.002849000	1.968317000	0.216849000
C	0.183121000	3.882787000	-0.338886000
H	-0.634820000	4.270422000	-0.962114000
H	-0.039479000	4.199306000	0.689675000
C	1.511437000	4.496361000	-0.790703000
H	1.742316000	4.219401000	-1.826610000
H	1.490797000	5.590705000	-0.735788000
H	2.341121000	4.147308000	-0.163858000
C	-4.288666000	-0.280200000	-0.011953000
H	-4.172844000	0.801141000	0.064004000
H	-5.321119000	-0.634534000	-0.034905000

5 [Pd(**DTE^o-COCF$_3$**)(**Ph**)**I**]·**Sn(CH$_2$CH)Bu$_3$**

Pd	-1.650388000	0.314665000	-0.744168000
S	2.525395000	0.371927000	2.100538000
S	7.547359000	1.706051000	-0.158139000
P	-0.108924000	1.349319000	0.680812000
F	11.756736000	-1.107500000	0.803115000
F	10.563517000	-1.685575000	-0.924935000
F	9.844577000	-2.110638000	1.087195000
O	10.408686000	1.147620000	0.782449000
C	-0.651867000	1.474454000	2.426113000
C	-1.689219000	0.646023000	2.878781000
H	-2.173217000	-0.038326000	2.192464000
C	-2.093486000	0.693424000	4.213866000
H	-2.901228000	0.051116000	4.550932000
C	-1.464365000	1.564871000	5.105532000
H	-1.780582000	1.602163000	6.144076000
C	-0.429174000	2.391949000	4.660745000
H	0.061548000	3.072107000	5.350783000
C	-0.023445000	2.352061000	3.326461000
H	0.771793000	3.008802000	2.988703000
C	0.408081000	3.052250000	0.212360000
C	-0.350779000	4.162945000	0.612392000
H	-1.201571000	4.034432000	1.271997000
C	-0.025182000	5.437973000	0.150973000
H	-0.623762000	6.288843000	0.462849000
C	1.059384000	5.619584000	-0.711506000
H	1.309415000	6.613918000	-1.070212000
C	1.818641000	4.518103000	-1.110963000
H	2.665056000	4.650033000	-1.779063000
C	1.493974000	3.239347000	-0.654480000
H	2.086058000	2.390073000	-0.976805000
C	1.448509000	0.427937000	0.721957000
C	1.992751000	-0.251101000	-0.336583000
H	1.482395000	-0.357396000	-1.286738000
C	3.289387000	-0.804574000	-0.075512000
C	3.706902000	-0.565602000	1.222046000
C	4.960371000	-1.014477000	1.908476000
H	5.386013000	-1.871287000	1.381118000
H	4.767608000	-1.307497000	2.945262000
H	5.717707000	-0.221782000	1.919683000
C	4.037397000	-1.547986000	-1.099634000
H	3.235509000	-3.537805000	-1.198507000
C	4.390789000	-2.988387000	-2.995722000
H	4.126095000	-2.411688000	-3.889118000
C	5.745286000	-2.484114000	-2.451139000
C	5.338983000	-1.416725000	-1.446648000
C	6.325751000	-0.450899000	-0.941924000
C	7.641534000	-0.845105000	-0.569312000
H	7.984121000	-1.870725000	-0.623502000
C	8.428035000	0.193901000	-0.111317000
C	6.133232000	0.920327000	-0.791870000
C	4.917263000	1.731738000	-1.107788000
H	4.300909000	1.205702000	-1.840193000
H	5.182588000	2.714644000	-1.508987000
H	4.307523000	1.888574000	-0.211205000
C	9.792725000	0.176560000	0.368748000
C	10.508147000	-1.202602000	0.336461000
C	3.379631000	-2.679717000	-1.871080000
H	2.384282000	-2.414392000	-2.244989000
H	4.408528000	-4.045009000	-3.276389000
H	6.299601000	-3.285921000	-1.940921000
H	6.406131000	-2.094466000	-3.233253000
I	-1.092614000	-2.332528000	0.182272000
C	-2.224169000	2.177527000	-1.248089000
C	-3.241747000	2.791190000	-0.504477000
C	-1.678736000	2.848086000	-2.348732000

C	-3.709206000	4.061519000	-0.861110000
C	-2.143447000	4.121652000	-2.698046000
C	-3.158987000	4.731663000	-1.956618000
H	-3.678808000	2.287066000	0.353261000
H	-0.879550000	2.394782000	-2.929137000
H	-4.503070000	4.523260000	-0.279193000
H	-1.704447000	4.637385000	-3.548561000
H	-3.516312000	5.720824000	-2.229081000
C	-3.527459000	-0.225338000	-2.149431000
Sn	-5.016486000	-1.250437000	-0.914267000
H	-3.856849000	0.758995000	-2.480930000
C	-4.936102000	-3.396250000	-1.289195000
H	-5.779861000	-3.673600000	-1.932833000
H	-4.022141000	-3.578793000	-1.867079000
C	-6.830247000	-0.195686000	-1.532849000
H	-7.696210000	-0.639923000	-1.026674000
H	-6.982068000	-0.348024000	-2.608738000
C	-4.720389000	-0.783595000	1.195933000
H	-3.938050000	-1.450504000	1.572243000
H	-4.320413000	0.235324000	1.254798000
C	-5.987407000	-0.902244000	2.054822000
H	-6.773726000	-0.239112000	1.666734000
H	-6.393808000	-1.921927000	1.991428000
C	-5.738585000	-0.562496000	3.531969000
H	-4.965446000	-1.236697000	3.926081000
H	-5.323266000	0.452672000	3.600231000
C	-6.999487000	-0.663993000	4.394526000
H	-6.792474000	-0.418594000	5.442359000
H	-7.777580000	0.022518000	4.039035000
H	-7.416204000	-1.678110000	4.366377000
C	-6.733279000	1.305503000	-1.213323000
H	-5.841993000	1.737491000	-1.688734000
H	-6.590350000	1.449427000	-0.132439000
C	-7.957399000	2.118525000	-1.656989000
H	-8.112060000	1.975424000	-2.735414000
H	-8.855040000	1.722193000	-1.162399000
C	-7.811997000	3.612550000	-1.351387000
H	-6.936939000	4.034249000	-1.860727000
H	-8.692307000	4.180214000	-1.673587000
H	-7.680655000	3.784158000	-0.275794000
C	-4.928170000	-4.247289000	-0.010600000
H	-5.851596000	-4.079141000	0.562397000
H	-4.102293000	-3.930603000	0.640419000
C	-4.783797000	-5.750012000	-0.289884000
H	-5.604989000	-6.078334000	-0.942489000
H	-3.856082000	-5.918655000	-0.854049000
C	-4.769066000	-6.594419000	0.987505000
H	-5.697361000	-6.465828000	1.557676000
H	-4.660596000	-7.662028000	0.764907000
H	-3.937441000	-6.304176000	1.640976000
C	-2.401679000	-0.696719000	-2.766370000
H	-2.048251000	-1.715802000	-2.635111000
H	-1.894199000	-0.117991000	-3.540327000

5 [Pd(**DTE^c-COCF$_3$**)(**Ph**)**I**]·**Sn(CH$_2$CH)Bu$_3$**

Pd	-1.603912000	0.352943000	-0.527596000
C	6.013011000	-0.933803000	-1.035919000
C	5.305398000	-0.422134000	0.235071000
S	6.698897000	0.253343000	1.302298000
C	7.901080000	-0.625760000	0.320958000
C	7.400386000	-1.159763000	-0.839711000
H	8.012530000	-1.676433000	-1.569219000
C	4.287850000	-2.371810000	-3.969463000
C	3.250429000	-1.330702000	-3.480966000
C	3.879973000	-0.773201000	-2.223694000
C	5.271120000	-1.162512000	-2.160852000
C	5.650100000	-1.849358000	-3.449066000
H	4.275214000	-2.507082000	-5.053886000
H	2.260462000	-1.762595000	-3.304138000
H	3.126621000	-0.527286000	-4.220270000
H	6.399071000	-2.635042000	-3.317095000
H	6.067944000	-1.110634000	-4.147704000
H	4.074481000	-3.341633000	-3.506922000
C	1.970326000	0.403272000	-1.088717000
C	1.689750000	0.990746000	0.107467000
S	3.076139000	1.136916000	1.198791000
C	4.251475000	0.628166000	-0.181088000
C	3.335393000	0.010199000	-1.251676000
H	1.205422000	0.188411000	-1.826273000
P	0.034452000	1.637157000	0.525807000
C	-0.064656000	1.658044000	2.354027000
C	0.717813000	2.549314000	3.109391000
C	-0.925565000	0.766205000	3.010851000
C	0.648044000	2.533621000	4.501953000
C	-0.995388000	0.760433000	4.405068000
C	-0.207915000	1.639741000	5.150891000
H	1.370540000	3.261069000	2.614884000
H	-1.531815000	0.073600000	2.436868000
H	1.258996000	3.222350000	5.077888000
H	-1.664084000	0.067501000	4.906274000
H	-0.263050000	1.631675000	6.235686000
C	0.173838000	3.407334000	0.039968000
C	0.901961000	3.764801000	-1.104518000
C	-0.533262000	4.394457000	0.742796000
C	0.926314000	5.092165000	-1.535047000
C	-0.507728000	5.718733000	0.306031000
C	0.221448000	6.071563000	-0.832133000
H	1.449821000	3.011669000	-1.661675000
H	-1.114829000	4.132655000	1.619181000
H	1.498591000	5.358492000	-2.419102000
H	-1.063790000	6.473158000	0.854767000
H	0.238953000	7.103751000	-1.169754000
C	4.850819000	1.928341000	-0.766430000
H	5.501485000	1.691551000	-1.613407000
H	4.043222000	2.575172000	-1.119345000
H	5.433334000	2.472600000	-0.021594000
C	4.701741000	-1.642436000	0.969157000
H	3.909861000	-2.096848000	0.366789000
H	5.483742000	-2.387758000	1.133576000
H	4.281620000	-1.361478000	1.936523000
C	9.271179000	-0.659412000	0.786608000
O	9.667234000	-0.135560000	1.820457000
C	10.297796000	-1.399885000	-0.111062000
F	11.515372000	-1.389075000	0.441157000
F	9.938256000	-2.688896000	-0.309568000
F	10.384483000	-0.817106000	-1.329708000
I	-0.385826000	-2.114560000	0.321049000
C	-2.614812000	2.104310000	-0.599894000
C	-3.369013000	2.452849000	0.528693000
C	-2.562434000	2.990438000	-1.685028000
C	-4.068607000	3.665781000	0.565717000

C	-3.261943000	4.200019000	-1.644642000
C	-4.015025000	4.543199000	-0.517738000
H	-3.406136000	1.799954000	1.395233000
H	-1.962612000	2.753362000	-2.558810000
H	-4.649599000	3.920563000	1.448532000
H	-3.208179000	4.878313000	-2.492368000
H	-4.552494000	5.486314000	-0.485724000
C	-3.131083000	-1.130597000	-1.686429000
Sn	-5.004537000	-1.308178000	-0.575583000
H	-2.536048000	-2.042667000	-1.697343000
C	-6.516834000	-0.003294000	-1.453629000
H	-6.527054000	-0.234441000	-2.527027000
H	-7.483169000	-0.345596000	-1.060538000
C	-4.567053000	-1.018713000	1.545114000
H	-5.417117000	-0.527120000	2.033904000
H	-3.722219000	-0.324863000	1.603879000
C	-5.590593000	-3.387555000	-0.885044000
H	-5.582599000	-3.588305000	-1.963940000
H	-4.803944000	-4.014825000	-0.446943000
C	-6.957017000	-3.757483000	-0.287741000
H	-6.974388000	-3.530129000	0.788079000
H	-7.745936000	-3.138158000	-0.738753000
C	-7.321240000	-5.237017000	-0.480944000
H	-7.312170000	-5.471112000	-1.554573000
H	-6.538853000	-5.859437000	-0.025139000
C	-8.683398000	-5.602765000	0.115481000
H	-8.918230000	-6.662591000	-0.034804000
H	-8.707638000	-5.406494000	1.194373000
H	-9.487386000	-5.015339000	-0.344652000
C	-4.206871000	-2.329347000	2.260748000
H	-3.389146000	-2.832304000	1.726746000
H	-5.060063000	-3.021504000	2.237177000
C	-3.772625000	-2.116389000	3.717076000
H	-2.907711000	-1.440873000	3.727857000
H	-4.575184000	-1.602490000	4.264879000
C	-3.407850000	-3.421134000	4.429818000
H	-2.584098000	-3.930680000	3.915597000
H	-3.094235000	-3.241750000	5.464533000
H	-4.260172000	-4.111158000	4.454463000
C	-6.373351000	1.509978000	-1.241443000
H	-5.409510000	1.862751000	-1.630556000
H	-6.359800000	1.741724000	-0.168246000
C	-7.504161000	2.307017000	-1.909285000
H	-7.516527000	2.077130000	-2.984106000
H	-8.469015000	1.960555000	-1.512877000
C	-7.379821000	3.820232000	-1.711179000
H	-6.441589000	4.196693000	-2.133308000
H	-8.205520000	4.357083000	-2.192360000
H	-7.386981000	4.081774000	-0.646287000
C	-2.739167000	-0.155058000	-2.561390000
H	-3.343789000	0.726761000	-2.748624000
H	-1.896897000	-0.299096000	-3.241383000

TS5 [Pd(**DTE^{o}-COCF$_3$**)(**Ph**)**I**]·**Sn(CH$_2$CH)Bu$_3$**

Pd	-1.841621000	0.410348000	-1.298534000
S	2.167115000	0.463298000	2.031772000
S	7.469579000	1.435274000	-0.007771000
P	-0.387073000	1.391306000	0.433576000
F	11.404967000	-1.584479000	1.393574000
F	10.228446000	-2.254529000	-0.312449000
F	9.402718000	-2.385871000	1.699229000
O	10.243642000	0.754953000	1.104086000
C	-1.020874000	1.411268000	2.166393000
C	-1.535455000	0.208621000	2.677046000
H	-1.525964000	-0.683166000	2.061038000
C	-2.052002000	0.150148000	3.970544000
H	-2.448210000	-0.787691000	4.348902000
C	-2.064597000	1.294988000	4.772461000
H	-2.474288000	1.253130000	5.777601000
C	-1.541246000	2.490311000	4.277330000
H	-1.535386000	3.382152000	4.897257000
C	-1.017039000	2.550546000	2.983047000
H	-0.610371000	3.487215000	2.620516000
C	0.126816000	3.125162000	0.103316000
C	-0.841581000	4.142888000	0.136031000
H	-1.865238000	3.909256000	0.409249000
C	-0.500911000	5.450873000	-0.202243000
H	-1.261168000	6.226146000	-0.177153000
C	0.807596000	5.758652000	-0.590268000
H	1.069622000	6.777238000	-0.862564000
C	1.772247000	4.751236000	-0.633265000
H	2.790629000	4.980694000	-0.935936000
C	1.434972000	3.439873000	-0.287618000
H	2.189944000	2.662827000	-0.334805000
C	1.175774000	0.489145000	0.588223000
C	1.773415000	-0.235243000	-0.409924000
H	1.324409000	-0.360228000	-1.388433000
C	3.035383000	-0.813250000	-0.046488000
C	3.377952000	-0.533814000	1.265530000
C	4.571204000	-0.990881000	2.047299000
H	4.986305000	-1.896315000	1.598220000
H	4.310535000	-1.208536000	3.087811000
H	5.362341000	-0.231965000	2.052641000
C	3.805828000	-1.643539000	-0.983339000
H	2.882613000	-3.582989000	-1.038119000
C	4.187381000	-3.206489000	-2.775943000
H	4.021075000	-2.664054000	-3.713303000
C	5.532868000	-2.759227000	-2.164384000
C	5.133295000	-1.613919000	-1.246181000
C	6.149513000	-0.684347000	-0.732739000
C	7.414580000	-1.137239000	-0.263532000
H	7.692592000	-2.183441000	-0.242052000
C	8.242887000	-0.124071000	0.178192000
C	6.039782000	0.702671000	-0.670156000
C	4.892563000	1.566171000	-1.087053000
H	4.282692000	1.045119000	-1.828484000
H	5.233288000	2.515309000	-1.511685000
H	4.250085000	1.792078000	-0.228743000
C	9.574667000	-0.200098000	0.738402000
C	10.170246000	-1.627838000	0.884160000
C	3.127302000	-2.773323000	-1.741554000
H	2.179242000	-2.467009000	-2.197976000
H	4.153239000	-4.275997000	-3.000924000
H	5.997700000	-3.564718000	-1.576411000
H	6.268488000	-2.455837000	-2.917350000
I	-1.339954000	-2.268450000	-0.507146000
C	-2.088941000	2.232279000	-2.158995000
C	-3.229255000	3.035267000	-2.026446000
C	-1.021536000	2.718584000	-2.934306000
C	-3.288604000	4.305155000	-2.613982000
C	-1.082696000	3.982340000	-3.529504000

C	-2.214914000	4.785930000	-3.365971000
H	-4.080382000	2.687533000	-1.454051000
H	-0.121774000	2.121726000	-3.065308000
H	-4.178745000	4.916074000	-2.478669000
H	-0.239175000	4.340143000	-4.115671000
H	-2.258821000	5.771896000	-3.820742000
C	-2.991700000	-0.131512000	-2.904834000
Sn	-4.164666000	-1.084653000	-0.105518000
H	-3.910867000	0.427885000	-3.096540000
C	-5.170898000	-2.293429000	-1.614131000
H	-5.561487000	-1.620673000	-2.384559000
H	-4.402579000	-2.912628000	-2.086701000
C	-5.136137000	0.850283000	0.222268000
H	-6.108309000	0.546680000	0.635661000
H	-5.334683000	1.294181000	-0.757210000
C	-4.205421000	-2.135839000	1.813546000
H	-4.172062000	-3.205685000	1.573497000
H	-3.295406000	-1.909498000	2.373036000
C	-5.450757000	-1.796200000	2.645311000
H	-5.484669000	-0.716849000	2.850245000
H	-6.363838000	-2.027490000	2.077516000
C	-5.498778000	-2.551466000	3.982483000
H	-5.438580000	-3.630731000	3.788181000
H	-4.605758000	-2.294311000	4.568594000
C	-6.758366000	-2.242231000	4.796474000
H	-6.766020000	-2.783271000	5.749399000
H	-6.831538000	-1.171323000	5.020406000
H	-7.662450000	-2.526943000	4.245272000
C	-4.437246000	1.834868000	1.162510000
H	-3.453602000	2.098403000	0.758996000
H	-4.243545000	1.358717000	2.132594000
C	-5.239137000	3.124498000	1.387108000
H	-5.423397000	3.606519000	0.416718000
H	-6.226158000	2.871188000	1.797869000
C	-4.526512000	4.107406000	2.321116000
H	-3.561309000	4.418334000	1.904959000
H	-5.123907000	5.011304000	2.485856000
H	-4.329256000	3.650912000	3.298008000
C	-6.289872000	-3.160149000	-1.018184000
H	-7.053041000	-2.527143000	-0.542241000
H	-5.888866000	-3.803836000	-0.222900000
C	-6.972133000	-4.047147000	-2.072055000
H	-7.384103000	-3.409207000	-2.866054000
H	-6.211877000	-4.680456000	-2.549485000
C	-8.081174000	-4.924742000	-1.485086000
H	-8.868050000	-4.313857000	-1.025883000
H	-8.550284000	-5.548089000	-2.254857000
H	-7.686899000	-5.593226000	-0.709948000
C	-2.622067000	-1.037943000	-3.821129000
H	-1.719818000	-1.639378000	-3.723532000
H	-3.221357000	-1.225409000	-4.714936000

TS5 [Pd(**DTE^c-COCF$_3$**)(**Ph**)**I**]·**Sn(CH$_2$CH)Bu$_3$**

Pd	-1.589888000	-0.070767000	-1.124699000
I	-1.631622000	-2.263898000	0.676243000
C	-1.369990000	1.286239000	-2.618207000
C	-2.139990000	2.453781000	-2.719573000
C	-0.349059000	1.085484000	-3.563975000
C	-1.884394000	3.403604000	-3.715017000
C	-0.085227000	2.039255000	-4.553760000
C	-0.851449000	3.204403000	-4.633686000
H	-2.925432000	2.654472000	-2.002152000
H	0.253259000	0.181431000	-3.535595000
H	-2.486742000	4.308080000	-3.757580000
H	0.718396000	1.864618000	-5.265895000
H	-0.646839000	3.946029000	-5.401146000
C	-2.510435000	-1.125303000	-2.621606000
Sn	-4.334900000	-1.038054000	0.029010000
H	-3.267267000	-0.608398000	-3.218211000
C	-5.151730000	-2.794194000	-0.965351000
H	-5.360966000	-2.513514000	-2.003071000
H	-4.356340000	-3.544804000	-0.987919000
C	-4.974114000	0.862710000	-0.845852000
H	-6.064557000	0.737285000	-0.925141000
H	-4.581800000	0.942556000	-1.862824000
C	-4.869771000	-0.891388000	2.141619000
H	-4.657767000	-1.850835000	2.625505000
H	-4.216704000	-0.144710000	2.605018000
C	-6.343417000	-0.488558000	2.320208000
H	-6.558660000	0.427229000	1.751691000
H	-7.001491000	-1.264840000	1.906495000
C	-6.723977000	-0.248250000	3.789307000
H	-6.524643000	-1.161186000	4.366682000
H	-6.067903000	0.529014000	4.204903000
C	-8.188372000	0.165622000	3.961592000
H	-8.438137000	0.333921000	5.015166000
H	-8.403219000	1.092827000	3.416269000
H	-8.865645000	-0.607038000	3.578013000
C	-4.639135000	2.106729000	-0.017301000
H	-3.550404000	2.246354000	0.019006000
H	-4.961571000	1.975000000	1.024746000
C	-5.297042000	3.376992000	-0.579308000
H	-5.019449000	3.489902000	-1.636431000
H	-6.388289000	3.248825000	-0.562692000
C	-4.910014000	4.642496000	0.189788000
H	-3.831205000	4.822971000	0.124756000
H	-5.419988000	5.526526000	-0.209974000
H	-5.171965000	4.557778000	1.251974000
C	-6.410711000	-3.341444000	-0.276162000
H	-7.199823000	-2.576445000	-0.258581000
H	-6.194741000	-3.580881000	0.774303000
C	-6.960152000	-4.599539000	-0.966994000
H	-7.182569000	-4.364563000	-2.016770000
H	-6.175972000	-5.368579000	-0.983165000
C	-8.213778000	-5.153530000	-0.284068000
H	-9.023374000	-4.413597000	-0.282028000
H	-8.584429000	-6.050776000	-0.792299000
H	-8.009173000	-5.422354000	0.759306000
C	-2.177421000	-2.365679000	-3.012809000
H	-1.434717000	-2.966989000	-2.490430000
H	-2.648072000	-2.842736000	-3.875734000
C	5.925443000	-1.171466000	-0.320314000
C	5.150001000	-0.405588000	0.771638000
S	6.468609000	0.600280000	1.658239000
C	7.750203000	-0.414391000	0.944166000
C	7.317520000	-1.248219000	-0.055889000
H	7.980930000	-1.884892000	-0.628894000
C	4.384727000	-3.389069000	-2.847346000
C	3.269373000	-2.335230000	-2.635981000
C	3.824769000	-1.451327000	-1.541456000
C	5.234332000	-1.719540000	-1.364687000

C	5.695315000	-2.669059000	-2.442136000
H	4.415301000	-3.774212000	-3.869595000
H	2.302677000	-2.778816000	-2.377335000
H	3.116174000	-1.740467000	-3.547001000
H	6.485124000	-3.349361000	-2.111794000
H	6.091702000	-2.092231000	-3.289735000
H	4.217050000	-4.236663000	-2.173839000
C	1.818363000	-0.146394000	-0.777474000
C	1.458572000	0.729600000	0.204224000
S	2.801899000	1.216487000	1.259372000
C	4.052573000	0.438832000	0.086087000
C	3.206494000	-0.488031000	-0.803359000
H	1.097345000	-0.575047000	-1.464124000
C	4.603598000	1.577346000	-0.805128000
H	5.294594000	1.171090000	-1.549364000
H	3.777064000	2.062688000	-1.329692000
H	5.130303000	2.328630000	-0.214741000
C	4.594885000	-1.439574000	1.778224000
H	3.858855000	-2.086953000	1.292585000
H	5.414079000	-2.063005000	2.144714000
H	4.120110000	-0.952921000	2.632203000
C	9.104099000	-0.248994000	1.429155000
O	9.435376000	0.533242000	2.311356000
C	10.199600000	-1.124056000	0.763509000
F	11.397240000	-0.893668000	1.311333000
F	9.923449000	-2.441783000	0.897384000
F	10.287912000	-0.866659000	-0.562332000
P	-0.251886000	1.377732000	0.330646000
C	-0.529215000	1.617428000	2.136937000
C	-1.485475000	0.842144000	2.804595000
H	-2.067850000	0.117727000	2.252404000
C	-1.686335000	0.988279000	4.180213000
H	-2.428077000	0.373131000	4.683206000
C	-0.934785000	1.917780000	4.900375000
H	-1.084387000	2.028893000	5.971151000
C	0.005398000	2.714880000	4.238041000
H	0.582634000	3.451498000	4.789457000
C	0.203961000	2.572768000	2.864819000
H	0.916004000	3.218845000	2.360435000
C	-0.078794000	3.115016000	-0.254569000
C	-0.990147000	4.082438000	0.196393000
H	-1.727156000	3.830021000	0.952455000
C	-0.953671000	5.378636000	-0.318885000
H	-1.662892000	6.118872000	0.040729000
C	-0.009861000	5.720863000	-1.290228000
H	0.017685000	6.729809000	-1.691719000
C	0.897639000	4.760410000	-1.743331000
H	1.632185000	5.017664000	-2.500978000
C	0.863831000	3.462820000	-1.231090000
H	1.560883000	2.720067000	-1.603834000

6 [Pd(**DTE^{o}-COCF$_3$**)(**Ph**)(**CH$_2$CH**)(**SnBu$_3$**)**I**]

Pd	-1.847631000	1.014158000	-1.375774000
S	1.999572000	0.574236000	2.054326000
S	7.373463000	1.316859000	0.227450000
P	-0.446028000	1.781475000	0.444009000
F	11.099011000	-2.033813000	1.440513000
F	9.949441000	-2.460028000	-0.359717000
F	9.051304000	-2.757930000	1.602397000
O	10.070433000	0.384051000	1.349314000
C	-1.142481000	1.747432000	2.153353000
C	-2.104395000	0.779717000	2.480847000
H	-2.449643000	0.086499000	1.725065000
C	-2.621878000	0.701714000	3.774655000
H	-3.368754000	-0.050972000	4.009479000
C	-2.186102000	1.592570000	4.758160000
H	-2.591870000	1.535446000	5.764260000
C	-1.226918000	2.558093000	4.442893000
H	-0.881265000	3.252929000	5.203045000
C	-0.706251000	2.636605000	3.149705000
H	0.035166000	3.394463000	2.918698000
C	0.175595000	3.502856000	0.274930000
C	-0.736231000	4.562560000	0.412812000
H	-1.764346000	4.362099000	0.698945000
C	-0.332051000	5.873274000	0.168733000
H	-1.048404000	6.683526000	0.268864000
C	0.985338000	6.142782000	-0.217070000
H	1.296746000	7.165035000	-0.412292000
C	1.895523000	5.094007000	-0.354436000
H	2.920964000	5.294848000	-0.652378000
C	1.492399000	3.778096000	-0.112196000
H	2.202089000	2.967152000	-0.236687000
C	1.053677000	0.770400000	0.593100000
C	1.617558000	0.057981000	-0.433893000
H	1.186042000	0.031624000	-1.428554000
C	2.821874000	-0.638069000	-0.080379000
C	3.149779000	-0.467780000	1.253400000
C	4.286549000	-1.060016000	2.028636000
H	4.655937000	-1.955102000	1.522603000
H	3.980346000	-1.336456000	3.042493000
H	5.124401000	-0.357922000	2.112564000
C	3.561543000	-1.450980000	-1.056437000
H	2.524373000	-3.320784000	-1.273434000
C	3.891445000	-2.899465000	-2.952309000
H	3.779645000	-2.281232000	-3.849908000
C	5.246466000	-2.580576000	-2.283998000
C	4.894235000	-1.483130000	-1.291062000
C	5.951248000	-0.657252000	-0.689892000
C	7.177100000	-1.219472000	-0.235352000
H	7.394297000	-2.278756000	-0.291920000
C	8.051569000	-0.295064000	0.301974000
C	5.920711000	0.724937000	-0.519899000
C	4.838064000	1.686860000	-0.891666000
H	4.216364000	1.262151000	-1.682879000
H	5.246694000	2.641532000	-1.236253000
H	4.189773000	1.888326000	-0.031505000
C	9.362445000	-0.494793000	0.880319000
C	9.881711000	-1.959391000	0.894222000
C	2.834745000	-2.478261000	-1.909321000
H	1.919160000	-2.080135000	-2.361359000
H	3.799076000	-3.945714000	-3.256382000
H	5.649322000	-3.453609000	-1.749042000
H	6.015846000	-2.268044000	-2.998475000
I	-1.649237000	-1.728678000	-0.512983000
C	-1.926476000	2.856200000	-2.186783000
C	-3.070344000	3.662261000	-2.070621000
C	-0.800083000	3.402313000	-2.827398000
C	-3.074791000	4.984723000	-2.530669000
C	-0.806552000	4.718461000	-3.300597000
C	-1.941062000	5.520550000	-3.145269000
H	-3.968478000	3.265702000	-1.607436000

H	0.106264000	2.811769000	-2.934271000
H	-3.968250000	5.593181000	-2.406844000
H	0.083044000	5.120337000	-3.780194000
H	-1.940926000	6.547293000	-3.501544000
C	-3.081980000	0.522378000	-2.927440000
Sn	-4.438181000	-1.973835000	0.026277000
H	-3.935847000	1.189503000	-3.090693000
C	-4.977712000	-3.505049000	-1.414557000
H	-6.052175000	-3.693567000	-1.293158000
H	-4.839833000	-3.051490000	-2.403089000
C	-5.455339000	-0.074728000	-0.270507000
H	-6.495728000	-0.288490000	0.011764000
H	-5.448061000	0.132623000	-1.344862000
C	-4.317609000	-2.557907000	2.123906000
H	-4.326234000	-3.653533000	2.164076000
H	-3.330648000	-2.236501000	2.474476000
C	-5.422163000	-1.968957000	3.013268000
H	-5.412095000	-0.872643000	2.950038000
H	-6.412495000	-2.283284000	2.654941000
C	-5.272945000	-2.378149000	4.486156000
H	-5.320985000	-3.472913000	4.562254000
H	-4.271800000	-2.091480000	4.836688000
C	-6.335461000	-1.746479000	5.389391000
H	-6.210803000	-2.055282000	6.433221000
H	-6.280055000	-0.651513000	5.356824000
H	-7.345537000	-2.035371000	5.074720000
C	-4.914196000	1.118947000	0.521355000
H	-3.897529000	1.350363000	0.172605000
H	-4.822483000	0.870154000	1.585512000
C	-5.777556000	2.380268000	0.391658000
H	-5.907118000	2.625214000	-0.671395000
H	-6.783689000	2.175578000	0.783361000
C	-5.164980000	3.574407000	1.130253000
H	-4.180270000	3.824306000	0.718661000
H	-5.795933000	4.466754000	1.051716000
H	-5.030131000	3.350282000	2.195456000
C	-4.176851000	-4.807046000	-1.294367000
H	-4.319414000	-5.249528000	-0.298335000
H	-3.103111000	-4.592103000	-1.383648000
C	-4.563702000	-5.843960000	-2.359177000
H	-5.636315000	-6.066427000	-2.273969000
H	-4.418782000	-5.403676000	-3.355184000
C	-3.756635000	-7.140153000	-2.245774000
H	-3.905892000	-7.614983000	-1.268411000
H	-4.047782000	-7.863818000	-3.015353000
H	-2.682927000	-6.947337000	-2.358982000
C	-2.977759000	-0.515269000	-3.774103000
H	-2.164784000	-1.238750000	-3.720328000
H	-3.709363000	-0.693653000	-4.566834000

6 [Pd(**DTE^{c}-COCF$_3$**)(**Ph**)(**CH$_2$CH**)(**SnBu$_3$**)**I**]

C	5.400020000	-0.280362000	-0.817042000
C	4.512188000	-0.002133000	0.412695000
S	5.733321000	0.365690000	1.793867000
C	7.056833000	-0.358858000	0.841402000
C	6.733995000	-0.613817000	-0.468188000
H	7.442888000	-0.997603000	-1.192190000
C	4.123716000	-1.002884000	-4.207662000
C	3.041487000	-0.033554000	-3.670867000
C	3.480411000	0.227690000	-2.247021000
C	4.837514000	-0.235408000	-2.062331000
C	5.398408000	-0.664769000	-3.394761000
H	4.276195000	-0.913134000	-5.286122000
H	2.028352000	-0.443098000	-3.736006000
H	3.044802000	0.907398000	-4.237820000
H	6.104536000	-1.496346000	-3.320399000
H	5.931461000	0.179199000	-3.855044000
H	3.825499000	-2.035576000	-3.995331000
C	1.432635000	1.223305000	-1.178687000
C	0.980082000	1.559662000	0.062525000
S	2.196811000	1.446113000	1.343044000
C	3.557887000	1.164928000	0.074745000
C	2.805249000	0.822791000	-1.223824000
H	0.780712000	1.191789000	-2.045288000
C	4.271387000	2.520843000	-0.136135000
H	5.046189000	2.421626000	-0.901961000
H	3.545773000	3.265454000	-0.473640000
H	4.735722000	2.877808000	0.784441000
C	3.775487000	-1.313289000	0.775649000
H	3.082424000	-1.594572000	-0.022884000
H	4.506285000	-2.116374000	0.898314000
H	3.211558000	-1.212265000	1.704717000
C	8.332817000	-0.563874000	1.491456000
O	8.570535000	-0.285081000	2.660570000
C	9.471194000	-1.168432000	0.627331000
F	10.588412000	-1.324077000	1.345319000
F	9.120936000	-2.376958000	0.129461000
F	9.758211000	-0.369823000	-0.427359000
Pd	-2.263198000	1.072778000	-1.237957000
P	-0.753257000	2.041931000	0.385282000
C	-0.981875000	1.576706000	2.155752000
C	-1.499175000	0.308682000	2.460221000
H	-1.776000000	-0.364704000	1.659668000
C	-1.660964000	-0.092192000	3.786796000
H	-2.067295000	-1.075115000	4.004474000
C	-1.309042000	0.771500000	4.826297000
H	-1.439276000	0.462263000	5.859504000
C	-0.789134000	2.034789000	4.533314000
H	-0.509847000	2.709799000	5.337205000
C	-0.623940000	2.437391000	3.206874000
H	-0.219734000	3.421359000	2.993705000
C	-0.691727000	3.877453000	0.428545000
C	-1.818016000	4.563101000	0.913138000
H	-2.658368000	4.009286000	1.320252000
C	-1.871073000	5.953905000	0.856595000
H	-2.752160000	6.472645000	1.222806000
C	-0.801503000	6.676425000	0.317855000
H	-0.846740000	7.760688000	0.269587000
C	0.321610000	6.000179000	-0.160135000
H	1.157835000	6.555152000	-0.576091000
C	0.377568000	4.604725000	-0.108220000
H	1.250927000	4.088344000	-0.494018000
I	-1.034699000	-1.533781000	-0.981263000
C	-3.074072000	2.868775000	-1.651293000
C	-4.333235000	3.238191000	-1.152017000
C	-2.355938000	3.820608000	-2.396330000
C	-4.838453000	4.529289000	-1.346386000
C	-2.865047000	5.106840000	-2.603961000
C	-4.105029000	5.471162000	-2.071217000
H	-4.927727000	2.520224000	-0.595754000
H	-1.376184000	3.573725000	-2.797354000

H	-5.807640000	4.794918000	-0.929387000
H	-2.283951000	5.828110000	-3.174046000
H	-4.494370000	6.474516000	-2.222196000
C	-3.651909000	0.388299000	-2.569806000
Sn	-3.390008000	-2.741229000	0.087236000
H	-4.671005000	0.767561000	-2.435433000
C	-3.768213000	-4.243270000	-1.434694000
H	-4.670572000	-4.788217000	-1.129287000
H	-4.019238000	-3.692091000	-2.348611000
C	-4.980134000	-1.276908000	0.326285000
H	-5.811370000	-1.860226000	0.746528000
H	-5.282772000	-0.955630000	-0.674629000
C	-2.582235000	-3.422120000	1.993740000
H	-2.146961000	-4.415183000	1.832120000
H	-1.753436000	-2.748929000	2.237817000
C	-3.606163000	-3.452675000	3.138245000
H	-4.032171000	-2.451191000	3.287556000
H	-4.451890000	-4.105524000	2.880305000
C	-2.995478000	-3.934490000	4.462806000
H	-2.599928000	-4.950174000	4.326855000
H	-2.132215000	-3.301913000	4.711267000
C	-3.997147000	-3.917174000	5.620454000
H	-3.540203000	-4.268220000	6.552334000
H	-4.378098000	-2.903913000	5.796931000
H	-4.858773000	-4.561653000	5.408288000
C	-4.658186000	-0.073124000	1.216350000
H	-3.870091000	0.525701000	0.738053000
H	-4.250747000	-0.400645000	2.180706000
C	-5.867977000	0.831464000	1.483437000
H	-6.306184000	1.145390000	0.526241000
H	-6.646689000	0.255800000	2.002831000
C	-5.491732000	2.063526000	2.312496000
H	-4.749405000	2.675892000	1.787349000
H	-6.362654000	2.696363000	2.516261000
H	-5.055857000	1.770753000	3.275471000
C	-2.602875000	-5.209062000	-1.681385000
H	-2.360545000	-5.754494000	-0.758435000
H	-1.700392000	-4.642153000	-1.948440000
C	-2.896784000	-6.223555000	-2.796390000
H	-3.795986000	-6.798038000	-2.534524000
H	-3.137318000	-5.679149000	-3.719807000
C	-1.728577000	-7.180040000	-3.050877000
H	-1.486076000	-7.757094000	-2.150249000
H	-1.959149000	-7.892593000	-3.850754000
H	-0.825809000	-6.630669000	-3.344305000
C	-3.474795000	-0.471613000	-3.586431000
H	-2.505041000	-0.905574000	-3.827843000
H	-4.300646000	-0.786843000	-4.229832000

7 [Pd(**DTE°-COCF$_3$**)(**Ph**)(**vinyl**)]

Pd	-3.688645000	1.633919000	-0.331746000
S	0.298460000	-1.330543000	-1.731930000
S	4.817218000	-1.421213000	1.718462000
P	-2.531742000	-0.454339000	-0.597483000
F	9.598979000	-0.960173000	-0.147483000
F	8.567002000	0.890089000	0.358169000
F	7.987134000	-0.230789000	-1.417835000
O	7.738691000	-2.202612000	1.232101000
C	-3.126095000	-1.527413000	-1.961874000
C	-3.905279000	-0.947015000	-2.974134000
H	-4.172894000	0.104961000	-2.912033000
C	-4.343174000	-1.716341000	-4.054188000
H	-4.947417000	-1.259090000	-4.832454000
C	-4.010330000	-3.070693000	-4.127397000
H	-4.353624000	-3.670215000	-4.965724000
C	-3.241107000	-3.657391000	-3.118037000
H	-2.985966000	-4.711943000	-3.170092000
C	-2.801704000	-2.892141000	-2.037437000
H	-2.217909000	-3.359160000	-1.249963000
C	-2.554145000	-1.537837000	0.884707000
C	-3.702033000	-2.296921000	1.162602000
H	-4.523432000	-2.324763000	0.453499000
C	-3.800339000	-3.006572000	2.358970000
H	-4.695966000	-3.584553000	2.567486000
C	-2.757020000	-2.966819000	3.288563000
H	-2.836720000	-3.519131000	4.220530000
C	-1.614564000	-2.210926000	3.017421000
H	-0.799109000	-2.175592000	3.734629000
C	-1.514077000	-1.494802000	1.822687000
H	-0.629832000	-0.897772000	1.624777000
C	-0.775405000	-0.182712000	-0.958656000
C	-0.081261000	0.947435000	-0.602773000
H	-0.552202000	1.786400000	-0.100152000
C	1.319241000	0.905370000	-0.914860000
C	1.673909000	-0.269181000	-1.555154000
C	3.007618000	-0.683772000	-2.096587000
H	3.635005000	0.196179000	-2.256019000
H	2.906760000	-1.214714000	-3.048439000
H	3.535162000	-1.345076000	-1.399421000
C	2.224673000	2.015618000	-0.583848000
H	1.935666000	3.563764000	-2.047783000
C	2.916284000	4.292676000	-0.211199000
H	2.513030000	4.596288000	0.761205000
C	4.104493000	3.332235000	0.011796000
C	3.450449000	1.959070000	-0.012015000
C	4.167829000	0.783918000	0.503879000
C	5.544271000	0.557421000	0.220842000
H	6.134476000	1.222782000	-0.396755000
C	6.048912000	-0.596698000	0.787572000
C	3.638686000	-0.205650000	1.328812000
C	2.252879000	-0.319767000	1.878760000
H	1.775007000	0.662233000	1.893443000
H	2.253998000	-0.727902000	2.893996000
H	1.641742000	-0.978636000	1.251997000
C	7.378380000	-1.161624000	0.703142000
C	8.406750000	-0.356912000	-0.138656000
C	1.864229000	3.444307000	-0.956657000
H	0.834428000	3.703107000	-0.684360000
H	3.194993000	5.200711000	-0.752820000
H	4.844170000	3.407597000	-0.799182000
H	4.642967000	3.523723000	0.946324000
C	-4.865460000	0.958640000	1.113629000
C	-6.133644000	0.440907000	0.810934000
C	-4.397394000	0.891664000	2.435019000
C	-6.903201000	-0.169981000	1.809337000
C	-5.173241000	0.284106000	3.428034000
C	-6.425740000	-0.253301000	3.119188000
H	-6.523676000	0.497044000	-0.201744000
H	-3.419473000	1.286785000	2.693741000
H	-7.878880000	-0.578811000	1.556804000
H	-4.790491000	0.226827000	4.444150000

H	-7.023506000	-0.729373000	3.891634000
C	-4.614080000	3.418252000	-0.188490000
H	-5.681086000	3.513721000	0.035374000
C	-3.931573000	4.545898000	-0.454086000
H	-2.858412000	4.548371000	-0.657926000
H	-4.407858000	5.529640000	-0.488237000

7 [Pd(**DTE^{c}-COCF$_3$**)(**Ph**)(**vinyl**)]

Pd	3.618446000	-1.439421000	-0.872987000
P	2.358548000	0.529117000	-0.308712000
C	2.439517000	1.910029000	-1.513487000
C	2.846108000	1.617879000	-2.824315000
H	3.138499000	0.603610000	-3.084955000
C	2.883862000	2.625725000	-3.790052000
H	3.201205000	2.391839000	-4.802174000
C	2.520956000	3.931081000	-3.451153000
H	2.553365000	4.716116000	-4.201324000
C	2.122297000	4.230109000	-2.144852000
H	1.844325000	5.245731000	-1.878427000
C	2.082482000	3.226219000	-1.177026000
H	1.784398000	3.468678000	-0.161666000
C	2.788995000	1.285071000	1.307285000
C	3.939719000	2.084010000	1.400567000
H	4.503845000	2.337248000	0.508341000
C	4.374414000	2.543230000	2.643345000
H	5.270612000	3.153599000	2.705125000
C	3.667136000	2.212145000	3.802518000
H	4.008887000	2.569732000	4.769582000
C	2.522618000	1.417089000	3.714073000
H	1.966627000	1.157910000	4.610702000
C	2.086034000	0.950006000	2.472580000
H	1.201969000	0.322121000	2.415820000
C	5.142255000	-0.975712000	0.309430000
C	6.254283000	-0.292695000	-0.204549000
C	5.070747000	-1.227403000	1.688066000
C	7.259430000	0.166532000	0.656284000
C	6.080972000	-0.770579000	2.541096000
C	7.175281000	-0.067307000	2.030583000
H	6.339056000	-0.102312000	-1.271018000
H	4.220175000	-1.755609000	2.108606000
H	8.109145000	0.706778000	0.245283000
H	6.004360000	-0.960580000	3.608925000
H	7.955249000	0.291451000	2.696588000
C	4.609224000	-3.110546000	-1.401874000
H	5.694684000	-3.132658000	-1.538713000
C	3.922508000	-4.231272000	-1.684930000
H	2.841863000	-4.308703000	-1.546512000
H	4.401611000	-5.132920000	-2.076209000
C	-4.161606000	-0.991166000	-0.018236000
C	-3.313366000	0.234374000	-0.412430000
S	-4.398002000	1.708858000	0.018025000
C	-5.830953000	0.651079000	0.107689000
C	-5.545076000	-0.690281000	0.090899000
H	-6.298243000	-1.462100000	0.193887000
C	-3.032345000	-4.502155000	-0.246667000
C	-1.707262000	-3.771635000	0.083177000
C	-2.104667000	-2.312213000	0.097271000
C	-3.547274000	-2.201754000	0.134637000
C	-4.143533000	-3.576248000	0.307330000
H	-3.076234000	-5.510259000	0.172864000
H	-0.907048000	-3.989988000	-0.631053000
H	-1.339472000	-4.062020000	1.076782000
H	-5.106479000	-3.697139000	-0.196383000
H	-4.305065000	-3.771897000	1.376831000
H	-3.142159000	-4.585485000	-1.333568000
C	0.100496000	-1.102133000	-0.003256000
C	0.574432000	0.166402000	-0.167688000
S	-0.683774000	1.414973000	-0.210613000
C	-1.978766000	0.174799000	0.363437000
C	-1.323740000	-1.196859000	0.117982000
H	0.750855000	-1.971633000	0.006148000
C	-2.133809000	0.355097000	1.892064000
H	-2.845103000	-0.376639000	2.285910000
H	-1.168411000	0.193611000	2.378308000
H	-2.488254000	1.355653000	2.144182000
C	-3.136777000	0.216834000	-1.948559000
H	-2.552316000	-0.656067000	-2.254101000
H	-4.118568000	0.157869000	-2.424748000
H	-2.631598000	1.116476000	-2.304715000
C	-7.132429000	1.274108000	0.236667000
O	-7.320899000	2.482955000	0.280206000
C	-8.357935000	0.327206000	0.336955000
F	-9.495703000	1.022767000	0.429539000
F	-8.444338000	-0.477775000	-0.746990000
F	-8.265326000	-0.473342000	1.424153000

SnIBu$_3$

I	1.169164000	-1.383911000	-1.741181000
Sn	-0.035112000	0.415328000	0.084233000
C	1.571952000	1.802191000	0.547585000
H	1.827317000	2.317440000	-0.385380000
H	1.146882000	2.556133000	1.222777000
C	-0.637727000	-0.897566000	1.708850000
H	-1.015381000	-0.251778000	2.512139000
H	0.268384000	-1.382892000	2.088465000
C	-1.706879000	1.226004000	-1.043259000
H	-1.294069000	1.822424000	-1.864446000
H	-2.226469000	0.373517000	-1.494627000
C	-2.671563000	2.062511000	-0.188095000
H	-3.060836000	1.458767000	0.643906000
H	-2.138127000	2.906410000	0.271918000
C	-3.856790000	2.608808000	-0.998510000
H	-3.472755000	3.218022000	-1.828033000
H	-4.393383000	1.767401000	-1.457700000
C	-4.825227000	3.439371000	-0.152001000
H	-5.660458000	3.817027000	-0.752287000
H	-5.246396000	2.843094000	0.666485000
H	-4.319002000	4.303497000	0.295097000
C	-1.689953000	-1.939926000	1.307718000
H	-1.308542000	-2.555875000	0.482109000
H	-2.590068000	-1.439156000	0.923853000
C	-2.090726000	-2.859025000	2.470891000
H	-1.193420000	-3.365366000	2.852284000
H	-2.473550000	-2.246638000	3.299065000
C	-3.139736000	-3.900274000	2.071047000
H	-2.768895000	-4.544487000	1.264778000
H	-3.408864000	-4.544814000	2.915377000
H	-4.058086000	-3.419063000	1.713270000
C	2.813926000	1.151640000	1.171160000
H	3.206273000	0.376810000	0.498925000

H	2.543010000	0.637752000	2.104079000
C	3.928641000	2.165663000	1.467379000
H	4.203155000	2.678255000	0.535226000
H	3.540845000	2.941449000	2.142062000
C	5.171441000	1.518682000	2.084995000
H	5.595229000	0.760245000	1.415831000
H	5.953017000	2.260081000	2.285529000
H	4.929939000	1.024149000	3.033679000

TS7 [Pd(**DTE^{o}-COCF$_3$**)(**Ph**)(**vinyl**)]

Pd	3.669729000	-1.121465000	-1.109665000
S	-0.328542000	2.214633000	-0.467744000
S	-4.709156000	-0.251870000	2.247583000
P	2.513018000	0.795665000	-0.397987000
F	-9.592962000	0.660684000	0.878492000
F	-8.573662000	-0.962439000	-0.156239000
F	-8.070349000	1.107581000	-0.613643000
O	-7.631579000	0.583262000	2.627086000
C	3.129131000	2.434530000	-0.959637000
C	3.801769000	2.501695000	-2.188935000
H	3.976671000	1.590693000	-2.756444000
C	4.250645000	3.729160000	-2.681394000
H	4.771737000	3.771434000	-3.633662000
C	4.036601000	4.897109000	-1.945793000
H	4.389676000	5.851585000	-2.325943000
C	3.374284000	4.836516000	-0.716000000
H	3.210939000	5.742624000	-0.139397000
C	2.922927000	3.611813000	-0.222573000
H	2.421437000	3.571735000	0.739965000
C	2.624230000	0.879875000	1.436759000
C	3.857205000	1.219772000	2.017954000
H	4.686951000	1.531220000	1.390266000
C	4.029876000	1.141273000	3.398837000
H	4.990869000	1.398890000	3.834599000
C	2.976848000	0.719329000	4.216148000
H	3.114767000	0.654042000	5.291698000
C	1.750783000	0.375290000	3.643762000
H	0.927913000	0.045702000	4.272265000
C	1.574497000	0.451406000	2.259727000
H	0.622363000	0.168082000	1.822960000
C	0.734330000	0.850944000	-0.759835000
C	0.018798000	-0.199151000	-1.277867000
H	0.481372000	-1.148520000	-1.528233000
C	-1.386863000	0.054009000	-1.424352000
C	-1.727862000	1.337422000	-1.036513000
C	-3.067236000	2.007265000	-1.067110000
H	-3.721974000	1.499381000	-1.779187000
H	-2.984677000	3.058464000	-1.360761000
H	-3.554849000	1.971593000	-0.085840000
C	-2.314244000	-0.960437000	-1.947512000
H	-2.131057000	-0.971504000	-4.089145000
C	-3.070140000	-2.804695000	-3.300073000
H	-2.644025000	-3.716605000	-2.867287000
C	-4.216536000	-2.303154000	-2.395307000
C	-3.517124000	-1.335578000	-1.452643000
C	-4.176601000	-0.897733000	-0.214118000
C	-5.559217000	-0.562087000	-0.174876000
H	-6.196430000	-0.591354000	-1.049999000
C	-6.006089000	-0.178218000	1.074654000
C	-3.582630000	-0.794988000	1.041856000
C	-2.169491000	-1.089320000	1.431347000
H	-1.727685000	-1.798852000	0.728038000
H	-2.105756000	-1.504775000	2.441475000
H	-1.565711000	-0.175842000	1.402448000
C	-7.324307000	0.246174000	1.493313000
C	-8.414945000	0.265515000	0.386814000
C	-2.016395000	-1.677937000	-3.253749000
H	-0.986730000	-2.050479000	-3.304115000
H	-3.398206000	-3.041746000	-4.315885000
H	-4.985444000	-1.770153000	-2.974354000
H	-4.729196000	-3.111386000	-1.862410000
C	4.892973000	-1.821381000	0.371528000
C	6.181468000	-1.273763000	0.511652000
C	4.277993000	-2.392230000	1.504044000
C	6.814045000	-1.251079000	1.759881000
C	4.911764000	-2.361499000	2.746276000
C	6.181952000	-1.788777000	2.882766000
H	6.690622000	-0.858869000	-0.354097000
H	3.296224000	-2.848982000	1.415792000
H	7.803833000	-0.809694000	1.849764000
H	4.409451000	-2.784736000	3.612847000
H	6.671796000	-1.767381000	3.852364000
C	4.746351000	-2.817994000	-1.439573000
H	5.705592000	-2.684623000	-1.947691000
C	4.139199000	-4.018047000	-1.502229000
H	3.231215000	-4.239542000	-0.943359000
H	4.546107000	-4.836904000	-2.097224000

TS7 [Pd(**DTE^{c}-COCF$_3$**)(**Ph**)(**vinyl**)]

Pd	-3.723900000	1.225815000	-0.965410000
P	-2.284899000	-0.539315000	-0.394674000
C	-2.281583000	-2.017219000	-1.487840000
C	-2.571972000	-1.825155000	-2.847141000
H	-2.827949000	-0.831541000	-3.207503000
C	-2.538441000	-2.903035000	-3.734052000
H	-2.765301000	-2.745487000	-4.784637000
C	-2.221640000	-4.181022000	-3.267551000
H	-2.199704000	-5.021032000	-3.956068000
C	-1.939446000	-4.380780000	-1.913068000
H	-1.697000000	-5.374490000	-1.547383000
C	-1.969136000	-3.305281000	-1.024404000
H	-1.758377000	-3.470445000	0.027724000
C	-2.754613000	-1.191713000	1.259694000
C	-3.960591000	-1.902974000	1.373677000
H	-4.539663000	-2.139419000	0.486008000
C	-4.430329000	-2.292187000	2.627027000
H	-5.367879000	-2.834885000	2.703636000
C	-3.704851000	-1.973951000	3.779148000
H	-4.073804000	-2.275071000	4.755477000
C	-2.508011000	-1.263107000	3.671255000
H	-1.938853000	-1.013205000	4.562361000
C	-2.034260000	-0.869192000	2.417318000
H	-1.107920000	-0.307848000	2.345399000
C	-5.408589000	1.182547000	0.190508000
C	-6.476333000	0.348961000	-0.188670000
C	-5.271382000	1.525642000	1.550156000
C	-7.349506000	-0.170576000	0.773527000
C	-6.142808000	1.001039000	2.504423000
C	-7.184371000	0.146895000	2.123036000
H	-6.623697000	0.099327000	-1.235802000

H	-4.475007000	2.194647000	1.863658000
H	-8.159994000	-0.825147000	0.461900000
H	-6.006842000	1.259070000	3.551783000
H	-7.861364000	-0.258750000	2.869566000
C	-5.130251000	2.672494000	-1.229981000
H	-5.854891000	2.489183000	-2.028163000
C	-4.907168000	3.938698000	-0.831811000
H	-4.268891000	4.176316000	0.017608000
H	-5.379452000	4.787967000	-1.327419000
C	4.227904000	1.025457000	-0.138102000
C	3.385308000	-0.244546000	-0.370518000
S	4.460034000	-1.641423000	0.284988000
C	5.892979000	-0.580043000	0.248662000
C	5.608741000	0.745153000	0.036086000
H	6.361226000	1.524676000	0.042516000
C	3.106669000	4.468541000	-0.874191000
C	1.774414000	3.788655000	-0.472493000
C	2.169867000	2.346218000	-0.245615000
C	3.611227000	2.244455000	-0.166269000
C	4.205797000	3.630576000	-0.175193000
H	3.143332000	5.525301000	-0.598117000
H	0.989730000	3.902907000	-1.226962000
H	1.386012000	4.215073000	0.462496000
H	5.178506000	3.681655000	-0.672059000
H	4.346648000	3.973328000	0.859650000
H	3.238236000	4.400189000	-1.959724000
C	-0.034162000	1.128301000	-0.226261000
C	-0.505736000	-0.151021000	-0.224253000
S	0.752617000	-1.389757000	-0.054704000
C	2.036249000	-0.078622000	0.364184000
C	1.386852000	1.243373000	-0.084380000
H	-0.684647000	1.986237000	-0.364564000
C	2.163469000	-0.040573000	1.905506000
H	2.874164000	0.735274000	2.204701000
H	1.191585000	0.195779000	2.345724000
H	2.504235000	-0.997244000	2.304146000
C	3.237300000	-0.445084000	-1.896682000
H	2.659039000	0.374266000	-2.333860000
H	4.227761000	-0.452625000	-2.358302000
H	2.737779000	-1.386843000	-2.130909000
C	7.190223000	-1.176150000	0.489979000
O	7.376691000	-2.365864000	0.712139000
C	8.414899000	-0.223402000	0.470371000
F	9.549764000	-0.895259000	0.689295000
F	8.523041000	0.407368000	-0.721565000
F	8.303821000	0.733739000	1.420929000

8 [Pd(**DTE^{o}-COCF$_3$**)]·**Styrene**

Pd	3.591888000	-1.703151000	-0.260285000
S	-0.481475000	1.024434000	-1.788445000
S	-4.938260000	1.481651000	1.692108000
P	2.338946000	0.204629000	-0.563526000
F	-9.742616000	1.073955000	-0.126800000
F	-8.771261000	-0.774180000	0.493149000
F	-8.167962000	0.206294000	-1.356445000
O	-7.827972000	2.340695000	1.152041000
C	2.845907000	1.291586000	-1.965630000
C	3.442493000	0.696884000	-3.088286000
H	3.624033000	-0.375122000	-3.092745000
C	3.805180000	1.474431000	-4.189206000
H	4.266384000	1.004437000	-5.053379000
C	3.583858000	2.854208000	-4.174853000
H	3.871721000	3.460452000	-5.029195000
C	2.998095000	3.454145000	-3.057205000
H	2.827867000	4.527033000	-3.040470000
C	2.630293000	2.678457000	-1.955744000
H	2.180019000	3.154481000	-1.089780000
C	2.385812000	1.350701000	0.885221000
C	3.638747000	1.585240000	1.477959000
H	4.526535000	1.101097000	1.081993000
C	3.750727000	2.432562000	2.579875000
H	4.726658000	2.600572000	3.025977000
C	2.612976000	3.045683000	3.113519000
H	2.699264000	3.698493000	3.977661000
C	1.364106000	2.811398000	2.534601000
H	0.474857000	3.282830000	2.944171000
C	1.249987000	1.970646000	1.423871000
H	0.274207000	1.802807000	0.981946000
C	0.566565000	-0.059091000	-0.891012000
C	-0.148342000	-1.144507000	-0.451720000
H	0.305860000	-1.946873000	0.121030000
C	-1.543630000	-1.112804000	-0.787403000
C	-1.874819000	0.002764000	-1.535676000
C	-3.197784000	0.383441000	-2.126737000
H	-3.836353000	-0.499058000	-2.211447000
H	-3.081566000	0.823883000	-3.122187000
H	-3.723101000	1.112556000	-1.498807000
C	-2.472358000	-2.177011000	-0.378468000
H	-2.226370000	-3.828002000	-1.732745000
C	-3.223762000	-4.403322000	0.149123000
H	-2.827502000	-4.650305000	1.140282000
C	-4.386217000	-3.398518000	0.305830000
C	-3.695407000	-2.048740000	0.186660000
C	-4.373451000	-0.818590000	0.620391000
C	-5.740082000	-0.559313000	0.320901000
H	-6.354642000	-1.239833000	-0.255055000
C	-6.201211000	0.646416000	0.813533000
C	-3.806913000	0.200337000	1.382822000
C	-2.418121000	0.290136000	1.928617000
H	-2.001010000	-0.711761000	2.054880000
H	-2.393043000	0.808747000	2.891387000
H	-1.764454000	0.830534000	1.235970000
C	-7.508939000	1.253224000	0.694516000
C	-8.571769000	0.431123000	-0.085553000
C	-2.150119000	-3.636368000	-0.652288000
H	-1.126514000	-3.901795000	-0.363680000
H	-3.527443000	-5.338647000	-0.329052000
H	-5.129886000	-3.510270000	-0.497366000
H	-4.927090000	-3.510920000	1.251837000
C	6.297815000	-1.089405000	0.806551000
C	6.985323000	0.034498000	0.311705000
C	6.135076000	-1.207100000	2.200781000
C	7.476895000	1.015628000	1.173337000
C	6.627728000	-0.228703000	3.060550000
C	7.298788000	0.890526000	2.553394000
H	7.119633000	0.142193000	-0.761950000
H	5.606414000	-2.061257000	2.612257000
H	7.997870000	1.877747000	0.765995000
H	6.485265000	-0.336284000	4.132413000
H	7.679742000	1.652390000	3.227483000
C	5.755409000	-2.071362000	-0.159206000

H	6.113153000	-1.930828000	-1.179442000
C	5.086228000	-3.258150000	0.133710000
H	4.950136000	-3.602568000	1.157096000
H	5.003923000	-4.025027000	-0.634012000

8 [Pd(**DTE^c-COCF$_3$**)]·**Styrene**

Pd	-3.448200000	1.126844000	-1.230422000
P	-2.228000000	-0.522079000	-0.192580000
C	-2.206479000	-2.176720000	-1.006656000
C	-2.336102000	-2.218569000	-2.404359000
H	-2.476991000	-1.292804000	-2.957095000
C	-2.289442000	-3.438160000	-3.080778000
H	-2.388454000	-3.459560000	-4.162410000
C	-2.126021000	-4.627998000	-2.366365000
H	-2.096510000	-5.578299000	-2.891918000
C	-2.007322000	-4.594735000	-0.974744000
H	-1.883716000	-5.518011000	-0.415792000
C	-2.045944000	-3.375559000	-0.295029000
H	-1.952006000	-3.361357000	0.786174000
C	-2.718689000	-0.879591000	1.550566000
C	-4.083889000	-0.782897000	1.867490000
H	-4.799929000	-0.495629000	1.103518000
C	-4.527500000	-1.046586000	3.164020000
H	-5.586276000	-0.965264000	3.391915000
C	-3.613044000	-1.396031000	4.160860000
H	-3.957256000	-1.592340000	5.172445000
C	-2.252832000	-1.487018000	3.854688000
H	-1.536725000	-1.757375000	4.625712000
C	-1.805077000	-1.233456000	2.556257000
H	-0.746837000	-1.318573000	2.332816000
C	-6.341893000	1.215332000	-0.534972000
C	-7.055634000	0.007722000	-0.424864000
C	-6.381163000	2.112416000	0.550466000
C	-7.767912000	-0.304605000	0.733672000
C	-7.092470000	1.800591000	1.706062000
C	-7.787211000	0.589067000	1.807315000
H	-7.035262000	-0.696077000	-1.253422000
H	-5.839861000	3.051716000	0.493836000
H	-8.305806000	-1.246445000	0.797599000
H	-7.104676000	2.503652000	2.534501000
H	-8.339415000	0.348766000	2.711351000
C	-5.562662000	1.468987000	-1.767033000
H	-5.764796000	0.762890000	-2.572563000
C	-4.829440000	2.610503000	-2.074687000
H	-4.816188000	3.478613000	-1.418547000
H	-4.541144000	2.796448000	-3.107257000
C	4.285721000	1.089265000	-0.051442000
C	3.443182000	-0.152467000	-0.403658000
S	4.556212000	-1.605716000	0.026583000
C	5.979556000	-0.529549000	0.061656000
C	5.673318000	0.808416000	0.029915000
H	6.418335000	1.591660000	0.102369000
C	3.105824000	4.584546000	-0.302620000
C	1.799120000	3.842775000	0.072541000
C	2.215891000	2.388506000	0.094847000
C	3.657636000	2.295311000	0.095649000
C	4.242932000	3.678896000	0.231706000
H	3.149505000	5.598673000	0.102485000
H	0.978478000	4.041294000	-0.624110000
H	1.453130000	4.143481000	1.070991000
H	5.189818000	3.803131000	-0.300993000
H	4.433145000	3.891074000	1.293350000
H	3.184735000	4.654959000	-1.393166000
C	0.024429000	1.147082000	0.065467000
C	-0.439126000	-0.130612000	-0.057878000
S	0.835654000	-1.361994000	-0.112905000
C	2.126934000	-0.094787000	0.402944000
C	1.447436000	1.263474000	0.151784000
H	-0.636758000	2.008042000	0.053289000
C	2.320275000	-0.242989000	1.931526000
H	3.035412000	0.501846000	2.292466000
H	1.365579000	-0.075802000	2.436953000
H	2.689213000	-1.235247000	2.195558000
C	3.226421000	-0.165059000	-1.934614000
H	2.623290000	0.694875000	-2.240734000
H	4.194695000	-0.103586000	-2.437407000
H	2.723452000	-1.077086000	-2.261432000
C	7.289125000	-1.133353000	0.170364000
O	7.496291000	-2.339717000	0.228816000
C	8.503912000	-0.169693000	0.229540000
F	9.652943000	-0.849257000	0.307306000
F	8.557901000	0.621027000	-0.867376000
F	8.426453000	0.646572000	1.306787000

9 [Pd(**DTE^o-COCF$_3$**)]

Pd	4.507364000	1.929860000	-0.922858000
S	0.922959000	-0.636106000	1.544000000
S	-4.009181000	-1.810477000	-1.190099000
P	3.536350000	0.155235000	-0.076135000
F	-8.594726000	-1.150803000	1.068862000
F	-7.752871000	0.578465000	0.046535000
F	-6.917840000	-0.075780000	1.949419000
O	-6.804039000	-2.590722000	-0.206272000
C	4.267290000	-0.573496000	1.459522000
C	4.968174000	0.270849000	2.333931000
H	5.111025000	1.315252000	2.067657000
C	5.482040000	-0.222179000	3.534652000
H	6.023341000	0.441881000	4.202776000
C	5.307561000	-1.566925000	3.870885000
H	5.712356000	-1.952673000	4.802328000
C	4.616300000	-2.416420000	3.003273000
H	4.480204000	-3.463726000	3.258244000
C	4.097783000	-1.924674000	1.803800000
H	3.566713000	-2.596372000	1.136579000
C	3.457365000	-1.296704000	-1.220679000
C	4.638164000	-2.003597000	-1.506348000
H	5.566524000	-1.732177000	-1.010308000
C	4.625167000	-3.061707000	-2.414469000
H	5.543061000	-3.605708000	-2.619790000
C	3.437160000	-3.420344000	-3.060484000
H	3.429244000	-4.243274000	-3.769597000
C	2.263401000	-2.714572000	-2.790246000
H	1.335850000	-2.985299000	-3.287546000
C	2.272291000	-1.658773000	-1.875028000
H	1.355928000	-1.114849000	-1.673243000
C	1.791473000	0.388420000	0.414248000
C	0.947578000	1.343643000	-0.091388000
H	1.281079000	2.085180000	-0.809526000
C	-0.404554000	1.261030000	0.387532000
C	-0.568819000	0.239679000	1.306327000

C	-1.788520000	-0.142868000	2.087671000
H	-2.483687000	0.698811000	2.131577000
H	-1.533449000	-0.434906000	3.111396000
H	-2.316227000	-0.984025000	1.623226000
C	-1.445629000	2.200168000	-0.055165000
H	-1.138370000	4.052630000	0.990794000
C	-2.382718000	4.286537000	-0.814024000
H	-2.126011000	4.381840000	-1.874977000
C	-3.496111000	3.229523000	-0.656309000
C	-2.721285000	1.939705000	-0.428701000
C	-3.394610000	0.641603000	-0.579029000
C	-4.715074000	0.422421000	-0.094498000
H	-5.284127000	1.184651000	0.422695000
C	-5.193132000	-0.852222000	-0.327746000
C	-2.885457000	-0.485300000	-1.220175000
C	-1.558899000	-0.658809000	-1.886973000
H	-1.161533000	0.312971000	-2.188142000
H	-1.629861000	-1.302024000	-2.769256000
H	-0.839456000	-1.111666000	-1.195826000
C	-6.464118000	-1.441549000	0.033349000
C	-7.454979000	-0.512189000	0.787701000
C	-1.179369000	3.697185000	-0.049398000
H	-0.215236000	3.955733000	-0.501406000
H	-2.676431000	5.276214000	-0.453378000
H	-4.133658000	3.441461000	0.215075000
H	-4.161311000	3.171541000	-1.524809000

9 [Pd(**DTE^{c}-COCF$_3$**)]

Pd	-4.398312000	1.454495000	-1.569073000
C	3.255307000	0.957781000	-0.159860000
C	2.278157000	-0.202564000	-0.431883000
S	3.219469000	-1.738365000	0.106753000
C	4.755998000	-0.830373000	0.072632000
C	4.601709000	0.529072000	-0.051673000
H	5.431996000	1.225162000	-0.030197000
C	2.474927000	4.539468000	-0.657691000
C	1.093361000	3.975766000	-0.243308000
C	1.344394000	2.488558000	-0.120666000
C	2.765066000	2.234507000	-0.101776000
C	3.503437000	3.549463000	-0.057123000
H	2.632718000	5.567038000	-0.320389000
H	0.300197000	4.217708000	-0.957887000
H	0.783016000	4.380350000	0.729871000
H	4.456548000	3.529463000	-0.593029000
H	3.720045000	3.810435000	0.988443000
H	2.560545000	4.527841000	-1.749954000
C	-0.972861000	1.498477000	-0.089507000
C	-1.576423000	0.274220000	-0.124691000
S	-0.446276000	-1.090861000	-0.074532000
C	0.976611000	0.059025000	0.357449000
C	0.452620000	1.462580000	0.004536000
H	-1.534894000	2.422741000	-0.175215000
P	-3.402806000	0.046800000	-0.217983000
C	-3.521375000	-1.741086000	-0.676581000
C	-3.847829000	-2.753356000	0.237244000
C	-3.268393000	-2.083427000	-2.016685000
C	-3.913787000	-4.084504000	-0.182959000
C	-3.320618000	-3.414193000	-2.428676000
C	-3.646978000	-4.418328000	-1.511867000
H	-4.046886000	-2.511598000	1.275901000
H	-3.030380000	-1.302402000	-2.734808000
H	-4.170083000	-4.860285000	0.533139000
H	-3.114707000	-3.666340000	-3.465119000
H	-3.696566000	-5.454589000	-1.833997000
C	-3.883763000	0.087214000	1.566035000
C	-3.020800000	-0.328524000	2.593935000
C	-5.169739000	0.541960000	1.895868000
C	-3.439597000	-0.285508000	3.924578000
C	-5.588926000	0.575549000	3.227404000
C	-4.723438000	0.165435000	4.243972000
H	-2.026413000	-0.694806000	2.361052000
H	-5.838265000	0.874811000	1.105806000
H	-2.762600000	-0.606909000	4.711124000
H	-6.587236000	0.929221000	3.469279000
H	-5.046126000	0.198857000	5.280726000
C	1.151020000	0.000782000	1.894486000
H	1.943010000	0.686908000	2.208113000
H	0.219125000	0.306584000	2.376865000
H	1.408517000	-1.004415000	2.231736000
C	2.061192000	-0.296758000	-1.960039000
H	1.560945000	0.603260000	-2.329770000
H	3.030084000	-0.382080000	-2.458079000
H	1.456740000	-1.165633000	-2.227277000
C	5.988954000	-1.567815000	0.226030000
O	6.061653000	-2.782922000	0.372463000
C	7.303067000	-0.742932000	0.214280000
F	8.372197000	-1.538370000	0.326812000
F	7.428014000	-0.033116000	-0.931386000
F	7.331235000	0.143478000	1.237324000

Styrene

C	-0.515538000	-0.222795000	-0.000002000
C	0.407702000	-1.284288000	-0.000009000
C	-0.012020000	1.092865000	-0.000004000
C	1.782698000	-1.045776000	0.000017000
C	1.360006000	1.331669000	-0.000009000
C	2.265352000	0.264236000	-0.000003000
H	0.038917000	-2.307248000	-0.000007000
H	-0.697631000	1.934932000	0.000000000
H	2.475377000	-1.883001000	0.000045000
H	1.726997000	2.354482000	-0.000002000
H	3.334892000	0.454696000	0.000003000
C	-1.956087000	-0.533097000	-0.000010000
H	-2.189957000	-1.597810000	-0.000056000
C	-2.974585000	0.337329000	0.000021000
H	-2.830639000	1.414724000	0.000033000
H	-4.003115000	-0.011640000	-0.000016000

i [Pd(**DTE^{o}-COCF$_3$**)]·**PhI**

Pd	3.521250000	-0.333356000	0.101178000
S	-0.637712000	2.172059000	-1.323219000
S	-5.335990000	1.060100000	1.769642000
P	2.253530000	1.537495000	-0.141530000
F	-9.891425000	0.050129000	-0.419831000
F	-8.560400000	-1.644436000	-0.104378000
F	-8.134097000	-0.203078000	-1.681214000
O	-8.330922000	1.393304000	1.214334000
C	2.735235000	2.796546000	-1.400197000
C	3.581186000	2.398208000	-2.445566000

H	3.967690000	1.381973000	-2.461889000
C	3.928891000	3.298054000	-3.455243000
H	4.585580000	2.979104000	-4.259831000
C	3.440274000	4.606254000	-3.424658000
H	3.714464000	5.308260000	-4.207118000
C	2.602769000	5.013238000	-2.382101000
H	2.224000000	6.031045000	-2.352407000
C	2.251001000	4.114743000	-1.373669000
H	1.607749000	4.444179000	-0.563178000
C	1.992917000	2.543672000	1.385488000
C	3.013804000	3.404497000	1.822505000
H	3.912427000	3.529389000	1.224102000
C	2.876711000	4.111887000	3.016830000
H	3.669995000	4.780394000	3.339834000
C	1.724947000	3.961312000	3.795888000
H	1.620265000	4.512236000	4.726253000
C	0.711806000	3.098779000	3.372852000
H	-0.186562000	2.975350000	3.971590000
C	0.844148000	2.392856000	2.174465000
H	0.052679000	1.722520000	1.855762000
C	0.559868000	1.083641000	-0.646531000
C	0.020759000	-0.169025000	-0.499877000
H	0.590837000	-1.000444000	-0.099474000
C	-1.354351000	-0.278434000	-0.896584000
C	-1.851080000	0.915741000	-1.387099000
C	-3.206594000	1.214606000	-1.950261000
H	-3.688529000	0.288560000	-2.272664000
H	-3.144908000	1.890450000	-2.809179000
H	-3.857675000	1.683434000	-1.203073000
C	-2.088109000	-1.547552000	-0.786529000
H	-1.479993000	-2.808144000	-2.419351000
C	-2.411040000	-3.934858000	-0.768181000
H	-2.012920000	-4.301237000	0.184864000
C	-3.752212000	-3.214732000	-0.511335000
C	-3.333964000	-1.766461000	-0.303511000
C	-4.262607000	-0.808912000	0.314538000
C	-5.645568000	-0.779030000	-0.021139000
H	-6.085734000	-1.439903000	-0.757375000
C	-6.367028000	0.180471000	0.662144000
C	-3.947837000	0.128331000	1.295901000
C	-2.626374000	0.396000000	1.942018000
H	-1.983734000	-0.483082000	1.858634000
H	-2.738722000	0.653547000	2.999522000
H	-2.116501000	1.228291000	1.444161000
C	-7.772041000	0.514197000	0.575183000
C	-8.610745000	-0.331375000	-0.422529000
C	-1.479696000	-2.835260000	-1.319663000
H	-0.434856000	-2.965316000	-1.014750000
H	-2.506316000	-4.792373000	-1.440027000
H	-4.423769000	-3.289400000	-1.379808000
H	-4.301429000	-3.619540000	0.345709000
I	2.805962000	-3.536428000	0.056928000
C	4.525275000	-2.215641000	0.355394000
C	5.393621000	-1.961774000	-0.737067000
C	4.939278000	-1.919281000	1.680219000
C	6.677113000	-1.456183000	-0.485413000
C	6.228492000	-1.416379000	1.895407000
C	7.099706000	-1.203147000	0.822347000
H	5.096892000	-2.230470000	-1.744905000
H	4.290519000	-2.147708000	2.518486000
H	7.346691000	-1.280548000	-1.322481000
H	6.549471000	-1.207729000	2.911931000
H	8.104541000	-0.834684000	1.005429000

i [Pd(**DTE^c-COCF$_3$**)]·**PhI**

Pd	-3.317228000	-0.330795000	0.357298000
C	4.440808000	-1.195475000	-0.273072000
C	3.739568000	0.010409000	0.382766000
S	5.069853000	1.337578000	0.456535000
C	6.335713000	0.109403000	0.184760000
C	5.854443000	-1.119321000	-0.194489000
H	6.494776000	-1.953998000	-0.453219000
C	2.790412000	-4.395978000	-1.115502000
C	1.634209000	-3.386685000	-1.323589000
C	2.240546000	-2.067007000	-0.899470000
C	3.675409000	-2.200302000	-0.798276000
C	4.082569000	-3.562688000	-1.302319000
H	2.737205000	-5.248737000	-1.797024000
H	0.733553000	-3.644037000	-0.757048000
H	1.345059000	-3.337770000	-2.382360000
H	4.950351000	-3.976311000	-0.781036000
H	4.341803000	-3.494007000	-2.368314000
H	2.756920000	-4.782305000	-0.090747000
C	0.239342000	-0.562459000	-0.626903000
C	-0.061625000	0.683939000	-0.160923000
S	1.353756000	1.636719000	0.324627000
C	2.509542000	0.385501000	-0.472785000
C	1.636238000	-0.866846000	-0.666640000
H	-0.529517000	-1.279805000	-0.895138000
P	-1.786088000	1.299538000	-0.009690000
C	-1.611362000	2.581228000	1.307643000
C	-1.248913000	3.910120000	1.040620000
C	-1.827998000	2.181441000	2.636533000
C	-1.099451000	4.820847000	2.088974000
C	-1.669859000	3.091959000	3.681735000
C	-1.306403000	4.413683000	3.409030000
H	-1.083139000	4.237180000	0.019045000
H	-2.122789000	1.155925000	2.846081000
H	-0.820407000	5.848218000	1.872509000
H	-1.837028000	2.771791000	4.706367000
H	-1.189619000	5.124849000	4.221946000
C	-2.030580000	2.274578000	-1.557700000
C	-0.979742000	2.781440000	-2.338157000
C	-3.356209000	2.507295000	-1.960783000
C	-1.253521000	3.508996000	-3.498753000
C	-3.625834000	3.244107000	-3.114710000
C	-2.574412000	3.743877000	-3.887780000
H	0.051712000	2.617935000	-2.044722000
H	-4.175938000	2.102523000	-1.371985000
H	-0.431982000	3.893651000	-4.096638000
H	-4.655479000	3.418120000	-3.414322000
H	-2.783674000	4.309788000	-4.791166000
C	2.863623000	0.927925000	-1.878609000
H	3.504203000	0.214226000	-2.404755000
H	1.946407000	1.059800000	-2.458368000
H	3.384110000	1.885044000	-1.820715000
C	3.382567000	-0.377192000	1.836575000
H	2.645038000	-1.185219000	1.844683000
H	4.282375000	-0.726143000	2.349237000
H	2.975219000	0.470943000	2.390076000
C	7.717996000	0.507290000	0.331693000

O	8.089686000	1.633054000	0.641954000
C	8.791744000	-0.580733000	0.064422000
F	10.024274000	-0.095527000	0.247401000
F	8.630979000	-1.640387000	0.890673000
F	8.709610000	-1.047965000	-1.203629000
I	-6.150524000	-1.446996000	-0.861569000
C	-4.747484000	-1.880465000	0.755562000
C	-3.784885000	-2.906736000	0.571862000
C	-5.027002000	-1.390279000	2.057562000
C	-3.151530000	-3.457804000	1.694384000
C	-4.375251000	-1.964240000	3.157467000
C	-3.457528000	-3.003890000	2.980446000
H	-3.602442000	-3.312774000	-0.416912000
H	-5.791354000	-0.635218000	2.203911000
H	-2.429802000	-4.257151000	1.552030000
H	-4.606294000	-1.600338000	4.154553000
H	-2.977523000	-3.455810000	3.843164000

TSi [Pd(**DTE^{o}-COCF$_3$**)]·**PhI**

Pd	3.508072000	-0.389879000	0.095727000
S	-0.712601000	2.165077000	-1.274926000
S	-5.371941000	0.931874000	1.829606000
P	2.184636000	1.469173000	-0.144686000
F	-9.947306000	0.092057000	-0.390211000
F	-8.634109000	-1.633198000	-0.185335000
F	-8.195009000	-0.100231000	-1.669694000
O	-8.368145000	1.317560000	1.316511000
C	2.693867000	2.735465000	-1.383167000
C	3.564961000	2.342972000	-2.410333000
H	3.949800000	1.326032000	-2.426391000
C	3.940151000	3.249730000	-3.404001000
H	4.615804000	2.935089000	-4.194404000
C	3.453826000	4.558726000	-3.375535000
H	3.749222000	5.266009000	-4.145384000
C	2.591295000	4.959837000	-2.351181000
H	2.214585000	5.978409000	-2.322865000
C	2.212378000	4.054597000	-1.358961000
H	1.551401000	4.380291000	-0.561355000
C	1.932767000	2.449476000	1.399675000
C	2.938628000	3.329014000	1.833652000
H	3.820374000	3.492620000	1.219864000
C	2.806835000	4.007524000	3.045585000
H	3.587773000	4.691444000	3.366284000
C	1.676097000	3.809191000	3.844079000
H	1.575274000	4.338282000	4.787436000
C	0.678186000	2.927491000	3.423611000
H	-0.203892000	2.766983000	4.037545000
C	0.805232000	2.249885000	2.208797000
H	0.026200000	1.564125000	1.891756000
C	0.487860000	1.044339000	-0.657835000
C	-0.053399000	-0.211597000	-0.559423000
H	0.516458000	-1.060683000	-0.198309000
C	-1.432768000	-0.299370000	-0.946531000
C	-1.930604000	0.916140000	-1.380074000
C	-3.290868000	1.242230000	-1.915983000
H	-3.779741000	0.331537000	-2.270033000
H	-3.235685000	1.951843000	-2.747681000
H	-3.932089000	1.682937000	-1.143548000
C	-2.169946000	-1.569620000	-0.884810000
H	-1.581763000	-2.757559000	-2.577853000
C	-2.504989000	-3.953637000	-0.971304000
H	-2.099228000	-4.367045000	-0.041082000
C	-3.839542000	-3.239455000	-0.666507000
C	-3.411321000	-1.804262000	-0.398208000
C	-4.326872000	-0.870451000	0.273302000
C	-5.711990000	-0.812083000	-0.048983000
H	-6.164044000	-1.431487000	-0.813438000
C	-6.419364000	0.118092000	0.687452000
C	-3.995787000	0.014061000	1.297223000
C	-2.665858000	0.238986000	1.942377000
H	-2.031933000	-0.640454000	1.810098000
H	-2.765525000	0.445968000	3.012147000
H	-2.153803000	1.090281000	1.480111000
C	-7.820841000	0.471275000	0.625253000
C	-8.670871000	-0.302328000	-0.420269000
C	-1.572563000	-2.834447000	-1.480579000
H	-0.525780000	-2.982810000	-1.190967000
H	-2.612157000	-4.777925000	-1.681793000
H	-4.521994000	-3.271452000	-1.529001000
H	-4.380414000	-3.680375000	0.177982000
I	2.862984000	-3.245324000	0.201999000
C	4.788524000	-2.011485000	0.324348000
C	5.584811000	-1.917742000	-0.840804000
C	5.343163000	-1.774327000	1.603340000
C	6.928570000	-1.551963000	-0.711915000
C	6.690093000	-1.410104000	1.697123000
C	7.481280000	-1.305761000	0.548688000
H	5.166543000	-2.157910000	-1.812080000
H	4.740432000	-1.902338000	2.495691000
H	7.544381000	-1.474480000	-1.603754000
H	7.120459000	-1.220695000	2.676645000
H	8.530329000	-1.039368000	0.636868000

TSi [Pd(**DTE^{c}-COCF$_3$**)]·**PhI**

Pd	-3.317747000	-0.453930000	0.274663000
C	4.386386000	-1.097096000	-0.286726000
C	3.673391000	0.092498000	0.386822000
S	4.992340000	1.429251000	0.485060000
C	6.268741000	0.215800000	0.198421000
C	5.799368000	-1.010671000	-0.201697000
H	6.447339000	-1.835863000	-0.471707000
C	2.762914000	-4.298473000	-1.175010000
C	1.600091000	-3.295186000	-1.375518000
C	2.195986000	-1.976789000	-0.933030000
C	3.631582000	-2.100752000	-0.828513000
C	4.049843000	-3.453830000	-1.348218000
H	2.718144000	-5.143835000	-1.866279000
H	0.698611000	-3.564939000	-0.815755000
H	1.315167000	-3.235392000	-2.434913000
H	4.919159000	-3.867499000	-0.829553000
H	4.311241000	-3.370597000	-2.412637000
H	2.728789000	-4.696470000	-0.154755000
C	0.182208000	-0.496256000	-0.648039000
C	-0.135334000	0.735863000	-0.155937000
S	1.267845000	1.692701000	0.357649000
C	2.441266000	0.471807000	-0.464118000
C	1.582140000	-0.785207000	-0.685242000
H	-0.575500000	-1.217603000	-0.936604000
P	-1.871605000	1.309985000	0.015191000
C	-1.742144000	2.506961000	1.410992000

C	-1.389856000	3.853486000	1.234184000
C	-1.980398000	2.022072000	2.707320000
C	-1.270626000	4.697861000	2.340118000
C	-1.854111000	2.867217000	3.810306000
C	-1.499506000	4.206629000	3.627708000
H	-1.209483000	4.245181000	0.238032000
H	-2.264639000	0.981764000	2.847974000
H	-0.998560000	5.739407000	2.194186000
H	-2.038215000	2.482188000	4.809324000
H	-1.406754000	4.866635000	4.485568000
C	-2.138872000	2.366538000	-1.471816000
C	-1.102078000	2.954916000	-2.212907000
C	-3.470120000	2.576607000	-1.868005000
C	-1.396003000	3.742899000	-3.328088000
C	-3.759755000	3.373875000	-2.976033000
C	-2.722804000	3.956470000	-3.709602000
H	-0.067071000	2.807248000	-1.923487000
H	-4.277582000	2.107268000	-1.311421000
H	-0.585934000	4.191536000	-3.896237000
H	-4.793420000	3.530889000	-3.271119000
H	-2.947627000	4.570020000	-4.577436000
C	2.791761000	1.047290000	-1.857268000
H	3.440195000	0.351040000	-2.397126000
H	1.873914000	1.182549000	-2.435275000
H	3.302548000	2.008098000	-1.778418000
C	3.318059000	-0.322795000	1.833439000
H	2.586471000	-1.136245000	1.826731000
H	4.220012000	-0.674079000	2.340707000
H	2.904182000	0.512554000	2.401296000
C	7.647492000	0.623016000	0.356587000
O	8.008123000	1.747865000	0.681984000
C	8.731173000	-0.453641000	0.083883000
F	9.958932000	0.040092000	0.275382000
F	8.575649000	-1.521000000	0.900952000
F	8.657225000	-0.910948000	-1.188197000
I	-6.047021000	-1.015253000	-0.440940000
C	-4.346102000	-2.232537000	0.541617000
C	-3.623267000	-3.128504000	-0.278750000
C	-4.376271000	-2.396142000	1.944684000
C	-2.895745000	-4.159149000	0.325189000
C	-3.638846000	-3.436978000	2.517442000
C	-2.905232000	-4.316502000	1.714510000
H	-3.655076000	-3.031859000	-1.358416000
H	-4.978228000	-1.738077000	2.561661000
H	-2.331672000	-4.846590000	-0.299418000
H	-3.650375000	-3.563268000	3.596554000
H	-2.349273000	-5.129819000	2.171307000

ii [Pd(**DTE^o-COCF$_3$**)(**Ph**)**I**]

Pd	3.694897000	-0.418786000	-0.249680000
S	-0.758742000	2.053497000	-1.334461000
S	-5.366584000	0.774176000	1.726033000
P	2.120542000	1.419588000	-0.206150000
F	-9.923321000	0.025061000	-0.564526000
F	-8.637450000	-1.721202000	-0.363067000
F	-8.156296000	-0.176479000	-1.821987000
O	-8.351322000	1.206106000	1.179951000
C	2.553666000	2.796449000	-1.339783000
C	3.303502000	2.485340000	-2.485131000
H	3.630219000	1.462080000	-2.657979000
C	3.624427000	3.479790000	-3.410399000
H	4.205934000	3.229093000	-4.292887000
C	3.201045000	4.793767000	-3.196410000
H	3.453108000	5.569530000	-3.913688000
C	2.455162000	5.110969000	-2.057783000
H	2.124653000	6.131973000	-1.889940000
C	2.130930000	4.118760000	-1.131178000
H	1.556908000	4.375440000	-0.246222000
C	1.911206000	2.195753000	1.443707000
C	2.913481000	3.046120000	1.939145000
H	3.757593000	3.319439000	1.311702000
C	2.822269000	3.557220000	3.234013000
H	3.597310000	4.221632000	3.605492000
C	1.738461000	3.217279000	4.049387000
H	1.669598000	3.616198000	5.057339000
C	0.746743000	2.361828000	3.564958000
H	-0.097001000	2.091182000	4.193520000
C	0.832724000	1.849382000	2.268695000
H	0.064389000	1.177270000	1.902496000
C	0.452088000	0.941265000	-0.724837000
C	-0.064965000	-0.327378000	-0.649050000
H	0.510687000	-1.169614000	-0.282281000
C	-1.439684000	-0.428692000	-1.048834000
C	-1.951646000	0.785821000	-1.471234000
C	-3.309986000	1.098177000	-2.019973000
H	-3.781702000	0.184118000	-2.387918000
H	-3.254183000	1.816071000	-2.844472000
H	-3.965784000	1.523097000	-1.251230000
C	-2.168320000	-1.705033000	-1.004298000
H	-1.574461000	-2.872250000	-2.709695000
C	-2.499306000	-4.087931000	-1.118153000
H	-2.092401000	-4.512774000	-0.193946000
C	-3.834908000	-3.379781000	-0.803511000
C	-3.409415000	-1.947025000	-0.521312000
C	-4.325371000	-1.018322000	0.156910000
C	-5.705798000	-0.940415000	-0.179868000
H	-6.155770000	-1.543782000	-0.958212000
C	-6.410504000	-0.011668000	0.561070000
C	-3.995133000	-0.151988000	1.196174000
C	-2.668526000	0.048870000	1.856007000
H	-2.042195000	-0.834665000	1.714945000
H	-2.774694000	0.239630000	2.928142000
H	-2.143852000	0.902389000	1.412398000
C	-7.806221000	0.361225000	0.485266000
C	-8.652318000	-0.387214000	-0.581705000
C	-1.567596000	-2.961274000	-1.613262000
H	-0.522031000	-3.111820000	-1.321334000
H	-2.605640000	-4.902906000	-1.839402000
H	-4.519723000	-3.403818000	-1.664388000
H	-4.372658000	-3.830269000	0.037828000
I	2.842182000	-1.822729000	1.817526000
C	5.086997000	-1.798293000	-0.493115000
C	4.912780000	-2.728134000	-1.528662000
C	6.330124000	-1.671194000	0.142923000
C	6.011581000	-3.465801000	-1.986339000
C	7.422025000	-2.413382000	-0.322364000
C	7.263810000	-3.307144000	-1.385755000
H	3.935981000	-2.876467000	-1.983756000
H	6.452476000	-0.998504000	0.987834000
H	5.882046000	-4.174577000	-2.800406000
H	8.390984000	-2.302414000	0.157881000

H	8.110570000	-3.892413000	-1.733490000

ii [Pd(**DTE**[c]**-COCF$_3$**)(**Ph**)**I**]

Pd	-3.686342000	-0.332679000	-0.074460000
C	4.242120000	-1.093444000	-0.336467000
C	3.528358000	0.113181000	0.305246000
S	4.858490000	1.438402000	0.410265000
C	6.127022000	0.209229000	0.164080000
C	5.655184000	-1.018919000	-0.225079000
H	6.300305000	-1.854387000	-0.468822000
C	2.605031000	-4.292599000	-1.194124000
C	1.453995000	-3.283523000	-1.430147000
C	2.054570000	-1.961479000	-1.007059000
C	3.489105000	-2.097320000	-0.877518000
C	3.901738000	-3.463021000	-1.367006000
H	2.561477000	-5.150902000	-1.869223000
H	0.543146000	-3.532866000	-0.876858000
H	1.184365000	-3.242080000	-2.494398000
H	4.760893000	-3.874264000	-0.829950000
H	4.176659000	-3.401733000	-2.429556000
H	2.553462000	-4.669785000	-0.166891000
C	0.049283000	-0.462745000	-0.767476000
C	-0.258045000	0.777323000	-0.294353000
S	1.146898000	1.740110000	0.208057000
C	2.317580000	0.492188000	-0.576251000
C	1.449179000	-0.760922000	-0.789094000
H	-0.709768000	-1.186458000	-1.042564000
P	-1.970160000	1.370097000	-0.125334000
C	-1.901477000	2.474935000	1.338125000
C	-1.526164000	3.824130000	1.243354000
C	-2.208945000	1.924597000	2.592976000
C	-1.455178000	4.610355000	2.394412000
C	-2.133455000	2.715646000	3.740353000
C	-1.757306000	4.057772000	3.642092000
H	-1.291517000	4.259382000	0.276580000
H	-2.500083000	0.880567000	2.669770000
H	-1.163759000	5.653816000	2.316113000
H	-2.371235000	2.284049000	4.708364000
H	-1.702638000	4.673270000	4.535513000
C	-2.245996000	2.495997000	-1.548872000
C	-1.254061000	2.836779000	-2.478414000
C	-3.550360000	2.994544000	-1.715090000
C	-1.563470000	3.670077000	-3.556419000
C	-3.852575000	3.830162000	-2.789917000
C	-2.858639000	4.168264000	-3.713369000
H	-0.242826000	2.461128000	-2.364568000
H	-4.327797000	2.733849000	-0.999733000
H	-0.788167000	3.931633000	-4.270847000
H	-4.862213000	4.212776000	-2.908294000
H	-3.094504000	4.815864000	-4.552834000
C	2.702636000	1.039883000	-1.970836000
H	3.355039000	0.328407000	-2.485426000
H	1.799079000	1.173849000	-2.571217000
H	3.220871000	1.997212000	-1.898206000
C	3.141303000	-0.277575000	1.750748000
H	2.401370000	-1.083291000	1.741915000
H	4.029832000	-0.630646000	2.279932000
H	2.725310000	0.570035000	2.298303000
C	7.507829000	0.606645000	0.341661000
O	7.871094000	1.731584000	0.661265000
C	8.587395000	-0.480503000	0.094877000
F	9.815203000	0.005135000	0.304686000
F	8.408956000	-1.541328000	0.915197000
F	8.530791000	-0.943675000	-1.175757000
I	-2.667130000	-2.195685000	1.492249000
C	-5.275047000	-1.491118000	-0.236211000
C	-6.395636000	-1.199906000	0.554690000
C	-5.382229000	-2.369868000	-1.323370000
C	-7.645891000	-1.717759000	0.195010000
C	-6.637030000	-2.882748000	-1.673387000
C	-7.766670000	-2.555434000	-0.917565000
H	-6.304509000	-0.571075000	1.437126000
H	-4.503508000	-2.648114000	-1.899839000
H	-8.519794000	-1.477690000	0.795359000
H	-6.725804000	-3.549389000	-2.527486000
H	-8.736093000	-2.967073000	-1.184323000
